# Discovery of novel 2,4-diarylaminopyrimidine hydrazone derivatives as potent anti-thyroid cancer agents capable of inhibiting FAK

**DOI:** 10.1080/14756366.2024.2423875

**Published:** 2024-11-19

**Authors:** Hongting Li, Mei-Qi Jia, Zhao-Long Qin, Changliang Lu, Weili Chu, Ze Zhang, Jinbo Niu, Jian Song, Sai-Yang Zhang, Lijun Fu

**Affiliations:** a Department of Thyroid Surgery, the First Affiliated Hospital of Zhengzhou University, Zhengzhou, Henan Province, China; b School of Basic Medical Sciences, Zhengzhou University, Zhengzhou, Henan Province, China; c School of Pharmaceutical Sciences, Institute of Drug Discovery & Development Key, Laboratory of Advanced Drug Preparation Technologies (Ministry of Education), Zhengzhou University, Zhengzhou, Henan Province, China; d Zhengzhou Xingyuan Foreign Language High School, Zhengzhou, Henan Province, China; e Department of Respiratory and Critical Care Medicine, The First Affiliated Hospital of Zhengzhou University, Zhengzhou, Henan Province, China; f The Third Affiliated Hospital of Zhengzhou University, Zhengzhou, China

**Keywords:** Thyroid cancer, 2,4-Diarylaminopyrimidine, Hydrazone, FAK, Antiproliferative activities

## Abstract

In this work, thirty 2,4-diarylaminopyrimidine-based hydrazones were designed, synthesised, and their anti-thyroid cancer activity were explored. The majority of compounds exhibit moderate to excellent cytotoxic activity against FAK overexpressing TPC-1 cells, with IC_50_ values ranging from 0.113 to 1.460 μM. Among them, compound **14f** displayed exceptional anti-proliferative effect against TPC-1 cells (IC_50_ = 0.113 μM) and potent FAK inhibitory potency (IC_50_ = 35 nM). In *silico* studies indicated that compound **14f** could well bind to FAK (Focal Adhesion Kinase) and have favourable pharmacokinetic profiles. In addition, compound **14f** could inhibit the phosphorylation of FAK at Tyr397, Tyr576/577 and Tyr925, and did not affect the expression level of FAK in TPC-1 cells. Compound **14f** was also effective in inhibiting the proliferation and migration of thyroid cancer cells TPC-1. Thus, these novel 4-arylaminopyrimidine hydrazone derivatives exhibited potent anti-thyroid cancer activities through the inhibition of FAK.

## Introduction

The incidence of thyroid cancer, the most prevalent endocrine malignancy, is progressively increasing over the past decades^1^. For the majority of subtypes, the prognosis remains favourable following traditional surgery, TSH suppression therapy and ^131^I treatment. However, there are still a few cases of differentiated thyroid cancer (DTC) that eventually develop resistance to radioactive ^131^I treatment and encounter recurrence or metastasis^2^. In addition, anaplastic thyroid cancer (ATC) is the type with the lowest degree of differentiation^3^. Although its incidence is low, the ATC has a high malignancy and could metastasise to distant sites early on, making it incurable by traditional treatments. Despite significant advancements in thyroid cancer treatment over the past decades, including the approval of various kinase inhibitors, such as VEGFR and RET inhibitors, the efficacy of these drugs remains unsatisfactory for patients with poorly DTC or ATC due to primary and acquired resistance^4–7^. Therefore, there is still an urgent need for new therapeutic strategies and novel chemical small molecules to address the treatment of thyroid cancer.

**Figure 1.**
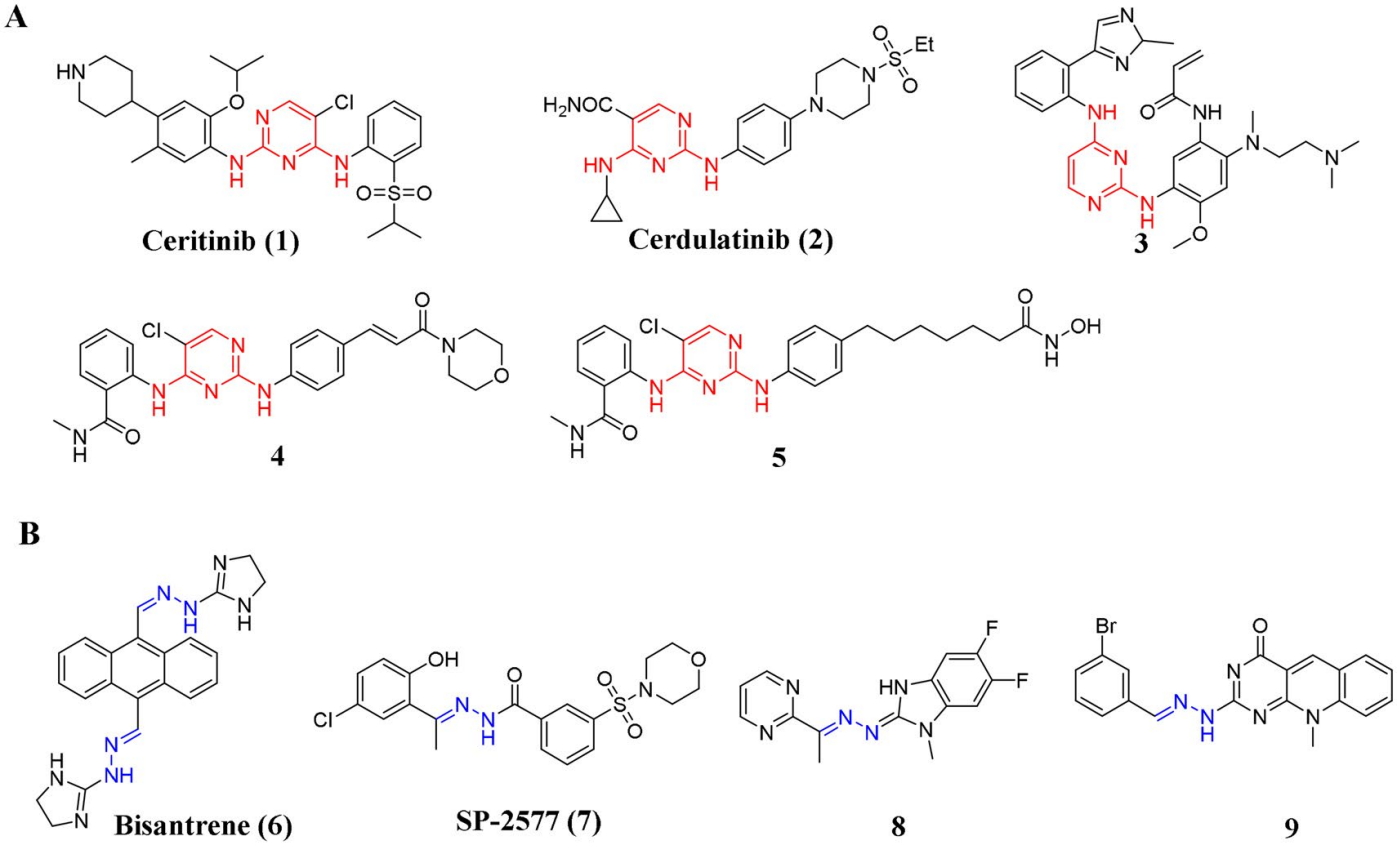
(**A**) Representative and reported 2,4-diaminopyrimidine derivative as anticancer agents; (**B**) Representative and reported anticancer agents bearing a hydrazone moiety.

Recently, the 2,4-diaminopyrimidine moiety is usually used as a core fragment to design novel anticancer agents^8–14^, especially for the discovery of kinase inhibitors, such as ALK inhibitor ceritinib (**1**)^15^, JAK/Syk inhibitor cerdulatinib (**2**)^16^ and EGFR inhibitor **3**^17^ (Figure 1A). Among them, ceritinib has been approved by FDA for the treatment of NSCLC (non-small cell lung cancer) with excellent market performance, and cerdulatinib was also approved to clinical trials for the treatment of Relapsed/Refractory B-cell Malignancies. Further researches have shown that the pyrimidine ring of 2,4-diaminopyrimidine moiety could often form critical hydrogen bonds with kinases, leading to strong inhibitory activity^11^^,^^18^^,^^19^. Importantly, the 2,4-aminopyrimidine moiety also used as the core scaffold for the design of anti-thyroid cancer agents, such as CDK4/6 inhibitor Abemaciclib^20^ and BRAF^V600E^ inhibitor Encorafenib^21^. Therefore, we here designed novel 2,4-diaminopyrimidine derivatives as anti-thyroid cancer agents. In our previous work, we also reported novel 2,4-diaminopyrimidine derivatives **4**^22^ and **5**^23^ as potent anticancer agents. Although compounds **4** and **5** had some advantageous effects on anticancer activities, further structural optimisation was still necessary for the development of new effective anticancer agents. The hydrazone moiety, due to its ability as a hydrogen bond donor and acceptor, has been widely used in drug design, especially in the design of anti-tumor drugs, and this functional group could endow flexible chemical structures to a certain extent^24–31^. For example, Bisantrene (**6**)^32^ has been approved for the treatment of human cancers, and SP-2577 (**7**)^33^ as a LSD1 inhibitor exhibited potent inhibitory effects on acute myelogenous leukaemia. In addition, compound **8**^34^ displayed significant antiproliferative activity against MCF7 cells (IC_50_ = 3.3 nM). 5-Deazaflavin hydrazone derivative **9**^35^ could also effectively inhibit the proliferative activity of MCF7 cells with an IC_50_ value of 0.1 nM (Figure 1B).

**Figure 2.**
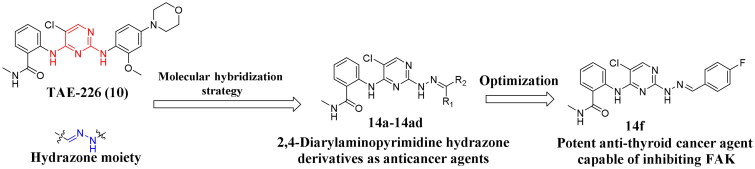
Design strategies for 2,4-diarylaminopyrimidine hydrazone derivatives.

**TAE-226**, as a Focal Adhesion Kinase (FAK) inhibitor, demonstrated high cytotoxicity against multiple types of cancer cells^36^. Although it has never progressed to the clinical trials, the distinctive 2,4-diaminopyrimidine scaffold of **TAE-226** has become the structural foundation for many subsequent anticancer agents, especially FAK inhibitors^37–42^. According to the co-crystallization of **TAE-226** and FAK (PDB:2JKK), the 2-amino junction of the pyrimidine core is closely connected to the DFG motif region of the FAK. And, the methyl carbamoyl moiety of **TAE-226** forms a hydrogen bond. The N atoms at the 1-position of the pyrimidine ring and the amino group connected to 2-position could form hydrogen bonds with Cys502. In addition, the 2-methoxyaniline morpholine moiety delves deep into the solvent region. In this work, we used the molecular hybridisation strategy to insert the hydrazone moiety into **TAE-226** at the C-2 position, and then explored structural modification on the solvent region, thereby obtaining novel 2,4-diarylaminopyrimidine hydrazone derivatives. We further explored the antiproliferative activity against thyroid cancer cells TPC-1 of the obtained compounds as well as the FAK inhibitory activity of the optimal compound (Figure 2). Among them, compound **14f** displayed exceptional antiproliferative effect against TPC-1 cells (IC_50_ = 0.113 μM) and potent FAK inhibitory potency (IC_50_ = 35 nM).

## Chemistry

**Scheme 1.**
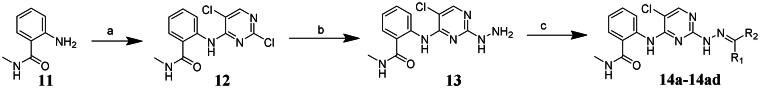
Synthesis of target compounds **14a-14ad**. Reagents and conditions: (a) 2,4,5-trichloropyrimidine, isopropyl alcohol, DIPEA, reflux, 12 h; (b) hydrazine hydrate, ethanol, 25 °C, 4 h. (c) aldehydes or ketones, ethanol, 80 °C, 5 h.

The synthesis of 2,4-diarylaminopyrimidine-based hydrazones is shown in Scheme 1. Through the nucleophilic substitution reaction, commercially available 2-amino-*N*-methylbenzamide (**11**) reacted with 2,4,5-trichloropyrimidine to give intermediate **12** in the presence of DIPEA in isopropyl alcohol, which was converted to the key intermediate **13** through anther substitution reaction with hydrazine hydrate. Finally, target compounds **14a-14ad** were prepared by the condensation reactions of intermediate **3** and a commercially available aldehyde or ketone.

## Results and discussion

### In vitro antiproliferative activity and structure-activity relationships

Considering that compounds **14a-14ad** are designed as anti-thyroid cancer agents, their anti-thyroid cancer cells TPC-1 activity was evaluated with the **TAE-226** as a positive control.

As a general trend, the majority of compounds exhibit moderate to excellent cytotoxic activity against FAK overexpressing TPC-1 cells, with IC_50_ values ranging from 0.113 to 1.460 μM (Table 1). The most promising compound **14f**, demonstrates significant activity in TPC-1 cells with an IC_50_ value of 0.113 μM. In comparison, it is approximately 8.57 times more potent than **TAE-226** (IC_50_ = 1.082 μM). To conduct a preliminary analysis of the structure-activity relationships, we synthesised compounds **14a-14r** with various aromatic substituents (R_1_ group) to investigate the impact of R_1_ substitutions on antiproliferative activity. As shown in Table 1, the results demonstrated these compounds (**14a-14r**) exhibit moderate to potent inhibitory activity against TPC-1 cells, with IC_50_ values ranging from 0.113 to 0.693 μM. When the R_1_ group is a phenyl moiety, the IC_50_ value of **14a** against TPC-1 cells is 0.306 μM. Based on the IC_50_ values of compounds **14a-14r**, analysis of structure-activity relationships reveals that variations in R_1_ substitutions significantly affect the antiproliferative activity against TPC-1 cells. Compared to **14a**, the incorporation of electron-withdrawing groups such as F (**14f**), CF_3_ (**14e**), CN (**14j**), and NO_2_ (**14i**) into the phenyl moiety at the para position leads to an augmentation in the antiproliferative activity of these compounds against TPC-1 cells, with IC_50_ values range from 0.113 to 0.193 μM. However, when electron-withdrawing groups chlorine (**14 g**) and bromine (**14n**) were introduced, a significant reduction in activity was observed with IC_50_ values of 0.409 and 0.590 μM, respectively. Furthermore, the introduction of electron-donating groups at the para position of the phenyl moiety (R_1_ group), such as methyl (**14b**), methoxy (**14c**), or hydroxy (**14d**), could sustain the antiproliferative activity. In addition, the results of the antiproliferative activity of compound 4k-4m further demonstrated that substituent groups positioned ortho of the phenyl moiety (R_1_ group) could significantly mitigate the cytotoxicity towards TPC-1 cells. The same trend was also observed in compounds **14n-14r**. Compared with compound **14c**, the introduction of the additional methoxy (**14o**) or F (**14q**) group at the 3-position of the phenyl moiety (R_1_ group) leads to a decrease in activity, while introducing the methoxy (**14n**) or F (**14p**) group at the 2-position maintains activity. Similarly, based on compound **14f**, maintaining activity could be achieved by introducing a F group at the 2-position of its R_1_ group (**14r**). Further substitution of the phenyl moiety (R_1_ group) with five- or six-membered heterocyclic groups, such as pyridine, thiophene, indole, and quinoline, resulted in the synthesis of compounds **14s-14ab**. Compared to compound **14a**, compounds **14v** and **14 u** containing thiophene and pyridine groups exhibited enhanced inhibitory potency, while replacement with a 2-pyridine (**14s**) or 3-pyridine (**14t**) group maintained activity. Introduction of larger indole moiety (**14x**, **14aa** and **14ab**), substituted indole moiety (**14 y** and **14z**) or quinoline group (**14w**) led to varying degrees of decreased antiproliferative activity against TPC-1 cells. These findings also suggested that an appropriate R_1_ group contributes to the binding affinity between the compound and active pocket of the kinase. As a continuation of SAR research, we synthesised compounds **14ac** and **14ad** by introducing a methyl group into the hydrazone moiety. Regrettably, the incorporation of the methyl group significantly attenuated the antiproliferative activity against TPC-1 cells compared to compounds **14c** and **14f**. Therefore, compound **14f** was consequently selected for further investigation and mechanistic studies.

**Table 1.** Antiproliferative activity **14a∼14ad** and **TAE-226** against thyroid cancer cells TPC-1.

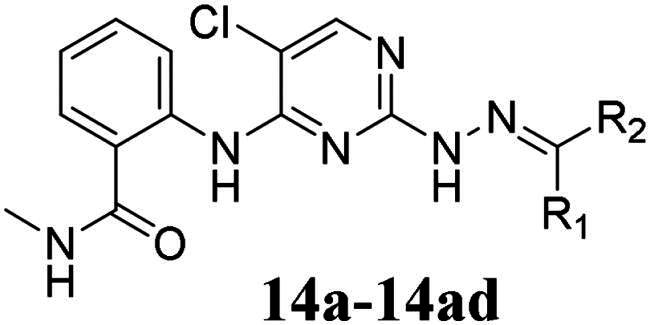							
Compd.	R_1_	R_2_	IC_50_ (μM)^a^	Compd.	R_1_	R_2_	IC_50_ (μM)^a^
**14a**	H	Ph	0.246 ± 0.038	**14q**	H	3-F-4-diOCH_3_-Ph	0.386 ± 0.047
**14b**	H	4-CH_3_-Ph	0.296 ± 0.021	**14r**	H	2, 4-diF-Ph	0.122 ± 0.032
**14c**	H	4-OCH_3_-Ph	0.257 ± 0.004	**14s**	H	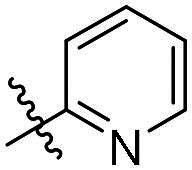	0.272 ± 0.016
**14d**	H	4-OH-Ph	0.255 ± 0.025	**14t**	H	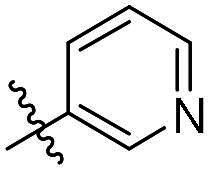	0.243 ± 0.001
**14e**	H	4-CF_3_-Ph	0.193 ± 0.029	**14 u**	H	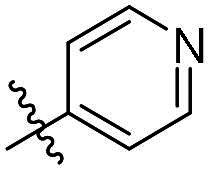	0.189 ± 0.005
**14f**	H	4-F-Ph	0.113 ± 0.003	**14v**	H	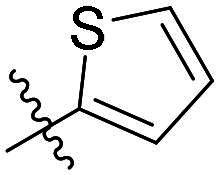	0.153 ± 0.005
**14 g**	H	4-Cl-Ph	0.409 ± 0.093	**14w**	H	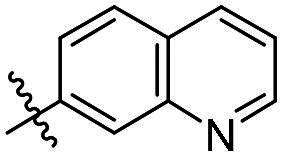	0.413 ± 0.015
**14h**	H	4-Br-Ph	0.590 ± 0.024	**14x**	H	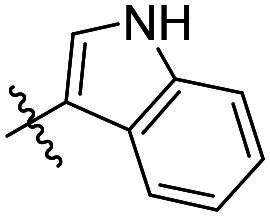	0.357 ± 0.010
**14i**	H	4-NO_2_-Ph	0.157 ± 0.008	**14 y**	H	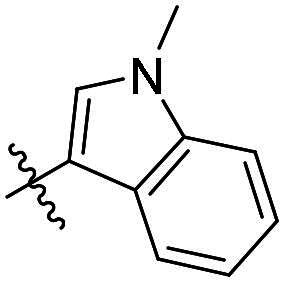	0.425 ± 0.023
**14j**	H	4-CN-Ph	0.126 ± 0.011	**14z**	H	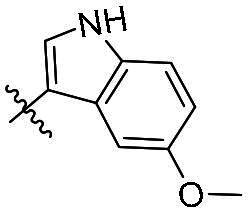	0.624 ± 0.044
**14k**	H	3-OCH_3_-Ph	0.517 ± 0.031	**14aa**	H	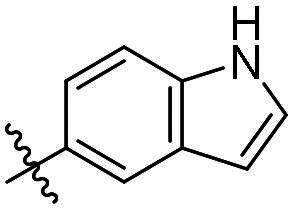	0.331 ± 0.012
**14 l**	H	3-F-Ph	0.428 ± 0.009	**14ab**	H	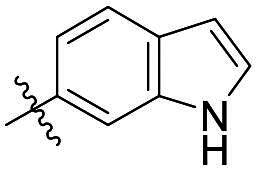	0.364 ± 0.015
**14 m**	H	3-CF_3_-Ph	0.693 ± 0.017	**14ac**	CH_3_	4-OCH_3_-Ph	0.612 ± 0.016
**14n**	H	2, 4-diOCH_3_-Ph	0.263 ± 0.076	**14ad**	CH_3_	4-F-Ph	1.460 ± 0.066
**14o**	H	3, 4-diOCH_3_-Ph	0.434 ± 0.012	**TAE-226**	**–**	**–**	1.082 ± 0.023
**14p**	H	2-F-4-diOCH_3_-Ph	0.228 ± 0.065				

^a^ MTT assay. Cells were treated with compounds or **TAE-226** for 48 h to obtain average IC_50_ values with SD (*n* = 3, duplicate).

### In vitro enzymatic assays and molecular docking study

**Figure 3.**
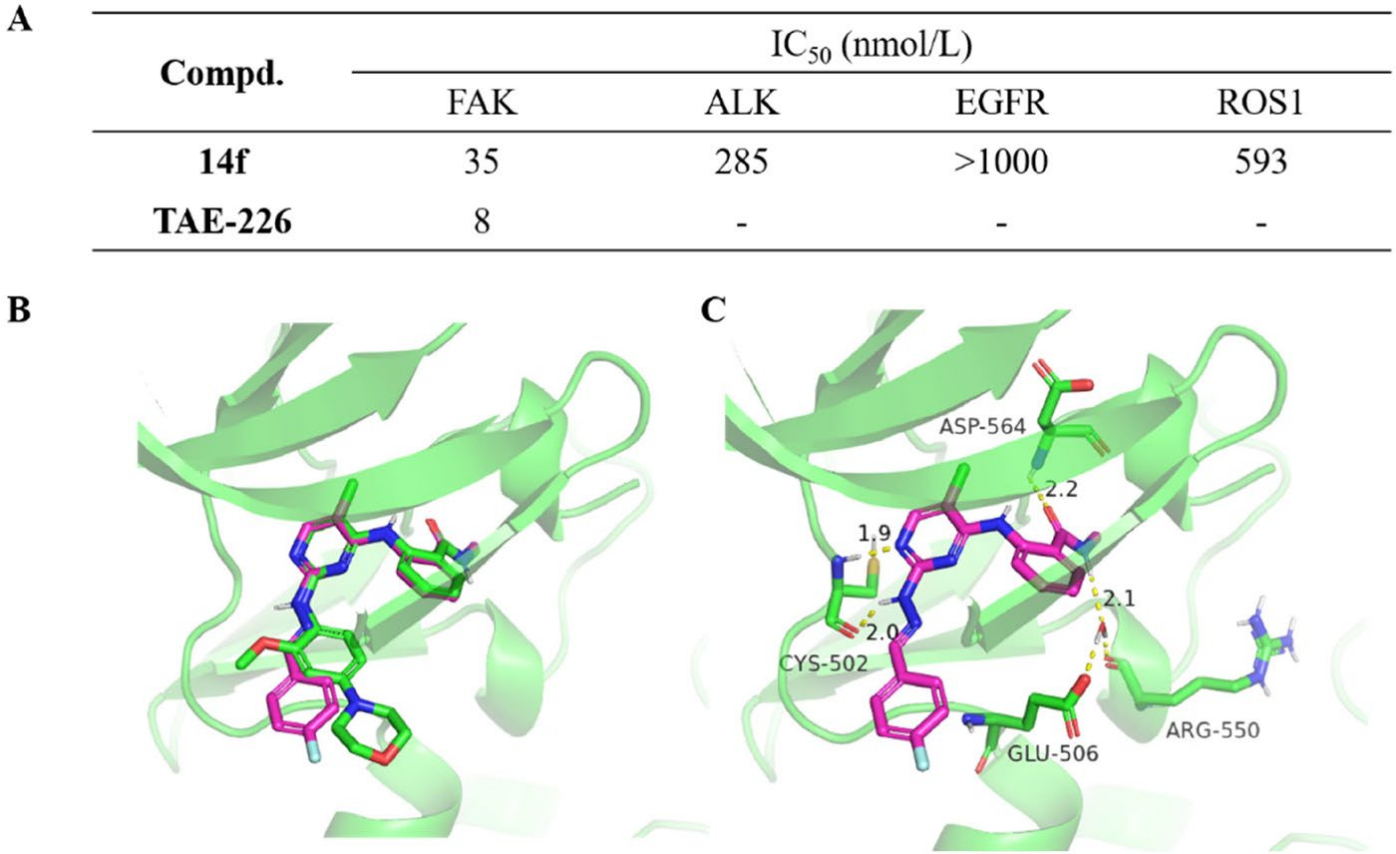
(A) Enzymatic inhibition of compound **14f** and TAE-226; (B) The binding mode comparison between compound **14f** with **TAE-226** (PDB: 2JKK); (C) The proposed binding mode of compound **14f** with FAK.

Given its origin from the FAK inhibitor **TAE-226** and remarkable antiproliferative activity against thyroid cancer cells TPC-1, the inhibitory potential of compound **14f** against FAK was further, evaluated. According to the reported work, the 4-arylaminopyrimidine derivatives possessing a hydrazone moiety might also exhibit certain inhibitory activities against another kinases^31^. Therefore, the activity of compound **14f** against ALK, EGFR, Pyk2, and TYK2 kinases was also tested. The results demonstrated that, although the FAK inhibitory activity exhibited a decrease in comparison to **TAE-226**, compound **14f** still displayed significant inhibition against FAK with an IC_50_ of 35 nM (Figure 3A). Additionally, it exhibited moderate inhibitory activity against four other kinases. Consequently, compound **14f** could be considered as a promising FAK inhibitor for potential applications in antithyroid cancer therapy.

To further elucidate the binding modes between 14f with FAK (PDB ID: 2JKK), we employed molecular docking analysis using MOE 2019. As shown in Figure 3(B), compound **14f** (pink) shared similar binding modes with TAE-226 (Green). It was observed the N atom at the 1-position of the pyrimidine ring and the N atom connected to the 2-position could form two hydrogen bonds with the Cys502 and Asp564. In addition, the methyl carbamoyl moiety also formed a hydrogen-bonding interaction with and Asp564 of FAK. The NH group on the methyl carbamoyl moiety could form hydrogen bonds with Glu506 and Arg550 through water mediation.

### In silico physicochemical properties, ADMET profiles, and drug-likeness data

The effectiveness of newly synthesised compounds is intricately linked to their biological activity, physicochemical, pharmacokinetics, and drug likeness properties, which are crucial for identifying potential candidate drugs.

**Figure 4.**
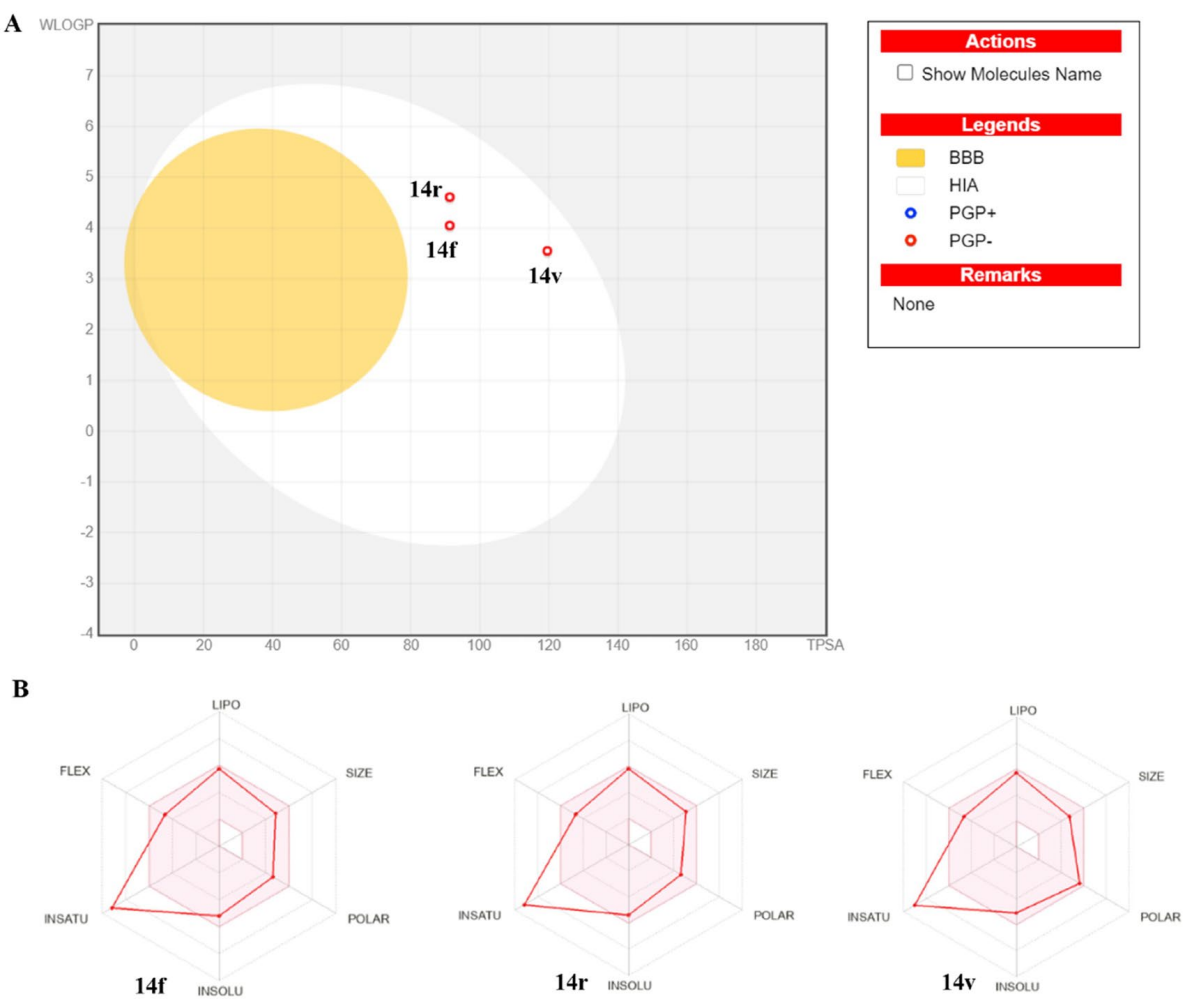
(A) The boiled-egg chart of the compounds **14f**, **14r** and **14v**; (B) The bioavailability radar chart of the compounds **14f**, **14r** and **14v**.

Here, we used the Swiss ADME Online software (www.SwissADME.ch) to measure in the silico ADME profile of the compound **14f**, **14r** and **14v**, which exhibited potent antiproliferative activity against thyroid cancer cells TPC-1. The results indicated that compounds **14f**, **14r** and **14v** exhibited a predicted clogP in a range of 3.44–4.61, moderate water solubility, and high GIT absorption. As shown in Figure 4, the Boiled-Egg chart suggests that these three compounds have GIT absorption, due to their location in the human intestinal absorption (HIA) area. However, due to the high polarity of the hydrazine moiety, compounds **14f**, **14r** and **14v** did not exhibit potential BBB permeability, indicating that these compounds face certain difficulties in reaching the CNS. The oral bioavailability of the compounds **14f**, **14r** and **14v** is predicted in the radar chart (Figure 4). The results indicated the measured physicochemical properties of these three compounds located in the ideal pink area, except for the INSATU parameter.

Furthermore, the metabolism of compounds **14f**, **14r** and **14v** is postulated to inhibit four or five main cytochrome P-450 (CYP) isoforms in the liver (Table 2), suggesting that these three compounds are easy to have potential drug-drug interactions. Moreover, compounds **14f**, **14r** and **14v** are not the substrates of Pg-p (Table 2), which might also exhibit potent activities against Pg-p overexpressing cancer cells. However, these results are based only on chemoinformatic simulations, and additional experiments are needed to verify this prediction.

**Table 2.** The *in silico* predicted ADME profiles for compounds **14f**, **14r** and **14v**.

Compd.	GI absorption	BBB permeability	Pg-p substrate	CYP1A2 inhibitor	CYP2C19 inhibitor	CYP2C9 inhibitor	CYP2D6 inhibitor	CYP3A4 inhibitor
**14f**	High	No	No	Yes	No	Yes	No	Yes
**14r**	High	No	No	Yes	Yes	Yes	No	Yes
**14v**	High	No	No	Yes	Yes	Yes	No	Yes

The physicochemical properties of compounds **14f**, **14r** and **14v** in Table 3 indicated that they have acceptable properties including molecular weight, Log P_o/w_, H-bond acceptors, rotatable bonds, TPSA and Log S, except for the rotatable bonds, which might help to demonstrate good physicochemical properties.

**Table 3.** *In silico* physicochemical properties for compounds **14f**, **14r** and **14v**.

Compd.	MW < 500	Log P_o/w_ < 5 (WLOGP)	HBA < 10	HBD < 5	NRB < 5	TPSA Å^2^ < 160	Log S (ESOL)
**14f**	398.82	4.05	5	3	7	91.30	−5.21
**14r**	386.86	4.61	6	3	7	91.30	−5.38
**14v**	386.86	3.55	4	3	7	119.54	−5.10

MW: molecular weight; Log P_o/w_ (WLOGP): partition coefficient octanol/water; HBA: number of H-bond acceptors; HBD: number of H-bond donors; NRB: number of rotatable bonds; TPSA: topological polar surface area; Log S (ESOL): aqueous solubility.

In addition, the results measured by the Swiss ADME Web-tool indicate that **14f**, **14r** and **14v** have acceptable drug-likeness and conform to the rules (Table 4). Importantly, there are lack of PAINS alert in the view of medicinal chemistry, dedicating that the *in vitro* bioassays of these three compounds might be robustly obtained.

**Table 4.** The drug-likeness of compounds **14f**, **14r** and **14v**.

Compd.	Lipinski #violations	Ghose	Veber	Egan	Muegge	Bioavail. Score	PAINS #alert
**14f**	0	Yes	Yes	Yes	Yes	0.55	0
**14r**	0	Yes	Yes	Yes	Yes	0.55	0
**14v**	0	Yes	Yes	Yes	Yes	0.55	0

### Effects of compound 14f on the FAK autophosphorylation in thyroid cancer cells TPC-1

**Figure 5.**
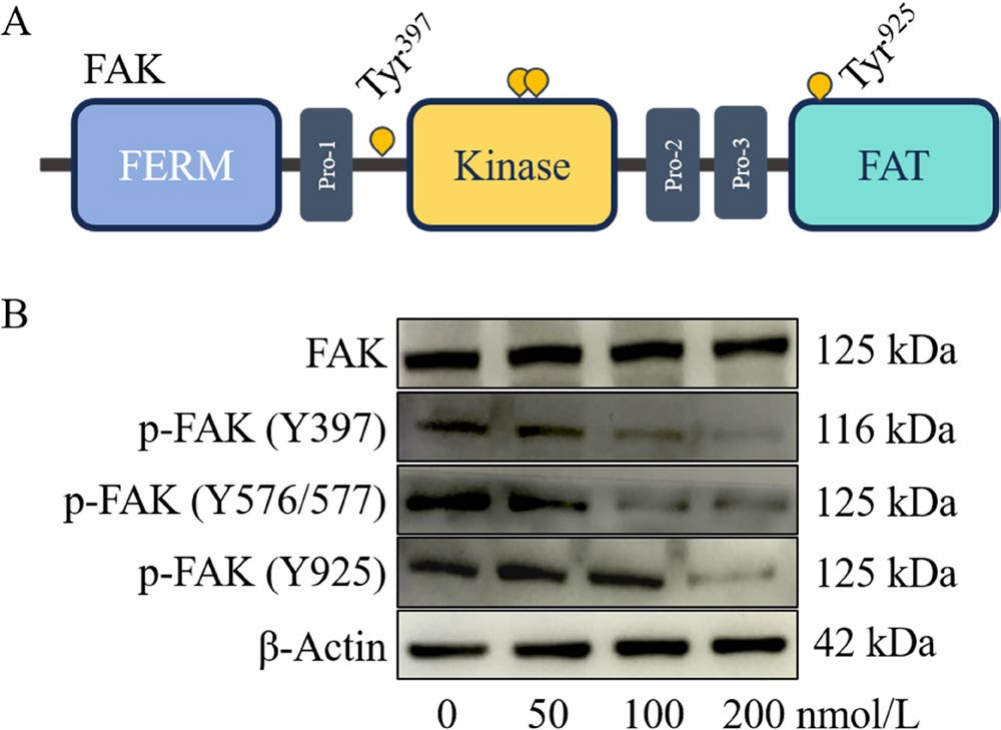
The structural diagram of the FAK protein (**A**) and the inhibitory effect of compound **14f** on FAK (**B**). In Figure 5(B), thyroid cancer cell TPC-1 was treated with DMSO or indicated concentrations of compound **14f** (50, 100 and 200 nmol/L) for 48 h.

The phosphorylation of FAK (as depicted in Figure 5A) positively regulates the proliferation and migration of cancer cells. Therefore, the effects of compound **14f** on the expression levels of FAK and its phosphorylation sites in thyroid cancer cells TPC-1 were examined. As shown in Figure 5(B), the results indicated that treatment with compound **14f** significantly increased the phosphorylation levels of FAK at Tyr397, Tyr576/577, and Tyr925 in a dose-dependent manner, with particularly pronounced inhibition observed at Tyr397 and Tyr576/577 sites. Importantly, no significant changes were observed in the expression level of FAK upon treatment with compound **14f** (Figure 5B). These results suggested that Figure 5(B) could act on FAK to inhibit its activation through phosphorylation without affecting its expression or degradation processes.

### Effects of compound 14f on the proliferation and migration of thyroid cancer cells TPC-1

The phosphorylation and subsequent activation of FAK could facilitate the proliferation and migration of cancer cells. Suppression of FAK activation through specific compounds has been shown to effectively inhibit the proliferative and migratory abilities of cancer cells. Therefore, the effects of compound **14f** on the proliferative and migratory capacities of thyroid cancer cells TPC-1 were evaluated.

**Figure 6.**
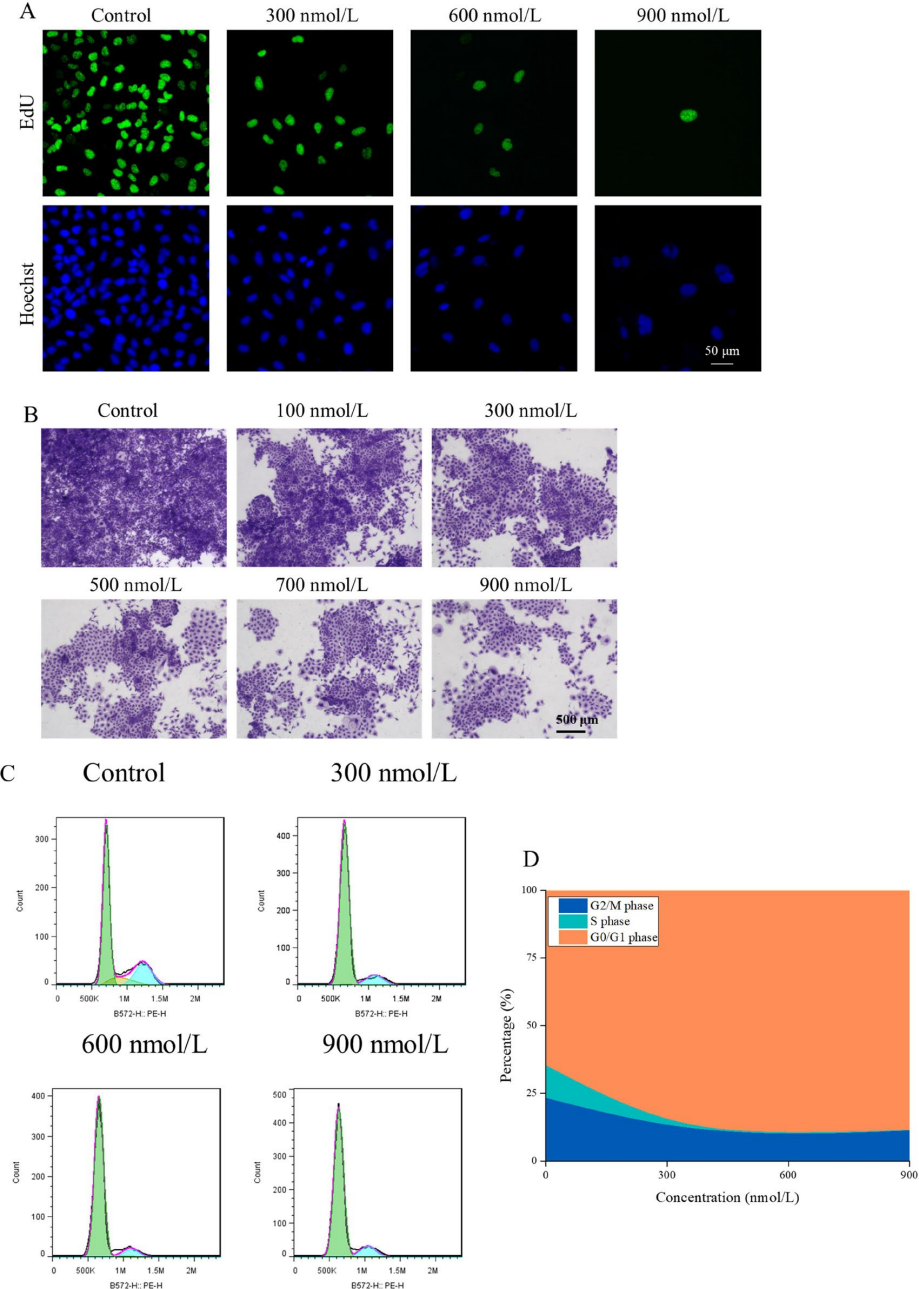
Effects of compound **14f** on the proliferation of thyroid cancer cells TPC-1. (**A**) In the EdU staining assay, TPC-1 cells treated with compound **14f** for 48 h were stained with EdU and Hoechst; (**B**) The colony formation of TPC-1 cells after treatment with various concentrations of compound **14f** for 5 days; (**C&D**) The changes in the cell cycle distribution of TPC-1 cells after a 48-h treatment.

Cells exhibiting positive EdU staining are indicative of robust proliferative activity. The experimental findings revealed there was a significant reduction in the number of thyroid cancer cells positive for EdU following treatment with compound **14f** (Figure 6A). The colony formation assay serves as an indirect measure to assess cellular proliferation. As shown in Figure 6(B), compound **14f** exhibited discernible inhibitory effects on the colony formation of TPC-1 cells starting from a concentration of 100 nmol/L over a span of 5-day treatment. In addition, we further explored the effects of compound 14f on the cell cycle distribution of TPC-1 cells, which could reflect the state of cellular proliferation. The results depicted in Figure 6(C&D) indicated that treatment with compound **14f** induced a pronounced G1 phase arrest in TPC-1 cells. These above results demonstrate that compound **14f** effectively inhibit the proliferation of thyroid cancer cells TPC-1.

**Figure 7.**
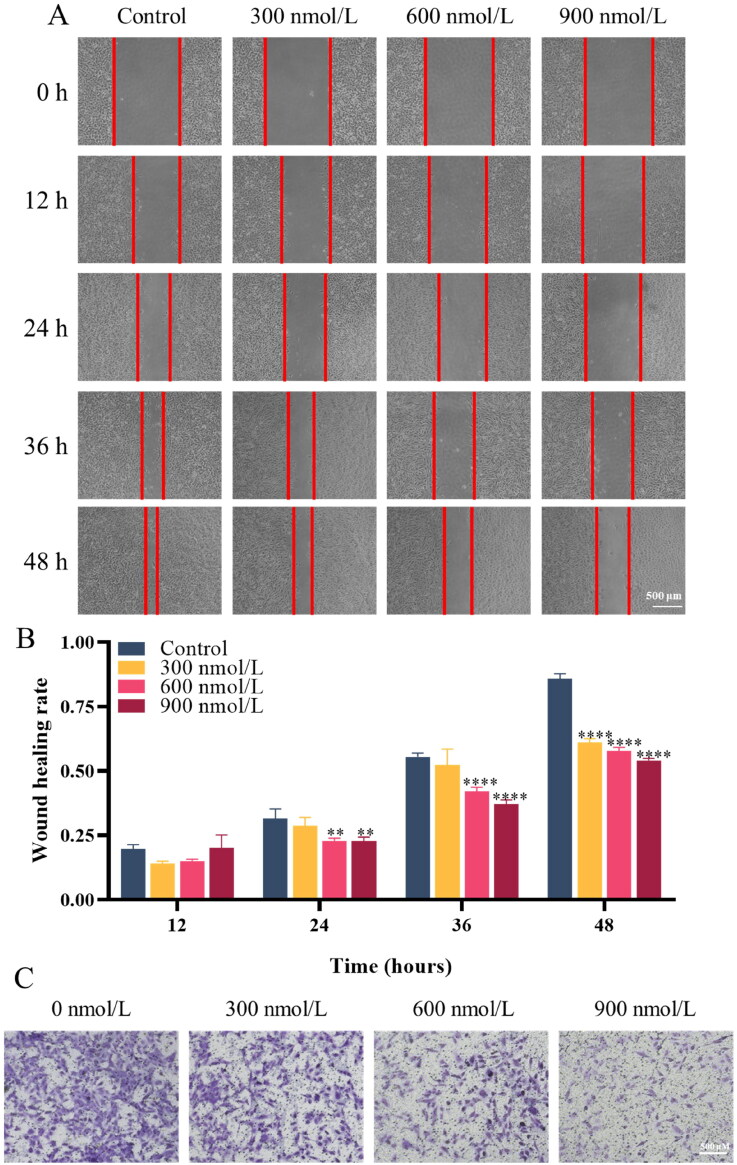
Effects of compound **14f** on the migration of thyroid cancer cells TPC-1. (**A&B**) In the wound healing assay, the healing of wounds in TPC-1 cells treated with compound **14f** for various durations is shown; (**C**) In the transwell migration assay, TPC-1 cells that migrated through the membrane after 48 h of treatment with compound **14f** are presented. **p < 0.01, ****p < 0.0001.

In the wound healing assay, the healing capacity of the cellular wound was impeded in the presence of compound **14f**, indicating that compound **14f** has a significant inhibitory effect on cell migration of thyroid cancer cells (Figure 7A). The transwell migration assay serves as an additional method to evaluate cellular migratory activity. Notably, a significant reduction in the number of cells that traversed through the membrane was observed upon exposure to compound **14f** (Figure 7A). These results demonstrated that compound **14f** effectively inhibited the migration of thyroid cancer cells TPC-1.

## Conclusions

In this study, we reported the discovery of novel 2,4-diarylaminopyrimidine-based hydrazones as anti-thyroid cancer agents. Based on the FAK inhibitor **TAE-226**, we designed and synthesised thirty novel compounds by introducing a hydrazone group, and evaluated their proliferation inhibitory activities against FAK-overexpressing TPC-1 cells. The majority of compounds exhibit moderate to excellent cytotoxic activity against FAK overexpressing TPC-1 cells, with IC_50_ values ranging from 0.113 to 1.460 μM. Among them, compound **14f** displayed exceptional anti-proliferative effect against TPC-1 cells (IC_50_ = 0.113 μM) and potent FAK inhibitory potency (IC_50_ = 35 nM). The results of molecular docking indicated compound **14f** could well bind to FAK. Furthermore, the calculated physicochemical properties showed that compound **14f** has favourable pharmacokinetic profiles. In addition, compound **14f** could inhibit the phosphorylation of FAK at Tyr397, Tyr576/577, and Tyr925 in a dose-dependent manner, and did not affect the expression level of FAK in TPC-1 cells. Treatment with compound **14f** was also effective in inhibiting the proliferation and migration of thyroid cancer cells TPC-1. Taken together, these results provided potential 2,4-diarylaminopyrimidine-based hydrazones for the treatment of thyroid cancers through the inhibition of FAK.

## Experimental section

### General chemical methods

All commercially available starting materials and solvents are used directly without further purification. The TLC was used to monitor the reactions. Column chromatography was performed using silica gel (200–300 mesh). Melting points were recorded by an X-5 micro melting apparatus. NMR spectra was acquired using a Bruker av400 spectrometer. HRMS was obtained on the Agilent 6545 LC/Q-TOF mass spectrometer.

### Procedure for the synthesis of 12

To a stirred solution of commercially available **11** (2.0 mmol) and 2,4,5-trichloropyrimidine (2.0 mmol) in isopropanol (20 ml) was added DIPEA (0.2 mmol). Then, the mixture was stirred at 85 °C for 12 h. Subsequently, the mixture was cooled to room temperature, and then the intermediate **12** was obtained after the filtration of the precipitation and the dryness of the filter cake without further purification.

### Procedure for the synthesis of 13

To a stirred solution of intermediate **12** (2.0 mmol) in ethanol (20 ml) was added hydrazine monohydrate (2.0 mmol). Then, the mixture was stirred at 25 °C for 4 h. Subsequently, the intermediate **13** was obtained after the filtration of the precipitation and the dryness of the filter cake without further purification.

### Procedure for the synthesis of 14a-14ad

To a stirred solution of intermediate **13** (1.0 mmol) in ethanol (10 ml) was added substituted aldehyde or ketone (1.0 mmol). Then, the mixture was stirred at 80 °C for 5 h. Subsequently, the filtrate was evaporated *in vacuo* to get crude products, which was then purified by the chromatography on silica gel to obtain compounds **14a-14ad**.

#### (E)-2-((2–(2-benzylidenehydrazineyl)-5-chloropyrimidin-4-yl)amino)-N-methylbenzamide (14a)

White solid; yield: 58%. M.p.199.1–202.1 °C.^1^H NMR (400 MHz, DMSO-*d_6_*) δ 11.95 (s, 1H), 11.26 (s, 1H), 9.37 (s, 1H), 8.80 (d, *J* = 4.5 Hz, 1H), 8.23 (s, 1H), 8.14 (s, 1H), 7.83 − 7.78 (m, 1H), 7.74 (d, *J* = 7.4 Hz, 2H), 7.60 (t, *J* = 7.4 Hz, 1H), 7.48 (t, *J* = 7.5 Hz, 2H), 7.39 (t, *J* = 7.3 Hz, 1H), 7.18 (t, *J* = 7.2 Hz, 1H), 2.84 (d, *J* = 4.5 Hz, 3H).^13^C NMR (100 MHz, DMSO-*d_6_*) δ 169.57, 158.13, 155.77, 155.31, 141.60, 140.49, 135.82, 132.09, 129.45, 129.29, 128.49, 126.67, 122.25, 121.38, 120.40, 105.92, 26.86. HRMS: calcd. C_19_H_18_ClN_6_O_1_ [M + H] ^+^, 381.1225, found: 381.1222.

#### (E)-2-((5-chloro-2–(2-(4-methylbenzylidene)hydrazineyl)pyrimidin-4-yl)amino)-N-methylbenzamide (14b)

White solid; yield: 62%. M.p.207.1–208.1 °C. ^1^H NMR (400 MHz, DMSO-*d_6_*) δ 11.94 (s, 1H), 11.19 (s, 1H), 9.37 (s, 1H), 8.80 (d, *J* = 4.4 Hz, 1H), 8.21 (s, 1H), 8.10 (s, 1H), 7.81 (d, *J* = 7.0 Hz, 1H), 7.60 (dd, *J* = 19.5, 7.7 Hz, 3H), 7.28 (d, *J* = 7.9 Hz, 2H), 7.17 (t, *J* = 7.3 Hz, 1H), 2.84 (d, *J* = 4.5 Hz, 3H), 2.35 (s, 3H). ^13^C NMR (100 MHz, DMSO-*d_6_*) δ 169.61, 158.18, 155.77, 141.67, 140.56, 139.14, 133.15, 132.15, 130.31, 129.93, 129.58, 128.54, 126.68, 121.79, 121.41, 120.38, 105.75, 26.54, 21.49. HRMS: calcd. C_20_H_20_ClN_6_O_1_ [M + H] ^+^, 395.1382, found: 395.1379.

#### (E)-2-((5-chloro-2–(2-(4-methoxybenzylidene)hydrazineyl)pyrimidin-4-yl)amino)-N-methylbenzamide (14c)

White solid; yield: 67%. M.p.187.7–188.9 °C. ^1^H NMR (400 MHz, DMSO-*d_6_*) δ 11.91 (s, 1H), 11.10 (s, 1H), 9.36 (s, 1H), 8.78 (d, *J* = 4.4 Hz, 1H), 8.20 (s, 1H), 8.09 (s, 1H), 7.80 (d, *J* = 7.7 Hz, 1H), 7.67 (d, *J* = 8.6 Hz, 2H), 7.60 (t, *J* = 7.8 Hz, 1H), 7.17 (t, *J* = 7.5 Hz, 1H), 7.05 (d, *J* = 8.6 Hz, 2H), 3.82 (s, 3H), 2.83 (d, *J* = 4.4 Hz, 3H). ^13^C NMR (100 MHz, DMSO-*d_6_*) δ 169.58, 160.55, 158.18, 155.73, 155.28, 141.68, 140.51, 132.17, 128.46, 128.40, 128.14, 122.20, 121.35, 120.35, 114.82, 105.51, 55.76, 26.85. HRMS: calcd. C_20_H_20_ClN_6_O_2_ [M + H] ^+^, 411.1331, found: 411.1325.

#### (E)-2-((5-chloro-2–(2-(4-hydroxybenzylidene)hydrazineyl)pyrimidin-4-yl)amino)-N-methylbenzamide (14d)

White solid; yield: 49%. M.p.187.8–189.2 °C. ^1^H NMR (400 MHz, DMSO-*d_6_*) δ 11.90 (s, 1H), 11.03 (s, 1H), 9.79 (s, 1H), 9.35 (s, 1H), 8.79 (d, *J* = 4.4 Hz, 1H), 8.19 (s, 1H), 8.05 (s, 1H), 7.80 (d, *J* = 7.4 Hz, 1H), 7.57 (t, *J* = 8.1 Hz, 3H), 7.17 (t, *J* = 7.4 Hz, 1H), 6.86 (d, *J* = 8.4 Hz, 2H), 2.83 (d, *J* = 4.4 Hz, 3H). ^13^C NMR (100 MHz, DMSO-*d_6_*) δ 169.58, 159.00, 158.21, 155.71, 155.29, 142.16, 140.54, 132.09, 128.46, 128.31, 126.85, 122.16, 121.37, 120.35, 116.17, 105.34, 26.85. HRMS: calcd. C_19_H_18_ClN_6_O_2_ [M + H] ^+^, 397.1174, found: 397.1174.

#### (E)-2-((5-chloro-2–(2-(4-(trifluoromethyl)benzylidene)hydrazineyl)pyrimidin-4-yl)amino)-N-methylbenzamide (14e)

White solid; yield: 56%. M.p.206.6–208.8 °C. ^1^H NMR (400 MHz, DMSO-*d_6_*) δ 11.99 (s, 1H), 11.49 (s, 1H), 9.33 (s, 1H), 8.81 (d, *J* = 4.4 Hz, 1H), 8.26 (s, 1H), 8.19 (s, 1H), 7.93 (d, *J* = 8.1 Hz, 2H), 7.83 (dd, *J* = 11.9, 8.3 Hz, 3H), 7.67 (t, *J* = 7.7 Hz, 1H), 7.18 (t, *J* = 7.3 Hz, 1H), 2.84 (d, *J* = 4.4 Hz, 3H). ^13^C NMR (100 MHz, DMSO-*d_6_*) δ169.59, 157.99, 155.84, 140.42, 139.85, 132.43, 132.37, 129.27, 128.96, 128.58, 127.08, 126.29, 126.16, 123.45, 121.35, 120.41, 106.52, 29.30. HRMS: calcd. C_20_H_17_ClF_3_N_6_O [M + H] ^+^, 449.1099, found: 449.1094.

#### (E)-2-((5-chloro-2–(2-(4-fluorobenzylidene)hydrazineyl)pyrimidin-4-yl)amino)-N-methylbenzamide (14f)

White solid; yield: 55%. M.p.176.1–177.8 °C. ^1^H NMR (400 MHz, DMSO-*d_6_*) δ 11.94 (s, 1H), 11.25 (s, 1H), 9.34 (s, 1H), 8.79 (d, *J* = 4.4 Hz, 1H), 8.22 (s, 1H), 8.13 (s, 1H), 7.83 − 7.74 (m, 3H), 7.61 (t, *J* = 7.7 Hz, 1H), 7.32 (t, *J* = 8.8 Hz, 2H), 7.17 (t, *J* = 7.5 Hz, 1H), 2.84 (d, *J* = 4.4 Hz, 3H). ^13^C NMR (100 MHz, DMSO-*d_6_*) δ 169.56, 164.19, 161.74, 158.11, 155.76, 155.28, 140.49, 140.44, 132.42, 132.39, 132.19, 128.64, 128.56, 128.47, 122.27, 121.34, 120.40, 116.43, 116.21, 105.94, 26.84. HRMS: calcd. C_19_H_17_ClFN_6_O [M + H] ^+^, 399.1131, found: 399.1129.

#### (E)-2-((5-chloro-2–(2-(4-chlorobenzylidene)hydrazineyl)pyrimidin-4-yl)amino)-N-methylbenzamide (14 g)

White solid; yield: 58%. M.p.194.1 − 195.8 °C. ^1^H NMR (400 MHz, DMSO-*d_6_*) δ 11.95 (s, 1H), 11.33 (s, 1H), 9.32 (s, 1H), 8.79 (d, *J* = 3.8 Hz, 1H), 8.23 (s, 1H), 8.12 (s, 1H), 7.77 (dd, *J* = 24.4, 7.9 Hz, 3H), 7.62 (t, *J* = 7.7 Hz, 1H), 7.54 (d, *J* = 8.1 Hz, 2H), 7.17 (t, *J* = 7.4 Hz, 1H), 2.84 (d, *J* = 4.0 Hz, 3H). ^13^C NMR (100 MHz, DMSO-*d_6_*) δ 169.59, 158.53, 158.08, 155.93, 155.80, 140.47, 140.24, 134.82, 133.79, 129.63, 129.34, 128.30, 121.36, 120.39, 112.65, 106.14, 26.75. HRMS: calcd. C_19_H_17_Cl_2_N_6_O [M + H] ^+^, 415.10835, found: 415.10833.

#### (E)-2-((2–(2-(4-bromobenzylidene)hydrazineyl)-5-chloropyrimidin-4-yl)amino)-N-methylbenzamide (14h)

White solid; yield: 58%. M.p.192.1–193.2 °C. ^1^H NMR (400 MHz, DMSO-*d_6_*) δ 11.95 (s, 1H), 11.33 (s, 1H), 9.32 (s, 1H), 8.79 (d, *J* = 4.2 Hz, 1H), 8.23 (s, 1H), 8.11 (s, 1H), 7.81 (d, *J* = 7.7 Hz, 1H), 7.70 − 7.60 (m, 5H), 7.17 (t, *J* = 7.5 Hz, 1H), 2.84 (d, *J* = 4.3 Hz, 3H). ^13^C NMR (100 MHz, DMSO-*d_6_*) δ 169.57, 160.08, 158.01, 155.77, 155.29, 140.39, 140.30, 135.11, 132.27, 128.46, 122.60, 122.45, 122.34, 121.34, 120.40, 106.15, 26.85. HRMS: calcd. C_19_H_17_ClBrN_6_O [M + H] ^+^, 459.0330, found: 459.0325.

#### (E)-2-((5-chloro-2–(2-(4-nitrobenzylidene)hydrazineyl)pyrimidin-4-yl)amino)-N-methylbenzamide (14i)

White solid; yield: 58%. M.p.201.7–202.8 °C. ^1^H NMR (400 MHz, DMSO-*d_6_*) δ 12.00 (s, 1H), 11.63 (s, 1H), 9.29 (s, 1H), 8.80 (d, *J* = 4.5 Hz, 1H), 8.33 (d, *J* = 8.7 Hz, 2H), 8.27 (s, 1H), 8.21 (s, 1H), 7.95 (d, *J* = 8.8 Hz, 2H), 7.81 (d, *J* = 7.8 Hz, 1H), 7.68 (t, *J* = 7.8 Hz, 1H), 7.19 (t, *J* = 7.4 Hz, 1H), 2.84 (s, 3H). ^13^C NMR (100 MHz, DMSO-*d_6_*) δ 169.54, 157.81, 155.83, 155.32, 147.48, 142.32, 140.31, 139.00, 132.37, 128.50, 127.26, 126.45, 124.69, 122.45, 121.33, 106.86, 26.85. HRMS: calcd. C_19_H_17_ClN_7_O_3_ [M + H] ^+^, 426.1076, found: 426.1072.

#### (E)-2-((5-chloro-2–(2-(4-cyanobenzylidene)hydrazineyl)pyrimidin-4-yl)amino)-N-methylbenzamide (14j)

White solid; yield: 57%. M.p.200.7–202.1 °C. ^1^H NMR (400 MHz, DMSO-*d_6_*) δ 11.99 (s, 1H), 11.54 (s, 1H), 9.31 (s, 1H), 8.80 (d, *J* = 4.3 Hz, 1H), 8.25 (s, 1H), 8.16 (s, 1H), 7.91 (q, *J* = 8.3 Hz, 4H), 7.81 (d, *J* = 7.7 Hz, 1H), 7.64 (t, *J* = 7.8 Hz, 1H), 7.18 (t, *J* = 7.5 Hz, 1H), 2.84 (d, *J* = 4.3 Hz, 3H). ^13^C NMR (100 MHz, DMSO-*d_6_*) δ 169.55, 157.87, 155.81, 155.28, 140.39, 140.36, 139.38, 133.21, 132.25, 128.48, 127.06, 122.38, 121.35, 120.44, 119.37, 111.08, 106.68, 26.85. HRMS: calcd. C_20_H_17_ClN_7_O [M + H] ^+^, 406.1178, found: 406.1177.

#### (E)-2-((5-chloro-2–(2-(3-methoxybenzylidene)hydrazineyl)pyrimidin-4-yl)amino)-N-methylbenzamide (14k)

White solid; yield: 53%. M.p.199.3–200.4 °C. ^1^H NMR (400 MHz, DMSO-*d_6_*) ^1^H NMR (400 MHz, DMSO) δ 11.93 (s, 1H), 11.27 (s, 1H), 9.32 (s, 1H), 8.80 (d, *J* = 4.1 Hz, 1H), 8.22 (s, 1H), 8.11 (s, 1H), 7.80 (d, *J* = 7.6 Hz, 1H), 7.58 (t, *J* = 7.6 Hz, 1H), 7.38 (t, *J* = 7.9 Hz, 1H), 7.29 (d, *J* = 6.3 Hz, 2H), 7.17 (t, *J* = 7.3 Hz, 1H), 6.96 (d, *J* = 7.3 Hz, 1H), 3.82 (s, 3H), 2.84 (d, *J* = 4.3 Hz, 3H). ^13^C NMR (100 MHz, DMSO-*d_6_*) δ 169.56, 160.08, 158.10, 155.78, 155.28, 141.53, 140.44, 137.23, 132.01, 130.35, 128.48, 122.23, 121.33, 120.48, 119.53, 115.25, 111.47, 105.97, 55.67, 26.84. HRMS: calcd. C_20_H_20_ClN_6_O_2_ [M + H] ^+^, 411.1331, found: 411.1330.

#### (E)-2-((5-chloro-2–(2-(3-fluorobenzylidene)hydrazineyl)pyrimidin-4-yl)amino)-N-methylbenzamide (14 l)

White solid; yield: 48%. M.p.203.3–205.1 °C. ^1^H NMR (400 MHz, DMSO-*d_6_*) δ 11.98 (s, 1H), 11.41 (s, 1H), 9.37 (s, 1H), 8.81 (s, 1H), 8.19 (d, *J* = 44.4 Hz, 2H), 7.81 (d, *J* = 7.2 Hz, 1H), 7.55 (d, *J* = 19.0 Hz, 4H), 7.20 (d, *J* = 7.2 Hz, 2H), 2.85 (s, 3H). ^13^C NMR (100 MHz, DMSO-*d_6_*) δ 169.53, 164.23, 161.81, 158.02, 155.78, 155.31, 140.46, 139.93, 138.56, 138.48, 131.87, 131.38, 131.30, 128.51, 123.23, 122.29, 121.33, 120.46, 116.20, 115.99, 112.29, 112.08, 106.23, 26.85. HRMS: calcd. C_19_H_17_ClFN_6_O [M + H] ^+^, 399.1131, found: 399.1132.

#### (E)-2-((5-chloro-2–(2-(3-(trifluoromethyl)benzylidene)hydrazineyl)pyrimidin-4-yl)amino)-N-methylbenzamide (14 m)

White solid; yield: 60%. M.p.207.1–209.8 °C. ^1^H NMR (400 MHz, DMSO-*d_6_*) δ 11.97 (s, 1H), 11.46 (s, 1H), 9.30 (s, 1H), 8.81 (d, *J* = 4.4 Hz, 1H), 8.24 (d, *J* = 14.1 Hz, 2H), 8.10 (s, 1H), 7.96 (d, *J* = 6.8 Hz, 1H), 7.81 (d, *J* = 7.1 Hz, 1H), 7.77 − 7.66 (m, 2H), 7.55 (t, *J* = 7.5 Hz, 1H), 7.18 (t, *J* = 7.4 Hz, 1H), 2.84 (d, *J* = 4.4 Hz, 3H). ^13^C NMR (100 MHz, DMSO-*d_6_*) δ 169.52, 157.98, 155.82, 155.30, 140.38, 139.83, 136.96, 131.78, 130.97, 130.43, 130.25, 129.94, 128.52, 126.01, 125.52, 123.31, 122.23, 122.13, 121.28, 120.51, 106.35, 26.84. HRMS: calcd. C_20_H_17_ClF_3_N_6_O [M + H] ^+^, 449.1099, found: 449.1096.

#### (E)-2-((5-chloro-2–(2-(2,4-dimethoxybenzylidene)hydrazineyl)pyrimidin-4-yl)amino)-N-methylbenzamide (14n)

White solid; yield: 56%. M.p. 213.1–214.7 °C. ^1^H NMR (400 MHz, DMSO-*d_6_*) δ 11.90 (s, 1H), 11.08 (s, 1H), 9.36 (s, 1H), 8.78 (d, *J* = 4.4 Hz, 1H), 8.38 (s, 1H), 8.17 (s, 1H), 7.89 (d, *J* = 8.6 Hz, 1H), 7.79 (d, *J* = 6.9 Hz, 1H), 7.57 (t, *J* = 7.5 Hz, 1H), 7.16 (t, *J* = 7.5 Hz, 1H), 6.70 (d, *J* = 8.5 Hz, 1H), 6.63 (d, *J* = 2.2 Hz, 1H), 3.84 (d, *J* = 7.5 Hz, 6H), 2.83 (d, *J* = 4.5 Hz, 3H). ^13^C NMR (100 MHz, DMSO-*d_6_*) δ 169.58, 162.06, 158.85, 158.14, 155.66, 155.26, 138.51, 137.41, 132.13, 128.45, 122.15, 121.35, 121.26, 120.28, 116.66, 108.79, 106.83, 98.82, 56.19, 55.92, 26.85. HRMS: calcd. C_21_H_22_ClN_6_O_3_ [M + H] ^+^, 441.1436, found: 441.1435.

#### (E)-2-((5-chloro-2–(2-(3,4-dimethoxybenzylidene)hydrazineyl)pyrimidin-4-yl)amino)-N-methylbenzamide (14o)

White solid; yield: 56%. M.p. 214.1–215.2 °C. ^1^H NMR (400 MHz, DMSO-*d_6_*), 11.03 (s, 1H), 9.34 (s, 1H), 8.79 (d, *J* = 4.4 Hz, 1H), 8.35 (s, 1H), 8.20 (s, 1H), 7.79 (d, *J* = 8.5 Hz, 2H), 7.56 (t, *J* = 7.7 Hz, 1H), 7.16 (t, *J* = 7.5 Hz, 1H), 6.87 (dd, *J* = 31.1, 5.2 Hz, 2H), 3.80 (s, 3H), 2.84 (d, *J* = 4.4 Hz, 3H), 2.48 (s, 3H). ^13^C NMR (100 MHz, DMSO-*d_6_*) δ 169.54, 158.13, 155.75, 155.29, 150.43, 149.50, 142.19, 140.39, 131.99, 128.50, 122.17, 121.27, 120.94, 120.84, 120.54, 112.19, 108.85, 105.55, 56.06, 55.97, 26.84. HRMS: calcd. C_21_H_22_ClN_6_O_3_ [M + H] ^+^, 441.1436, found: 441.1430.

#### (E)-2-((5-chloro-2–(2-(2-fluoro-4-methoxybenzylidene)hydrazineyl)pyrimidin-4-yl)amino)-N-methylbenzamide (14p)

White solid; yield: 57%. M.p. 218.1–219.4 °C. ^1^H NMR (400 MHz, DMSO-*d_6_*), δ 11.94 (s, 1H), 11.25 (s, 1H), 9.33 (s, 1H), 8.79 (d, *J* = 4.4 Hz, 1H), 8.24 (d, *J* = 22.2 Hz, 2H), 7.91 (t, *J* = 8.7 Hz, 1H), 7.80 (d, *J* = 7.1 Hz, 1H), 7.59 (t, *J* = 7.6 Hz, 1H), 7.16 (t, *J* = 7.5 Hz, 1H), 7.02 − 6.86 (m, 2H), 3.84 (s, 3H), 2.83 (d, *J* = 4.4 Hz, 3H). ^13^C NMR (100 MHz, DMSO-*d_6_*) δ169.56, 158.18, 158.04, 155.76, 155.36, 155.30, 154.25, 140.60, 140.46, 134.58, 132.18, 128.47, 126.91, 126.84, 125.46, 122.25, 121.32, 120.36, 112.19, 105.84, 56.35, 26.85. HRMS: calcd. C_20_H_19_ClFN_6_O_2_ [M + H] ^+^, 429.1237, found: 429.1234.

#### (E)-2-((5-chloro-2–(2-(3-fluoro-4-methoxybenzylidene)hydrazineyl)pyrimidin-4-yl)amino)-N-methylbenzamide (14q)

White solid; yield: 57%. M.p. 234.1–235.7 °C. ^1^H NMR (400 MHz, DMSO-*d_6_*), ^1^H NMR (400 MHz, DMSO) δ 11.95 (s, 1H), 11.24 (s, 1H), 9.37 (s, 1H), 8.80 (d, *J* = 4.4 Hz, 1H), 8.21 (s, 1H), 8.06 (s, 1H), 7.81 (d, *J* = 7.4 Hz, 1H), 7.59 (dd, *J* = 16.7, 9.5 Hz, 2H), 7.45 (d, *J* = 8.4 Hz, 1H), 7.22 (dt, *J* = 14.9, 8.0 Hz, 2H), 3.90 (s, 3H), 2.84 (d, *J* = 4.4 Hz, 3H). ^13^C NMR (100 MHz, DMSO-*d_6_*) δ 169.55, 158.09, 155.74, 155.28, 153.45, 151.03, 148.39, 148.28, 140.52, 140.22, 131.89, 129.20, 129.14, 128.51, 123.99, 122.23, 121.32, 120.39, 114.40, 112.85, 112.66, 105.79, 56.61, 26.86. HRMS: calcd. C_20_H_19_ClFN_6_O_2_ [M + H] ^+^, 429.1237, found: 429.1232.

#### (E)-2-((5-chloro-2–(2-(2,4-difluorobenzylidene)hydrazineyl)pyrimidin-4-yl)amino)-N-methylbenzamide (14r)

White solid; yield: 57%. M.p. 243.2–245.1 °C. ^1^H NMR (400 MHz, DMSO-*d_6_*), δ 11.96 (s, 1H), 11.39 (s, 1H), 9.30 (s, 1H), 8.79 (d, *J* = 4.5 Hz, 1H), 8.28 (s, 1H), 8.23 (s, 1H), 8.02 (dd, *J* = 15.4, 8.4 Hz, 1H), 7.80 (d, *J* = 7.2 Hz, 1H), 7.60 (t, *J* = 7.6 Hz, 1H), 7.36 − 7.25 (m, 2H), 7.16 (t, *J* = 7.4 Hz, 1H), 2.83 (d, *J* = 4.4 Hz, 3H). ^13^C NMR (100 MHz, DMSO-*d_6_*) δ 169.55, 157.94, 155.78, 155.31, 140.38, 133.49, 133.46, 133.44, 132.23, 128.48, 127.43, 127.39, 122.33, 121.32, 120.39, 113.01, 112.97, 106.29, 104.92, 104.65, 26.86. HRMS: calcd. C_19_H_16_ClF_2_N_6_O [M + H] ^+^, 417.1037, found: 417.1033.

#### (E)-2-((5-chloro-2–(2-(pyridin-2-ylmethylene)hydrazineyl)pyrimidin-4-yl)amino)-N-methylbenzamide (14s)

White solid; yield: 56%. M.p. 217.1–218.2 °C. ^1^H NMR (400 MHz, DMSO-*d_6_*), δ 11.97 (s, 1H), 11.50 (s, 1H), 9.30 (s, 1H), 8.80 (d, *J* = 4.4 Hz, 1H), 8.58 (d, *J* = 4.6 Hz, 1H), 8.26 (s, 1H), 8.18 (s, 1H), 8.02 (d, *J* = 7.9 Hz, 1H), 7.93 (t, *J* = 7.3 Hz, 1H), 7.81 (d, *J* = 7.1 Hz, 1H), 7.62 (t, *J* = 7.5 Hz, 1H), 7.36 (dd, *J* = 6.3, 5.2 Hz, 1H), 7.18 (t, *J* = 7.4 Hz, 1H), 2.84 (d, *J* = 4.5 Hz, 3H). ^13^C NMR (100 MHz, DMSO-*d_6_*) δ 169.54, 157.89, 155.81, 155.33, 154.71, 149.82, 142.10, 140.35, 137.15, 132.18, 128.50, 123.86, 122.35, 121.34, 120.49, 119.26, 106.57, 26.85. HRMS: calcd. C_18_H_17_ClN_7_O [M + H] ^+^, 382.1178, found: 382.1173.

#### (E)-2-((5-chloro-2–(2-(pyridin-3-ylmethylene)hydrazineyl)pyrimidin-4-yl)amino)-N-methylbenzamide (14t)

White solid; yield: 52%. M.p. 217.1–218.1 °C. ^1^H NMR (400 MHz, DMSO-*d_6_*), δ 12.01 (s, 1H), 11.59 (s, 1H), 9.31 (s, 1H), 8.81 (d, *J* = 4.2 Hz, 1H), 8.65 (d, *J* = 5.3 Hz, 2H), 8.27 (s, 1H), 8.10 (s, 1H), 7.82 (d, *J* = 7.7 Hz, 1H), 7.64 (dd, *J* = 11.5, 6.6 Hz, 3H), 7.19 (t, *J* = 7.5 Hz, 1H), 2.84 (d, *J* = 4.3 Hz, 3H). ^13^C NMR (100 MHz, DMSO-*d_6_*) δ 169.53, 157.99, 155.78, 155.30, 150.04, 148.28, 140.41, 138.57, 133.08, 132.03, 131.67, 128.49, 124.44, 122.29, 121.36, 120.45, 106.31. HRMS: calcd. C_18_H_17_ClN_7_O [M + H] ^+^, 382.1178, found: 382.1174.

#### (E)-2-((5-chloro-2–(2-(pyridin-4-ylmethylene)hydrazineyl)pyrimidin-4-yl)amino)-N-methylbenzamide (14 u)

White solid; yield: 57%. M.p. 215.1–216.8 °C. ^1^H NMR (400 MHz, DMSO-*d_6_*), δ 11.97 (s, 1H), 11.45 (s, 1H), 9.34 (s, 1H), 8.85 (d, *J* = 34.2 Hz, 2H), 8.57 (s, 1H), 8.16 (dd, *J* = 37.2, 19.4 Hz, 3H), 7.81 (d, *J* = 7.5 Hz, 1H), 7.57 (dd, *J* = 23.7, 16.2 Hz, 2H), 7.18 (t, *J* = 7.1 Hz, 1H), 2.84 (d, *J* = 3.1 Hz, 3H). ^13^C NMR (100 MHz, DMSO-*d_6_*) δ 169.54, 157.83, 155.83, 155.30, 150.63, 142.97, 140.35, 138.79, 132.17, 128.50, 122.40, 121.33, 120.66, 120.46, 106.81, 26.86. HRMS: calcd. C_18_H_17_ClN_7_O [M + H] ^+^, 382.1178, found: 382.1176.

#### (E)-2-((5-chloro-2–(2-(thiophen-2-ylmethylene)hydrazineyl)pyrimidin-4-yl)amino)-N-methylbenzamide (14v)

White solid; yield: 57%. M.p. 214.6–216.1 °C. ^1^H NMR (400 MHz, DMSO-*d_6_*), δ 11.98 (s, 1H), 11.25 (s, 1H), 9.39 (s, 1H), 8.80 (d, *J* = 4.5 Hz, 1H), 8.32 (s, 1H), 8.21 (s, 1H), 7.80 (dd, *J* = 7.9, 1.3 Hz, 1H), 7.62 (dd, *J* = 17.4, 6.3 Hz, 2H), 7.33 (d, *J* = 2.8 Hz, 1H), 7.19 − 7.08 (m, 2H), 2.83 (d, *J* = 4.5 Hz, 3H).^13^C NMR (100 MHz, DMSO-*d_6_*) δ 169.61, 157.97, 155.75, 155.26, 141.02, 140.54, 137.03, 132.34, 128.99, 128.43, 128.18, 127.85, 122.13, 121.40, 120.24, 105.78, 26.85. HRMS: calcd. C_17_H_16_ClN_6_OS [M + H] ^+^, 387.0789, found: 387.0784.

#### (E)-2-((5-chloro-2–(2-(quinolin-7-ylmethylene)hydrazineyl)pyrimidin-4-yl)amino)-N-methylbenzamide (14w)

White solid; yield: 46%. M.p. 241.7–242.1 °C. ^1^H NMR (400 MHz, DMSO-*d_6_*), δ 11.96 (s, 1H), 11.53 (s, 1H), 9.43 (s, 1H), 8.99 (dd, *J* = 4.1, 1.7 Hz, 1H), 8.81 (d, *J* = 4.5 Hz, 1H), 8.48 − 8.43 (m, 2H), 8.25 (s, 1H), 8.04 (d, *J* = 7.1 Hz, 1H), 7.83 − 7.77 (m, 2H), 7.66 − 7.60 (m, 2H), 7.19 (t, *J* = 7.2 Hz, 1H), 2.84 (d, *J* = 4.5 Hz, 3H). ^13^C NMR (100 MHz, DMSO-*d_6_*) δ 169.58, 158.13, 155.76, 155.30, 151.12, 150.59, 145.51, 140.47, 138.56, 137.05, 132.75, 132.16, 129.26, 128.63, 128.46, 127.08, 124.86, 122.27, 121.44, 120.40, 106.02, 26.85. HRMS: calcd. C_22_H_19_ClN_7_O [M + H] ^+^, 432.1334, found: 432.1338.

#### (E)-2-((2–(2-((1H-indol-3-yl)methylene)hydrazineyl)-5-chloropyrimidin-4-yl)amino)-N-methylbenzamide (14x)

White solid; yield: 56%. M.p. 203.3–204.8 °C. ^1^H NMR (400 MHz, DMSO-*d_6_*),δ 11.84 (s, 1H), 11.46 (s, 1H), 10.86 (s, 1H), 9.43 (s, 1H), 8.79 (d, *J* = 4.4 Hz, 1H), 8.44 (d, *J* = 7.8 Hz, 1H), 8.36 (s, 1H), 8.19 (s, 1H), 7.81 (d, *J* = 7.1 Hz, 1H), 7.72 (d, *J* = 2.6 Hz, 1H), 7.60 (t, *J* = 7.4 Hz, 1H), 7.45 (d, *J* = 8.1 Hz, 1H), 7.23 (dd, *J* = 17.4, 7.6 Hz, 2H), 7.14 (t, *J* = 7.3 Hz, 1H), 2.84 (d, *J* = 4.5 Hz, 3H). ^13^C NMR (100 MHz, DMSO-*d_6_*) δ 169.61, 158.37, 155.90, 155.32, 140.60, 140.25, 137.60, 132.13, 131.71, 129.64, 128.53, 124.78, 123.00, 122.46, 122.00, 121.28, 120.65, 120.46, 112.86, 112.25, 26.86. HRMS: calcd. C_21_H_19_ClN_7_O [M + H] ^+^, 420.1334, found: 4202.1332.

#### ((E)-2-((5-chloro-2–(2-((1-methyl-1H-indol-3-yl)methylene) hydrazineyl) pyrimidin-4-yl)amino)-N-methylbenzamide (14 y)

White solid; yield: 58%. M.p. 217.2–219.1 °C. ^1^H NMR (400 MHz, DMSO-*d_6_*), δ 11.84 (s, 1H), 10.86 (s, 1H), 9.43 (s, 1H), 8.80 (d, *J* = 4.5 Hz, 1H), 8.45 (d, *J* = 7.7 Hz, 1H), 8.33 (s, 1H), 8.19 (s, 1H), 7.81 (d, *J* = 7.0 Hz, 1H), 7.71 (s, 1H), 7.60 (t, *J* = 7.5 Hz, 1H), 7.51 (d, *J* = 8.2 Hz, 1H), 7.30 (t, *J* = 7.4 Hz, 1H), 7.24 − 7.16 (m, 2H), 3.82 (s, 3H), 2.84 (d, *J* = 4.5 Hz, 3H). ^13^C NMR (100 MHz, DMSO-*d_6_*) δ 169.60, 158.32, 155.89, 155.32, 140.57, 139.73, 138.09, 133.39, 132.10, 128.53, 125.14, 123.08, 122.59, 122.02, 121.27, 120.87, 120.47, 111.91, 110.58, 104.57, 33.19, 26.85. HRMS: calcd. C_22_H_21_ClN_7_O [M + H] ^+^, 434.1491, found: 434.1487.

#### (E)-2-((5-chloro-2–(2-((5-methoxy-1H-indol-3-yl)methylene) hydrazineyl) pyrimidin-4-yl)amino)-N-methylbenzamide (14z)

White solid; yield: 57%. M.p. 217.1–218.2 °C. ^1^H NMR (400 MHz, DMSO-*d_6_*), δ 11.86 (s, 1H), 11.33 (s, 1H), 10.79 (s, 1H), 9.37 (s, 1H), 8.77 (d, *J* = 4.5 Hz, 1H), 8.35 (s, 1H), 8.19 (s, 1H), 7.90 (s, 1H), 7.78 (d, *J* = 6.9 Hz, 1H), 7.68 (d, *J* = 2.6 Hz, 1H), 7.55 (t, *J* = 7.4 Hz, 1H), 7.36 (d, *J* = 8.8 Hz, 1H), 7.12 (t, *J* = 7.5 Hz, 1H), 6.91 (dd, *J* = 8.7, 2.2 Hz, 1H), 3.68 (s, 3H), 2.84 (d, *J* = 4.5 Hz, 3H). ^13^C NMR (100 MHz, DMSO-*d_6_*) δ 169.63, 158.36, 155.80, 155.31, 154.77, 140.58, 132.75, 132.59, 130.16, 130.05, 128.45, 125.48, 121.83, 120.99, 120.09, 112.67, 112.46, 111.78, 106.00, 104.64, 56.18, 26.84. HRMS: calcd. C_22_H_21_ClN_7_O_2_ [M + H] ^+^, 450.1440, found: 434.1487.

#### (E)-2-((2–(2-((1H-indol-5-yl)methylene)hydrazineyl)-5-chloropyrimidin-4-yl)amino)-N-methylbenzamide (14aa)

White solid; yield: 65%. M.p. 218.1–219.9 °C. ^1^H NMR (400 MHz, DMSO-*d_6_*), δ 11.91 (s, 1H), 11.28 (s, 1H), 11.05 (s, 1H), 9.44 (s, 1H), 8.79 (d, *J* = 4.2 Hz, 1H), 8.21 (d, *J* = 5.5 Hz, 2H), 7.81 (s, 2H), 7.63 (t, *J* = 8.3 Hz, 2H), 7.49 (d, *J* = 8.5 Hz, 1H), 7.40 (s, 1H), 7.19 (t, *J* = 7.4 Hz, 1H), 6.51 (s, 1H), 2.84 (d, *J* = 4.3 Hz, 3H). ^13^C NMR (100 MHz, DMSO-*d_6_*) δ 169.60, 158.30, 155.72, 155.32, 143.83, 140.61, 136.97, 132.09, 128.47, 128.19, 127.04, 126.70, 122.16, 121.46, 120.37, 120.24, 119.39, 112.43, 105.20, 102.24, 26.85. HRMS: calcd. C_21_H_19_ClN_7_O [M + H] ^+^, 420.1334, found: 4202.1334.

#### (E)-2-((2–(2-((1H-indol-6-yl)methylene)hydrazineyl)-5-chloropyrimidin-4-yl)amino)-N-methylbenzamide (14ab)

White solid; yield: 54%. M.p. 216.1–217.7 °C. ^1^H NMR (400 MHz, DMSO-*d_6_*),δ 11.94 (s, 1H), 11.29 (s, 1H), 11.09 (s, 1H), 9.44 (s, 1H), 8.80 (d, *J* = 4.3 Hz, 1H), 8.22 (d, *J* = 5.8 Hz, 2H), 7.82 (d, *J* = 7.7 Hz, 1H), 7.72 − 7.59 (m, 3H), 7.51 (d, *J* = 8.0 Hz, 1H), 7.45 (s, 1H), 7.19 (t, *J* = 7.4 Hz, 1H), 6.48 (s, 1H), 2.85 (d, *J* = 4.3 Hz, 3H). ^13^C NMR (100 MHz, DMSO-*d_6_*) δ 169.61, 158.23, 155.75, 155.31, 143.73, 140.58, 136.42, 132.30, 129.17, 128.93, 128.47, 127.43, 122.17, 121.40, 120.81, 120.32, 117.77, 110.70, 105.36, 102.01, 26.86. HRMS: calcd. C_21_H_19_ClN_7_O [M + H] ^+^, 420.1334, found: 4202.1332.

#### (E)-2-((5-chloro-2–(2-(1–(4-methoxyphenyl)ethylidene)hydrazineyl)pyrimidin-4-yl)amino)-N-methylbenzamide (14ac)

White solid; yield: 56%. M.p. 216.7–217.1 °C. ^1^H NMR (400 MHz, DMSO-*d_6_*), δ 11.95 (s, 1H), 10.08 (s, 1H), 9.48 (d, *J* = 8.2 Hz, 1H), 8.79 (d, *J* = 4.4 Hz, 1H), 8.23 (s, 1H), 7.88 (d, *J* = 8.8 Hz, 2H), 7.80 (d, *J* = 7.0 Hz, 1H), 7.57 (t, *J* = 7.4 Hz, 1H), 7.17 (t, *J* = 7.3 Hz, 1H), 7.03 (d, *J* = 8.9 Hz, 2H), 3.83 (s, 3H), 2.84 (d, *J* = 4.4 Hz, 3H), 2.30 (s, 3H). ^13^C NMR (100 MHz, DMSO-*d_6_*) δ 169.59, 160.19, 158.96, 155.76, 155.13, 146.66, 140.58, 132.10, 131.82, 128.42, 127.51, 122.20, 121.48, 120.24, 114.17, 105.66, 55.73, 26.84, 13.75. HRMS: calcd. C_21_H_22_ClN_6_O_2_ [M + H] ^+^, 425.1487, found: 425.1485.

#### (E)-2-((5-chloro-2–(2-(1–(4-fluorophenyl)ethylidene)hydrazineyl)pyrimidin-4-yl)amino)-N-methylbenzamide (14ad)

White solid; yield: 54%. M.p. 222.6–223.8 °C. ^1^H NMR (400 MHz, DMSO-*d_6_*), δ 11.96 (s, 1H), 10.20 (s, 1H), 9.43 (d, *J* = 8.4 Hz, 1H), 8.78 (d, *J* = 4.5 Hz, 1H), 8.25 (s, 1H), 7.97 (dd, *J* = 8.8, 5.6 Hz, 2H), 7.87 − 7.73 (m, 1H), 7.58 (dd, *J* = 11.5, 4.2 Hz, 1H), 7.31 (t, *J* = 8.8 Hz, 2H), 7.17 (t, *J* = 7.4 Hz, 1H), 2.84 (d, *J* = 4.5 Hz, 3H), 2.33 (s, 3H). ^13^C NMR (100 MHz, DMSO-*d_6_*) δ 169.60, 164.10, 161.66, 158.87, 155.80, 155.12, 145.59, 140.50, 135.81, 132.12, 128.42, 128.17, 128.09, 122.28, 121.49, 120.32, 115.74, 115.52, 106.04, 26.85, 13.82. HRMS: calcd. C_20_H_19_ClFN_6_O [M + H] ^+^, 413.1287, found: 413.1282.

### Cell lines and cell culture

Thyroid cancer cells TCP-1 were provided by Wuhan Servicebio Co., Ltd. TCP-1 cells are cultured in RPMI-1640 medium supplemented with 10% foetal bovine serum (FBS), 100 U/mL penicillin, and 0.1 mg/mL streptomycin. All cells are maintained in a humidified incubator at 37 °C with 5% CO_2_.

### MTT assay

The TCP-1 cell line is seeded into a 96-well plate and incubated under conditions of 37 °C and 5% CO_2_. After the cells adhere, different concentrations of compounds are added and the incubation continues for 48 h under the same conditions. Subsequently, MTT reagent is added to the medium and the incubation is continued for an additional 4 h at 37 °C. After the incubation, the medium is aspirated, and 150 μL of DMSO solution is added to dissolve the purple-blue crystals by shaking. The absorbance of the solution is measured at a wavelength of 490 nm. The IC_50_ value of the compounds is calculated using SPSS software version 20.0.

### Kinase inhibition assay

The 50 µl reaction mixture contains 40 mM Tris, pH 7.4, 10 mM MgCl_2_, 0.1 mg/ml BSA, 1 mM DTT, 10 µM ATP, Kinase and the enzyme substrate. The compound was diluted in 10% DMSO and 5 µl of the dilution was added to a 50 µl reaction so that the final concentration of DMSO is 1% in all of the reactions. The assay was performed using a Kinase-Glo Plus luminescence kinase assay kit. It measures kinase activity by quantitating the amount of ATP remaining in solution following a kinase reaction. The luminescent signal from the assay is correlated with the amount of ATP present and is inversely correlated with the amount of kinase activity. The IC_50_ values were calculated using nonlinear regression with normalised dose-response fit using Prism GraphPad software.

### Molecular docking

The X-ray crystal structure of 2JKK (PDB: 2JKK) was retrieved from the Protein Data Bank. The protonation state of compound **14f** was set at pH = 7.4, and the compound **14f** were expanded to 3D structures using Open Babel. MOE was applied to prepare and parametrise the receptor protein and ligands. The docking grid documents were generated by Auto Grid of sitemap, and MOE was used for docking simulation. The optimal pose was selected to analyse interaction. Finally, the protein-ligand interaction figure was generated by PyMOL.

### ADMET prediction and drug-likeness properties

Here, we used the Swiss ADME Online software (www.SwissADME.ch) to measure in the silico ADME profile and drug-likeness properties.

### Western blot

Different concentrations of compound **14f** are applied to TCP-1 cells for 48 h, after which the cells are collected and lysed in RIPA lysis buffer (Solarbio, R0010). Protein concentration is determined using the BCA Protein Concentration Assay Kit (Solarbio, PC0020). Proteins were denatured using 4X SDS-PAGE loading buffer (Solarbio, P1016) at 100 °C for 10 min. The denatured protein samples are separated by SDS-PAGE electrophoresis. Proteins are transferred from the gel to nitrocellulose (NC) membrane (Pall Corporation, P/N 66485) and blocked with 5% non-fat milk for 1 h at room temperature. After washing with TBST, the primary antibody is added and incubated overnight at 4 °C. The membrane is washed with TBST and then incubated with the secondary antibody at room temperature for 1–2 h. The membrane is washed again with TBST, and the protein bands are visualised using an ECL (Solarbio, PE0010) ultra-sensitive luminescent reagent, followed by imaging with a chemiluminescence imaging analyser (Cytiva, AMERSHAM ImageQuant 800, JPN). The ladder used for Western Blot experiments was purchased from Thermo Scientific (Thermo Scientific, catalog number:26616). Information on the antibodies used in this study is provided below: Anti-FAK-antibody (Cell Signalling Technology, #3285S), Phospho-FAK (Tyr925) Antibody (Cell Signalling Technology, #3284S), Phospho-FAK (Tyr576/577) Antibody (Cell Signalling Technology, #3281S), Phospho-FAK (Tyr397) Antibody (Bioss, bs-3159R), β-Actin (Abways, AB0035), Goat Anti-Rabbit IgG (H + L) HRP (Cell Signalling Technology, #35401).

### Colony formatting assay

TCP-1 cells are plated at a density of 10,000 to 20,000 cells per well in a 6-well plate. After cell adhesion, the cells are treated with different concentrations of compound **14f** and cultured for 5 days in an incubator. At the end of the culture period, the medium is aspirated, and the cells are washed three times with PBS. The cells are then fixed with 4% paraformaldehyde for 20 min at room temperature. After fixation, the fixative is removed, and the cells are stained with 0.1% crystal violet at room temperature for 20 min. Following staining, the cells are rinsed with PBS until the purple colour is completely washed away, and then air-dried for image acquisition.

### EdU staining

TCP-1 cells are treated with compound **14f**, followed by the addition of an equal volume of 1X EdU working solution to the drug-containing medium, and incubated under conditions of 37 °C and 5% CO_2_ for 4 h. After incubation, the cells are fixed with 4% paraformaldehyde for 15 min. The fixative is then removed, and the cells are permeabilized with PBS containing 0.3% Triton X-100 at room temperature. After washing, the Click reaction working solution is added and incubated at 37 °C for 30 min. The cells are then washed and stained with a prepared Hoechst staining solution for 10 min to stain the cell nuclei. After staining, the staining solution is removed, the cells are washed, and images are captured and saved using a fluorescence microscope.

### Wound healing assay

TCP-1 cells are seeded into a 6-well cell culture plate and cultured in an incubator at 37 °C with 5% CO_2_. Once the cells uniformly cover the bottom of the well, a vertical line is drawn in the centre of each well to mark the initial scratch. Fresh medium is replaced and different concentrations of **14f** are added. Photographs are taken to document the healing at 0 h, 24 h, 36 h and 48 h post-scratch. Images are processed using ImageJ software. Data analysis is performed using GraphPad Prism software version 8.0.

### Transwell assay

A 24-well plate is filled with 500 μL of RPMI-1640 medium containing 20% serum per well, and Transwell chambers are placed in the wells. TCP-1 cells are resuspended in serum-free medium and mixed with compound **14f**, then added to the upper chamber of the Transwell chambers. The 24-well plates are incubated at 37 °C with 5% CO_2_ for 48 h. After incubation, the plates are removed, and the chambers are immersed in 4% paraformaldehyde and fixed at room temperature for 10 min. Following fixation, 0.1% crystal violet staining solution is added to the 24-well plates, and the chambers are stained at room temperature for 30 min. After staining, the plates are washed, the upper layer of cells is gently wiped off with a cotton swab, and the chambers are air-dried. Images are captured using a microscope.

## Supplementary Material

Supplemental Material

Original Image for Figure 7A 36h Control_2.tif

Original Image for Figure 7A 0h Control_3.tif

Original Image for Figure 7A 12h 300 nM_3.tif

Original Image for Figure 7A 0h 600 nM_1.tif

Original Image for Figure 7A 0h 600 nM_2.tif

Original Image for Figure 7A 24h 600 nM_3.tif

Supporting information.docx

Original Image for Figure 7A 12h 600 nM_3.tif

Original Image for Figure 7A 0h 900 nM_2.tif

Original Image for Figure 6B 300 nM.TIF

Original Image for Figure 6A 300 nM_Hoechst.tif

Original Image for Figure 7A 48h 600 nM_2.tif

Original Image for Figure 7A 36h 900 nM_2.tif

Original Image for Figure 6A 900 nM_Hoechst.tif

Original Image for Figure 7A 12h 600 nM_2.tif

Original Image for Figure 7A 36h 900 nM_3.tif

Original Image for Figure 7C 600 nM.TIF

Original Image for Figure 6A 300 nM_EdU.tif

Original Image for Figure 6B Control.TIF

Original Image for Figure 6A control_EdU.tif

Original Image for Figure 7A 12h 900 nM_1.tif

Original Image for Figure 7A 36h 300 nM_1.tif

Original Image for Figure 7A 0h 600 nM_3.tif

Original Image for Figure 7A 24h 600 nM_1.tif

Original Image for Figure 7A 24h Control_1.tif

Original Image for Figure 7A 24h 300 nM_3.tif

Original Image for Fig 5B_p_FAK Y397.tif

Original Image for Figure 6B 500 nM.TIF

Original Image for Figure 7A 48h 600 nM_1.tif

Original Image for Fig 5B_p_FAK Y925.tif

Original Image for Figure 7A 12h 300 nM_1.tif

Original Image for Figure 7A 24h 900 nM_1.tif

Original Image for Figure 7A 24h 600 nM_2.tif

Original Image for Fig 5B_β_actin.tif

Original Image for Figure 7A 12h 600 nM_1.tif

Original Image for Figure 7A 36h 600 nM_1.tif

Original Image for Figure 7A 36h 600 nM_2.tif

Original Image for Figure 7A 36h 600 nM_3.tif

Original Image for Figure 6B 700 nM.TIF

Original Image for Figure 7C Control.TIF

Original Image for Figure 7A 36h Control_3.tif

Original Image for Figure 7A 12h Control_3.tif

Original Image for Figure 7A 48h Control_3.tif

Original Image for Figure 7A 0h Control_1.tif

Original Image for Figure 7A 36h Control_1.tif

Original Image for Figure 6A 600 nM_Hoechst.tif

Original Image for Figure 6B 900 nM.TIF

Original Image for Figure 7A 48h 300 nM_1.tif

Original Image for Figure 7A 24h Control_2.tif

Original Image for Figure 7A 48h 900 nM_2.tif

Original Image for Figure 7A 24h 300 nM_2.tif

Original Image for Figure 7A 48h 300 nM_3.tif

Original Image for Figure 7A 48h 600 nM_3.tif

Original Image for Figure 7A 24h 300 nM_1.tif

Original Image for Figure 7C 300 nM.TIF

Original Image for Figure 7A 48h 300 nM_2.tif

Original Image for Figure 7A 48h 900 nM_3.tif

Original Image for Figure 7A 48h 900 nM_1.tif

Original Image for Figure 6A control_Hoechst.tif

Original Image for Figure 7A 0h 900 nM_1.tif

Original Image for Figure 7A 36h 300 nM_2.tif

Original Image for Figure 7A 0h 300 nM_3.tif

Original Image for Figure 7A 12h Control_2.tif

Original Image for Figure 7A 0h 300 nM_2.tif

Original Image for Figure 7A 36h 900 nM_1.tif

Original Image for Figure 7A 24h Control_3.tif

Original Image for Figure 7A 0h 900 nM_3.tif

Original Image for Figure 7A 48h Control_2.tif

Original Image for Figure 7A 48h Control_1.tif

Original Image for Figure 7A 12h Control_1.tif

Original Image for Fig 5B_FAK.tif

Original Image for Figure 7A 12h 300 nM_2.tif

Original Image for Figure 7A 24h 900 nM_3.tif

Original Image for Figure 7A 0h 300 nM_1.tif

Original Image for Figure 7A 24h 900 nM_2.tif

Original Image for Figure 6A 600 nM_EdU.tif

Original Image for Figure 7A 36h 300 nM_3.tif

Original Image for Figure 7A 12h 900 nM_3.tif

Original Image for Figure 7C 900 nM.TIF

Original Image for Fig 5B_p_FAK Y576 577.tif

Original Image for Figure 7A 12h 900 nM_2.tif

Original Image for Figure 7A 0h Control_2.tif

## Data Availability

Additional data may be requested from the authors.
