# Supplementary material for: Discovery of novel 2,4-diarylaminopyrimidine hydrazone derivatives as potent anti-thyroid cancer agents capable of inhibiting FAK
Source: J Enzyme Inhib Med Chem. 2024 Nov 19;39(1):2423875. doi: 10.1080/14756366.2024.2423875 (PMC11578424; doi:10.1080/14756366.2024.2423875)
Supplement: Supplemental Material [file IENZ_A_2423875_SM3717.pdf]

Discovery of novel 2,4-diarylaminopyrimidine hydrazone derivatives as potent anti-thyroid cancer agents capable of inhibiting FAK

Hongting Li [a, #, *], Mei-Qi Jia [b, #], Zhao-Long Qin [c], Changliang Lu [d], Weili Chu [e], Ze Zhang [a], Jinbo Niu [f], Jian Song [b], Sai-Yang Zhang [b], Lijun Fu [a, *]

a. Department of Thyroid Surgery, the First Affiliated Hospital of Zhengzhou University, the construction of east road, Erqi district, Zhengzhou,450052, Henan Province, China.

b. School of Basic Medical Sciences, Zhengzhou University, Zhengzhou 450001, Henan Province, China.

c. School of Pharmaceutical Sciences, Institute of Drug Discovery & Development Key, Laboratory of Advanced Drug Preparation Technologies (Ministry of Education), Zhengzhou University, Zhengzhou 450001, Henan Province, China.

d. Zhengzhou Xingyuan Foreign Language High School, Zhengzhou 450045, Henan Province, China.

e. Department of Respiratory and Critical Care Medicine, the First Affiliated Hospital of Zhengzhou University, the construction of east road, Erqi district, Zhengzhou,450052, Henan Province, China.

f. The Third Affiliated Hospital of Zhengzhou University, Zhengzhou, 450052, China.

* Corresponding author: Hong-Ting Li (tingting2003@126.com) and Lijun Fu (brease2003@163.com)

[#] These authors contributed equally to this work

- ^{1}H, ^{13}C-NMR and HRMS of compound **14a**

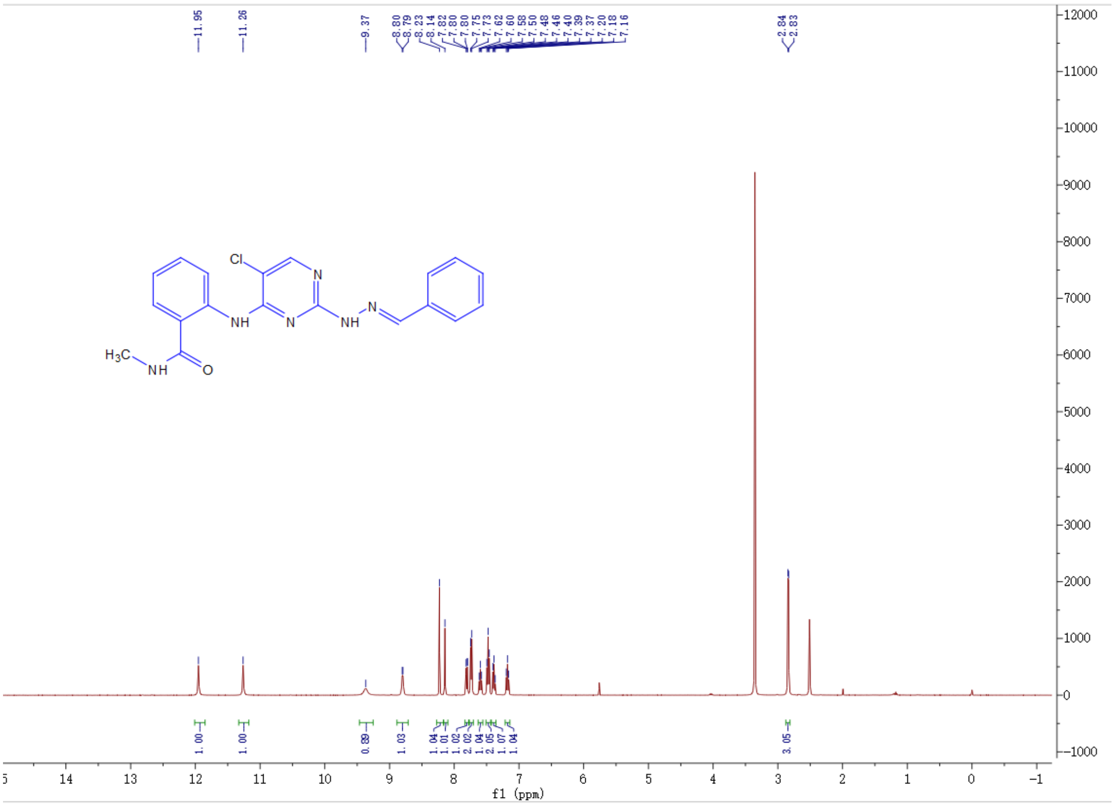

Figure S1. ^{1}H NMR spectrum of compound **14a** (400 MHz, DMSO-*d6*)

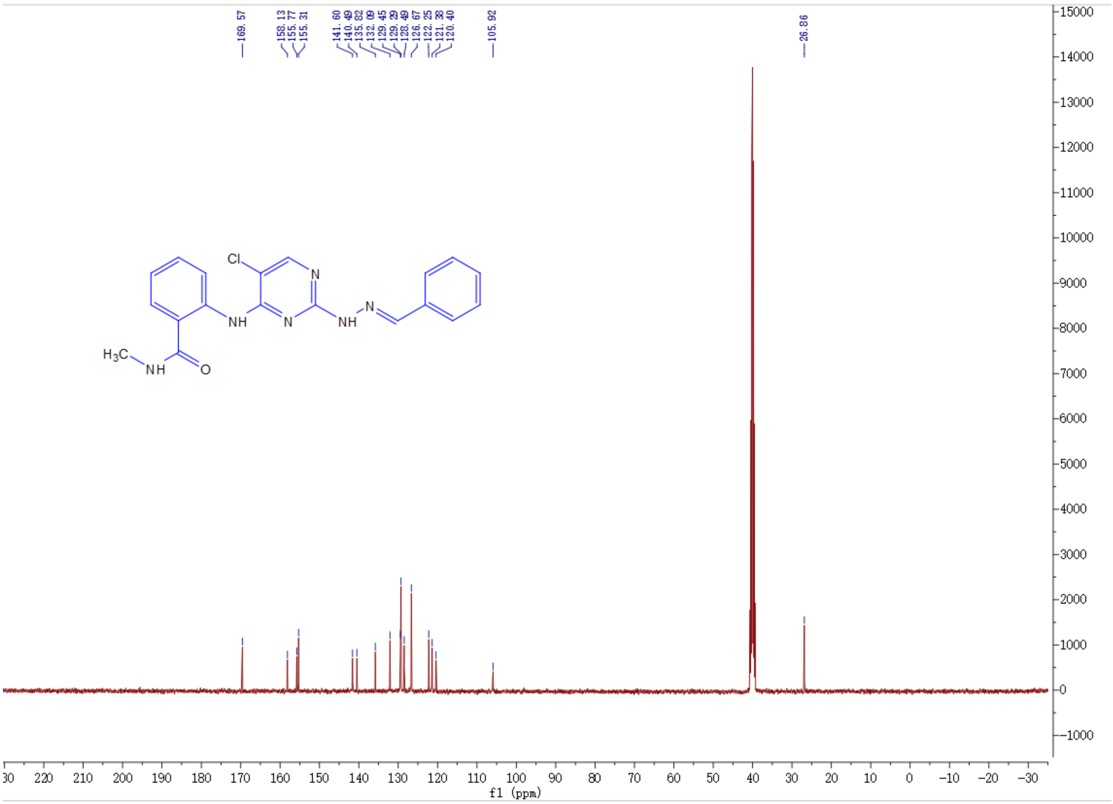

Figure S2. ^{13}C NMR spectrum of compound **14a** (100 MHz, DMSO-*d6*)

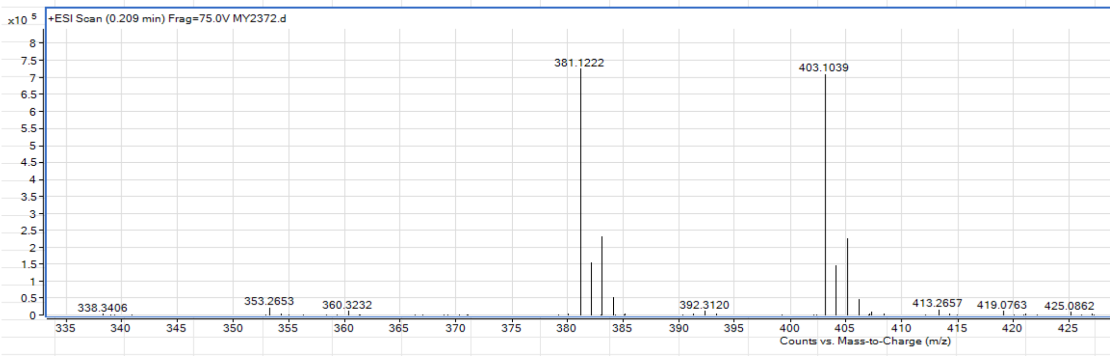

Figure S3. HRMS spectrum of compound **14a**

- ^{1}H, ^{13}C-NMR and HRMS of compound **14b**

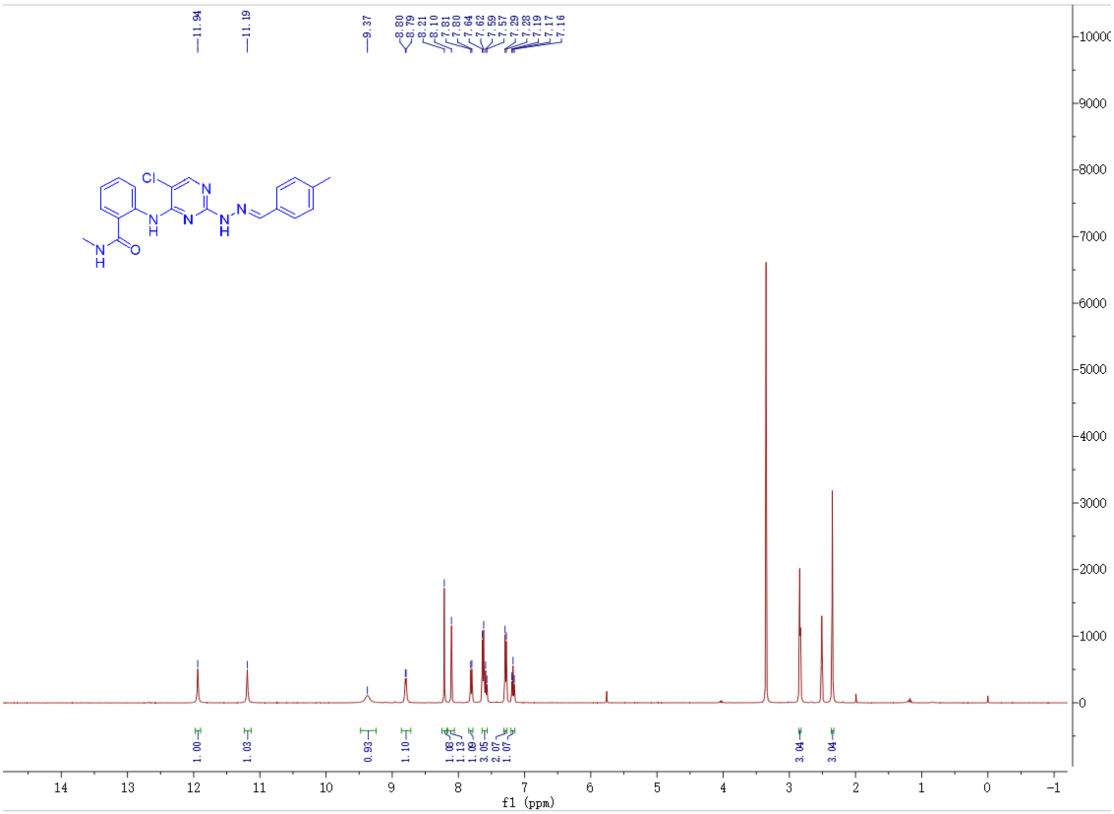

Figure S4. ^{1}H NMR spectrum of compound **14b** (400 MHz, DMSO-*d6*)

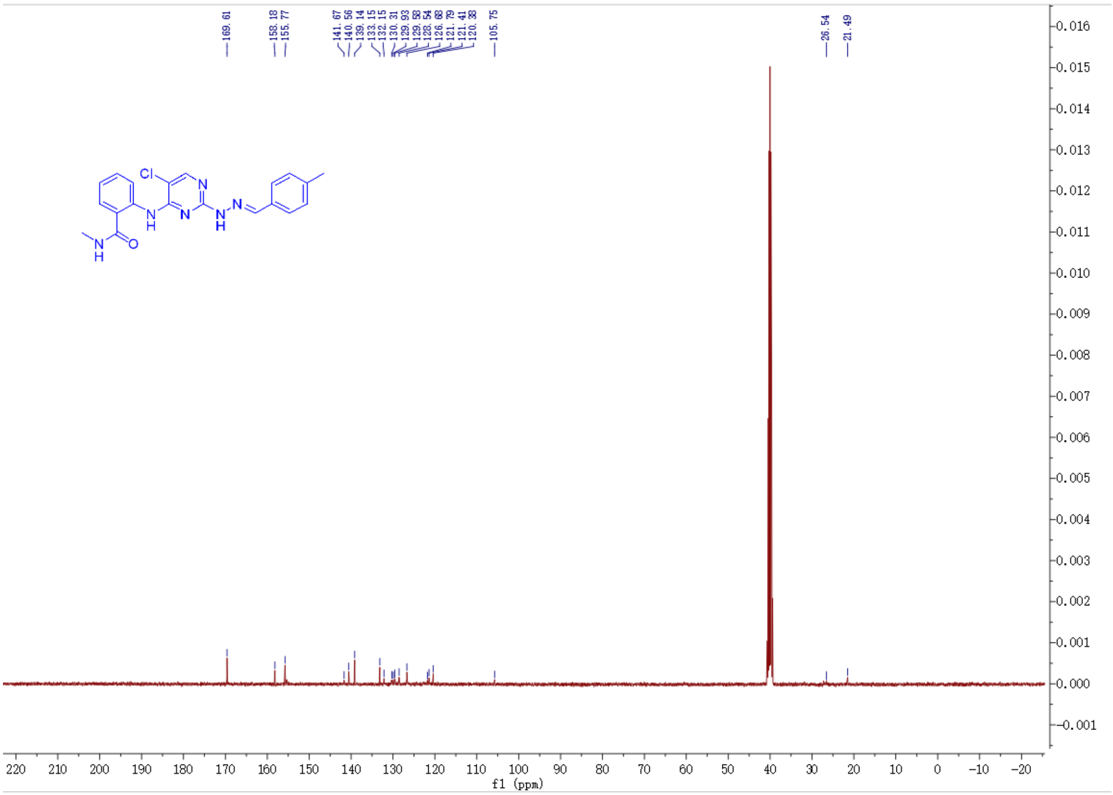

Figure S5. ^{13}C NMR spectrum of compound **14b** (100 MHz, DMSO-*d6*)

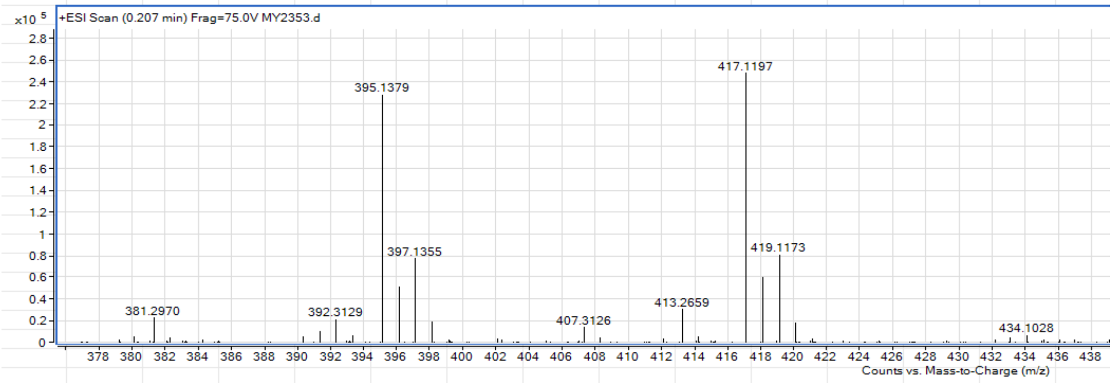

Figure S6. HRMS spectrum of compound **14b**

● ^{1}H, ^{13}C-NMR and HRMS of compound **14c**

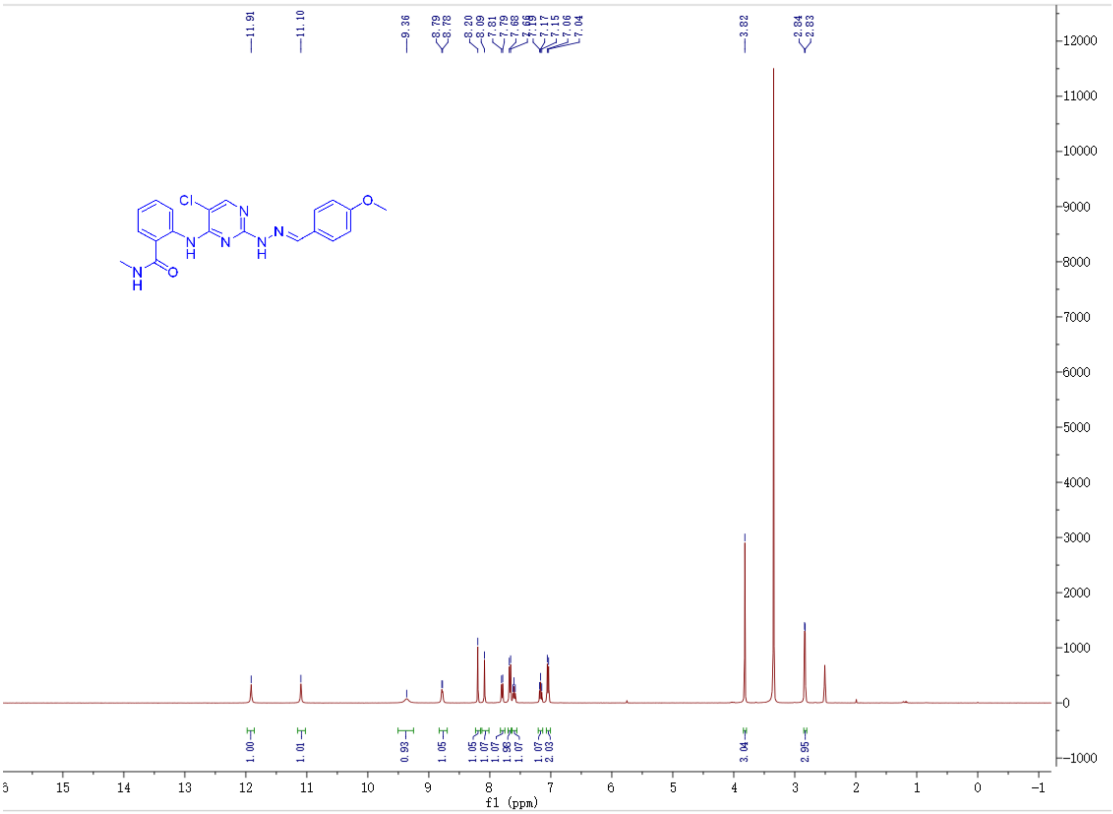

Figure S7. ^{1}H NMR spectrum of compound **14c** (400 MHz, DMSO-*d6*)

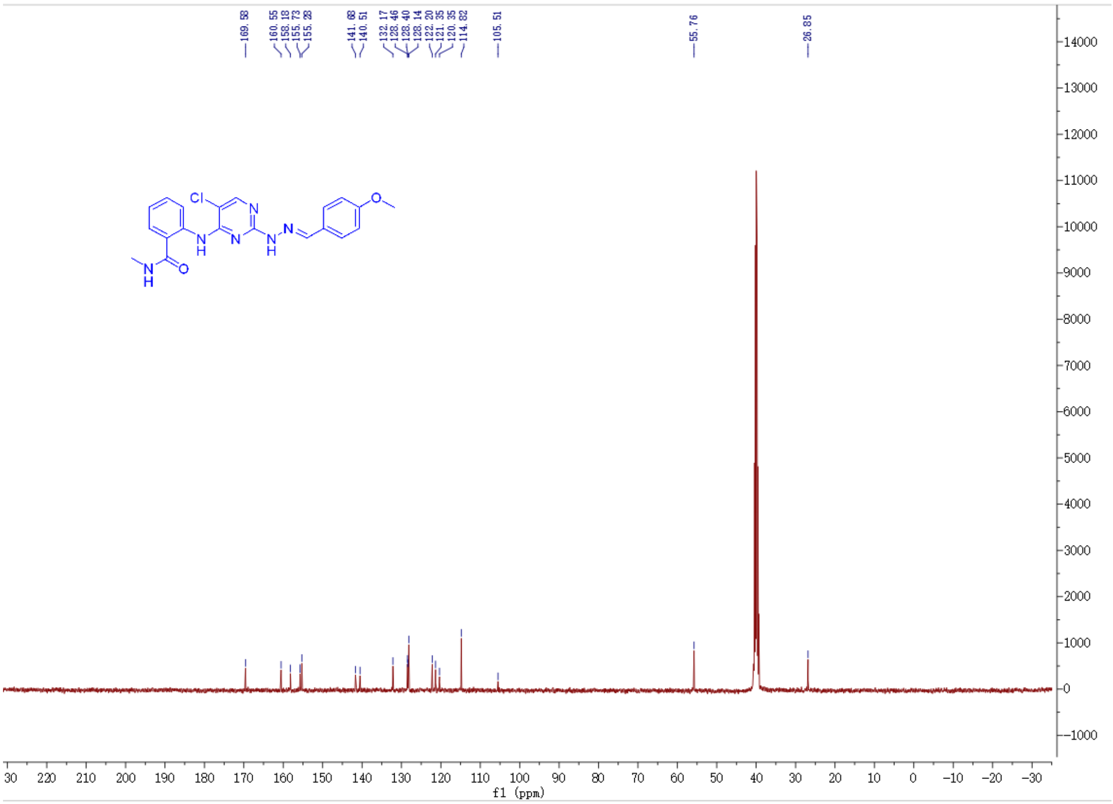

Figure S8. ^{13}C NMR spectrum of compound **14c** (100 MHz, DMSO-*d6*)

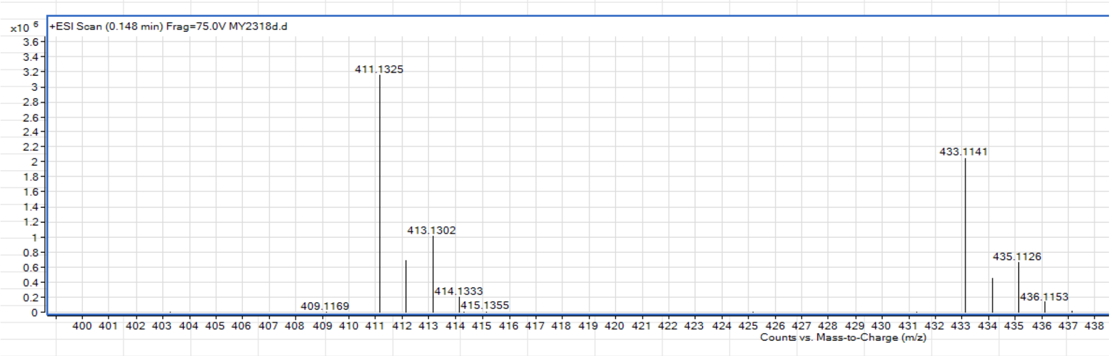

Figure S9. HRMS spectrum of compound **14c**

- ^{1}H, ^{13}C-NMR and HRMS of compound **14d**

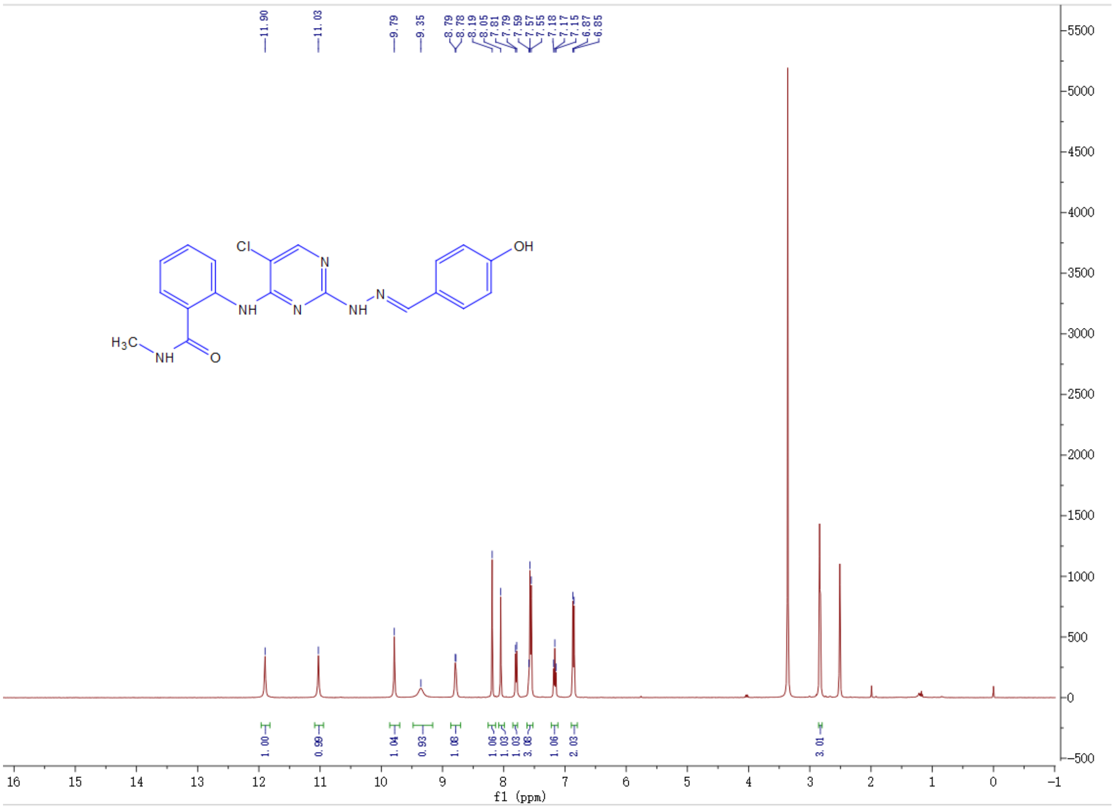

Figure S10. ^{1}H NMR spectrum of compound **14d** (400 MHz, DMSO-*d6*)

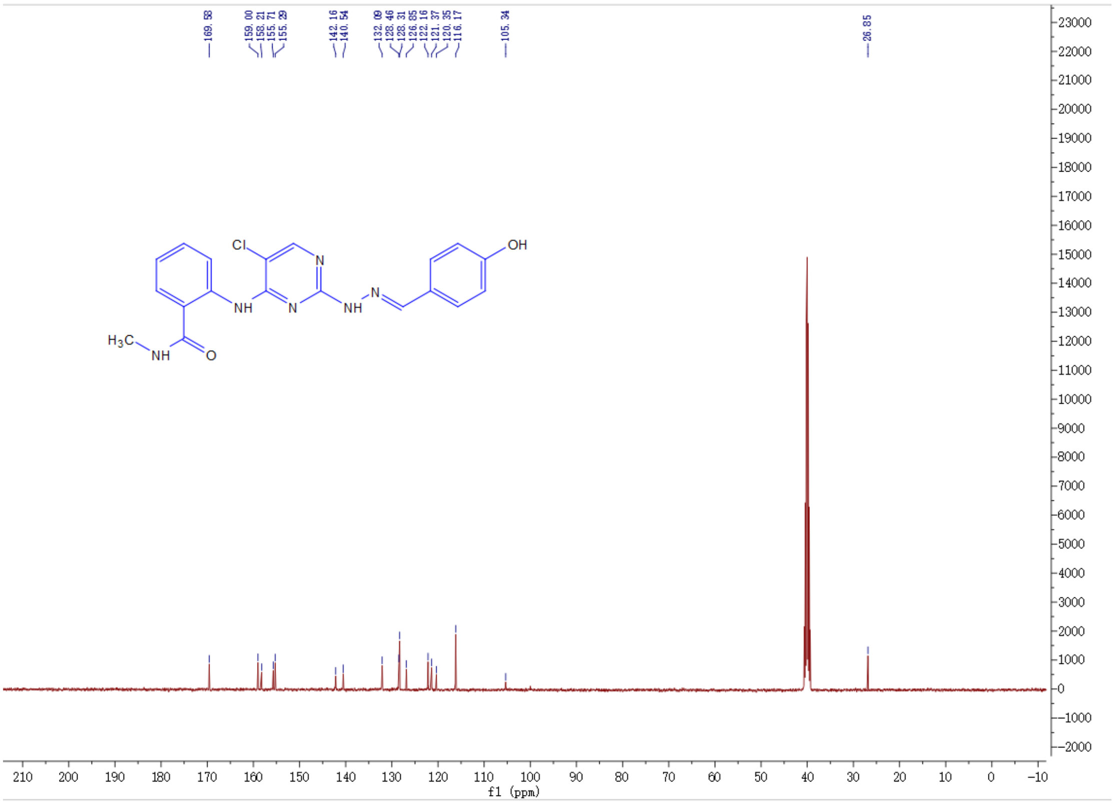

Figure S11. ^{13}C NMR spectrum of compound **14d** (100 MHz, DMSO-*d6*)

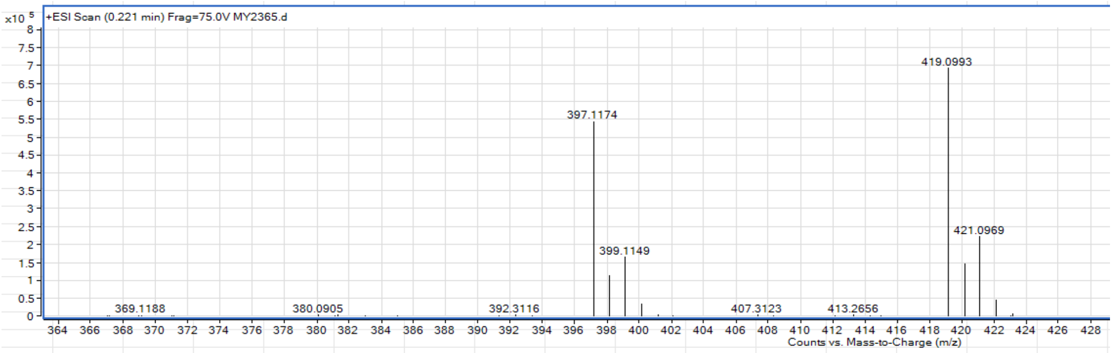

Figure S12. HRMS spectrum of compound **14d**

● ^{1}H, ^{13}C-NMR and HRMS of compound **14e**

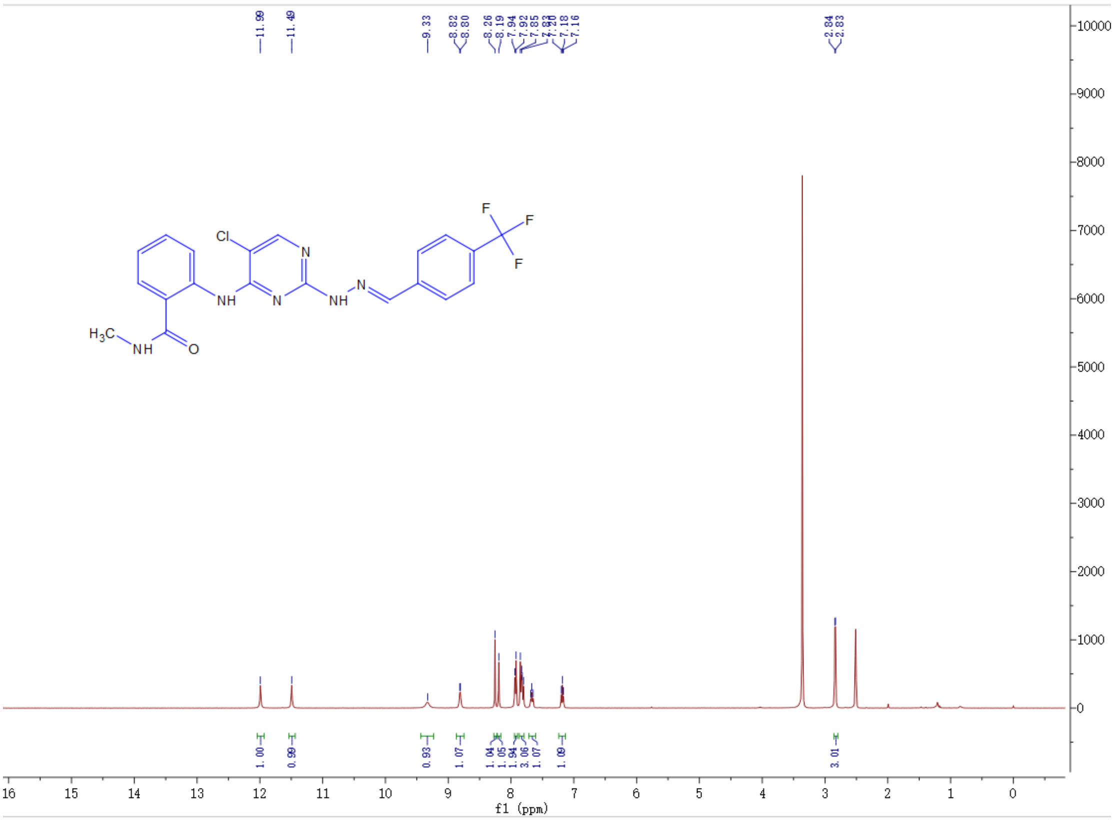

Figure S13. ^{1}H NMR spectrum of compound **14e** (400 MHz, DMSO-*d6*)

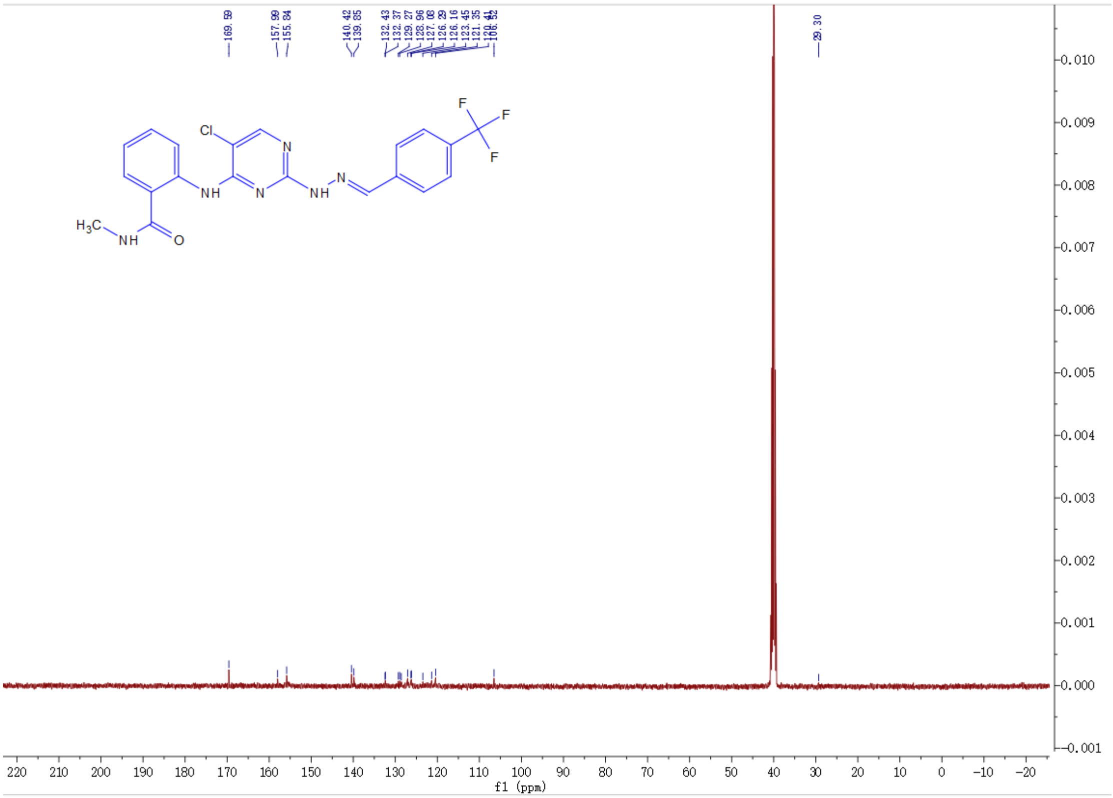

Figure S14. ^{13}C NMR spectrum of compound **14e** (100 MHz, DMSO-*d6*)

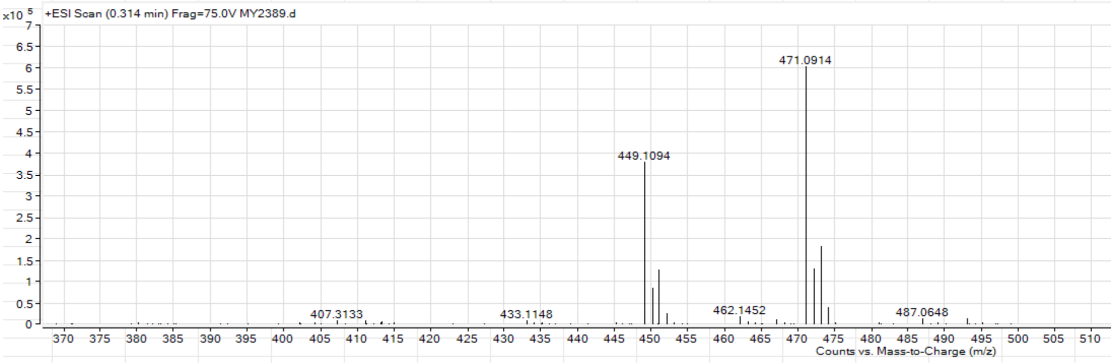

Figure S15. HRMS spectrum of compound **14e**

- ^{1}H, ^{13}C-NMR and HRMS of compound **14f**

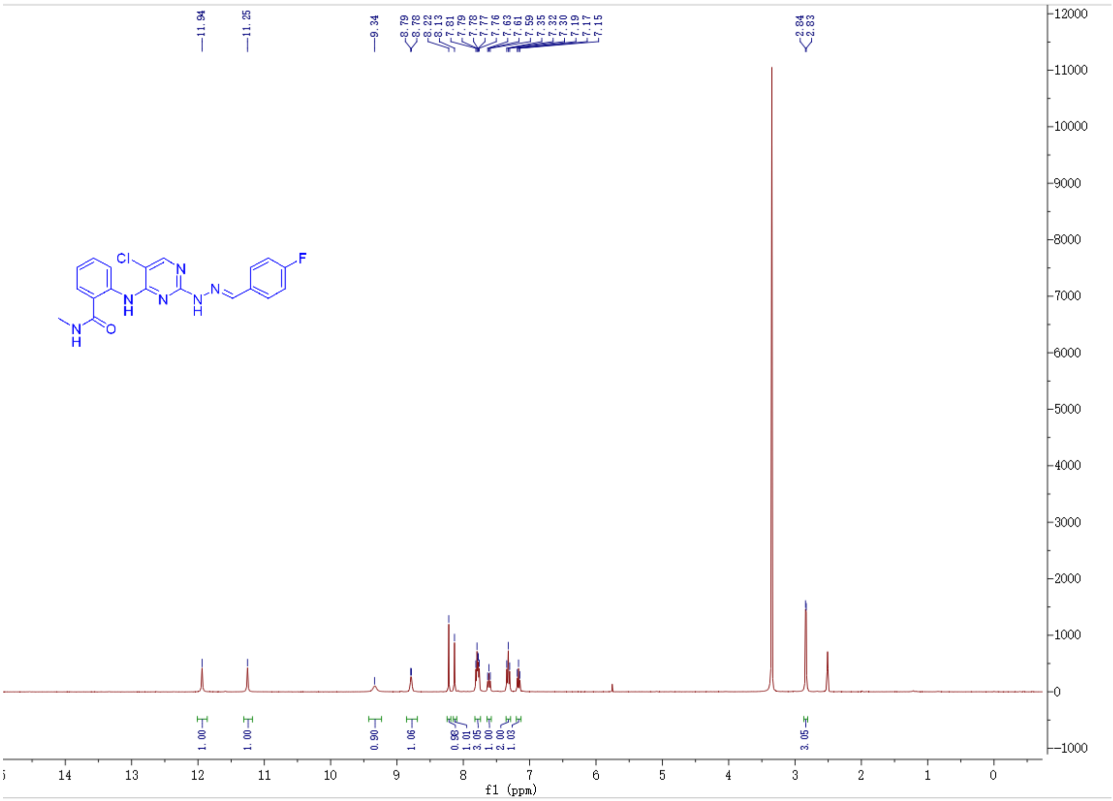

Figure S16. ^{1}H NMR spectrum of compound **14f** (400 MHz, DMSO-*d6*)

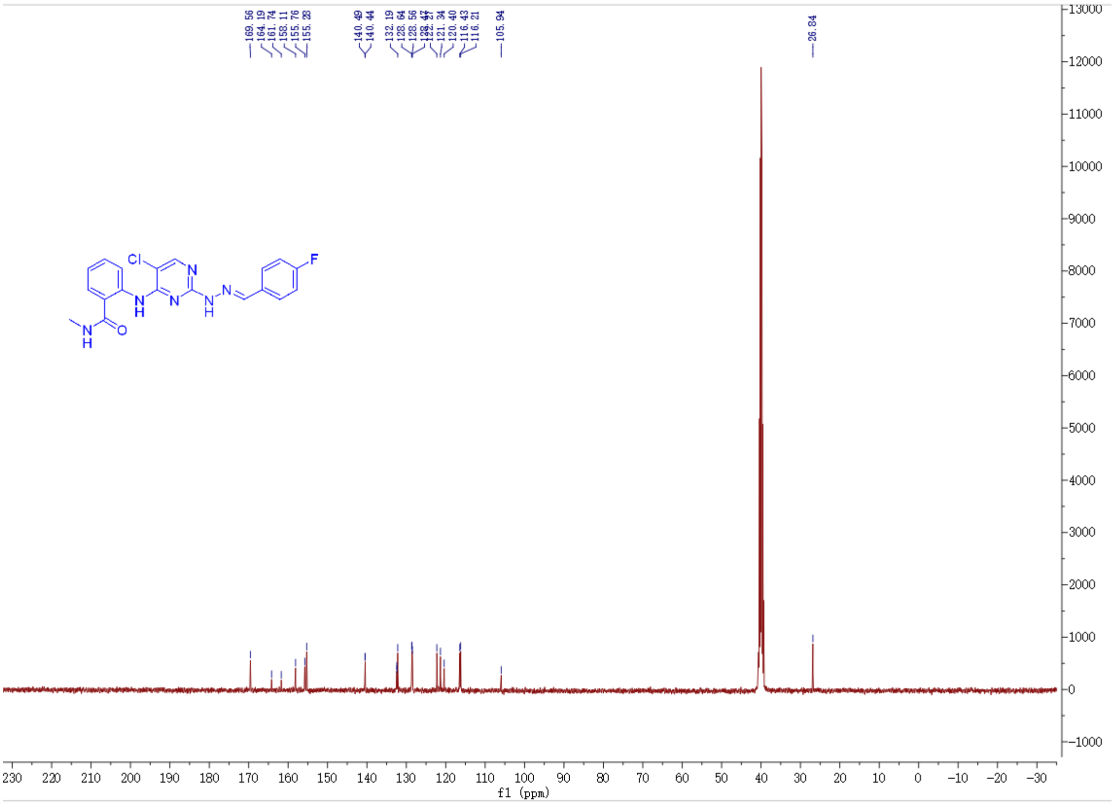

Figure S17. ^{13}C NMR spectrum of compound **14f** (100 MHz, DMSO-*d6*)

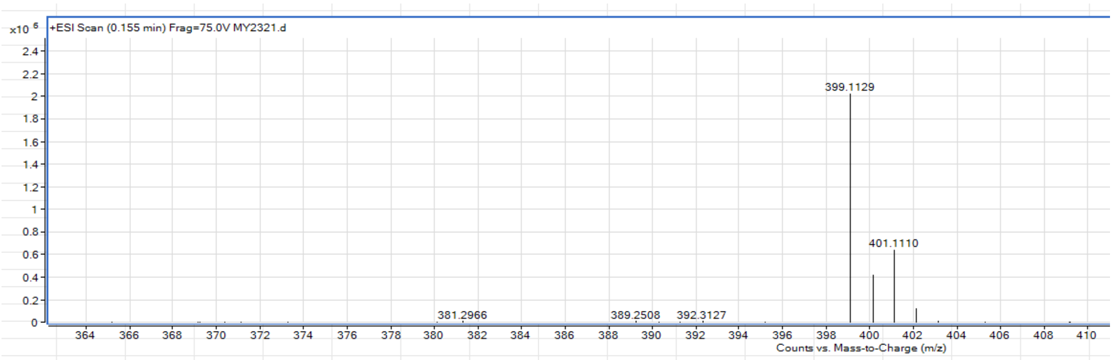

Figure S18. HRMS spectrum of compound **14f**

● ^{1}H, ^{13}C-NMR and HRMS of compound **14g**

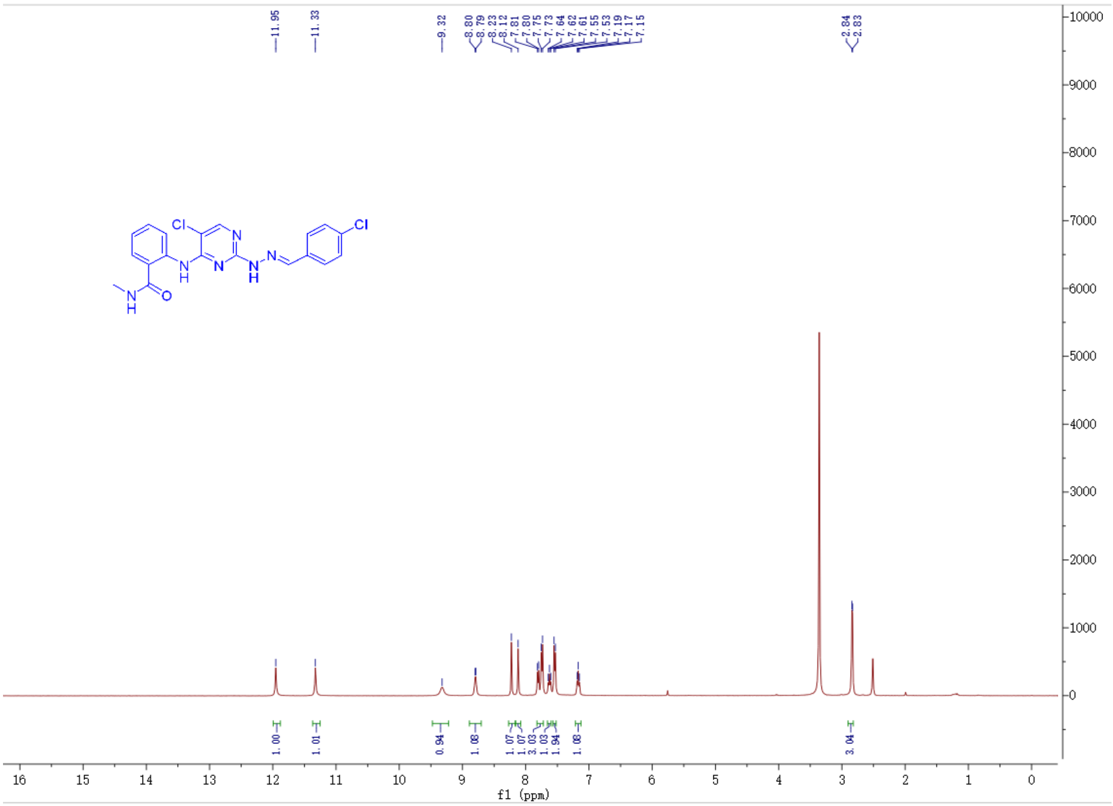

Figure S19. ^{1}H NMR spectrum of compound **14g** (400 MHz, DMSO-*d6*)

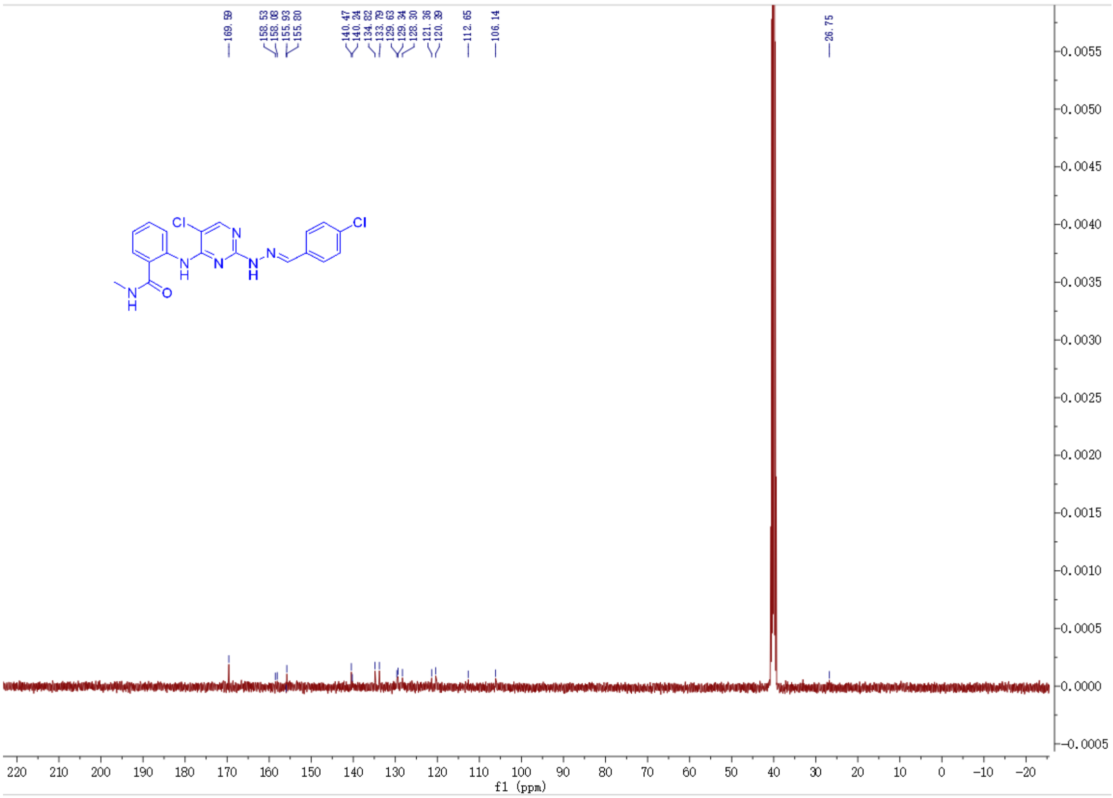

Figure S20. ^{13}C NMR spectrum of compound **14g** (100 MHz, DMSO-*d6*)

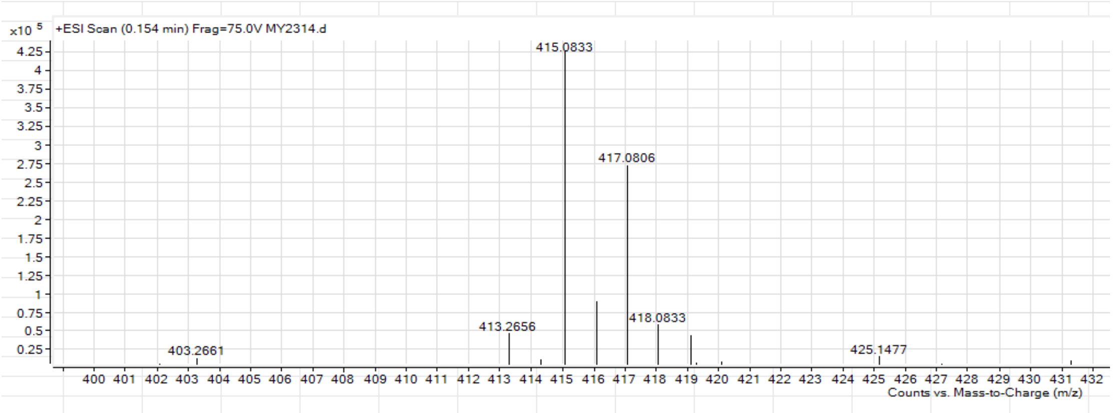

Figure S21. HRMS spectrum of compound **14g**

- ^{1}H, ^{13}C-NMR and HRMS of compound **14h**

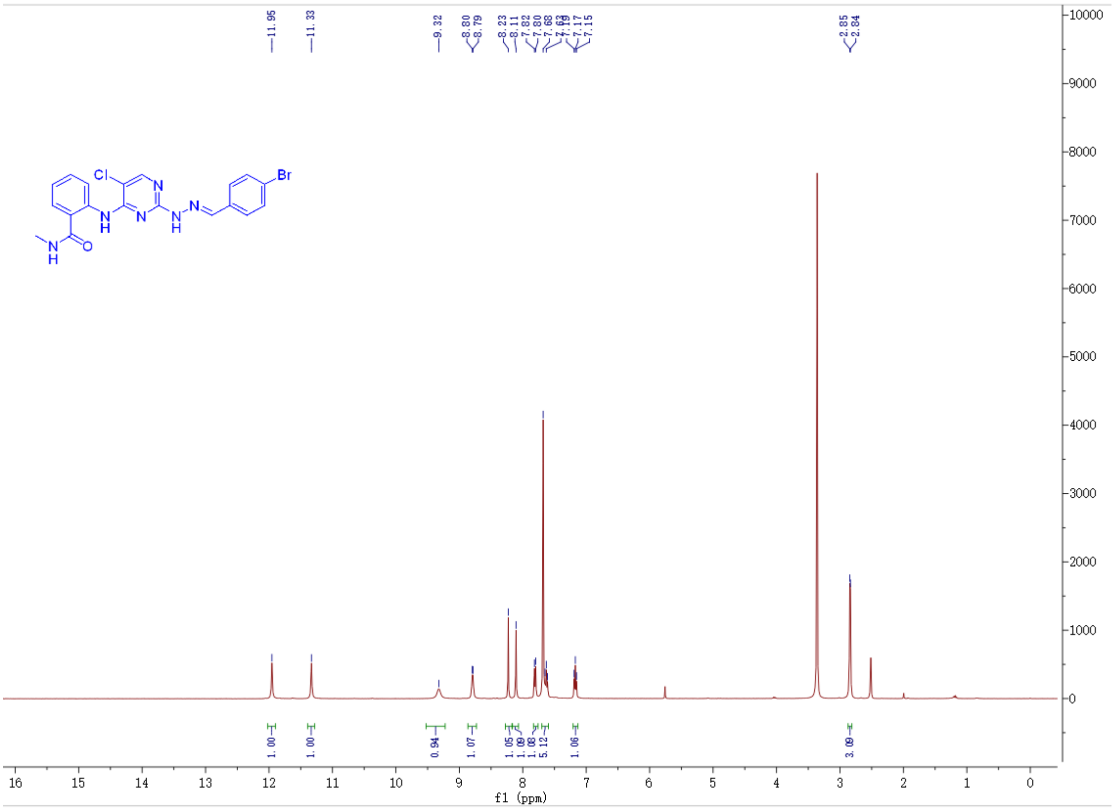

Figure S22. ^{1}H NMR spectrum of compound **14h** (400 MHz, DMSO-*d6*)

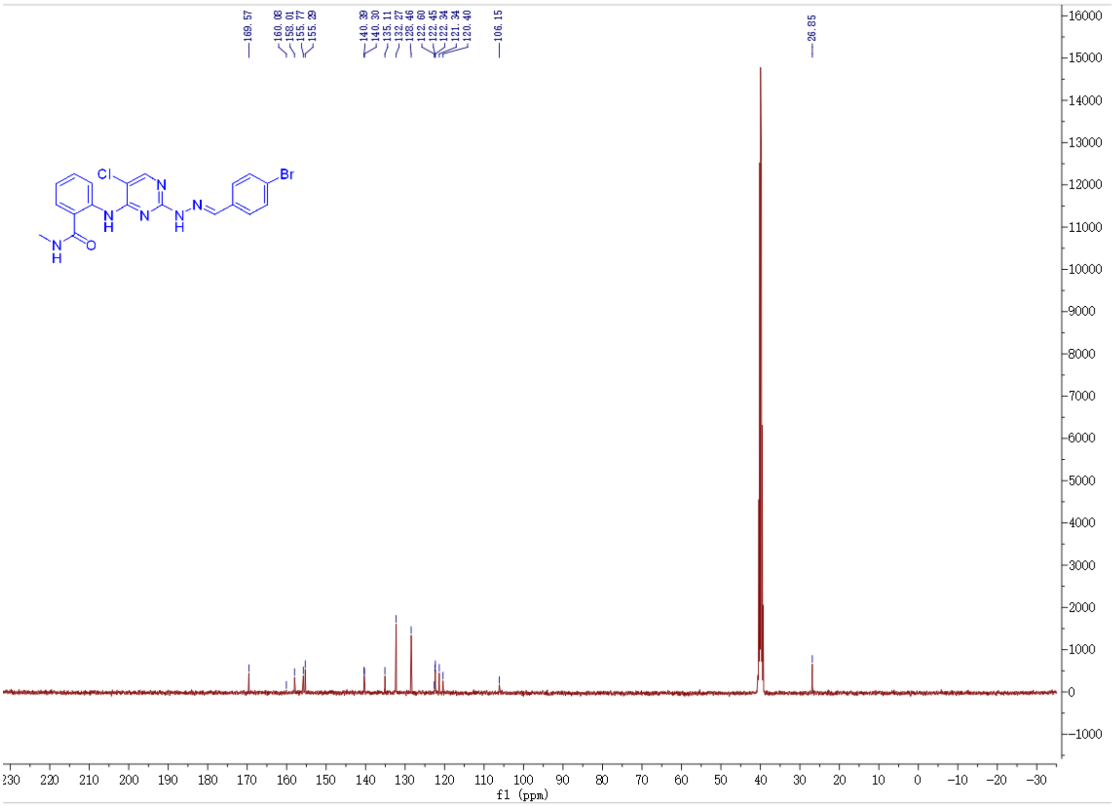

Figure S23. ^{13}C NMR spectrum of compound **14h** (100 MHz, DMSO-*d6*)

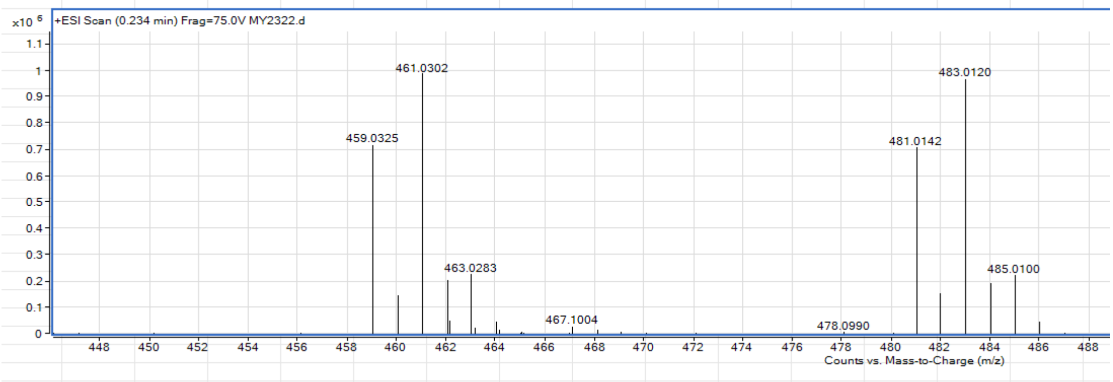

Figure S24. HRMS spectrum of compound **14h**

● ^{1}H, ^{13}C-NMR and HRMS of compound **14i**

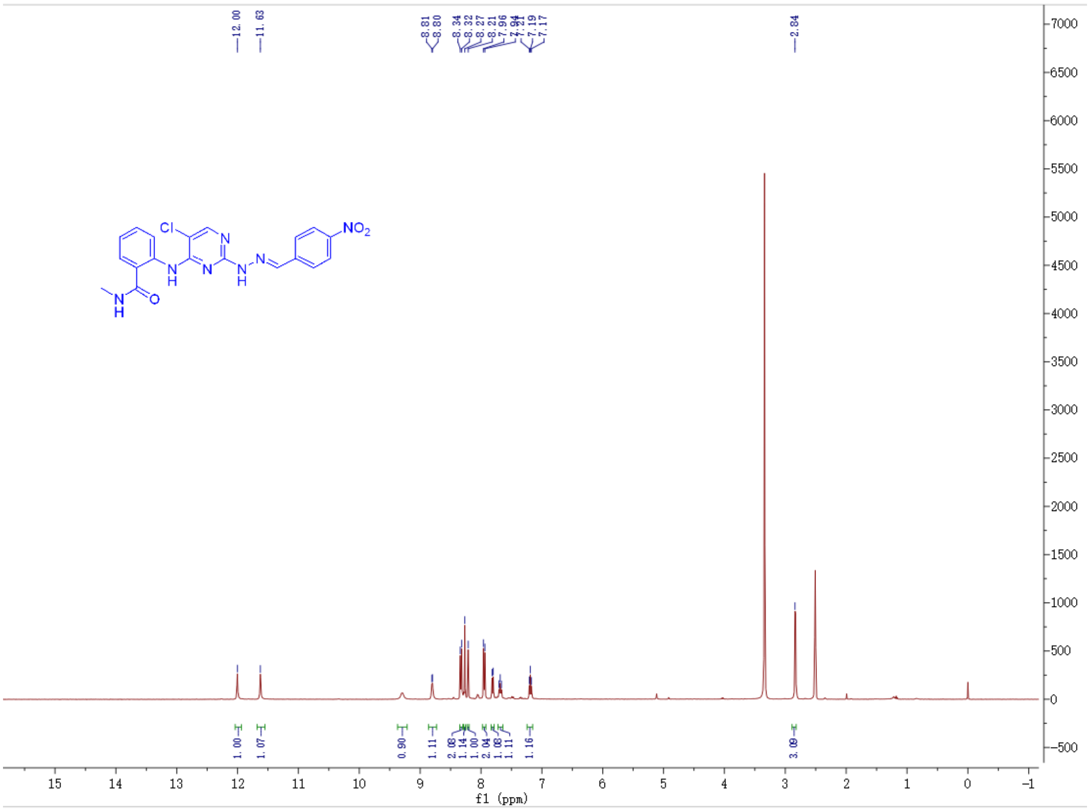

Figure S25. ^{1}H NMR spectrum of compound **14i** (400 MHz, DMSO-*d6*)

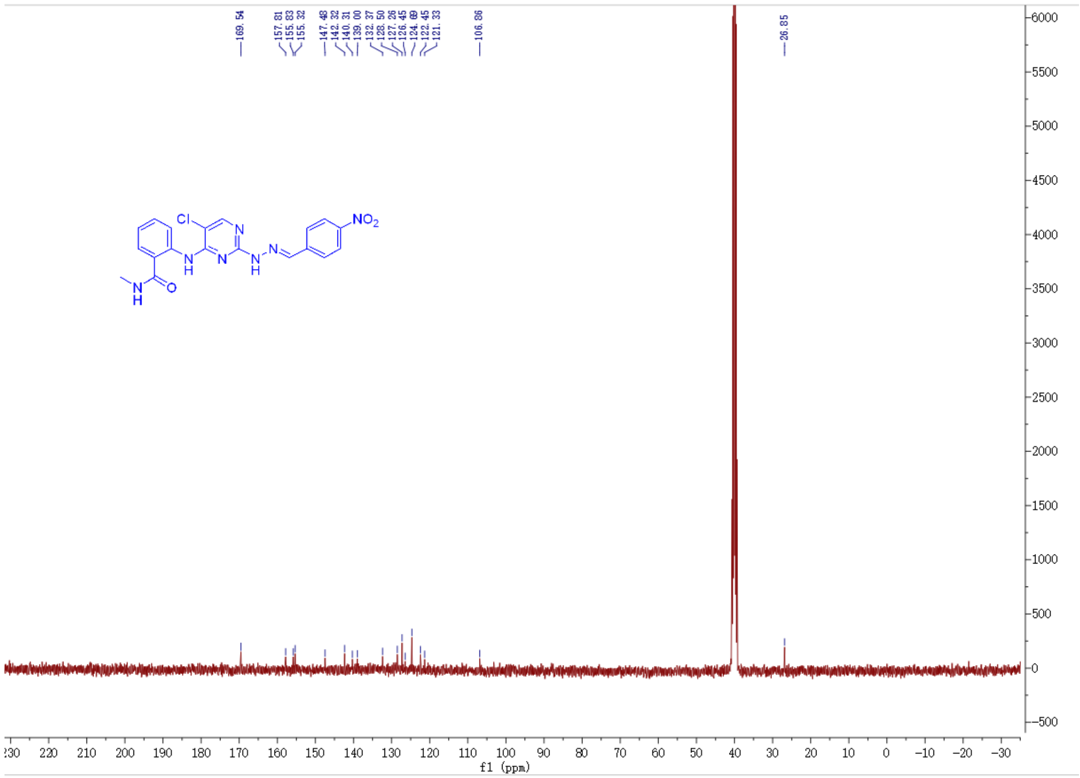

Figure S26. ^{13}C NMR spectrum of compound **14i** (100 MHz, DMSO-*d6*)

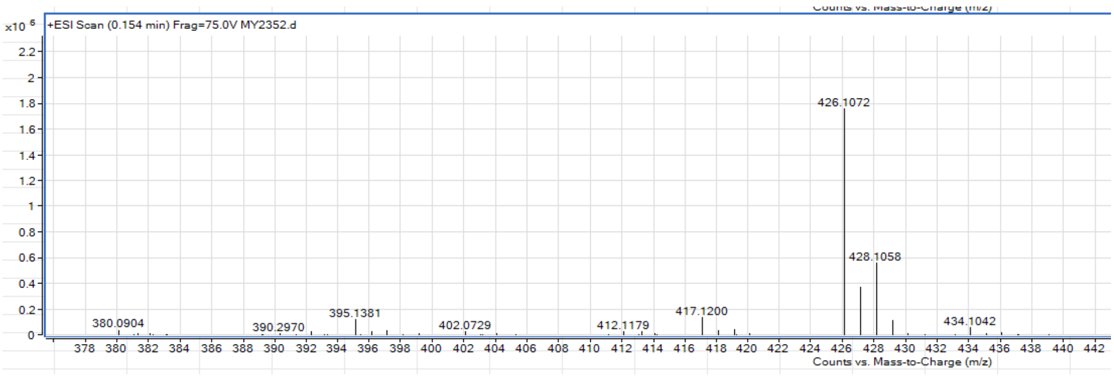

Figure S27. HRMS spectrum of compound **14i**

- ^{1}H, ^{13}C-NMR and HRMS of compound **14j**

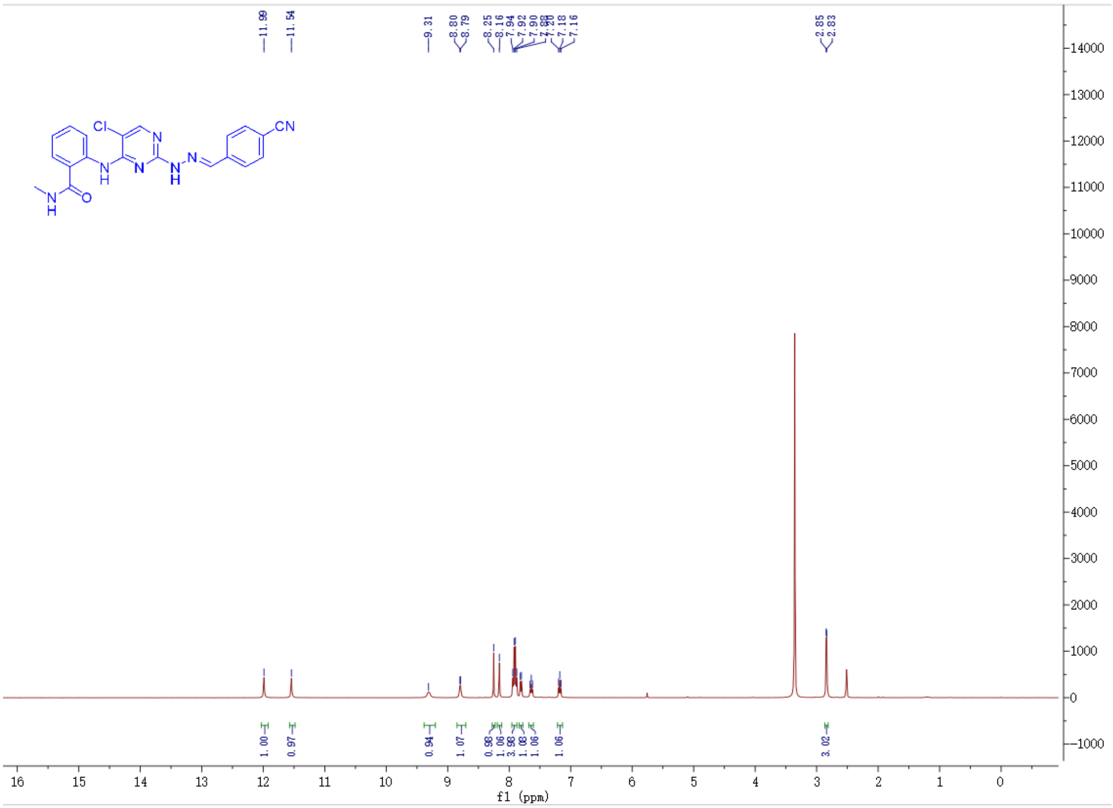

Figure S28. ^{1}H NMR spectrum of compound **14j** (400 MHz, DMSO-*d6*)

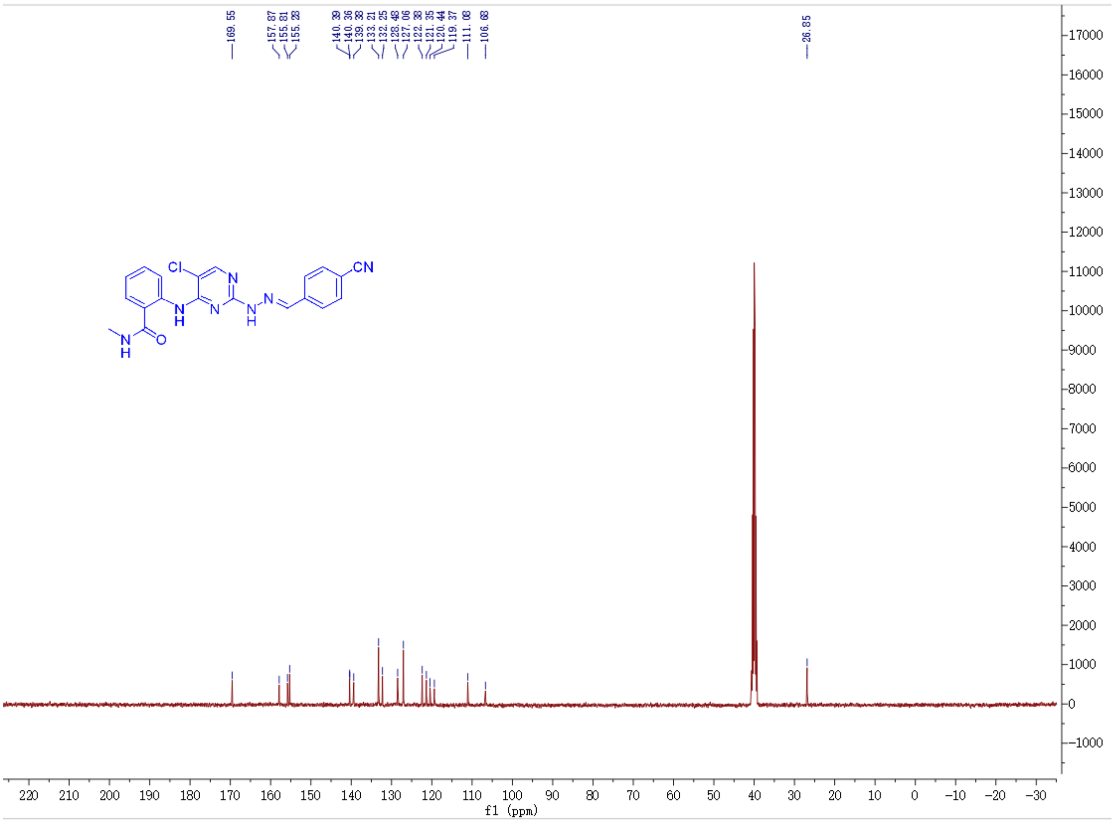

Figure S29. ^{13}C NMR spectrum of compound **14j** (100 MHz, DMSO-*d6*)

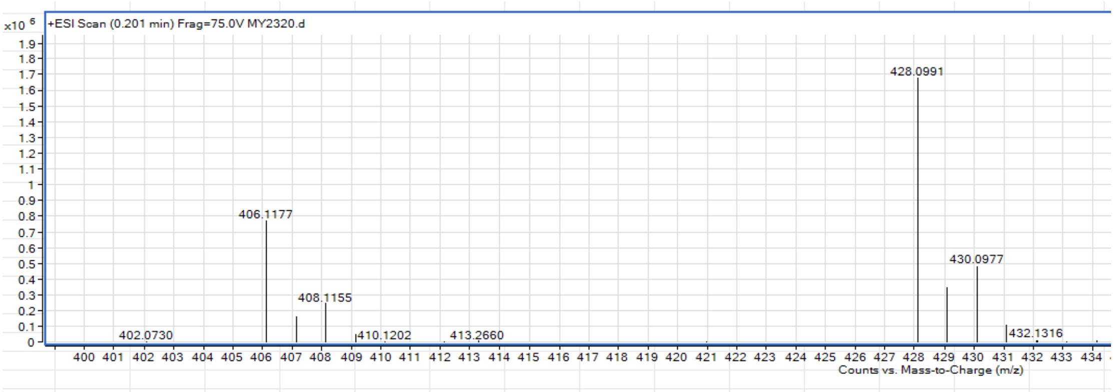

Figure S30. HRMS spectrum of compound **14j**

● ^{1}H, ^{13}C-NMR and HRMS of compound **14k**

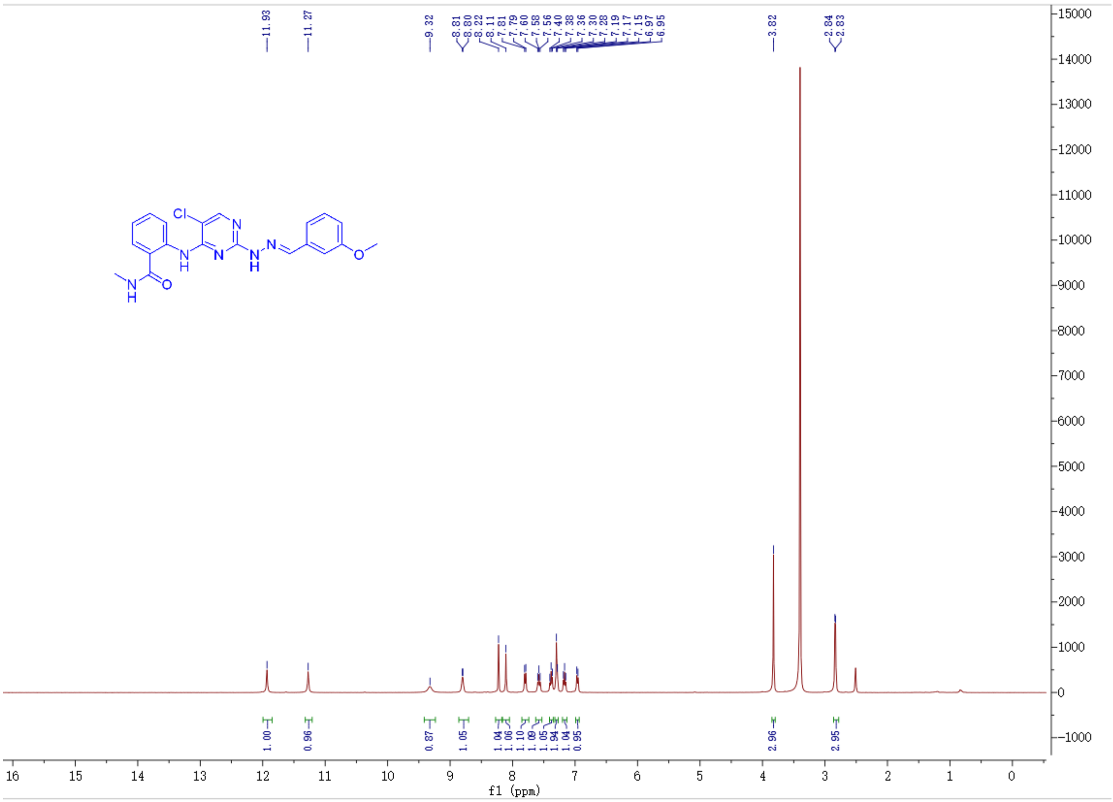

Figure S31. ^{1}H NMR spectrum of compound **14k** (400 MHz, DMSO-*d6*)

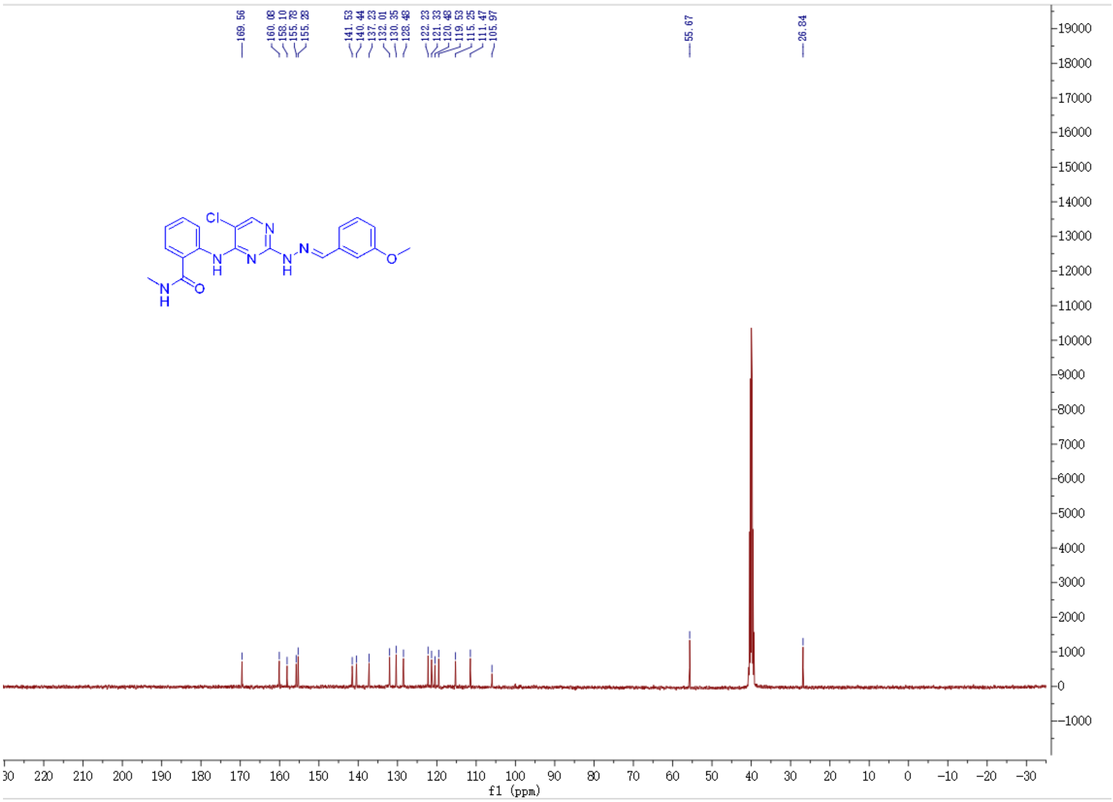

Figure S32. ^{13}C NMR spectrum of compound **14k** (100 MHz, DMSO-*d6*)

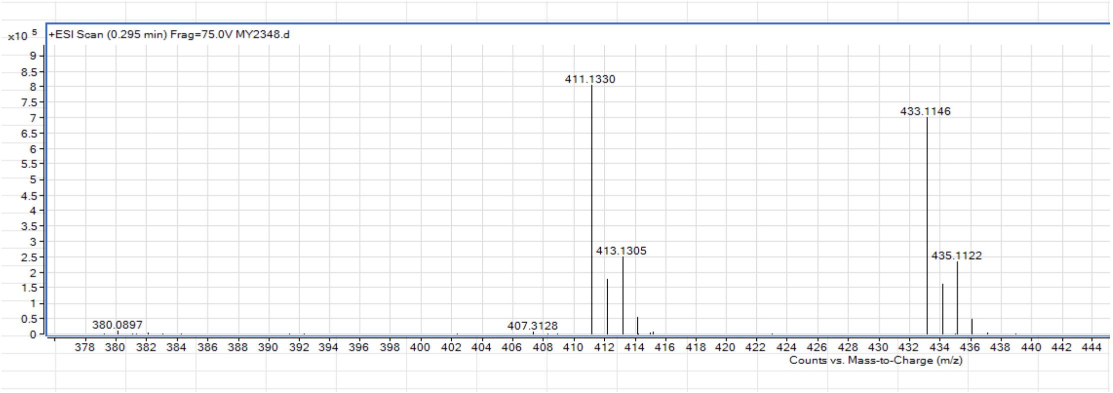

Figure S33. HRMS spectrum of compound **14k**

- ^{1}H, ^{13}C-NMR and HRMS of compound **14l**

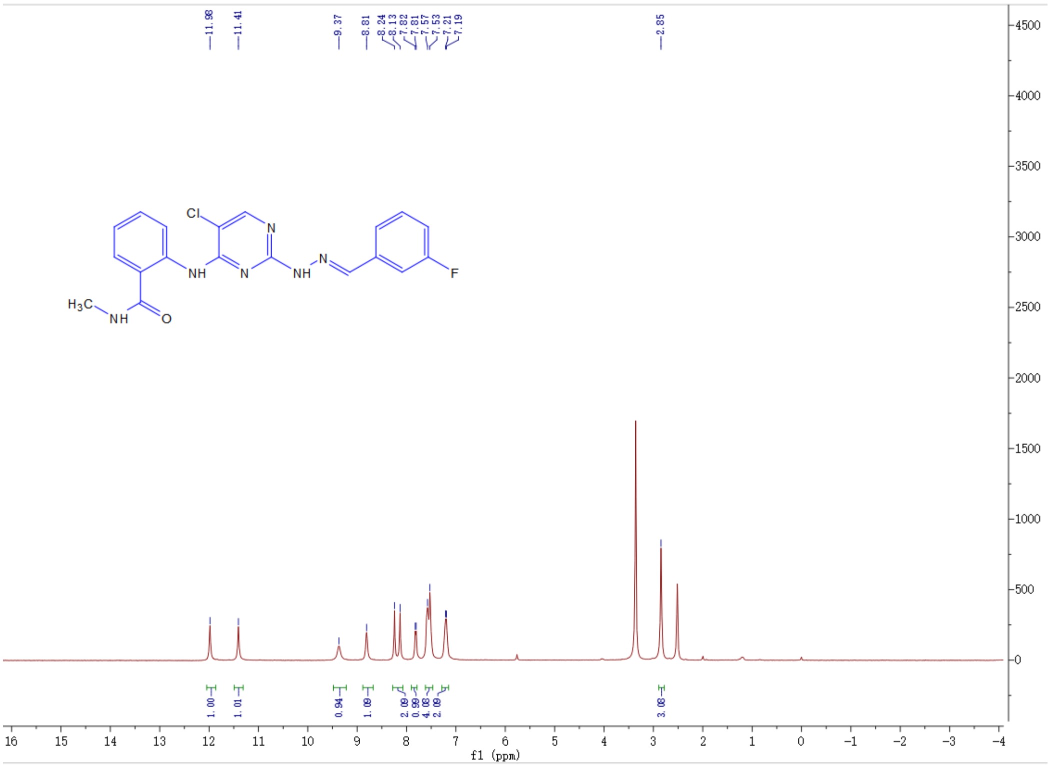

Figure S34. ^{1}H NMR spectrum of compound **14l** (400 MHz, DMSO-*d6*)

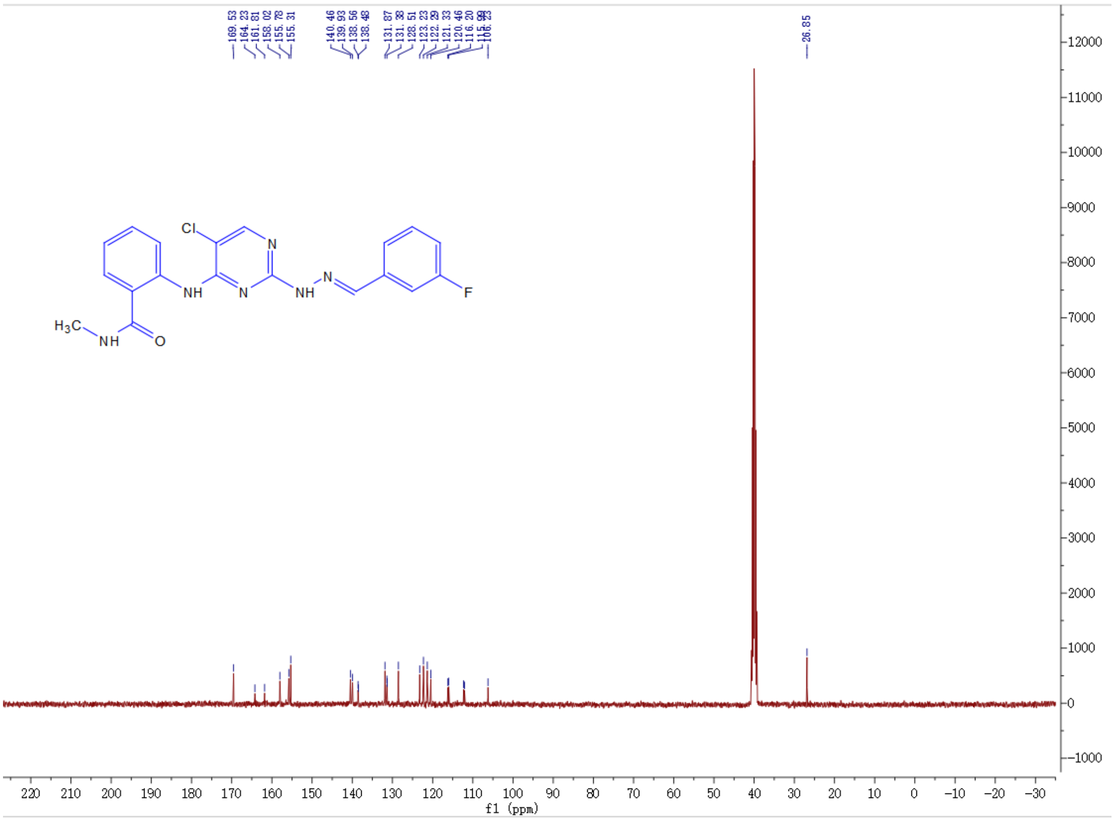

Figure S35. ^{13}C NMR spectrum of compound **14l** (100 MHz, DMSO-*d6*)

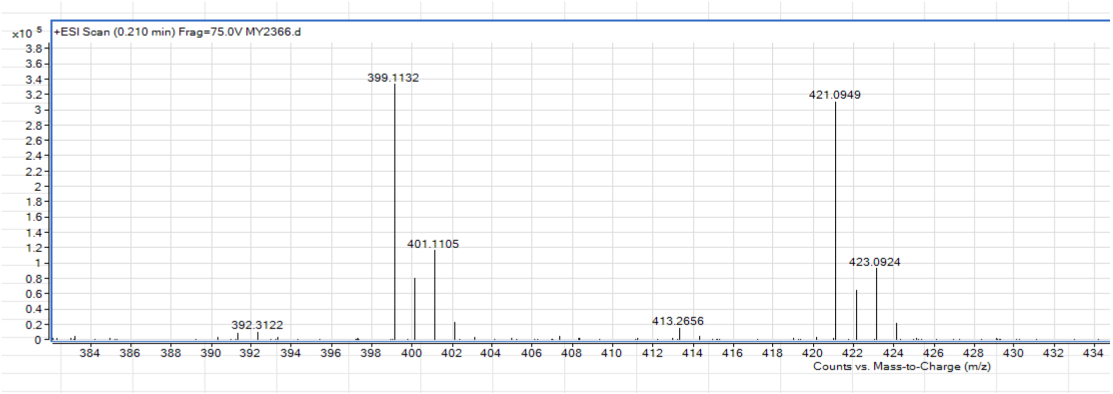

Figure S36. HRMS spectrum of compound **14l**

- ^{1}H, ^{13}C-NMR and HRMS of compound **14m**

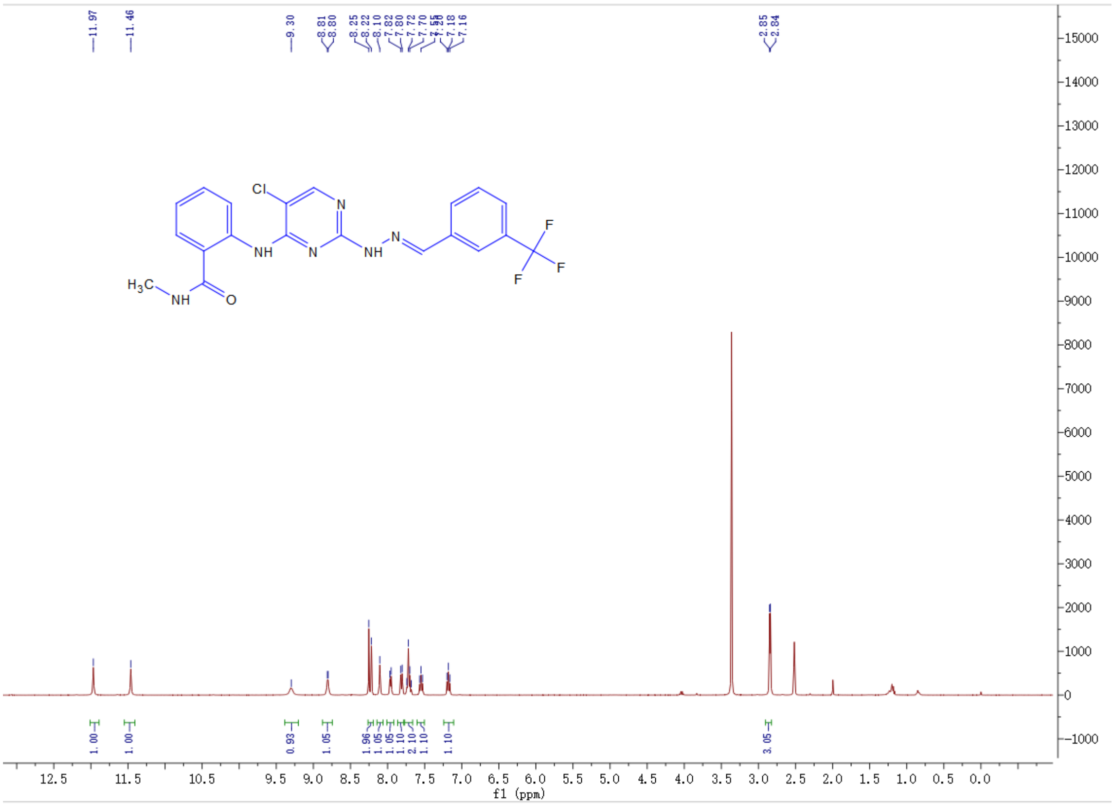

Figure S37. ^{1}H NMR spectrum of compound **14m** (400 MHz, DMSO-*d6*)

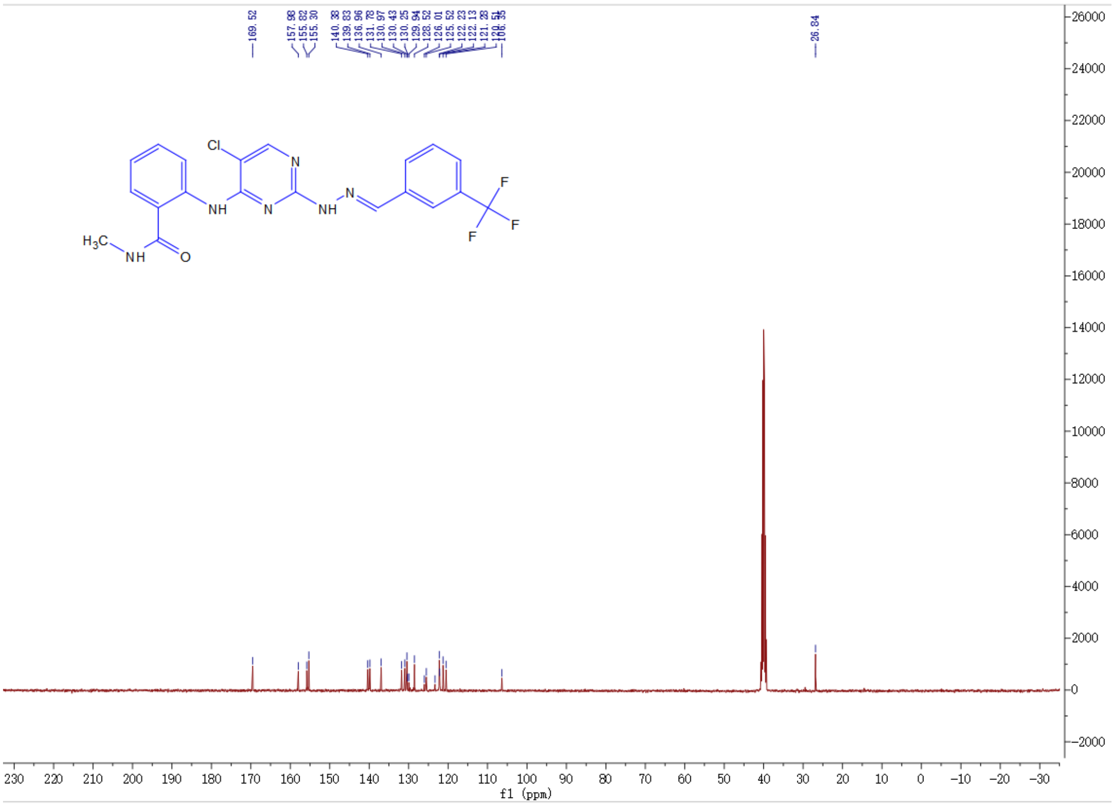

Figure S38. ^{13}C NMR spectrum of compound **14m** (100 MHz, DMSO-*d6*)

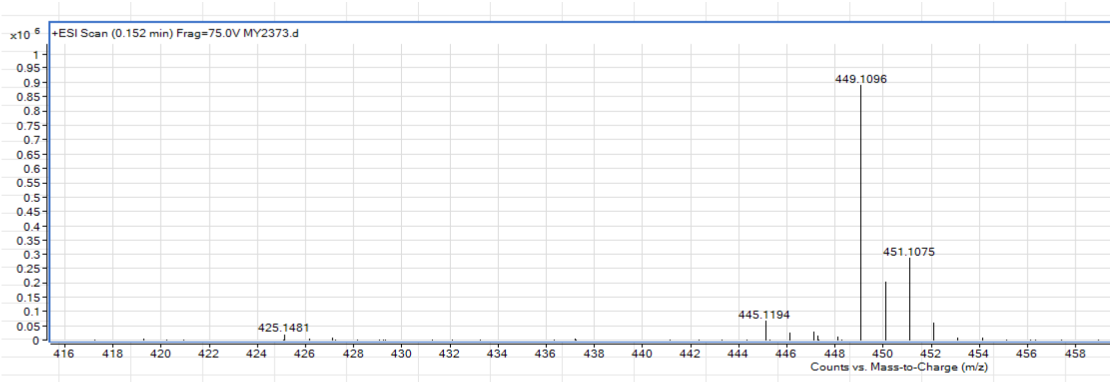

Figure S39. HRMS spectrum of compound **14m**

- ^{1}H, ^{13}C-NMR and HRMS of compound **14n**

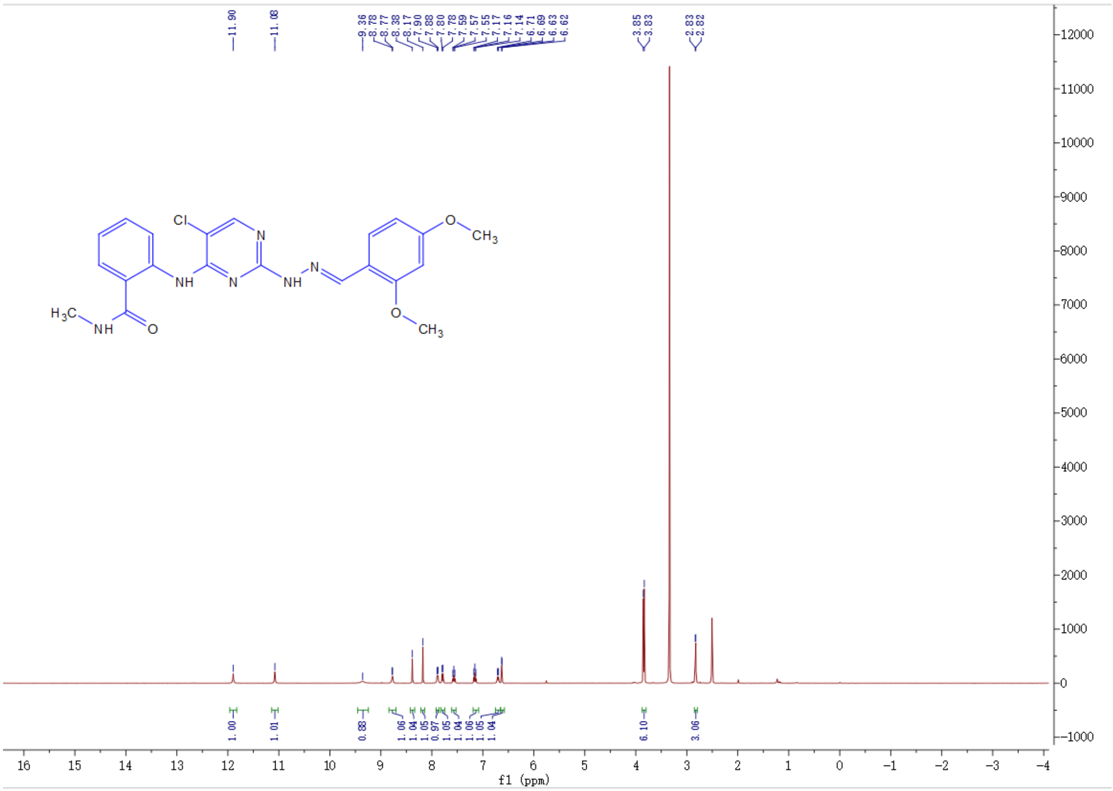

Figure S40. ^{1}H NMR spectrum of compound **14n** (400 MHz, DMSO-*d6*)

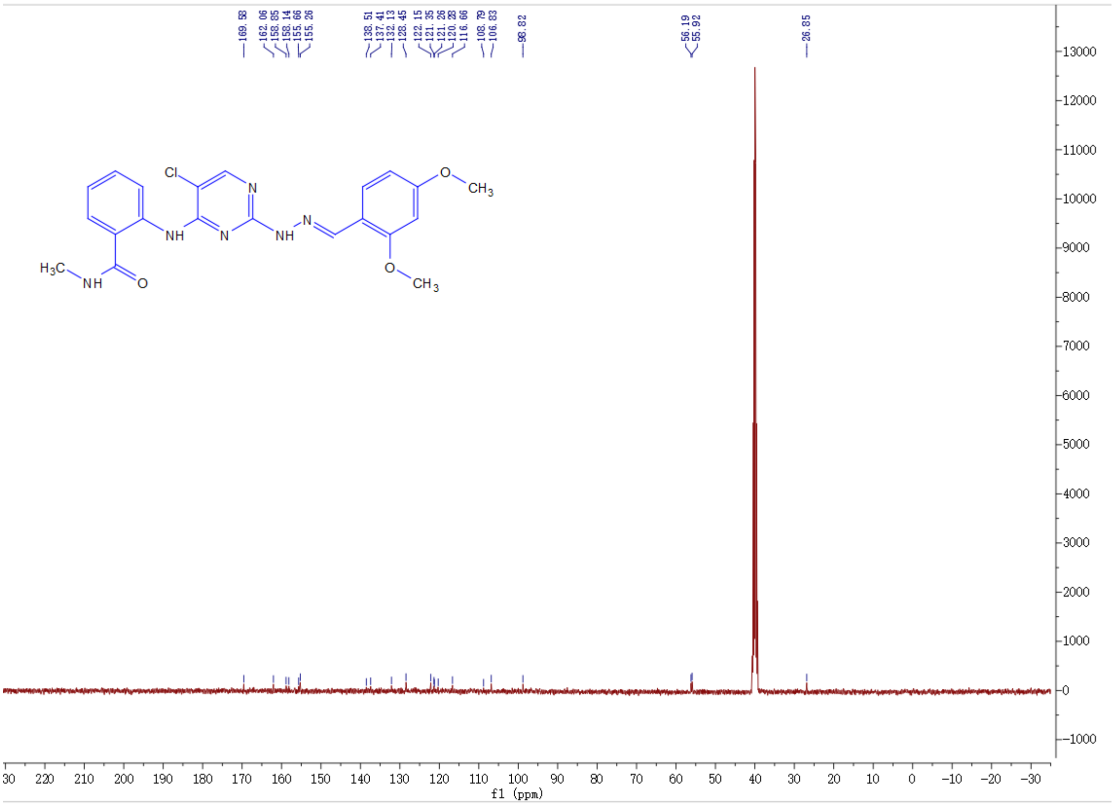

Figure S41. ^{13}C NMR spectrum of compound **14n** (100 MHz, DMSO-*d6*)

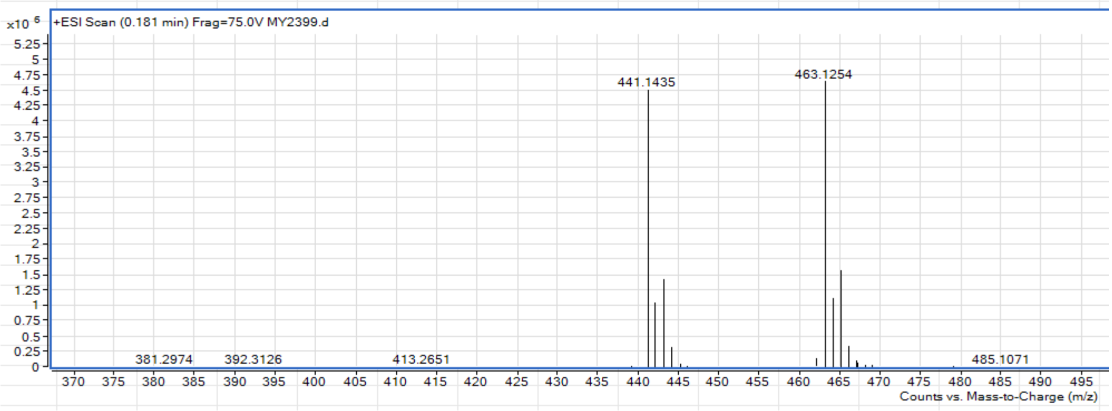

Figure S42. HRMS spectrum of compound **14n**

- ^{1}H, ^{13}C-NMR and HRMS of compound **14o**

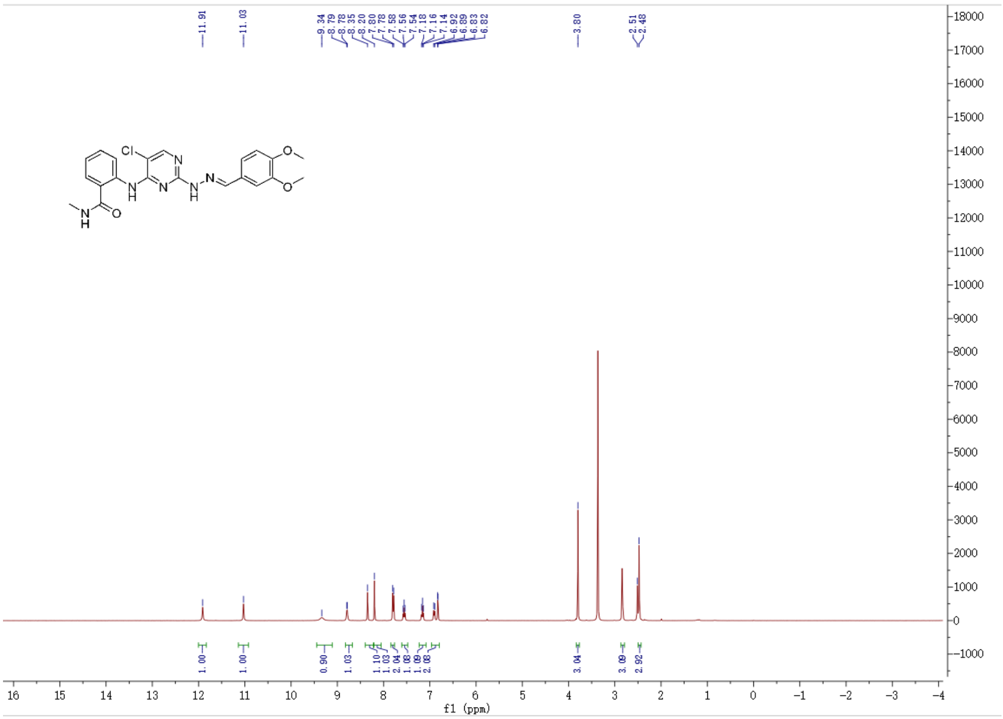

Figure S43. ^{1}H NMR spectrum of compound **14o** (400 MHz, DMSO-*d6*)

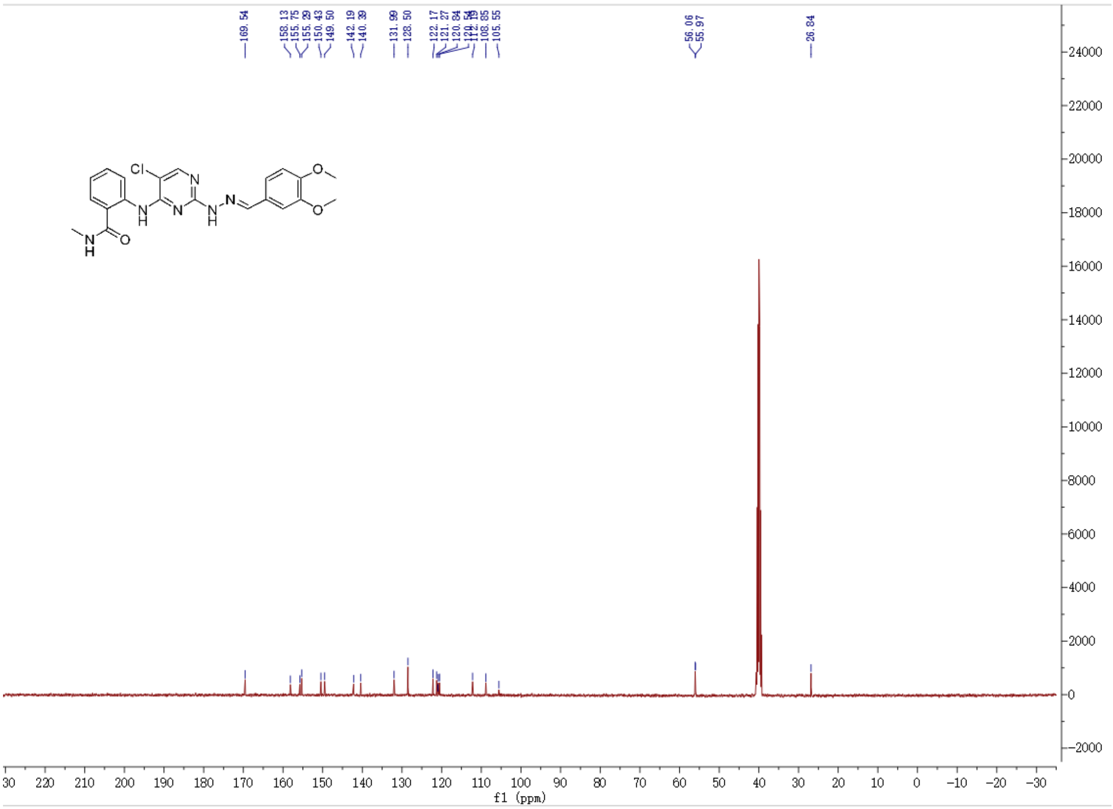

Figure S44. ^{13}C NMR spectrum of compound **14o** (100 MHz, DMSO-*d6*)

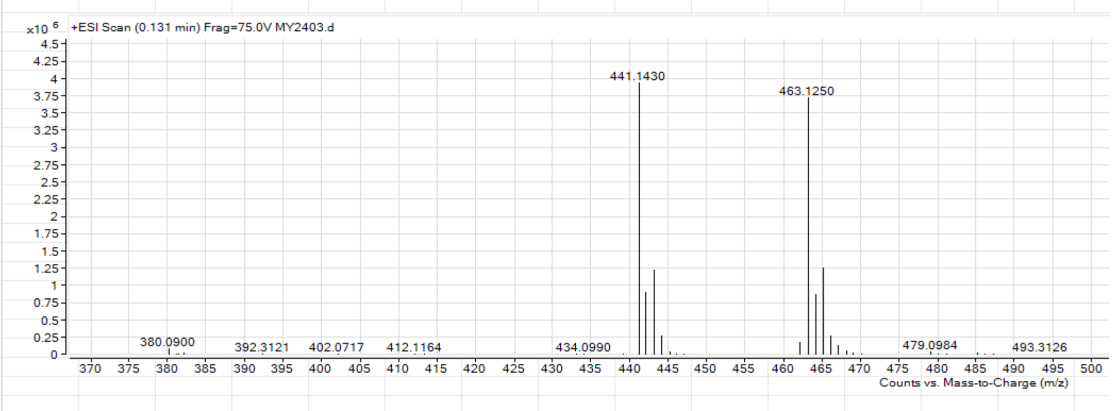

Figure S45. HRMS spectrum of compound **14o**

- ^{1}H, ^{13}C-NMR and HRMS of compound **14p**

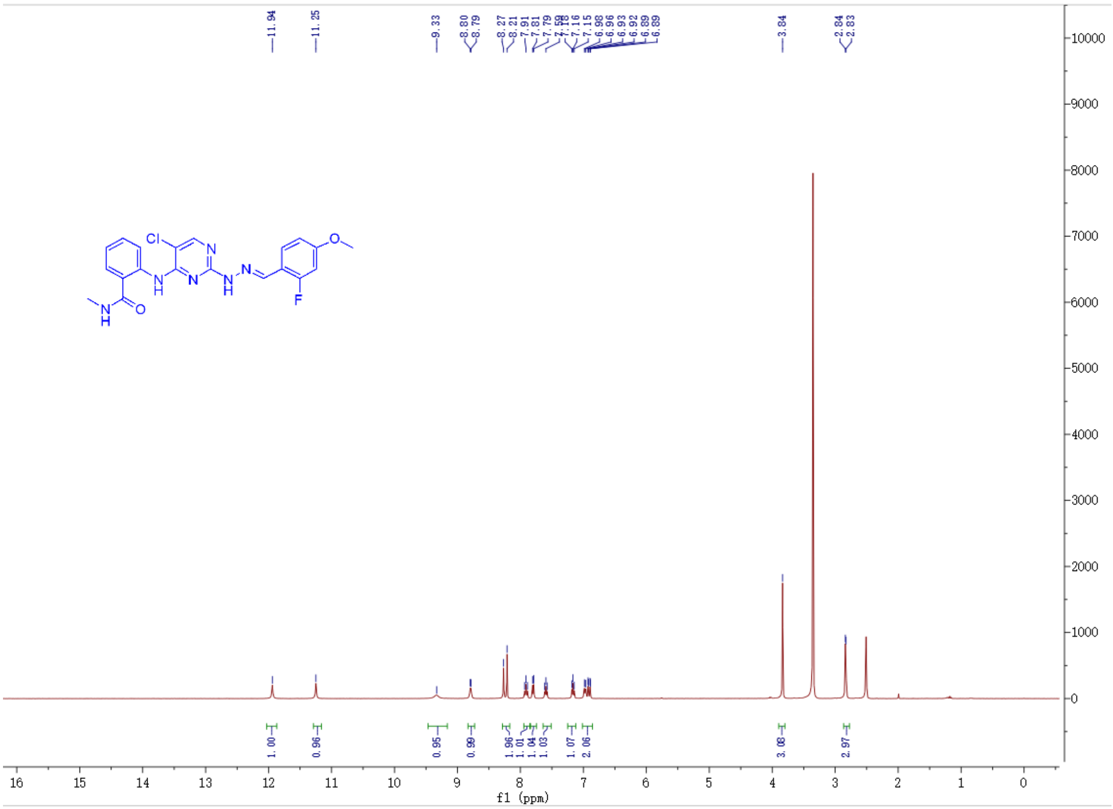

Figure S46. ^{1}H NMR spectrum of compound **14p** (400 MHz, DMSO-*d6*)

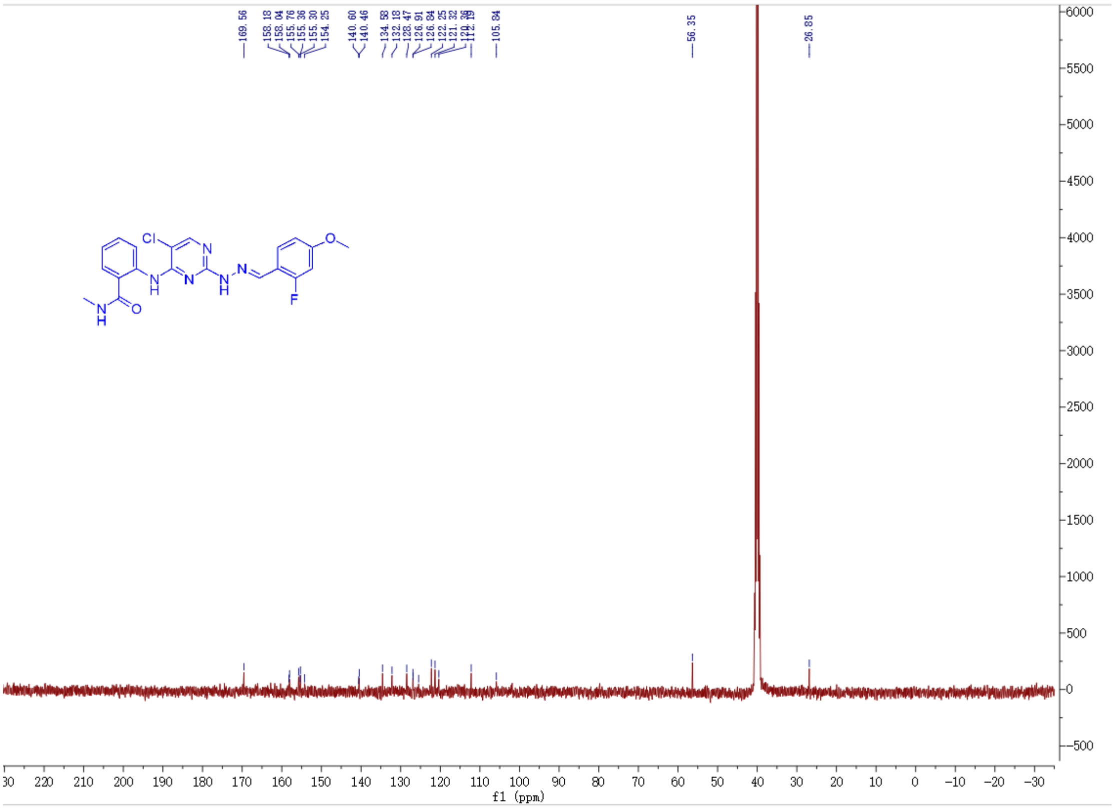

Figure S47. ^{13}C NMR spectrum of compound **14p** (100 MHz, DMSO-*d6*)

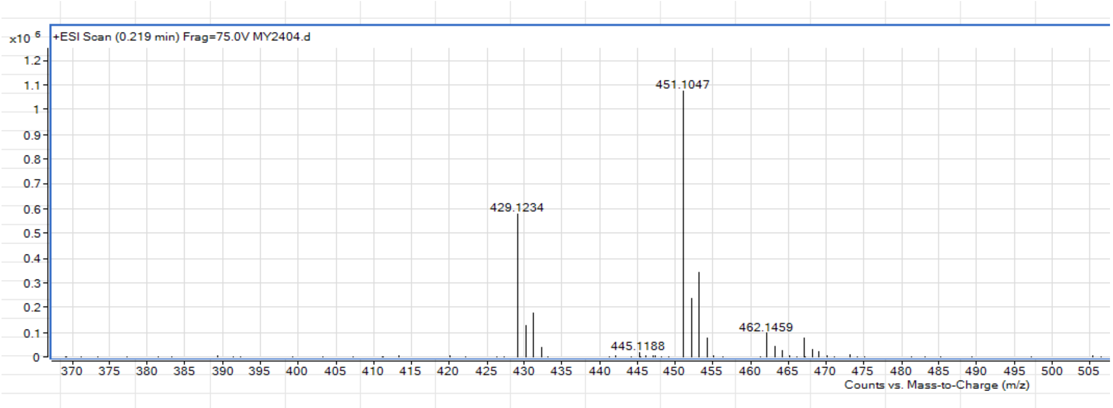

Figure S48. HRMS spectrum of compound **14p**

- ^{1}H, ^{13}C-NMR and HRMS of compound **14q**

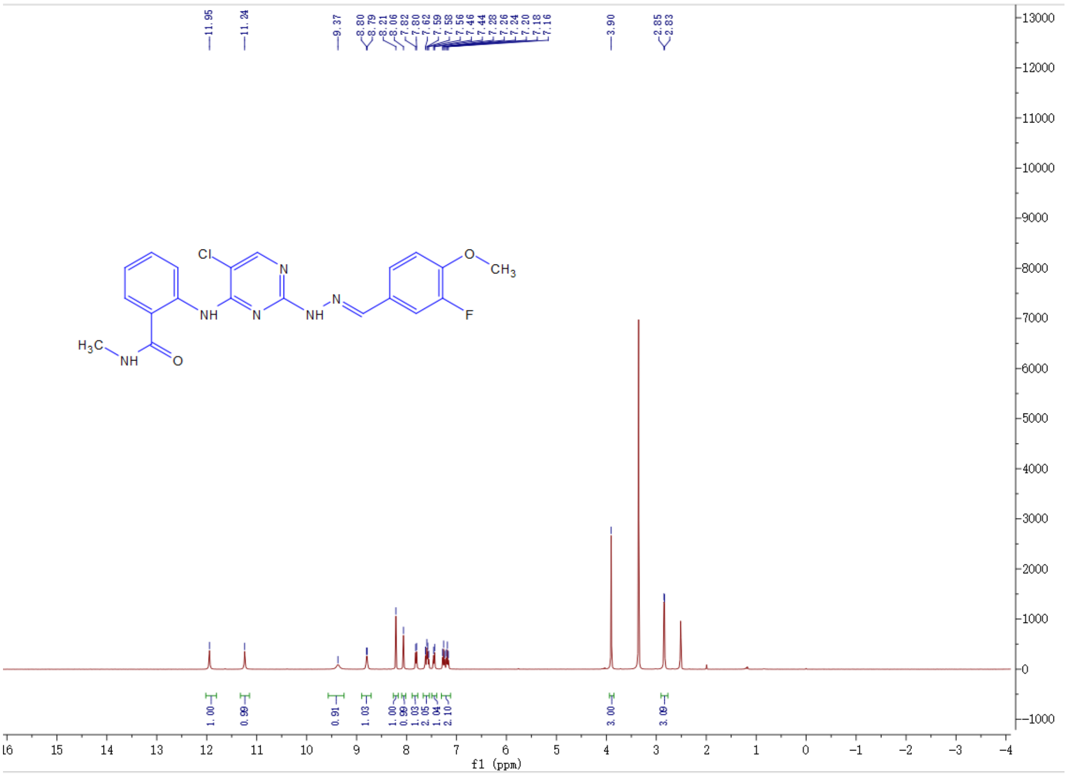

Figure S49. ^{1}H NMR spectrum of compound **14q** (400 MHz, DMSO-*d6*)

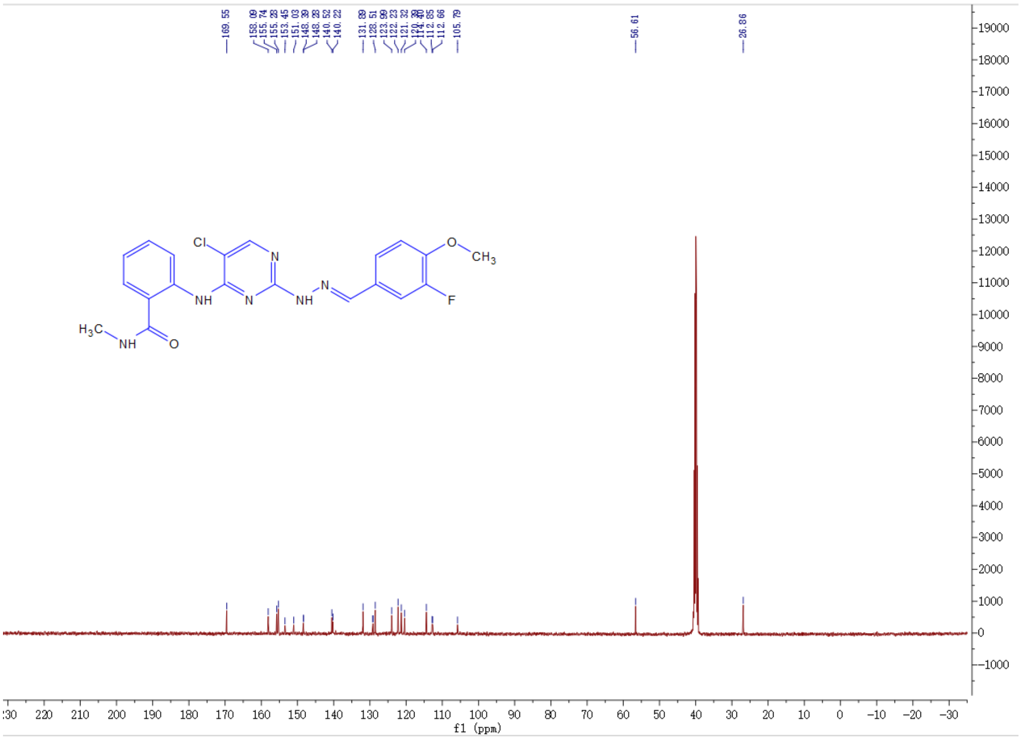

Figure S50. ^{13}C NMR spectrum of compound **14q** (100 MHz, DMSO-*d6*)

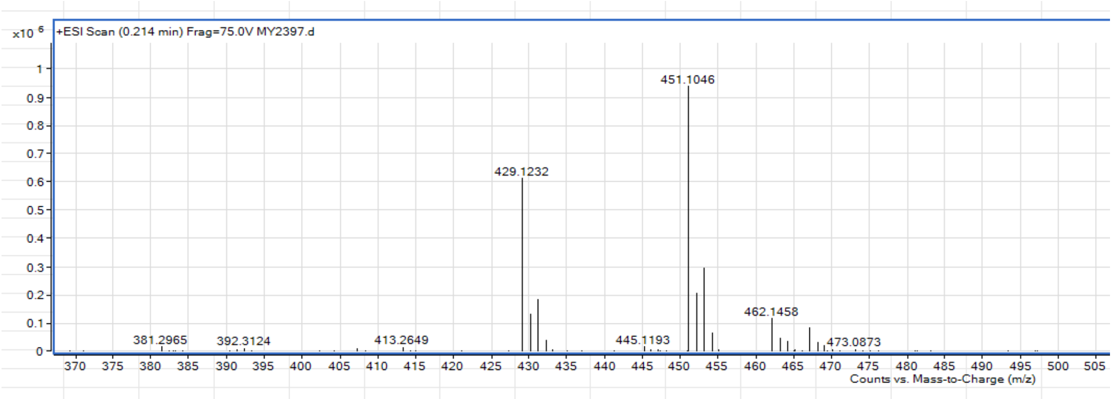

Figure S51. HRMS spectrum of compound **14q**

- ^{1}H, ^{13}C-NMR and HRMS of compound **14r**

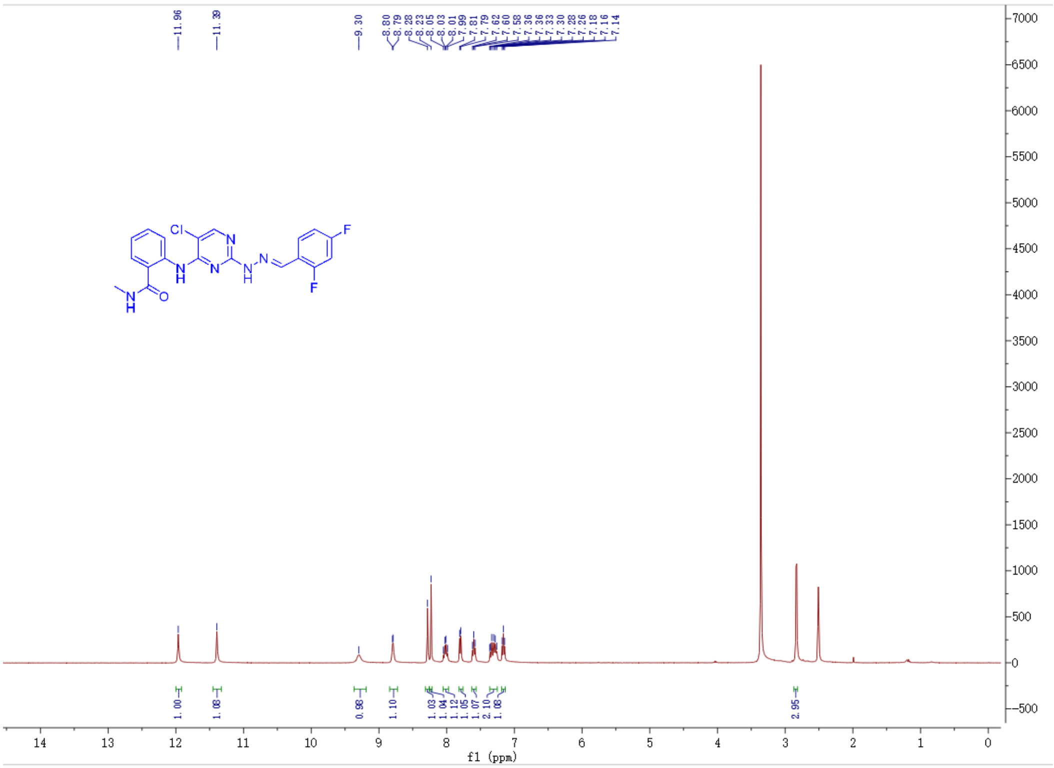

Figure S52. ^{1}H NMR spectrum of compound **14r** (400 MHz, DMSO-*d6*)

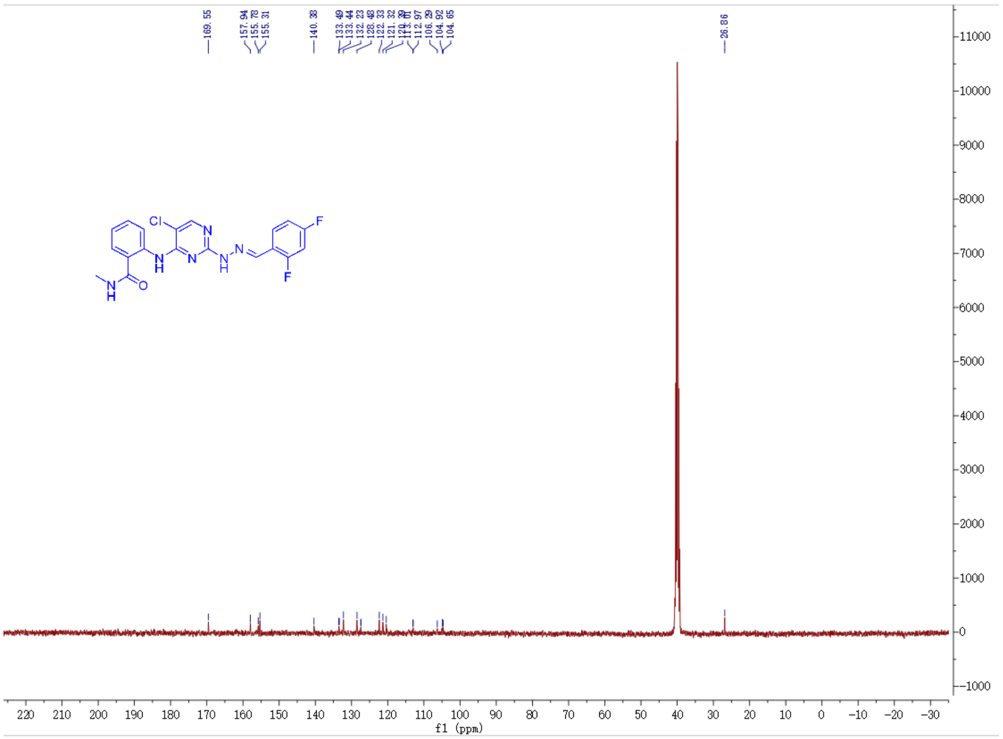

Figure S53. ^{13}C NMR spectrum of compound **14r** (100 MHz, DMSO-*d6*)

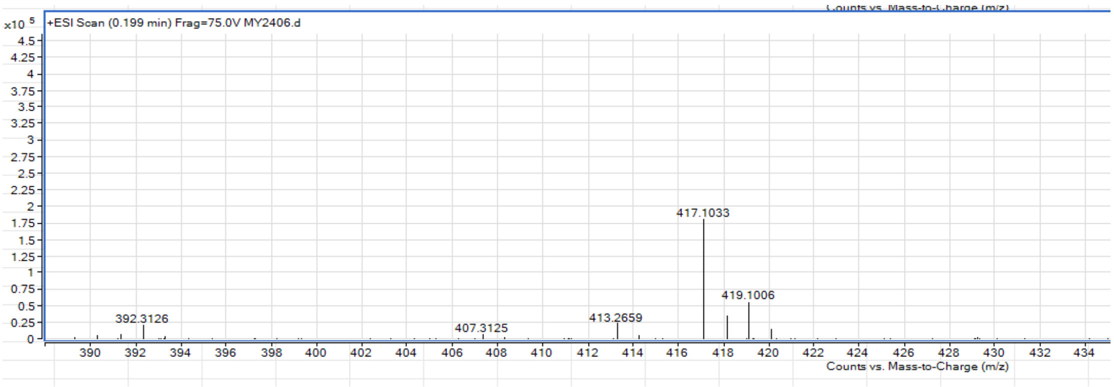

Figure S54. HRMS spectrum of compound **14r**

- ^{1}H, ^{13}C-NMR and HRMS of compound **14s**

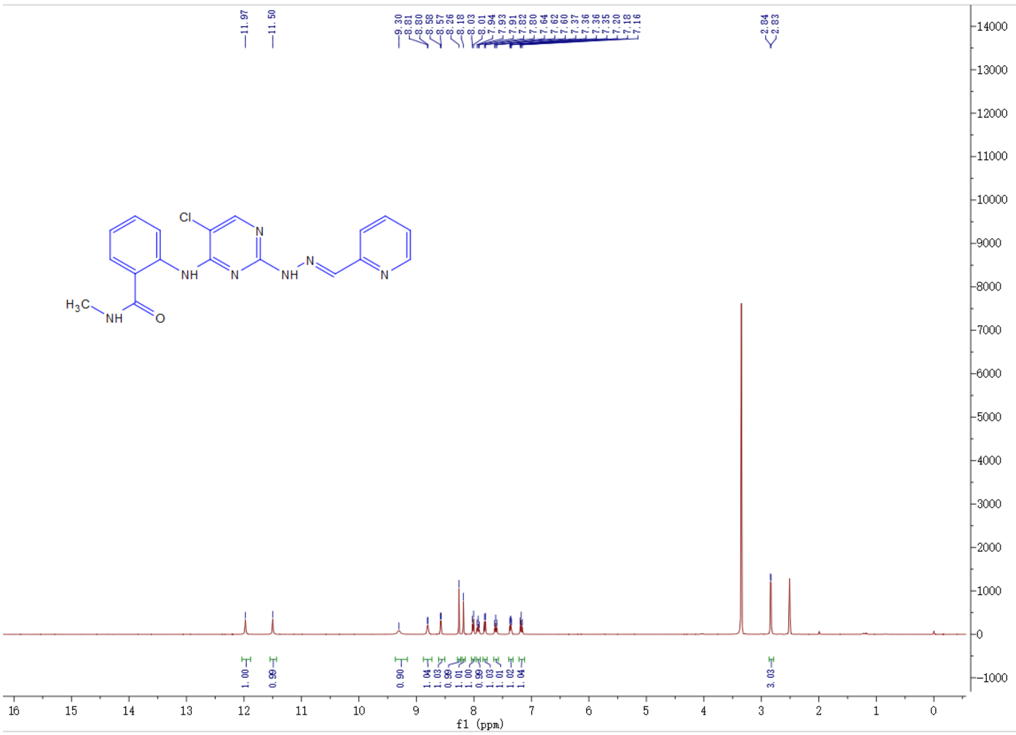

Figure S55. ^{1}H NMR spectrum of compound **14s** (400 MHz, DMSO-*d6*)

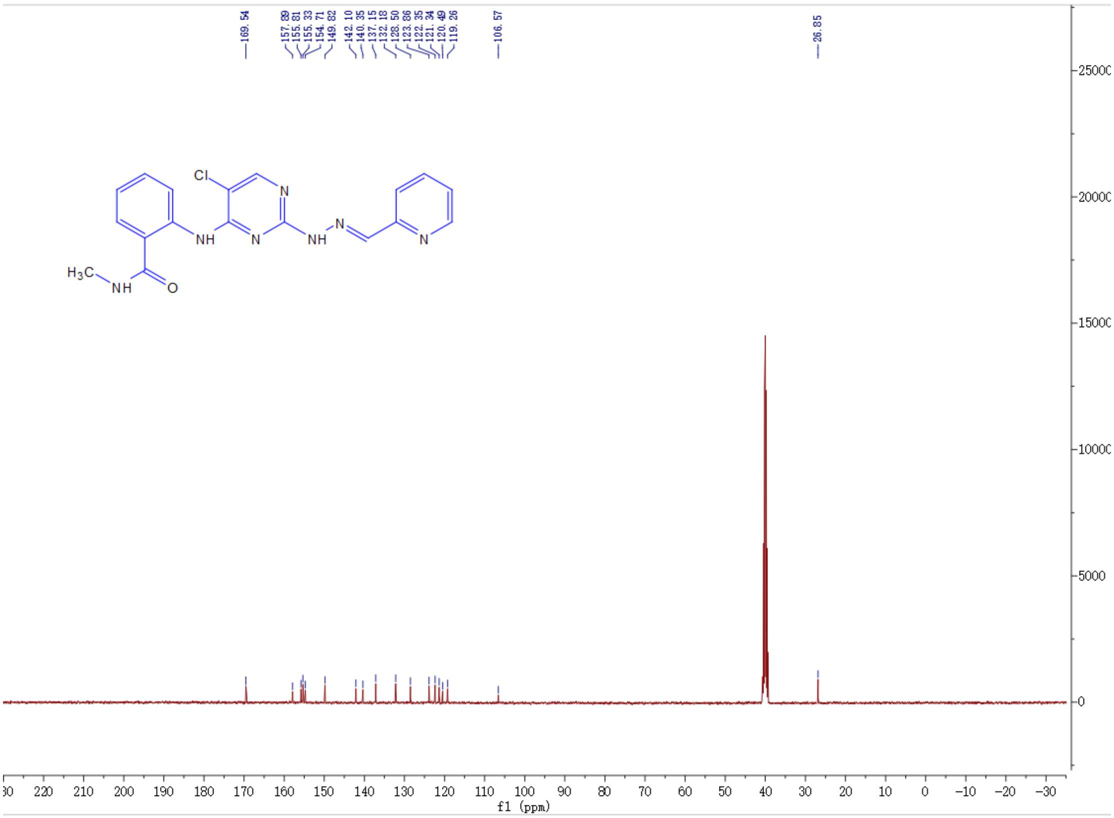

Figure S56. ^{13}C NMR spectrum of compound **14s** (100 MHz, DMSO-*d6*)

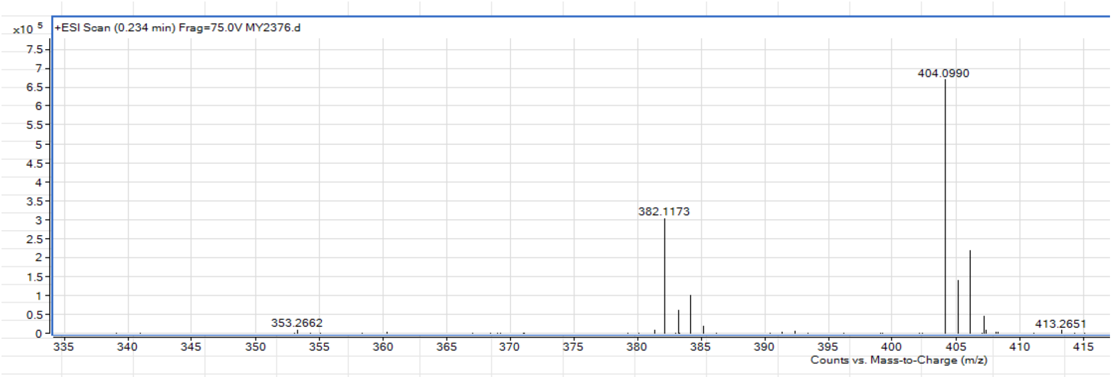

Figure S57. HRMS spectrum of compound **14s**

- ^{1}H, ^{13}C-NMR and HRMS of compound **14t**

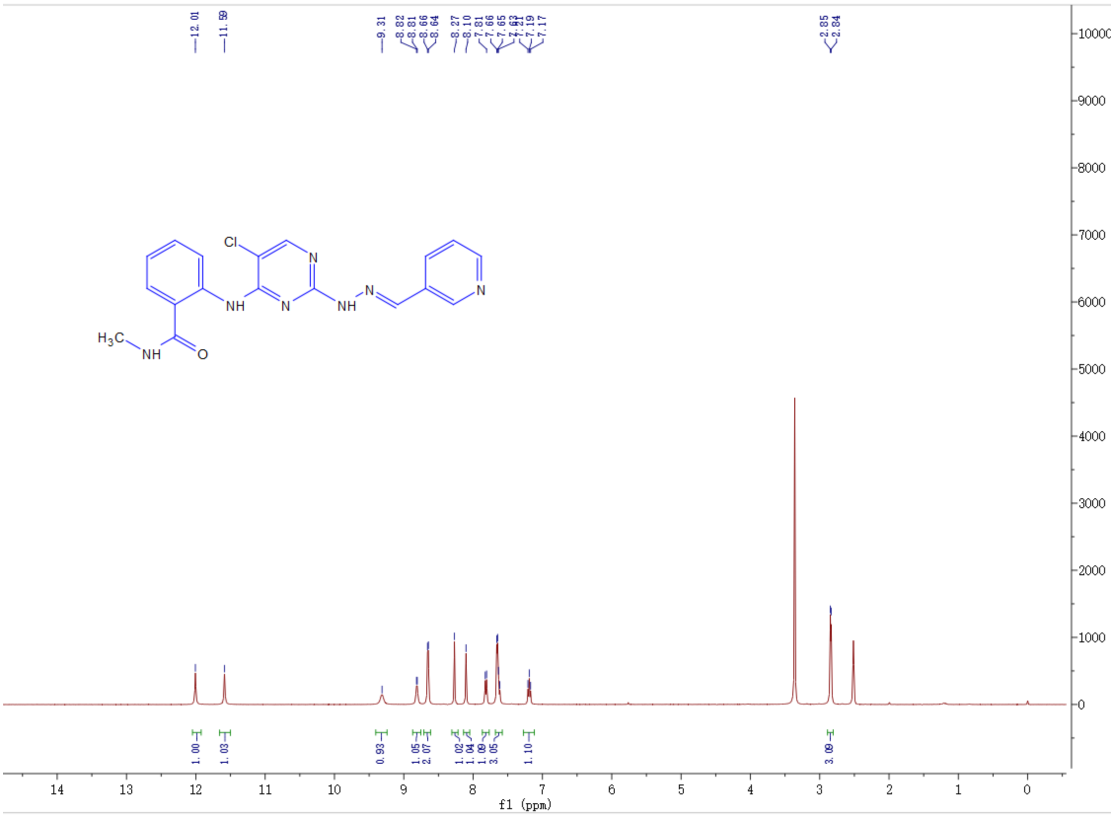

Figure S58. ^{1}H NMR spectrum of compound **14t** (400 MHz, DMSO-*d6*)

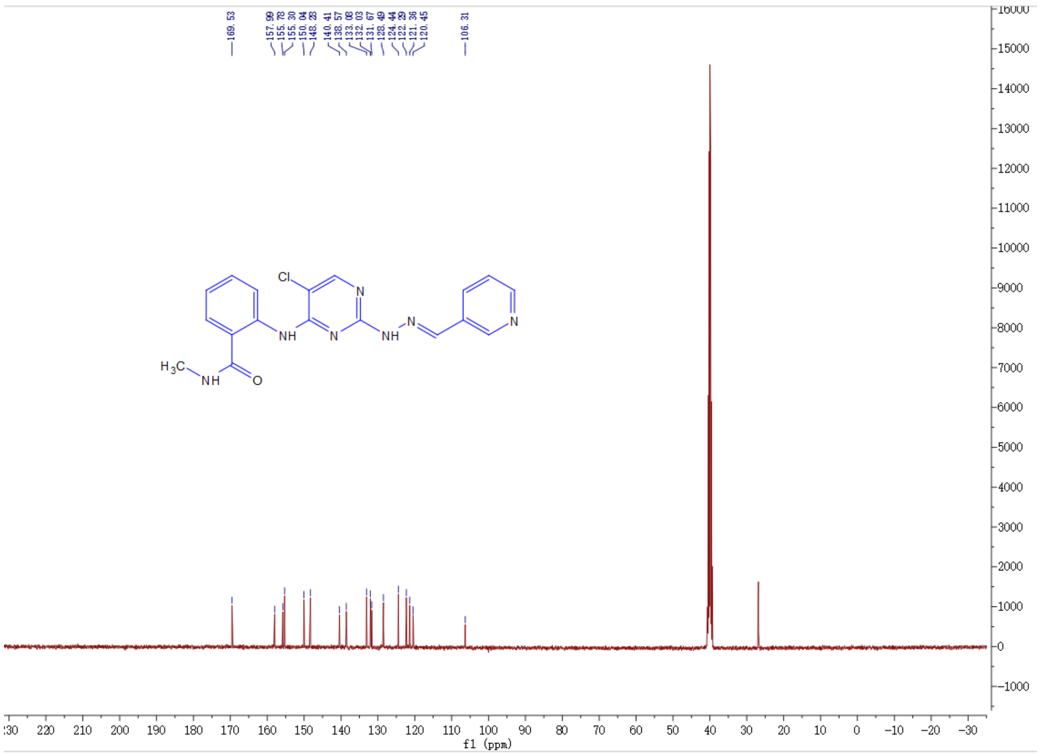

Figure S59. ^{13}C NMR spectrum of compound **14t** (100 MHz, DMSO-*d6*)

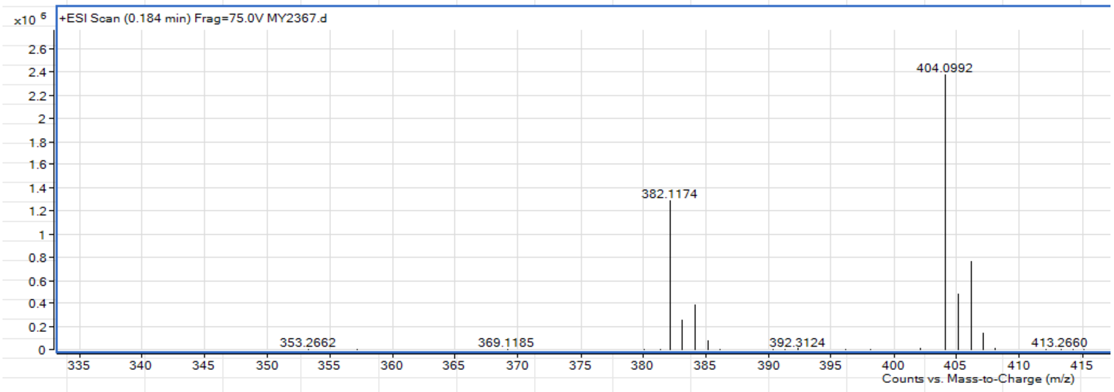

Figure S60. HRMS spectrum of compound **14t**

- ^{1}H and ^{13}C-NMR of compound **14u**

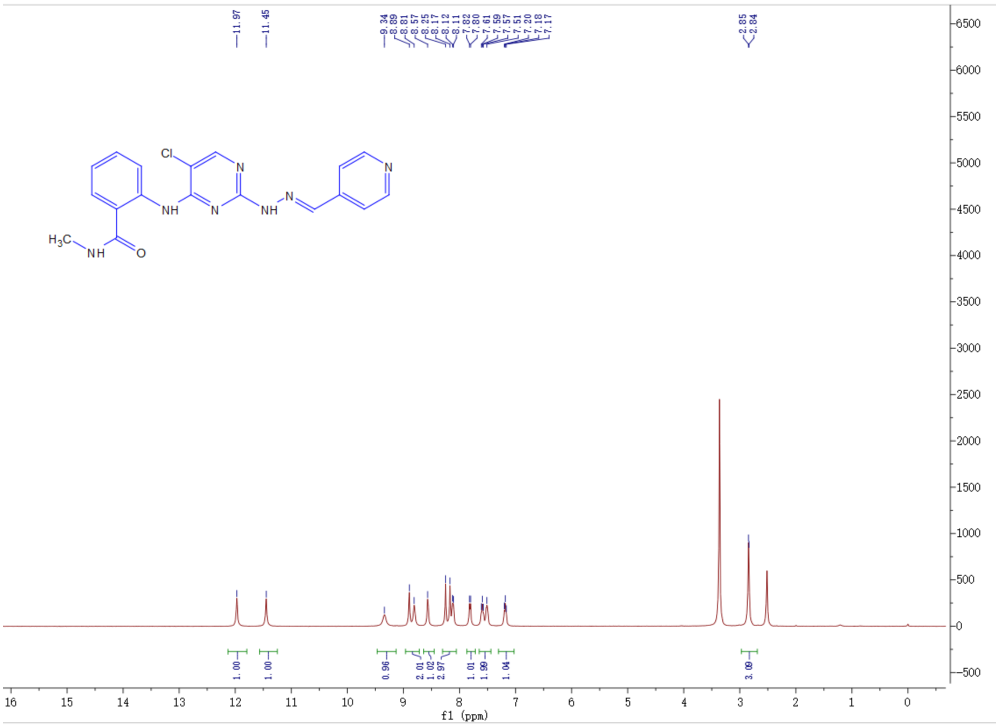

Figure S61. ^{1}H NMR spectrum of compound **14u** (400 MHz, DMSO-*d6*)

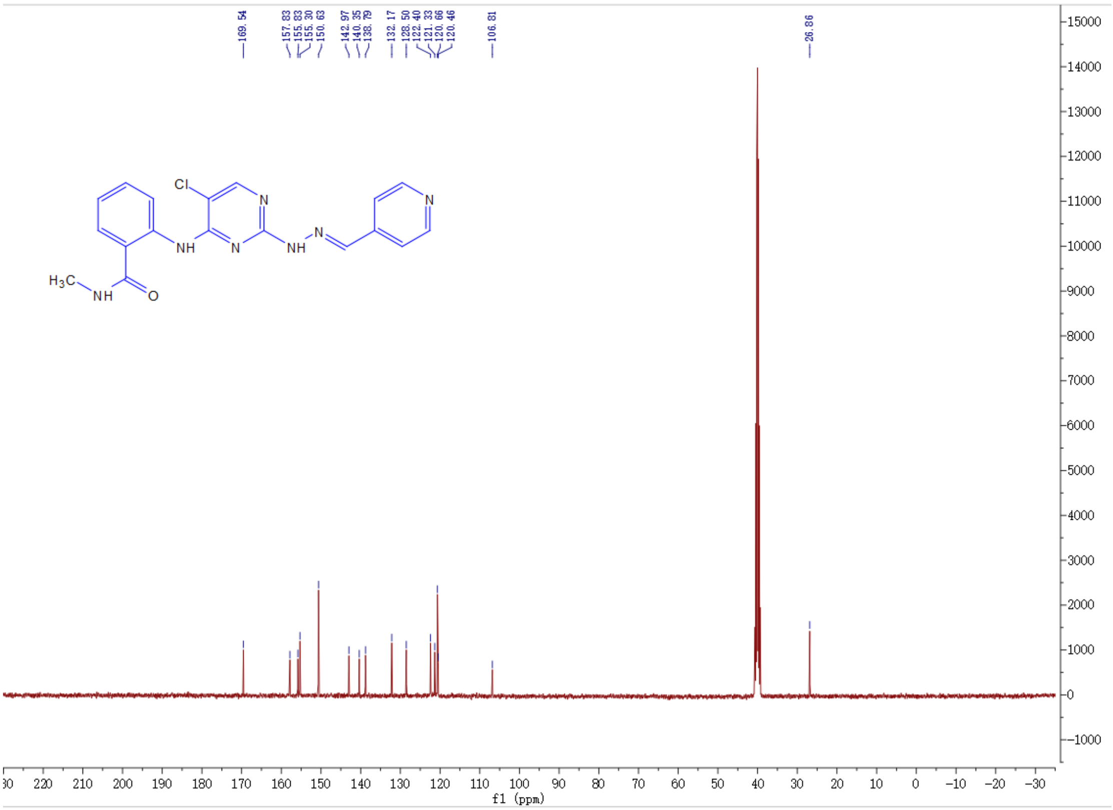

Figure S62. ^{13}C NMR spectrum of compound **14u** (100 MHz, DMSO-*d6*)

● ^{1}H, ^{13}C-NMR and HRMS of compound **14v**

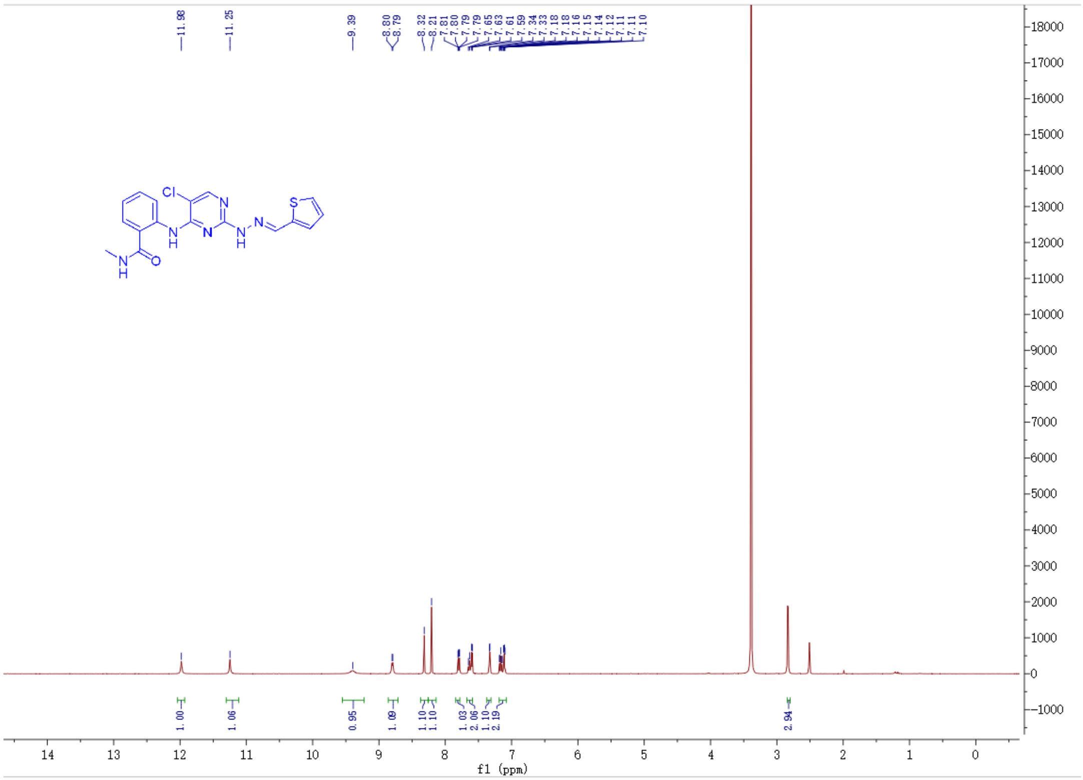

Figure S63. ^{1}H NMR spectrum of compound **14v** (400 MHz, DMSO-*d6*)

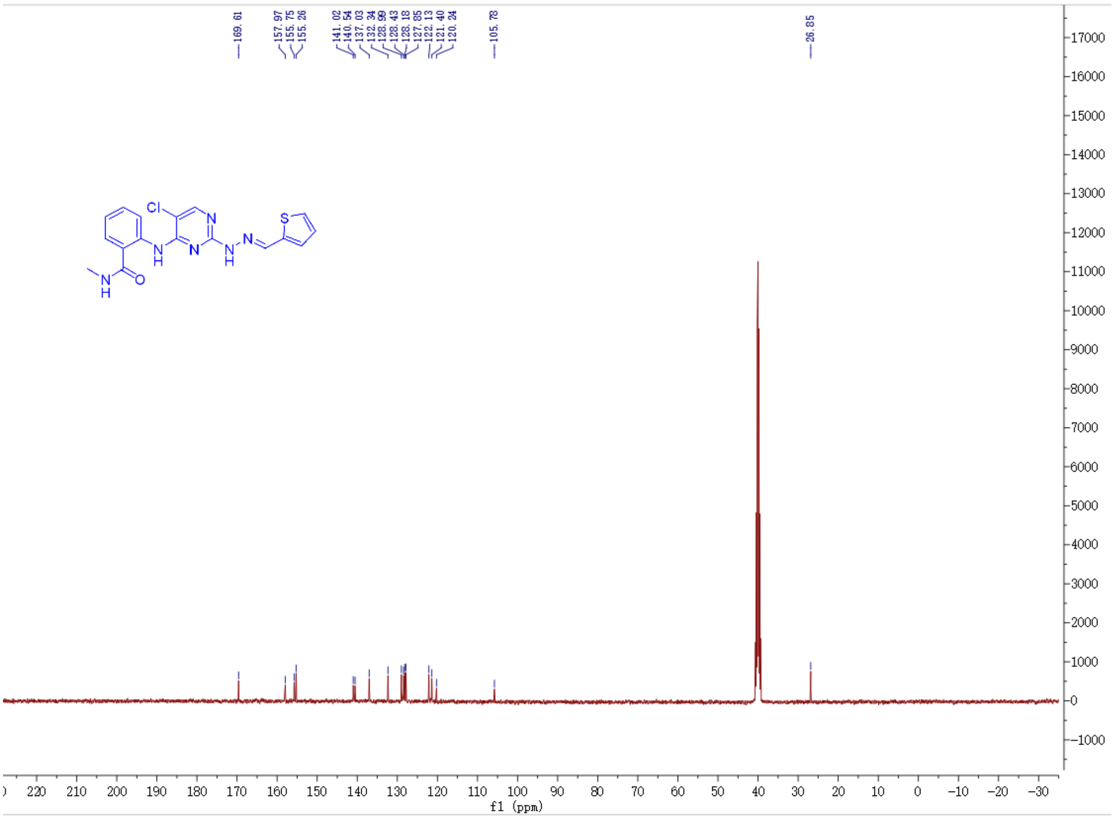

Figure S64. ^{13}C NMR spectrum of compound **14v** (100 MHz, DMSO-*d6*)

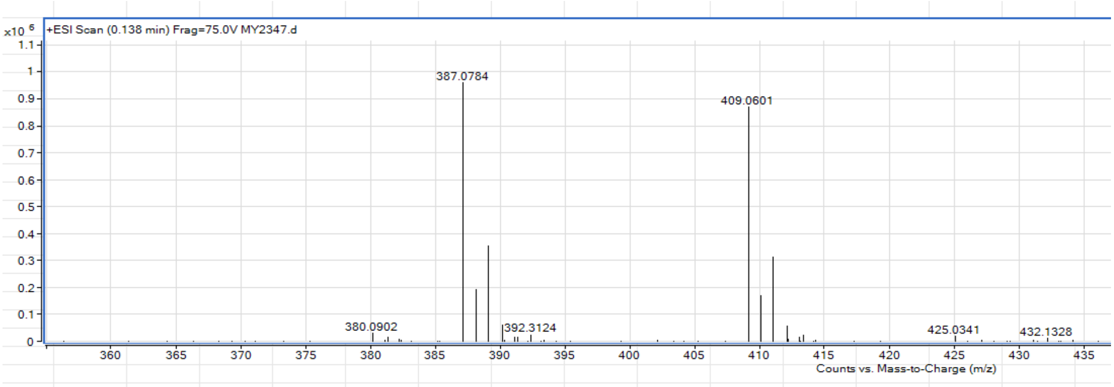

Figure S65. HRMS spectrum of compound **14v**

- ^{1}H, ^{13}C-NMR and HRMS of compound **14w**

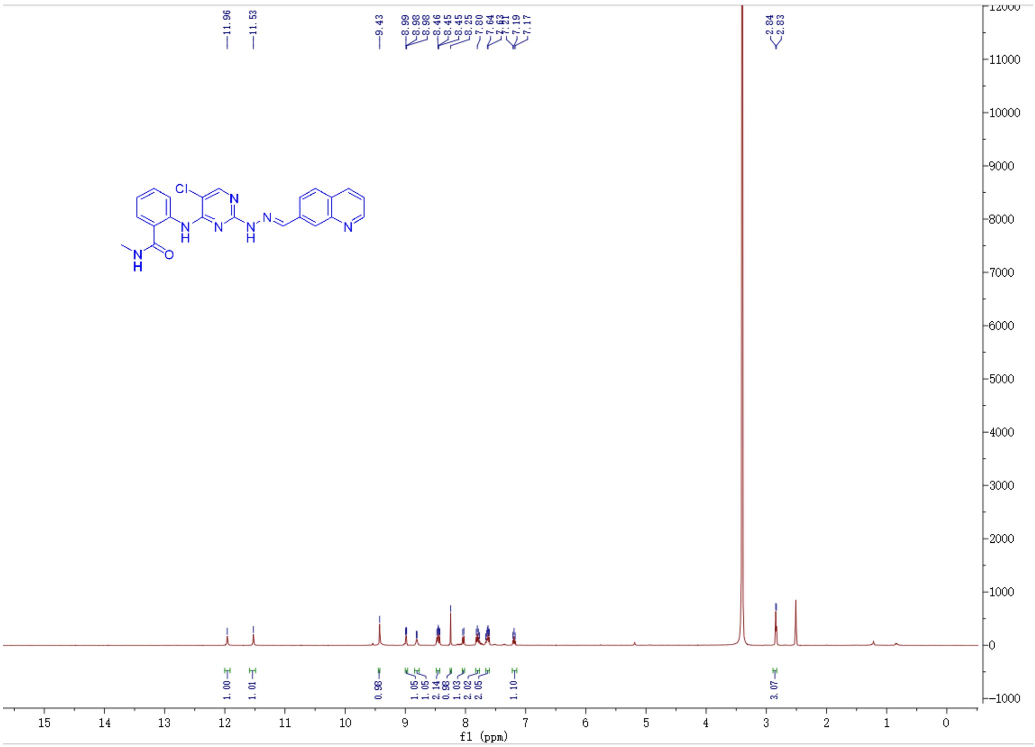

Figure S66. ^{1}H NMR spectrum of compound **14w** (400 MHz, DMSO-*d6*)

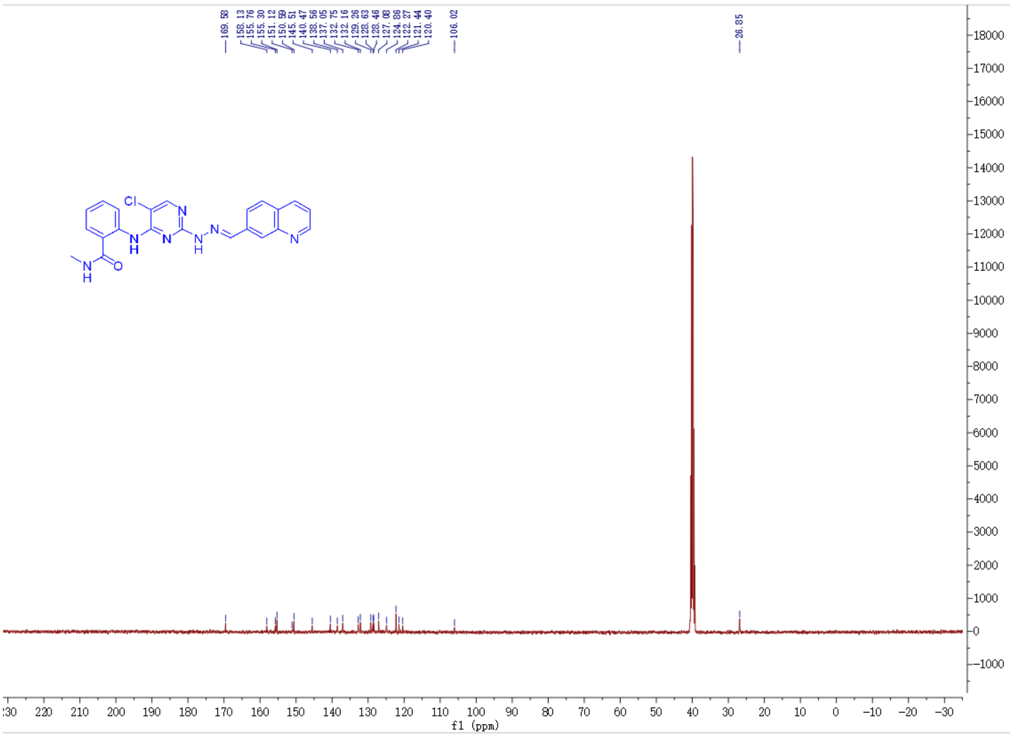

Figure S67. ^{13}C NMR spectrum of compound **14w** (100 MHz, DMSO-*d6*)

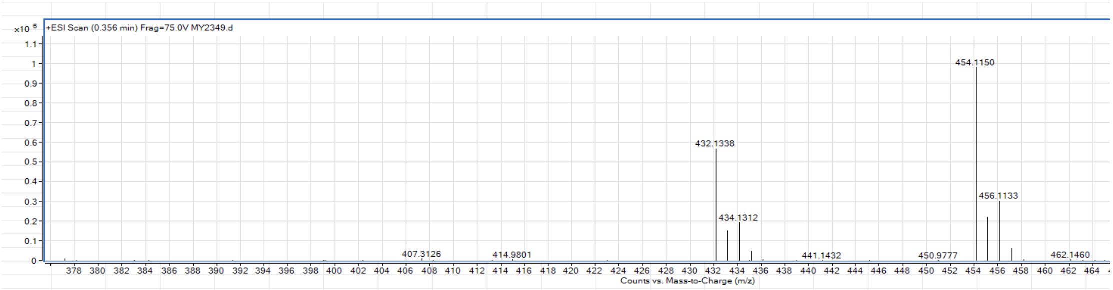

Figure S68. HRMS spectrum of compound **14w**

● ^{1}H, ^{13}C-NMR and HRMS of compound **14x**

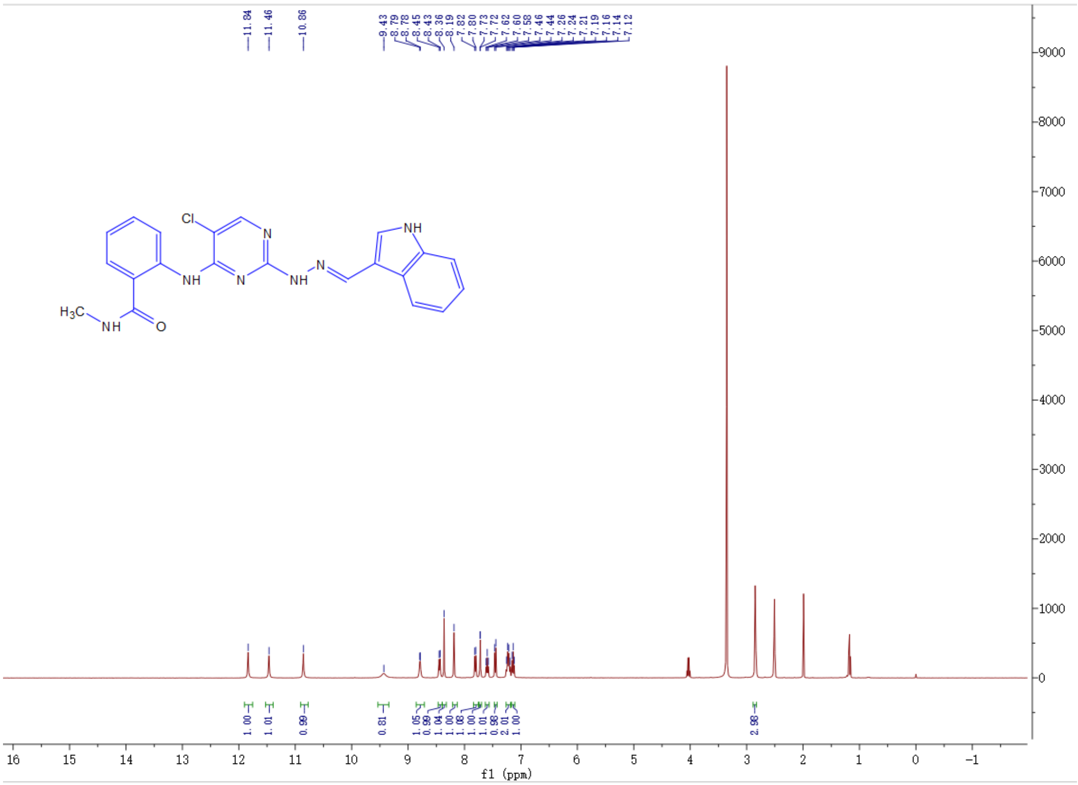

Figure S69. ^{1}H NMR spectrum of compound **14x** (400 MHz, DMSO-*d6*)

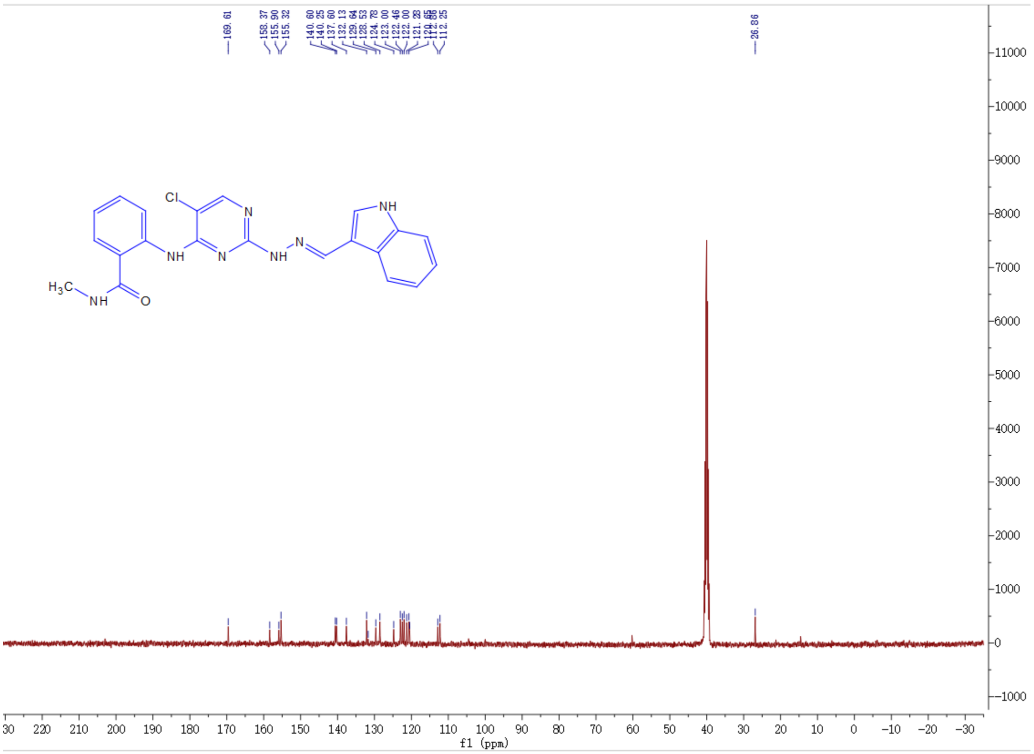

Figure S70. ^{13}C NMR spectrum of compound **14x** (100 MHz, DMSO-*d6*)

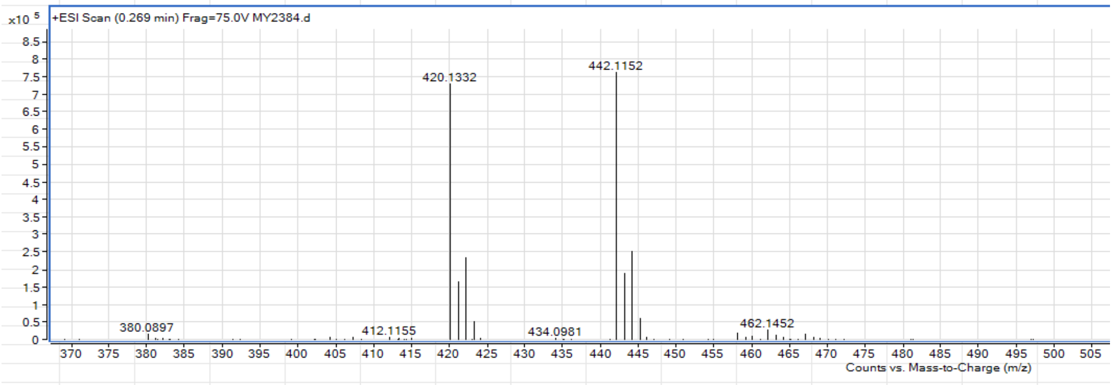

Figure S71. HRMS spectrum of compound **14x**

- ^{1}H, ^{13}C-NMR and HRMS of compound **14y**

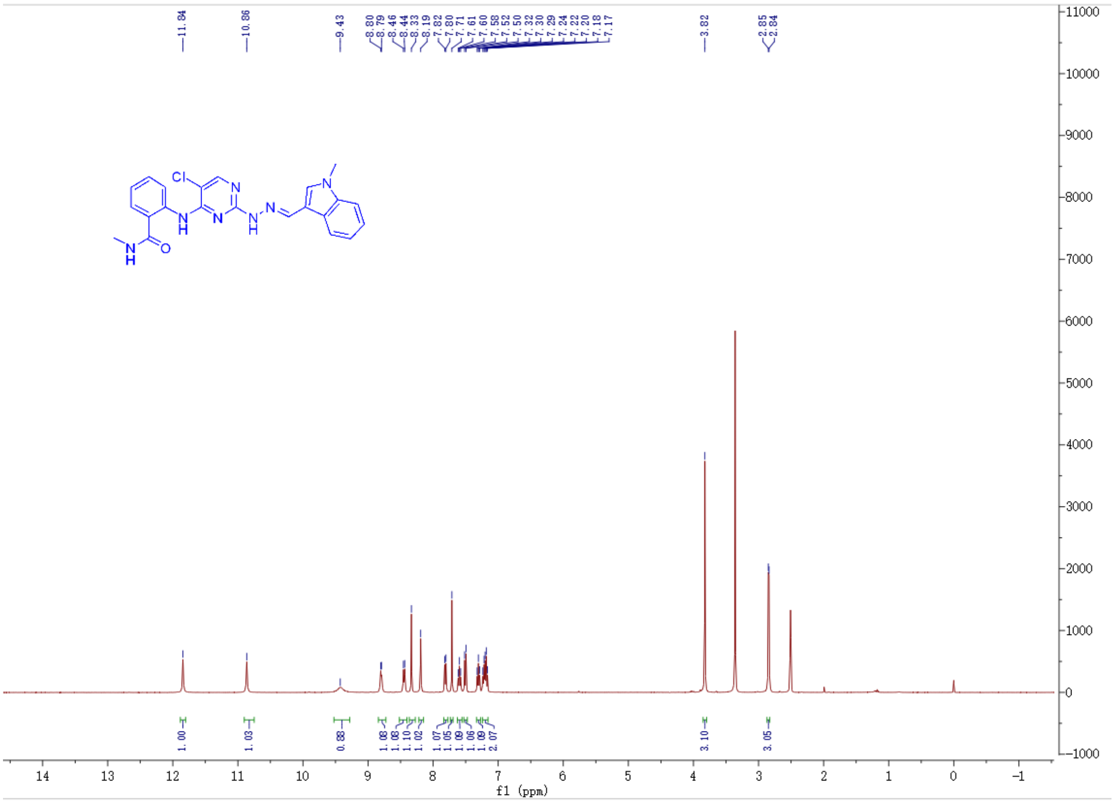

Figure S72. ^{1}H NMR spectrum of compound **14y** (400 MHz, DMSO-*d6*)

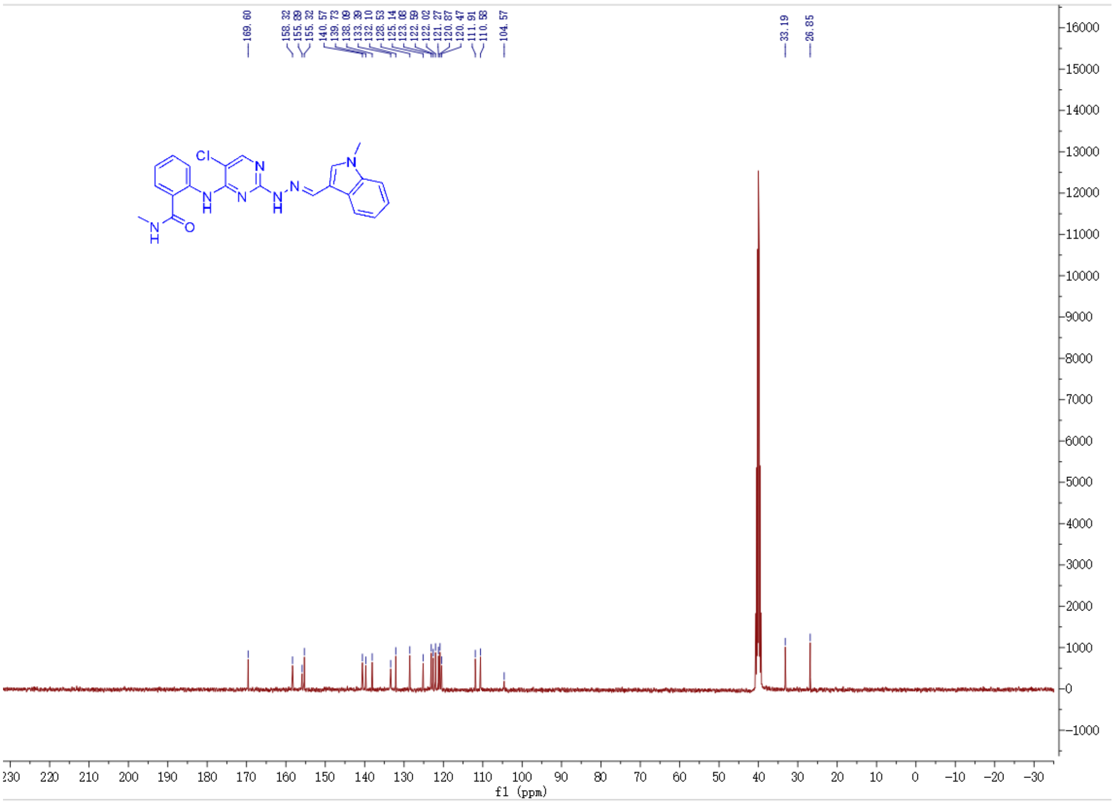

Figure S73. ¹³C NMR spectrum of compound **14y** (100 MHz, DMSO-*d6*)

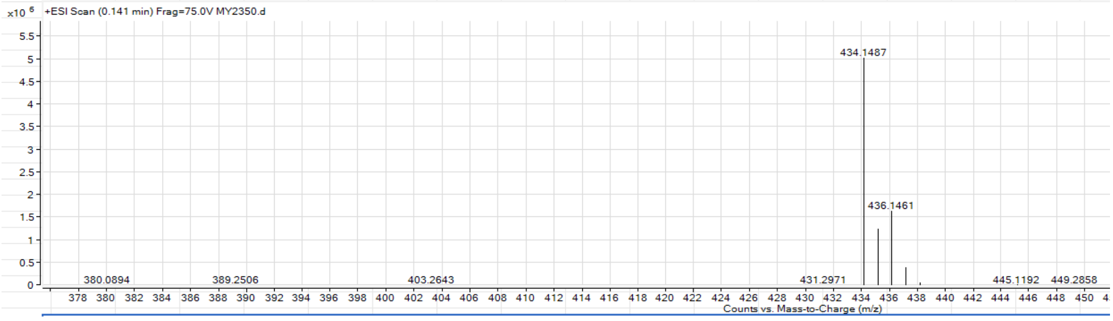

Figure S74. HRMS spectrum of compound **14y**

- ^{1}H, ^{13}C-NMR and HRMS of compound **14z**

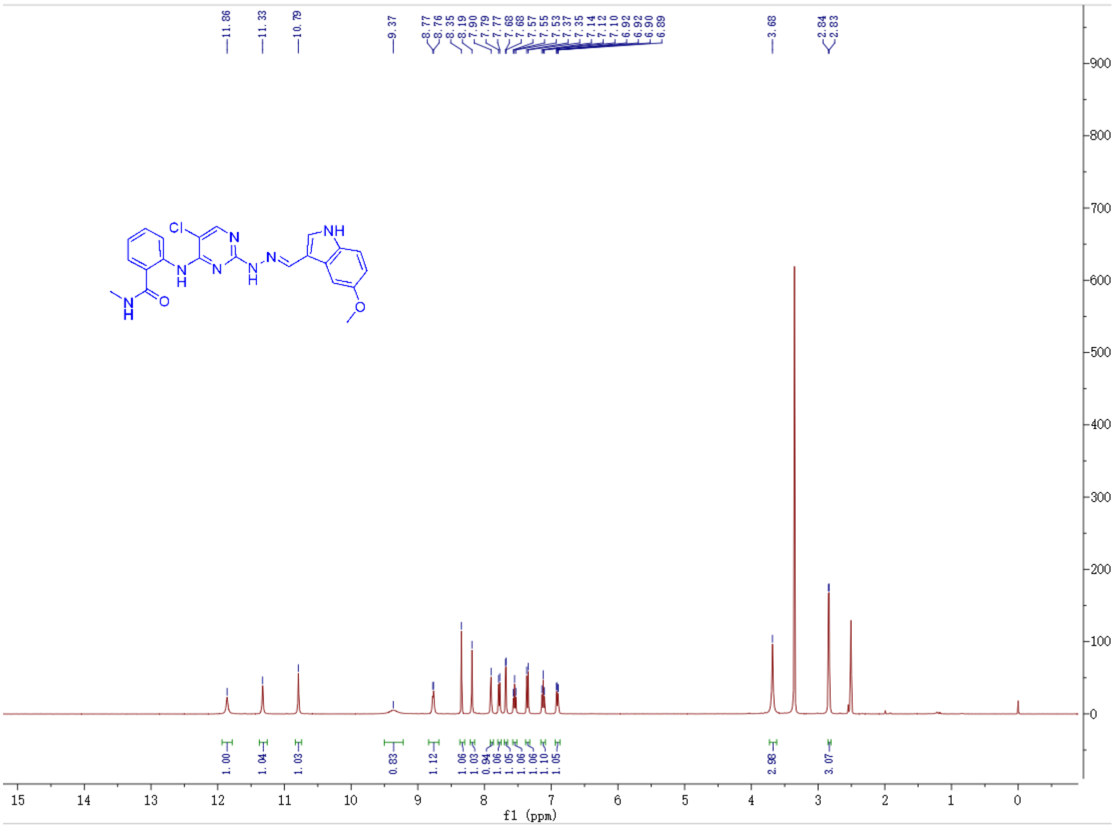

Figure S75. ^{1}H NMR spectrum of compound **14z** (400 MHz, DMSO-*d6*)

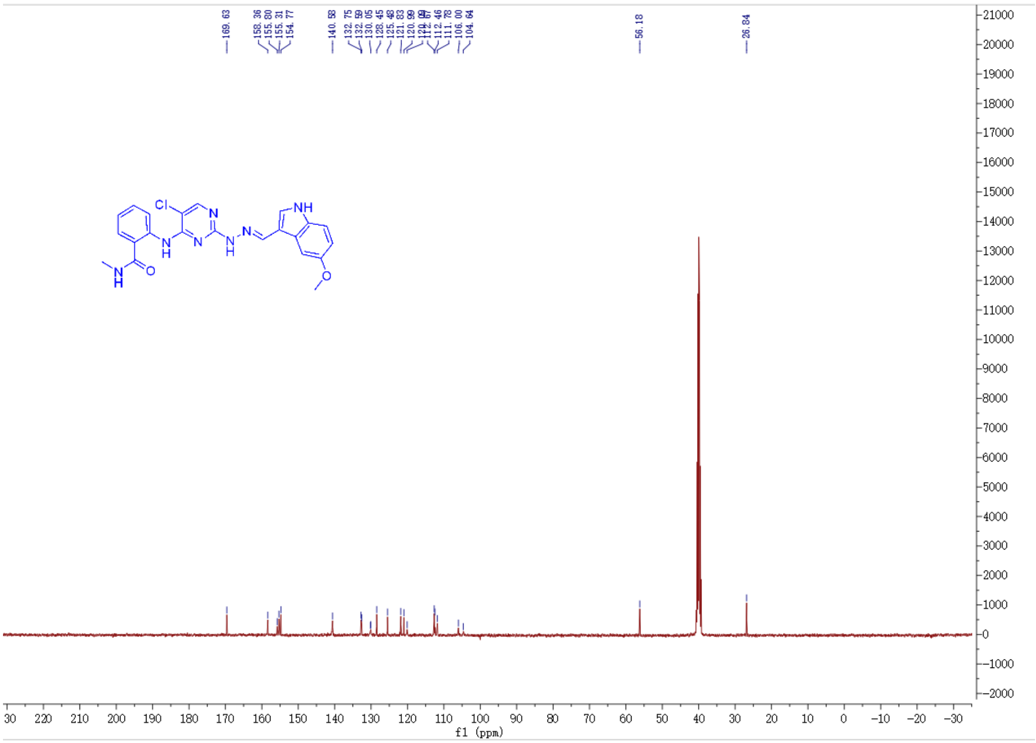

Figure S76. ^{13}C NMR spectrum of compound **14z** (100 MHz, DMSO-*d6*)

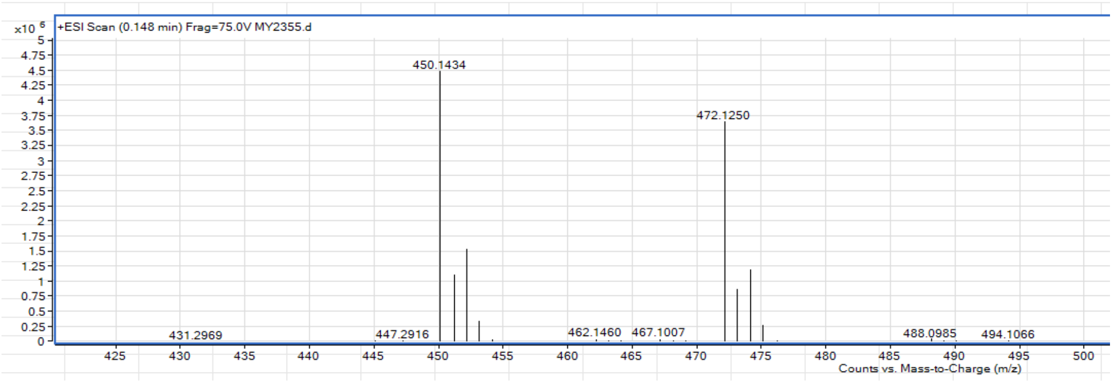

Figure S77. HRMS spectrum of compound **14z**

- ^{1}H, ^{13}C-NMR and HRMS of compound **14aa**

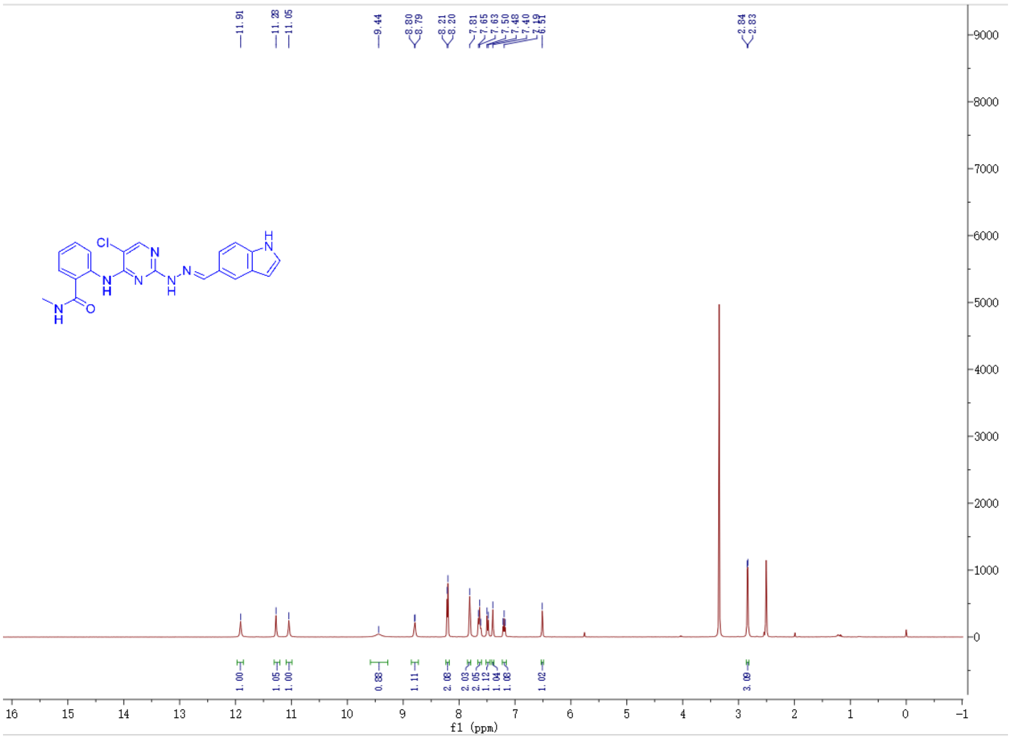

Figure S75. ^{1}H NMR spectrum of compound **14aa** (400 MHz, DMSO-*d6*)

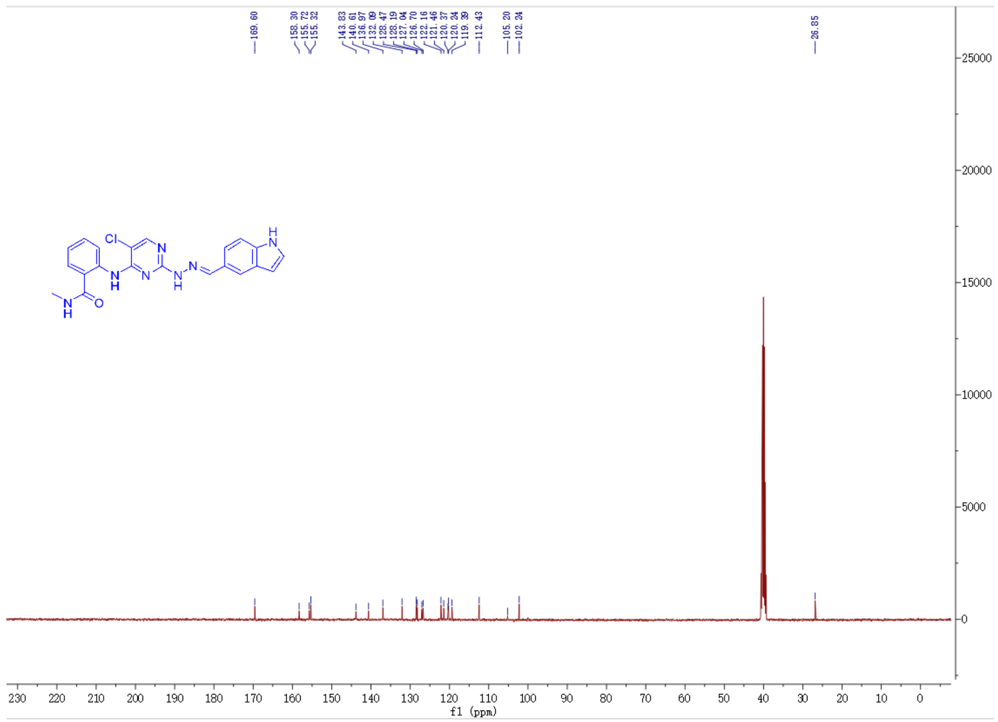

Figure S76. ^{13}C NMR spectrum of compound **14aa** (100 MHz, DMSO-*d6*)

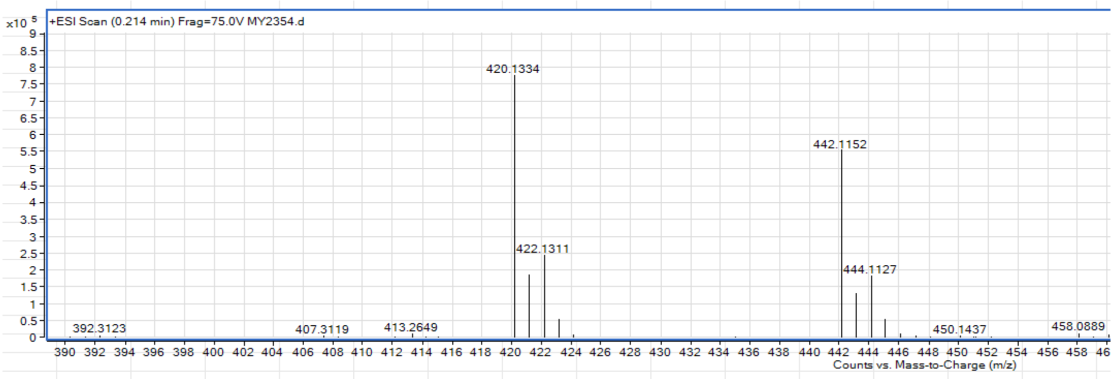

Figure S77. HRMS spectrum of compound **14aa**

● ^{1}H, ^{13}C-NMR and HRMS of compound **14ab**

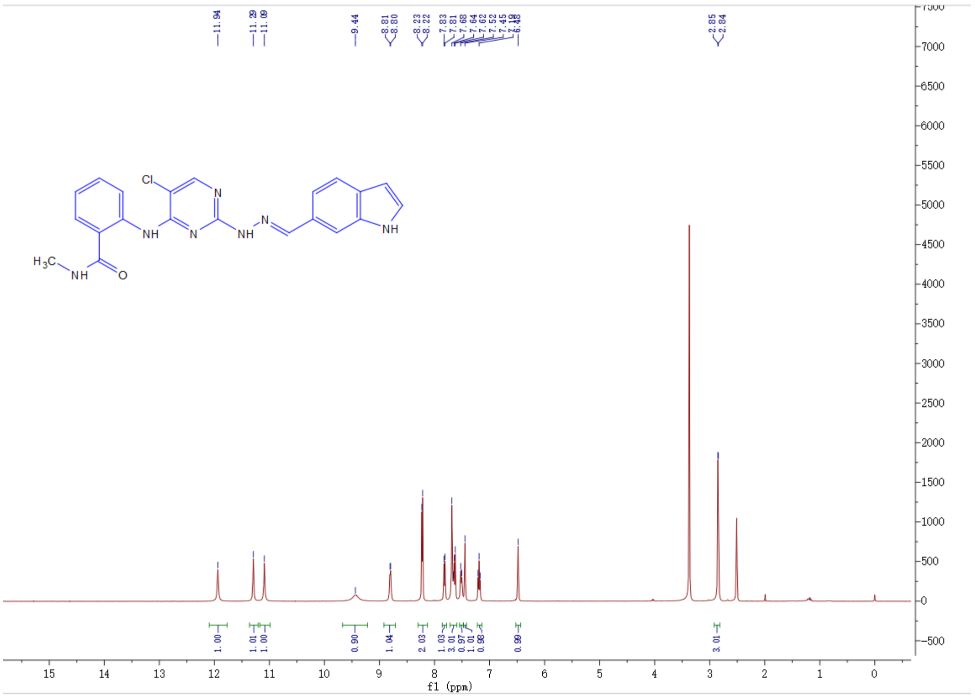

Figure S78. ^{1}H NMR spectrum of compound **14ab** (400 MHz, DMSO-*d6*)

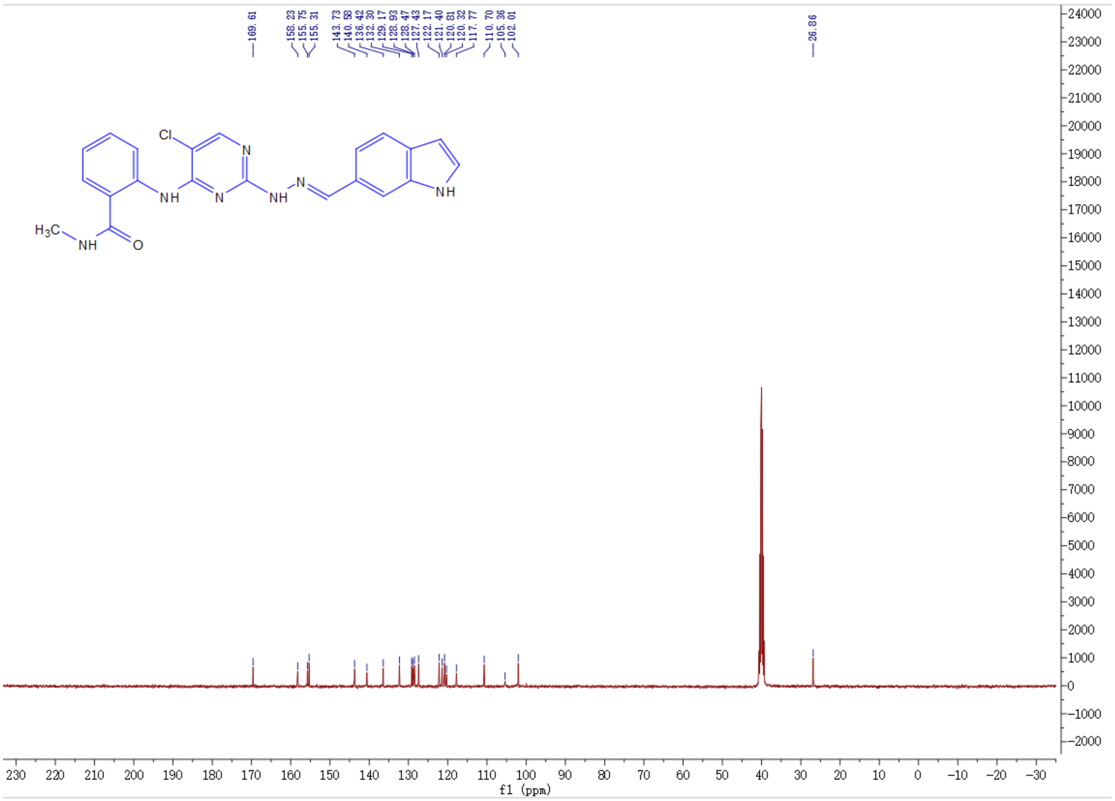

Figure S79. ^{13}C NMR spectrum of compound **14ab** (100 MHz, DMSO-*d6*)

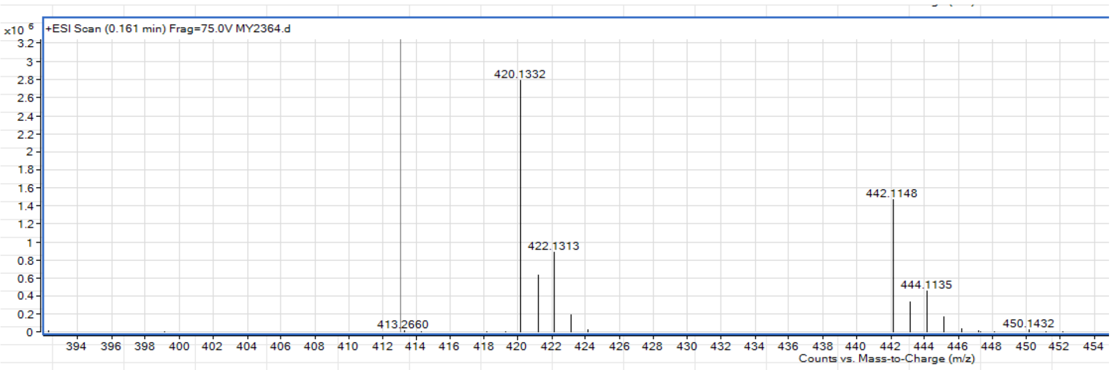

Figure S80. HRMS spectrum of compound **14ab**

- ^{1}H, ^{13}C-NMR and HRMS of compound **14ac**

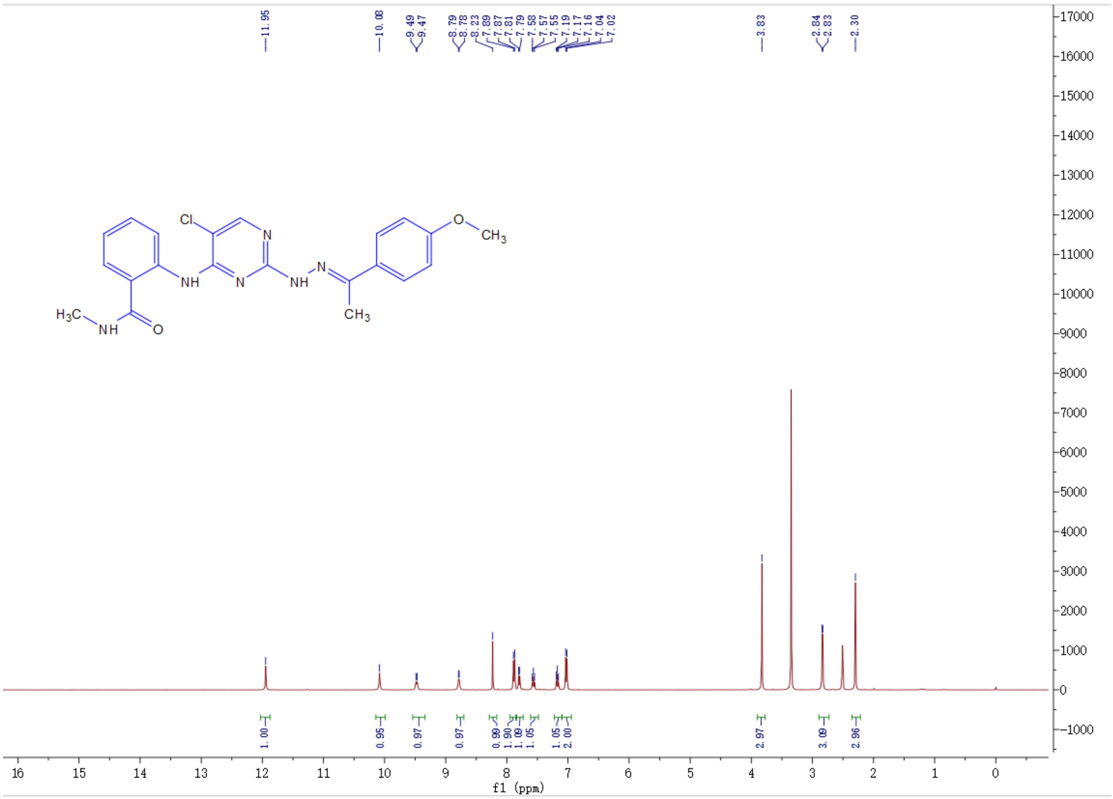

Figure S81. ^{1}H NMR spectrum of compound **14ac** (400 MHz, DMSO-*d6*)

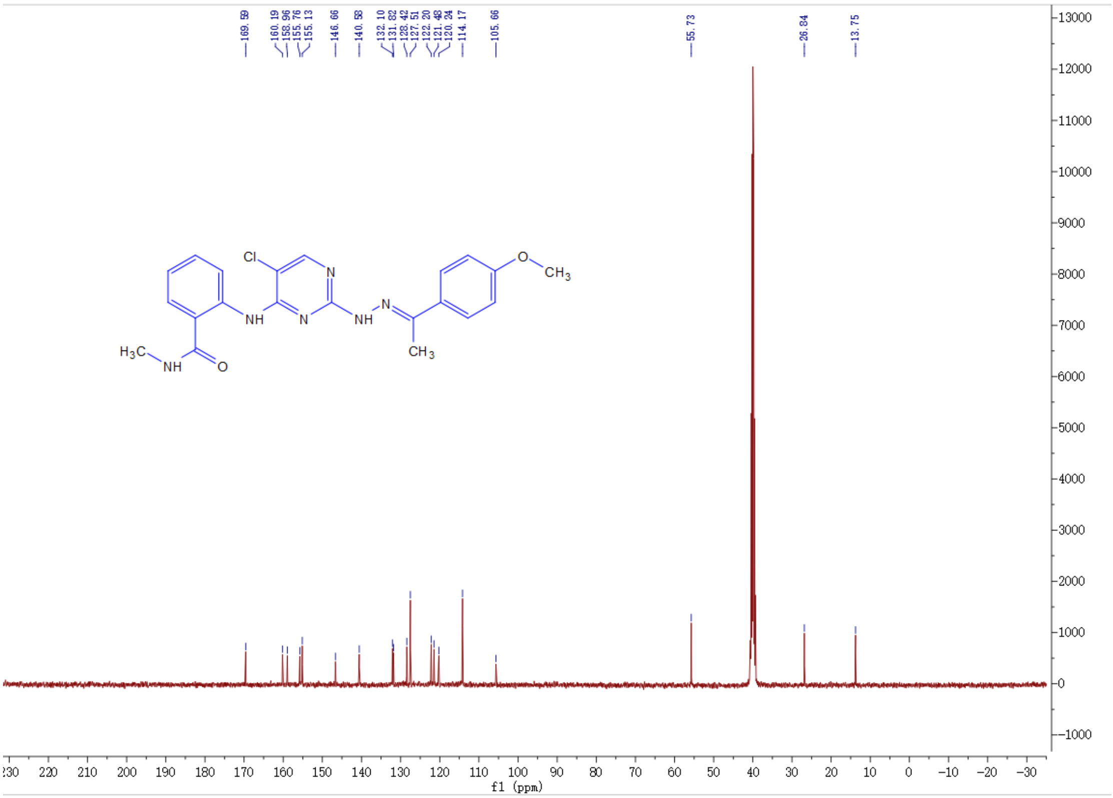

Figure S82. ^{13}C NMR spectrum of compound **14ac** (100 MHz, DMSO-*d6*)

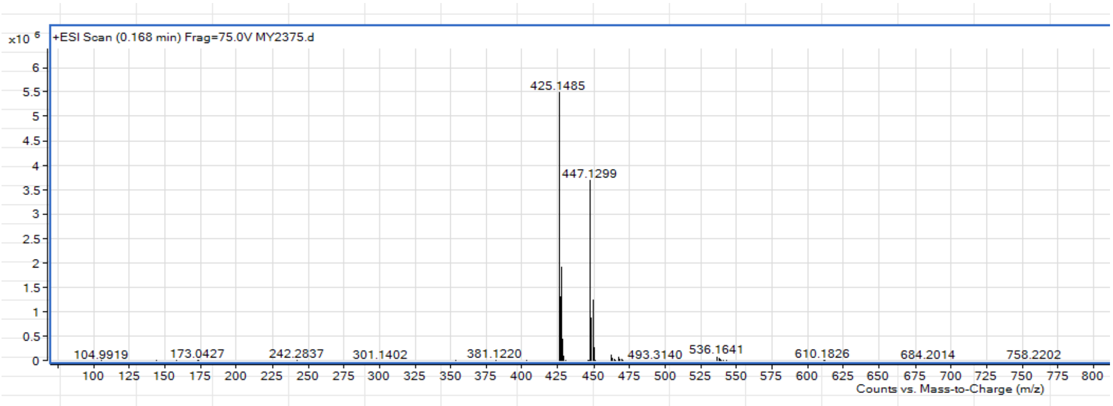

Figure S83. HRMS spectrum of compound **14ac**

● ^{1}H, ^{13}C-NMR and HRMS of compound **14ad**

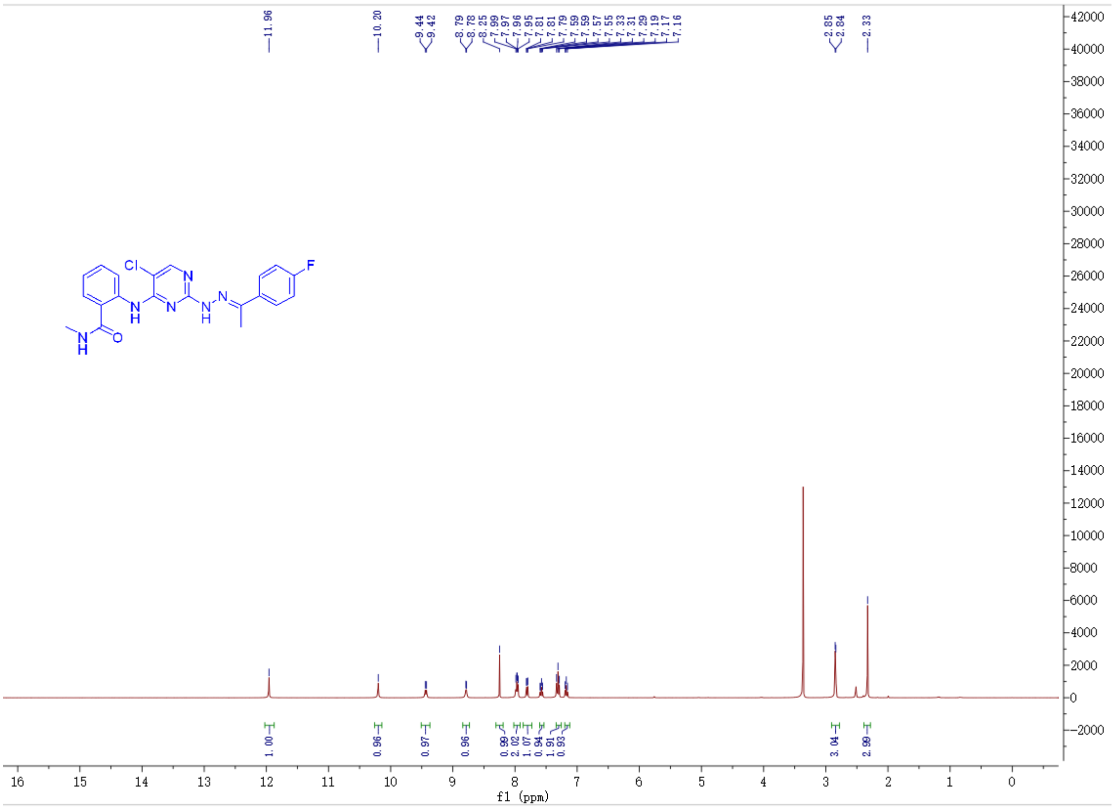

Figure S84. ^{1}H NMR spectrum of compound **14ad** (400 MHz, DMSO-*d6*)

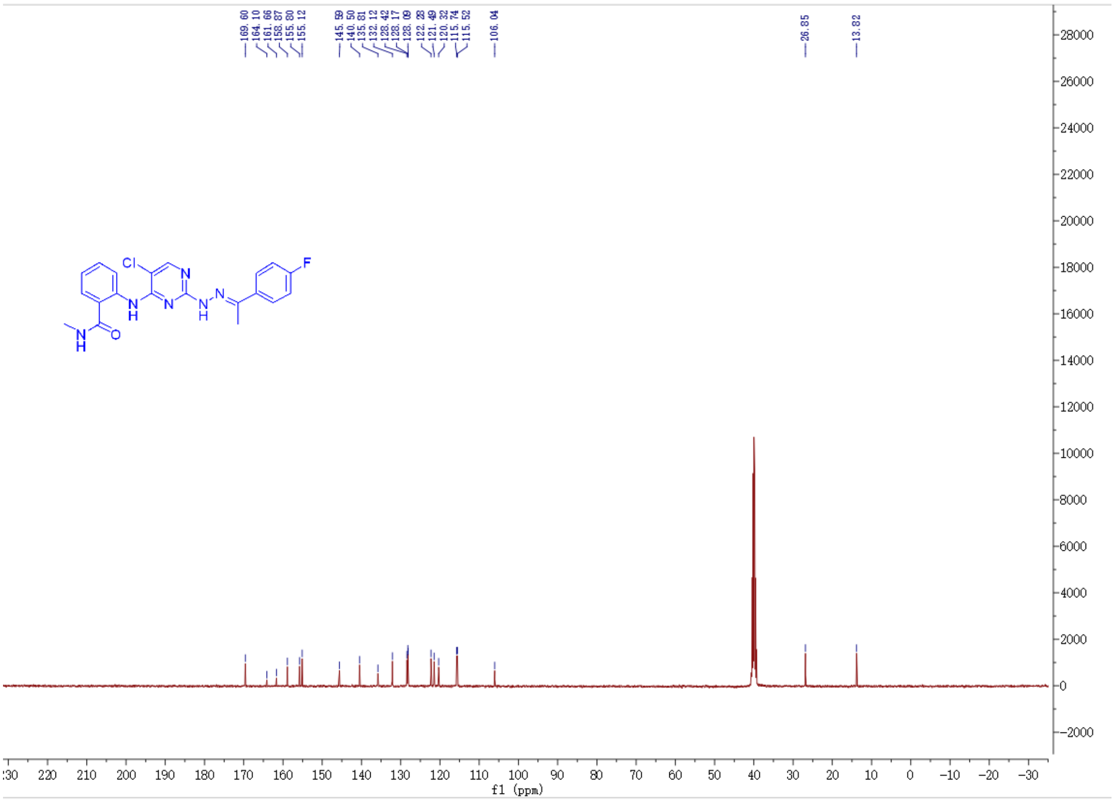

Figure S85. ^{13}C NMR spectrum of compound **14ad** (100 MHz, DMSO-*d6*)

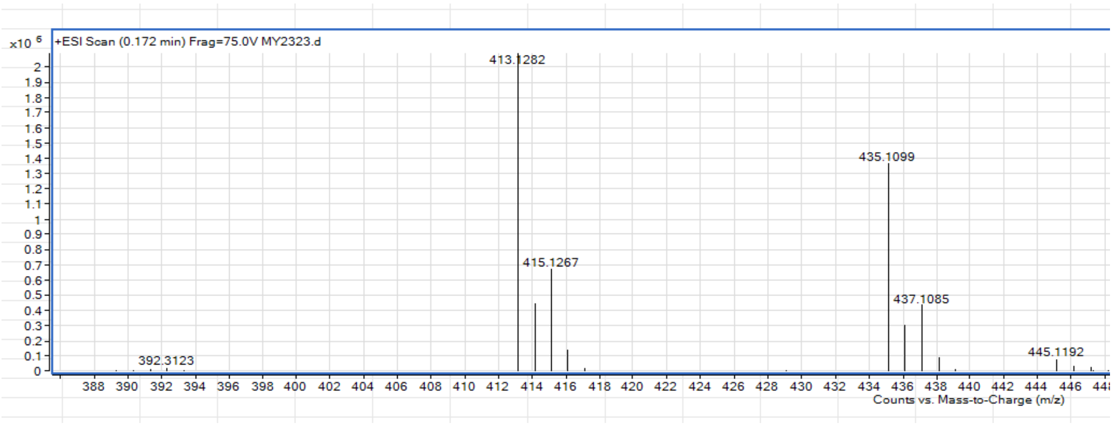

Figure S86. HRMS spectrum of compound **14ad**
