# Supplementary figures and images for: Discovery of novel 2,4-diarylaminopyrimidine hydrazone derivatives as potent anti-thyroid cancer agents capable of inhibiting FAK
Source: J Enzyme Inhib Med Chem. 2024 Nov 19;39(1):2423875. doi: 10.1080/14756366.2024.2423875 (PMC11578424; doi:10.1080/14756366.2024.2423875)

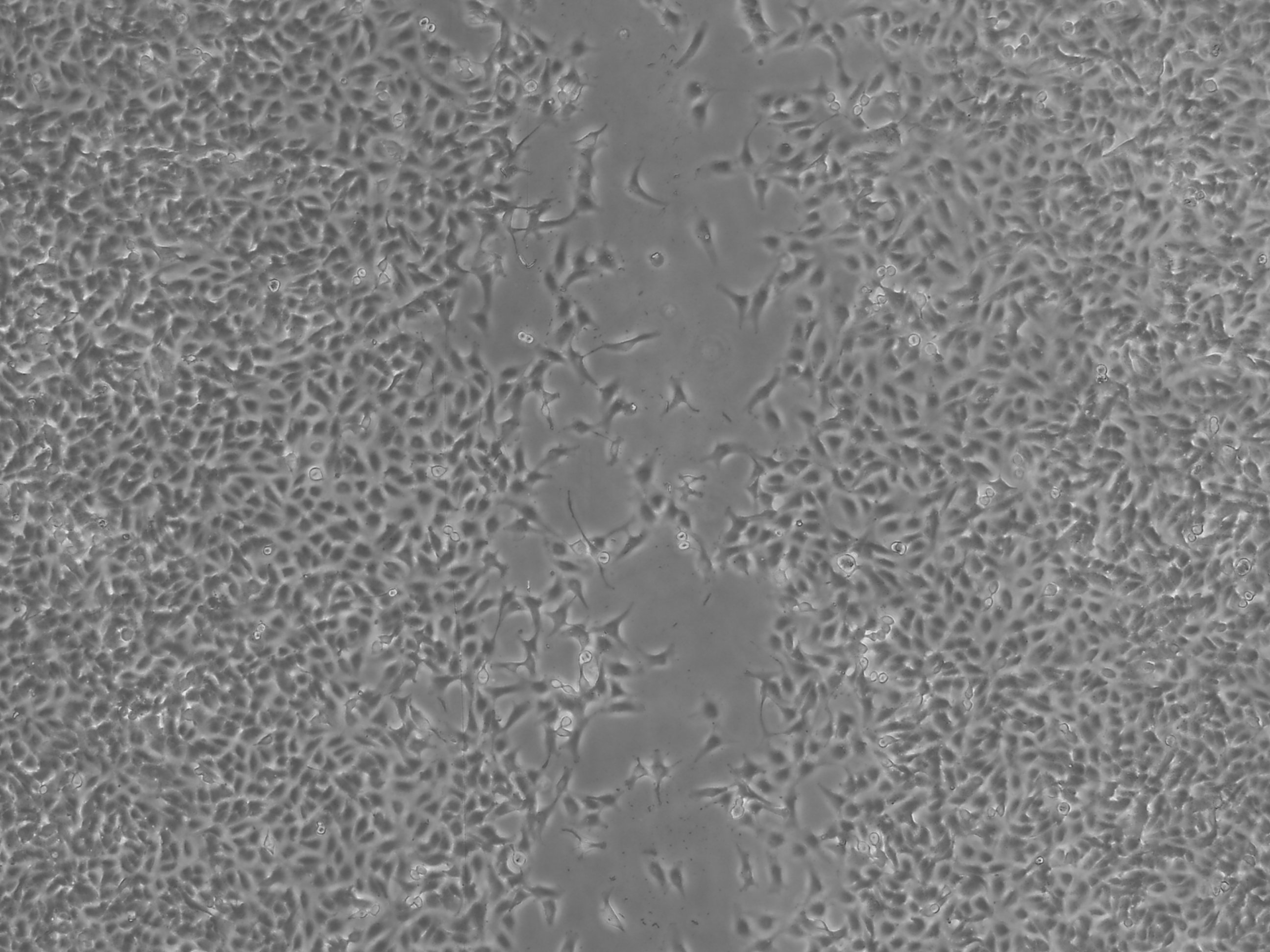

Supplement: Original Image for Figure 7A 36h Control_2.tif [file IENZ_A_2423875_SM5361.tif]

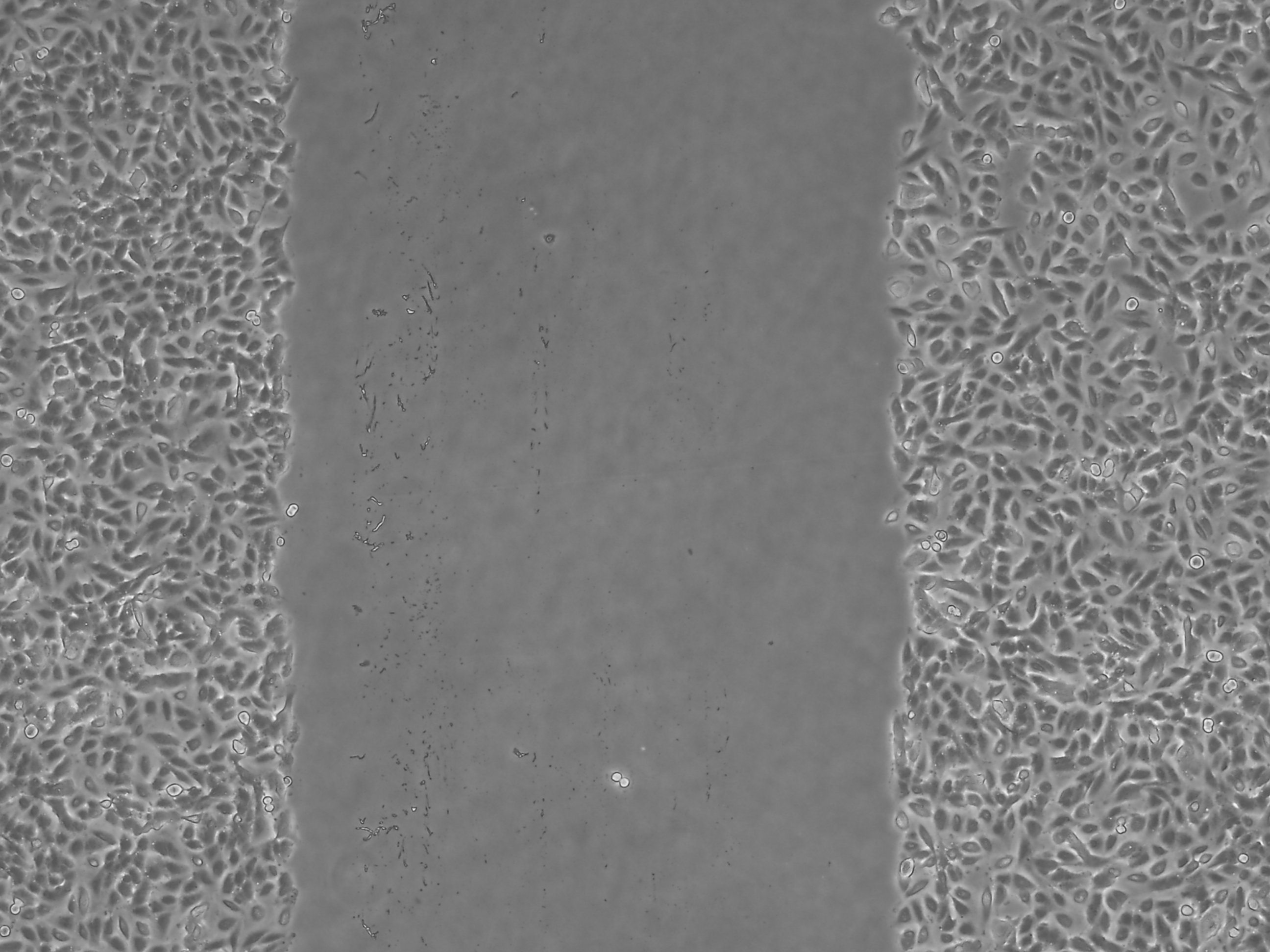

Supplement: Original Image for Figure 7A 0h Control_3.tif [file IENZ_A_2423875_SM5360.tif]

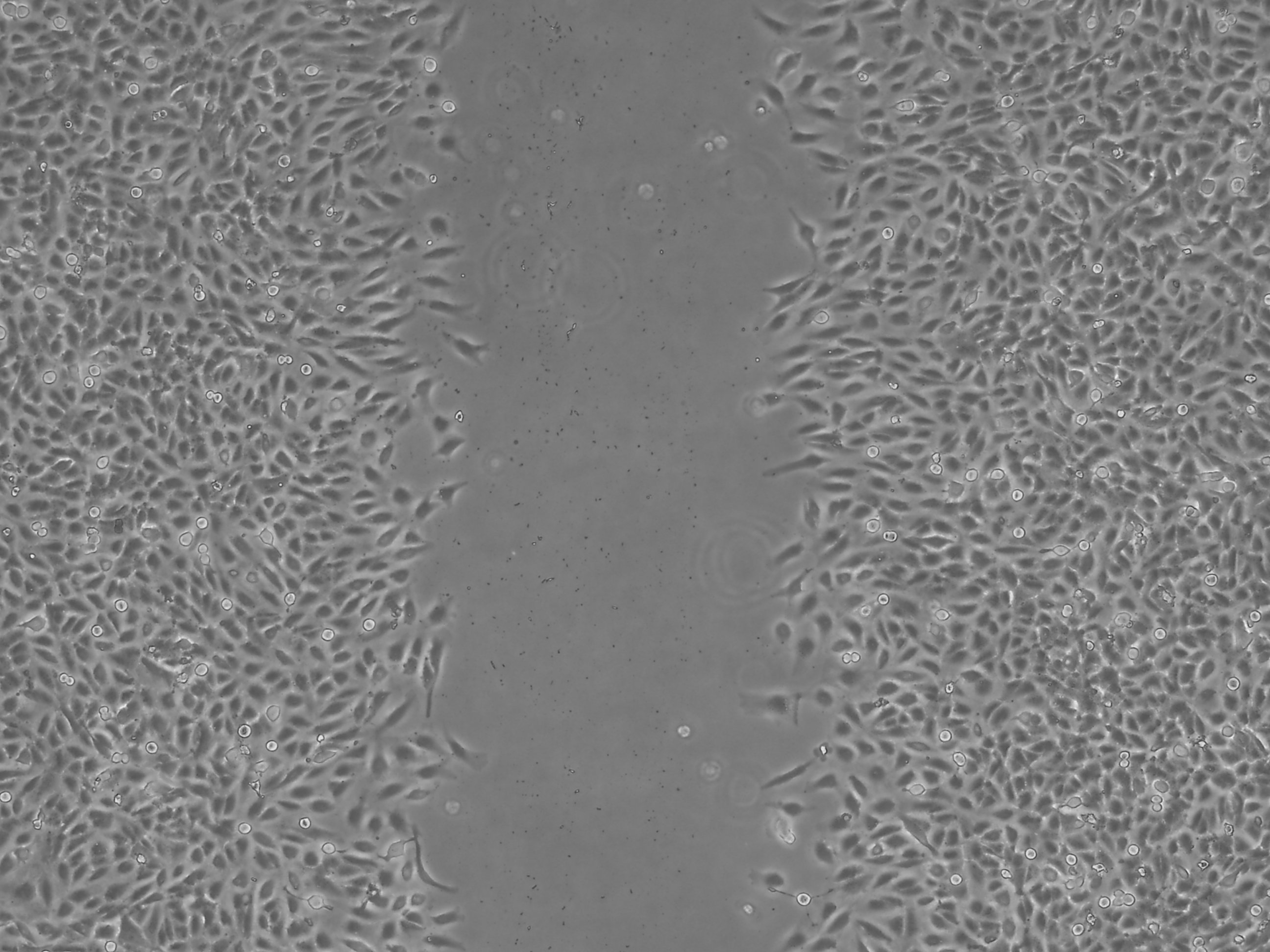

Supplement: Original Image for Figure 7A 12h 300 nM_3.tif [file IENZ_A_2423875_SM5359.tif]

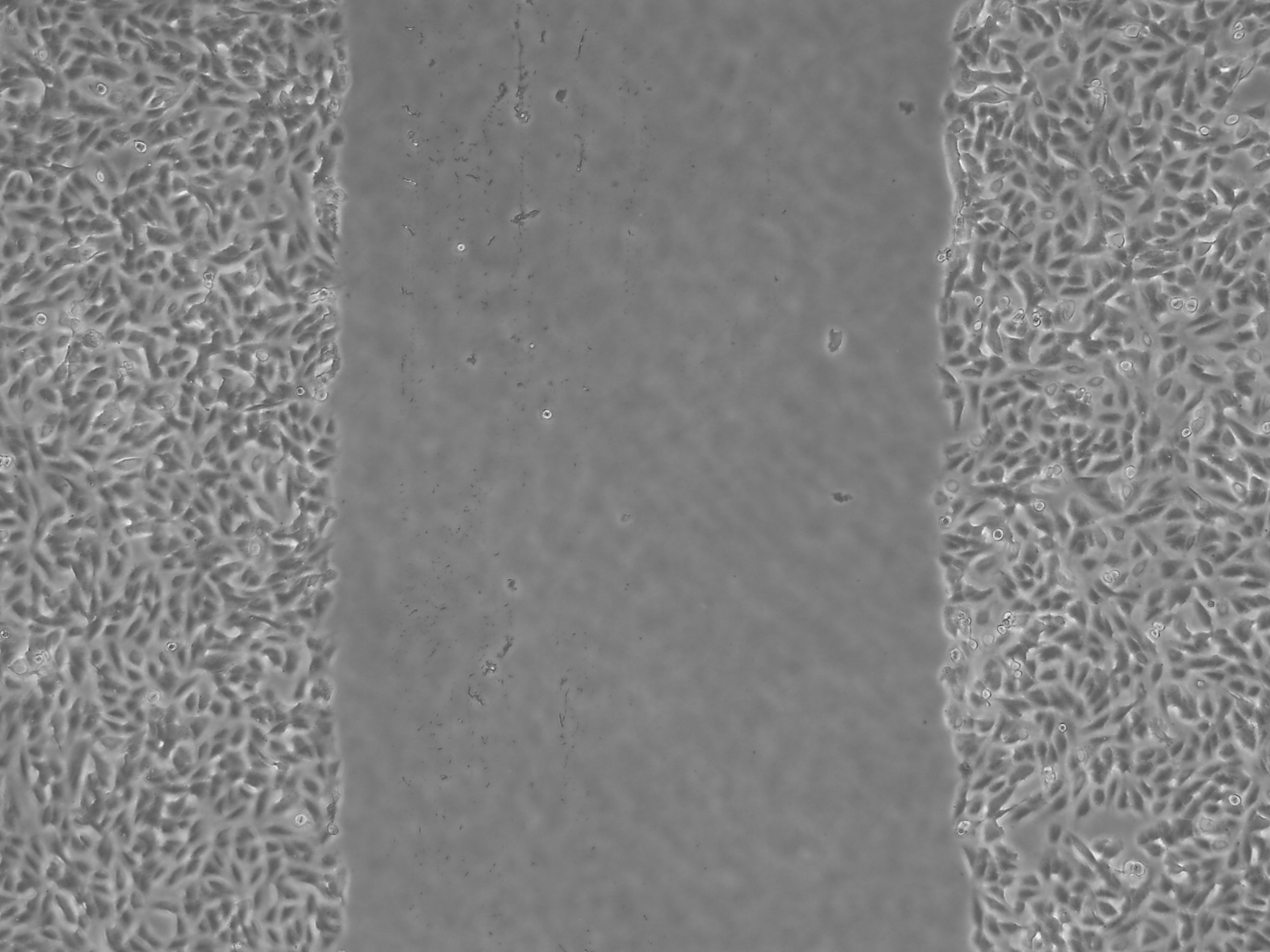

Supplement: Original Image for Figure 7A 0h 600 nM_1.tif [file IENZ_A_2423875_SM5358.tif]

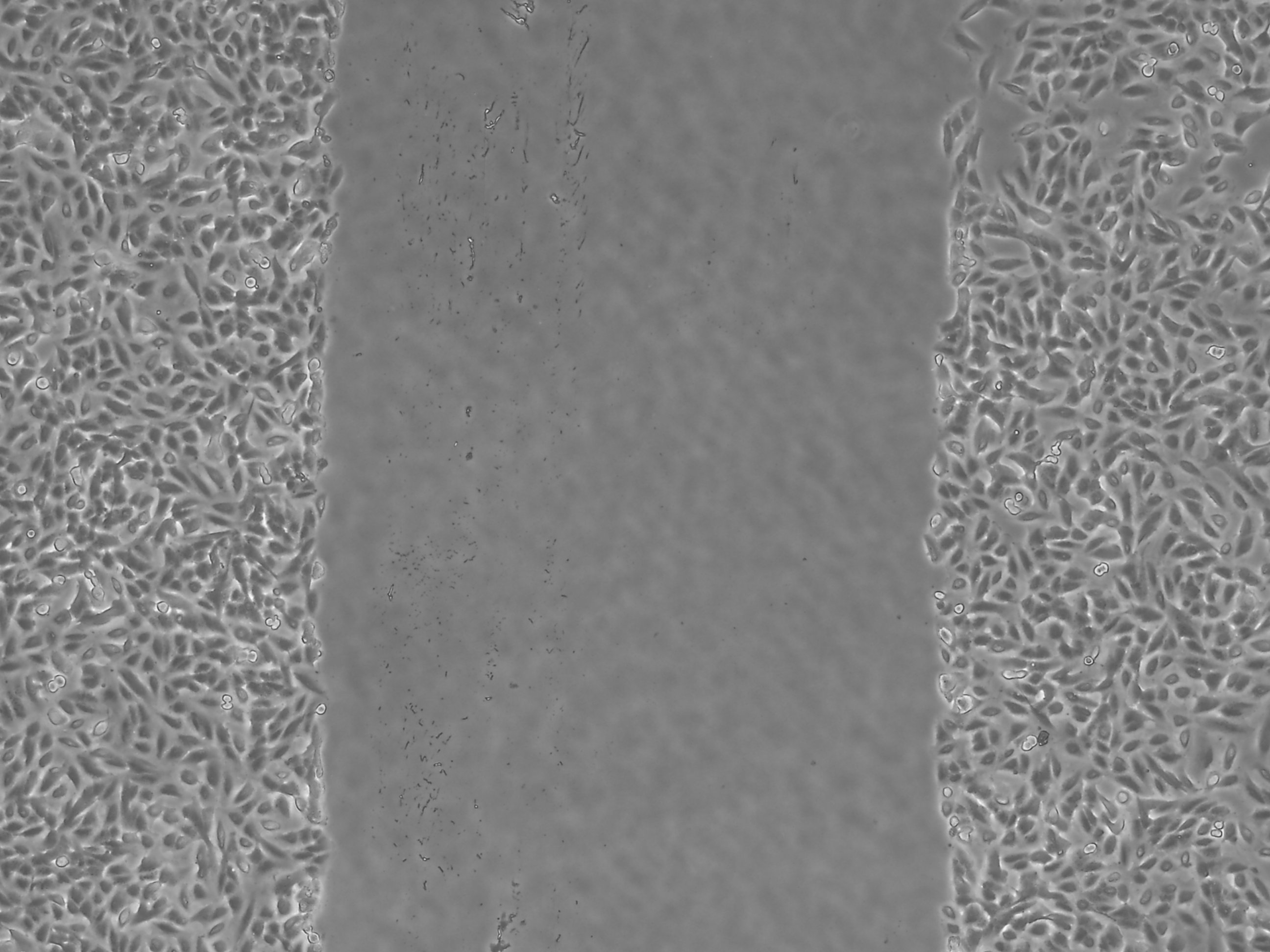

Supplement: Original Image for Figure 7A 0h 600 nM_2.tif [file IENZ_A_2423875_SM5357.tif]

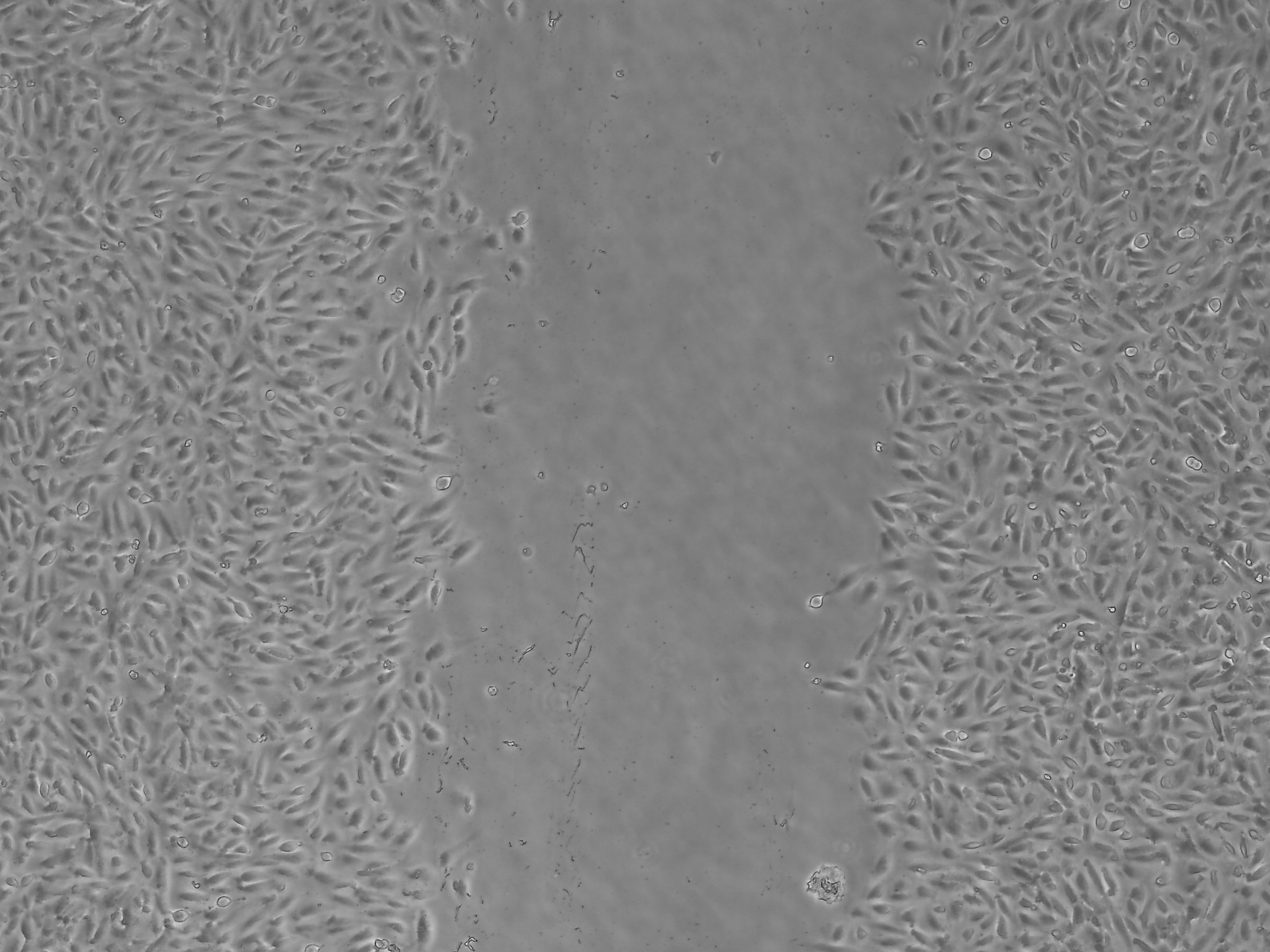

Supplement: Original Image for Figure 7A 24h 600 nM_3.tif [file IENZ_A_2423875_SM5356.tif]

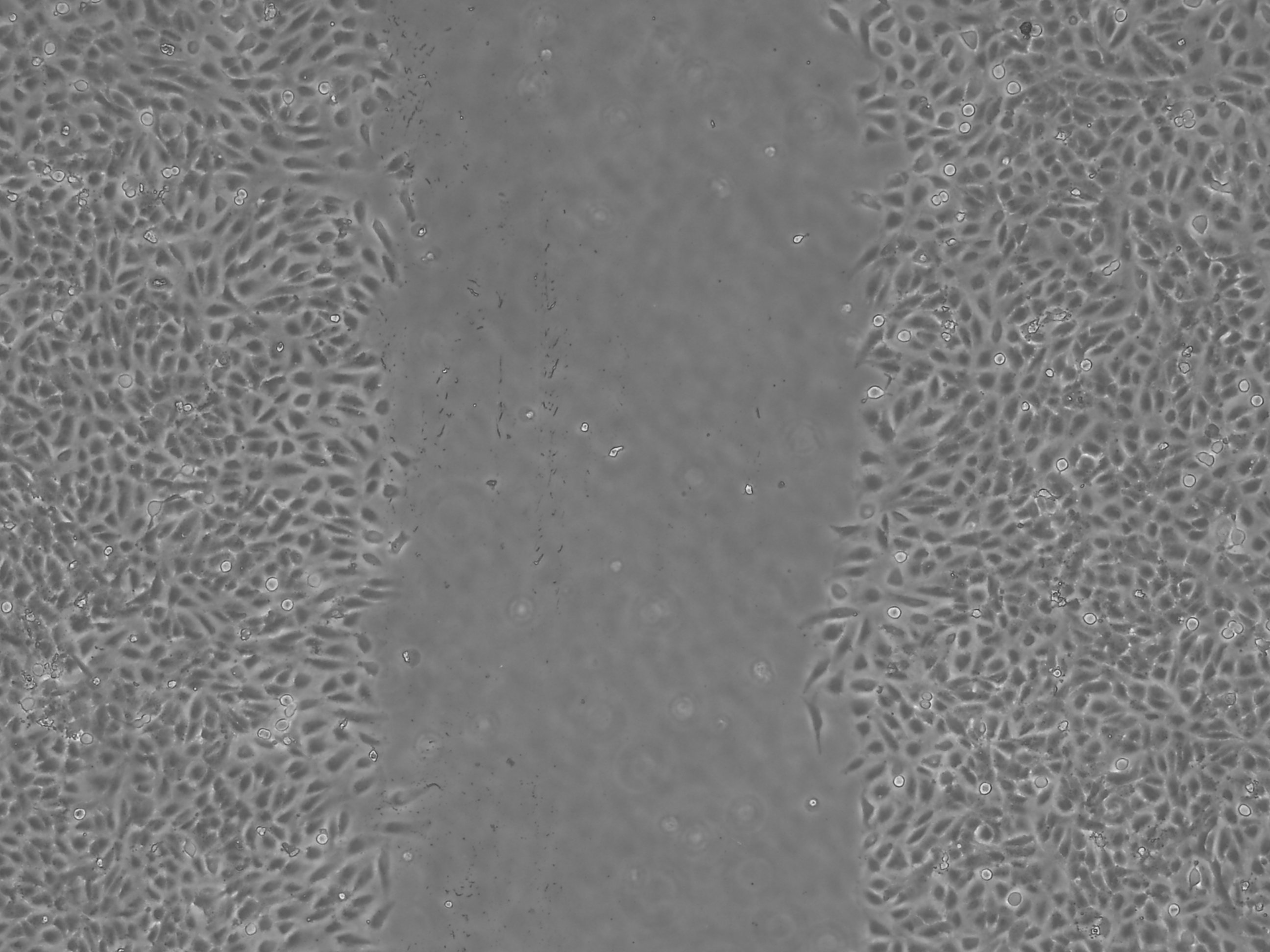

Supplement: Original Image for Figure 7A 12h 600 nM_3.tif [file IENZ_A_2423875_SM5354.tif]

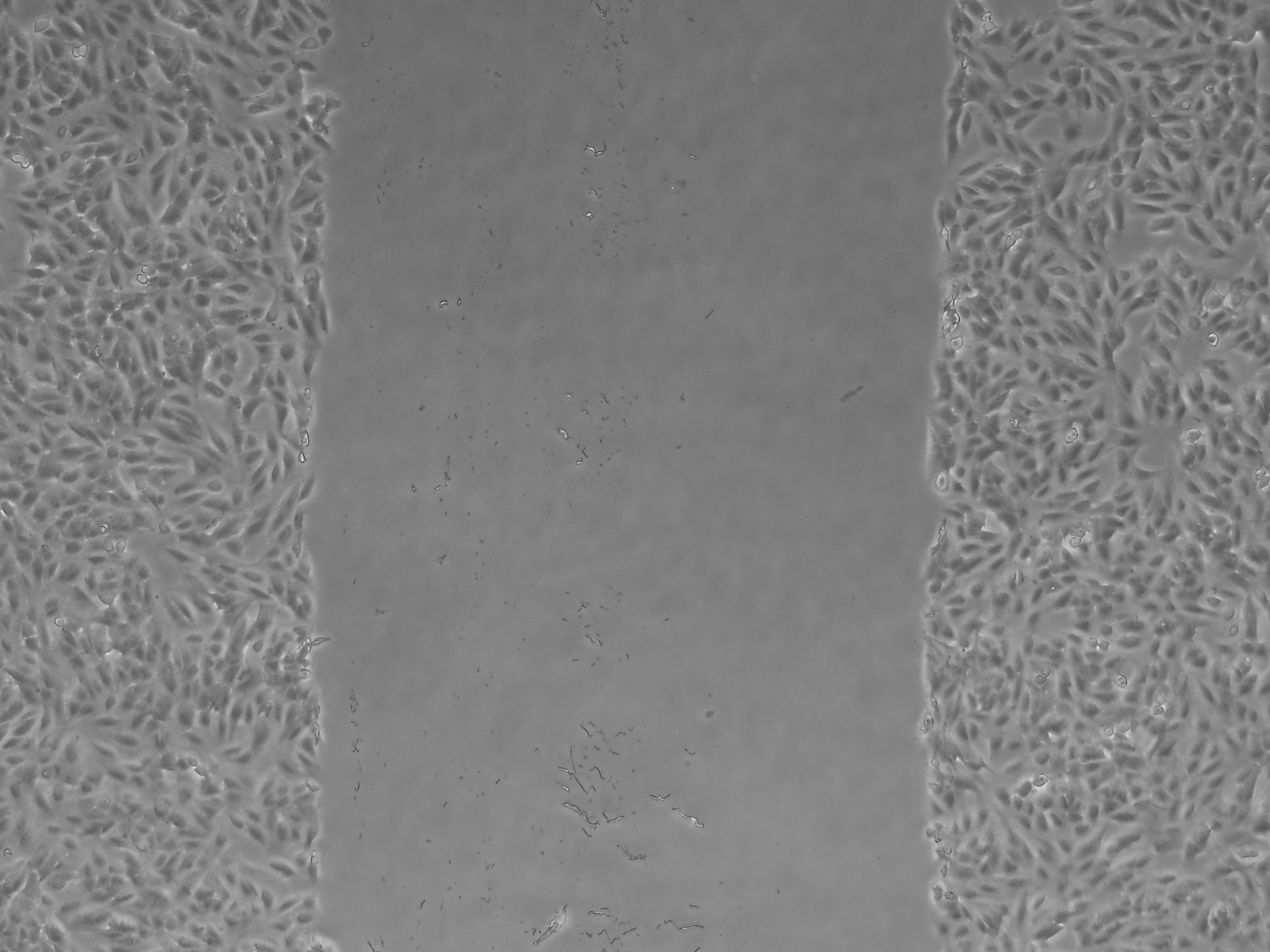

Supplement: Original Image for Figure 7A 0h 900 nM_2.tif [file IENZ_A_2423875_SM5353.tif]

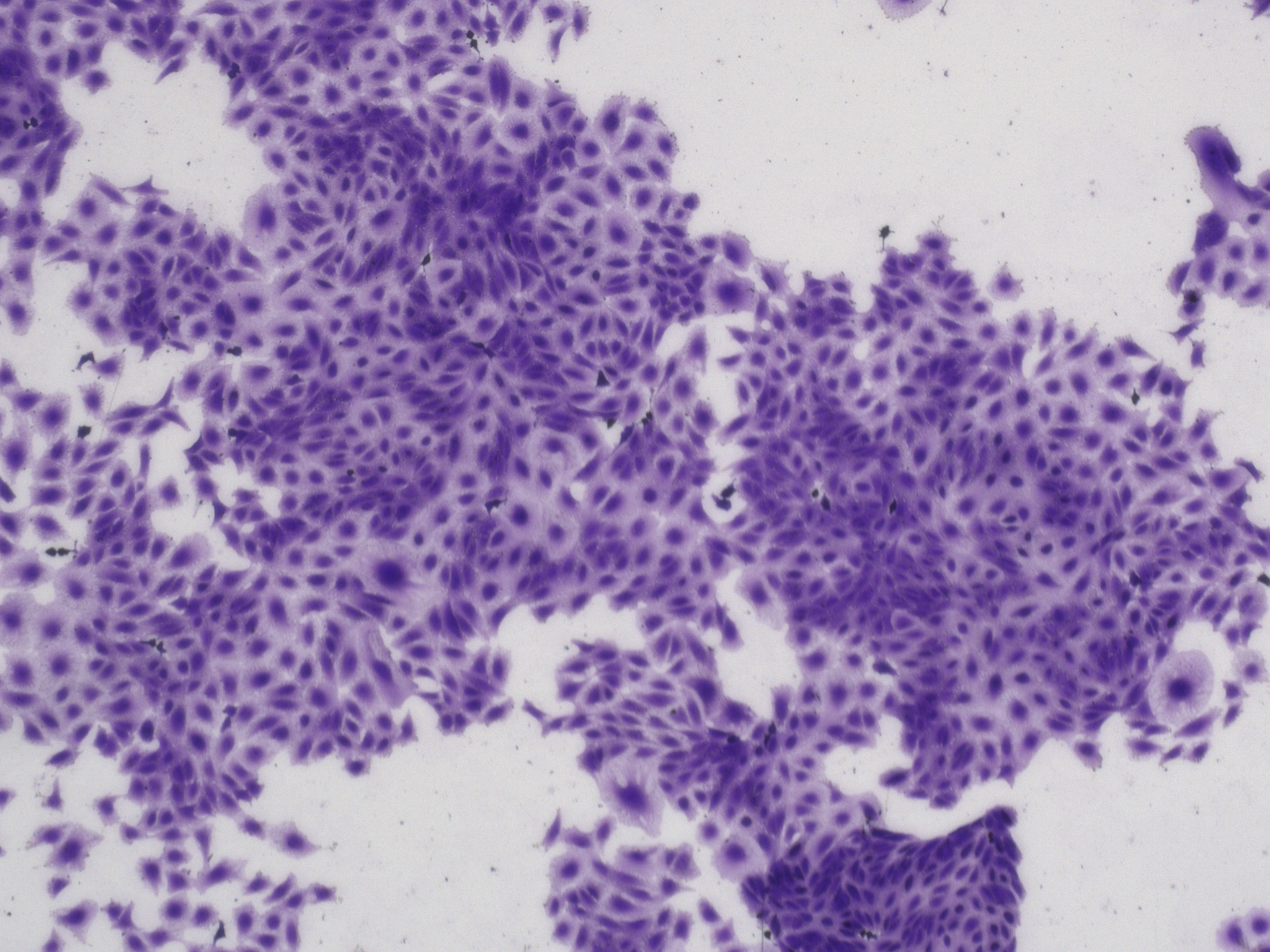

Supplement: Original Image for Figure 6B 300 nM.TIF [file IENZ_A_2423875_SM5352.tif]

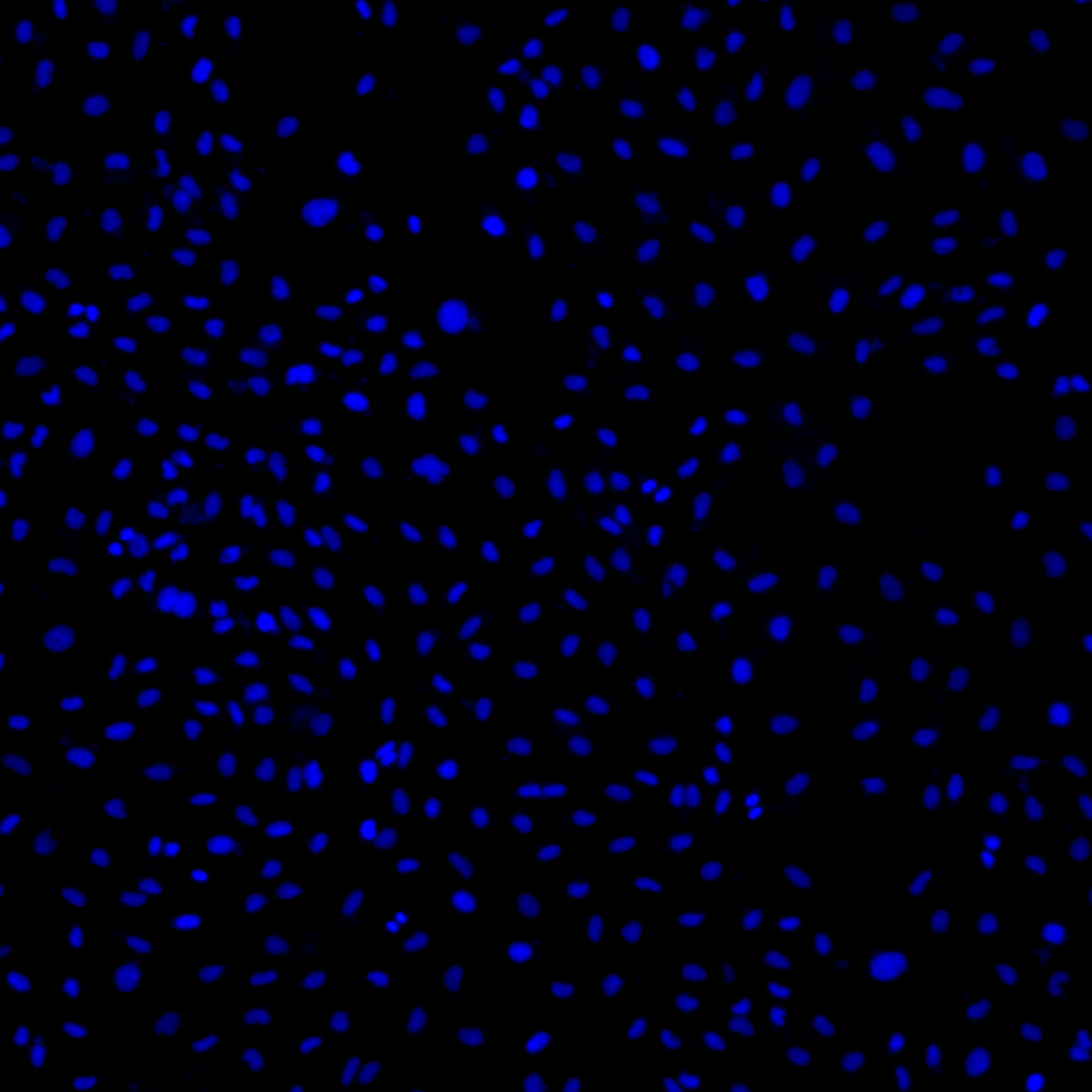

Supplement: Original Image for Figure 6A 300 nM_Hoechst.tif [file IENZ_A_2423875_SM5351.tif]

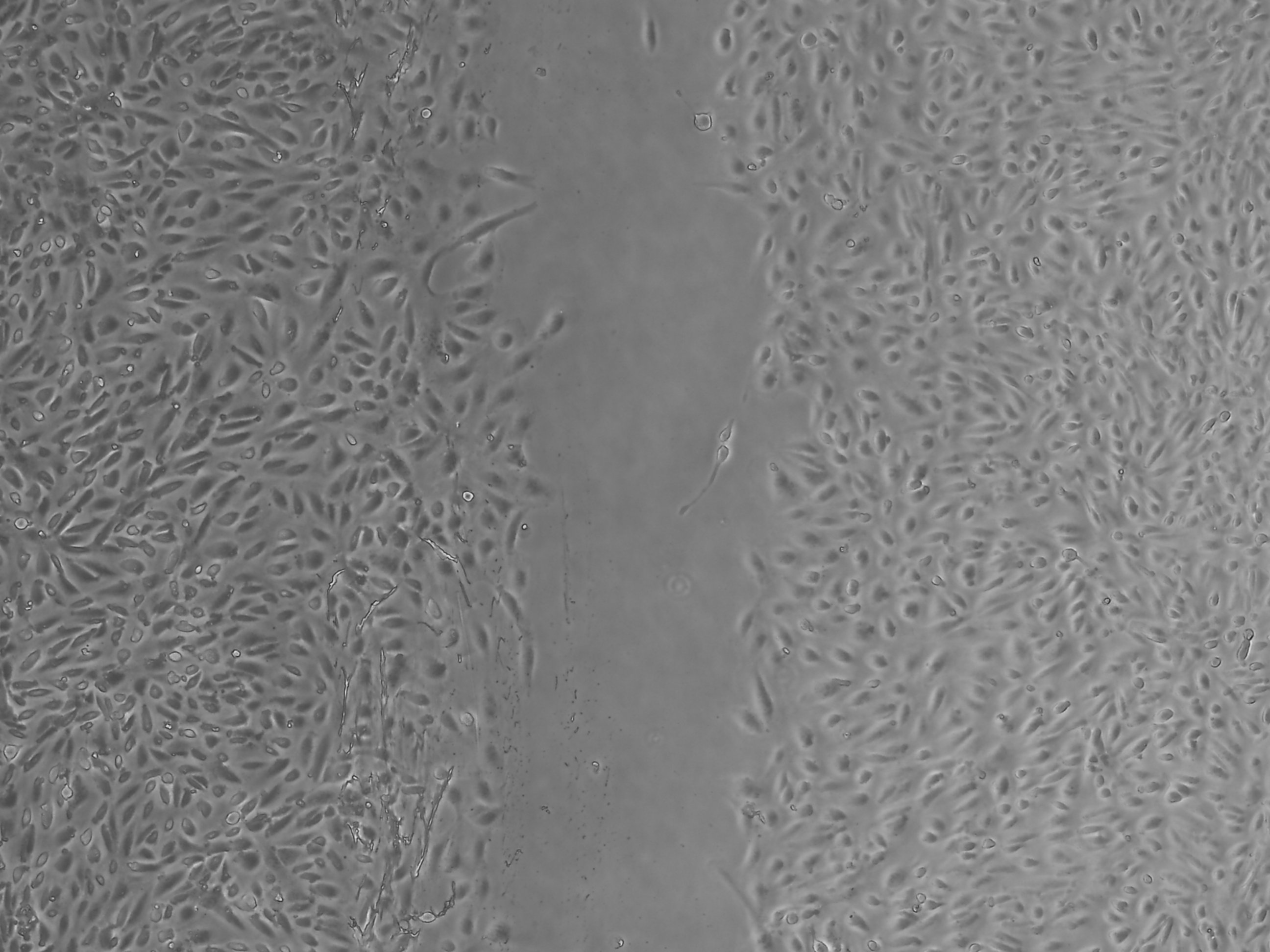

Supplement: Original Image for Figure 7A 48h 600 nM_2.tif [file IENZ_A_2423875_SM5350.tif]

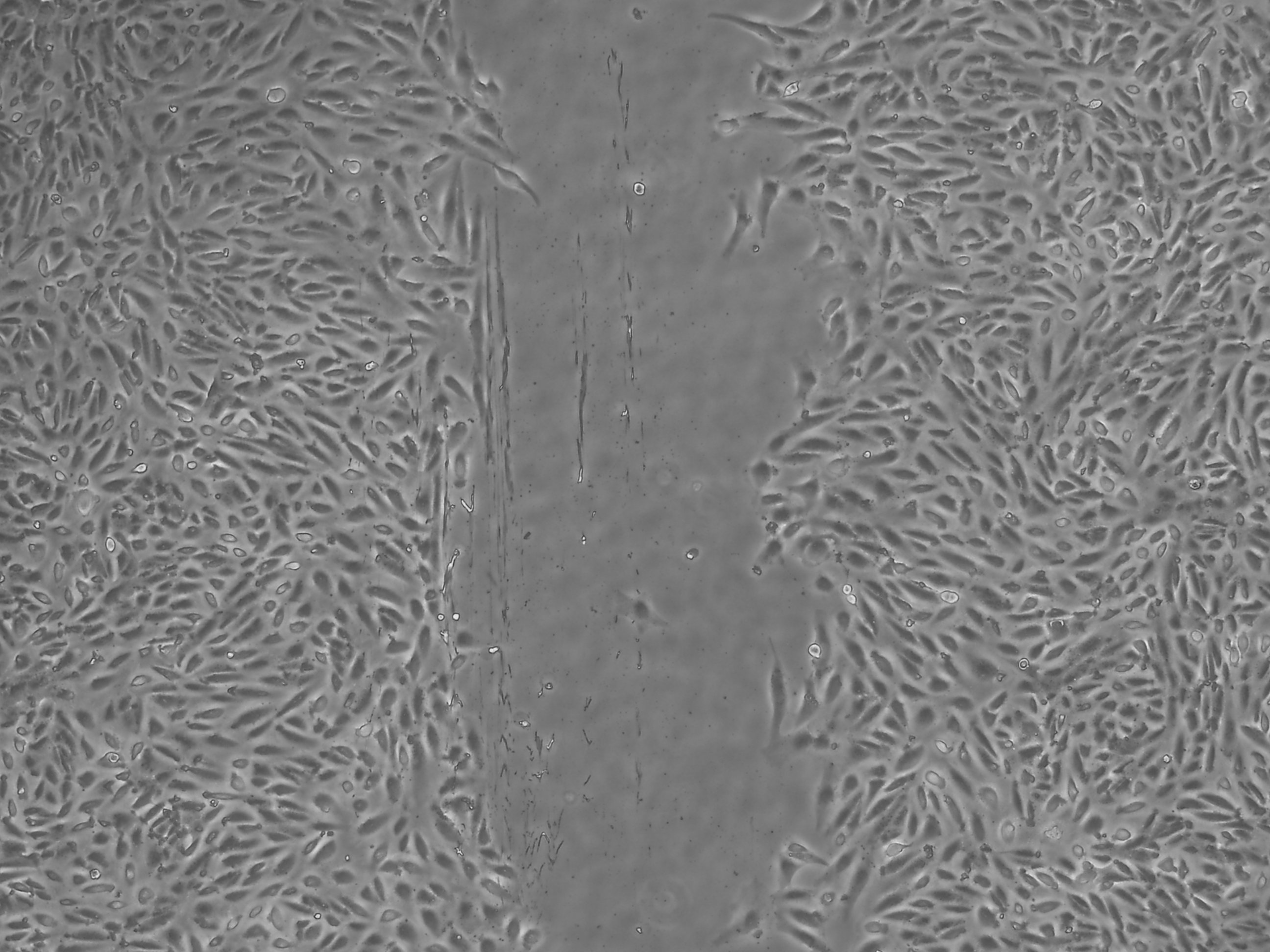

Supplement: Original Image for Figure 7A 36h 900 nM_2.tif [file IENZ_A_2423875_SM5349.tif]

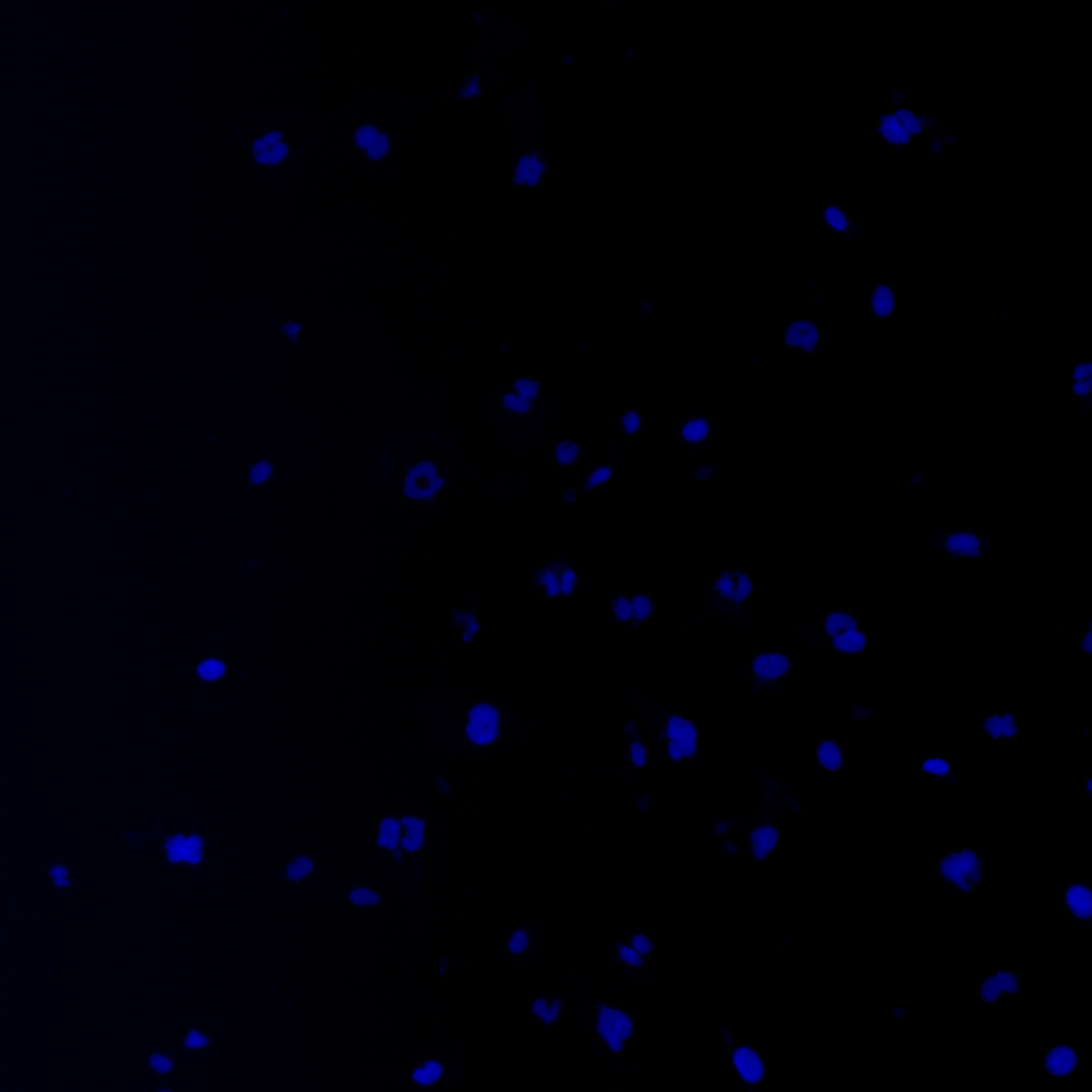

Supplement: Original Image for Figure 6A 900 nM_Hoechst.tif [file IENZ_A_2423875_SM5348.tif]

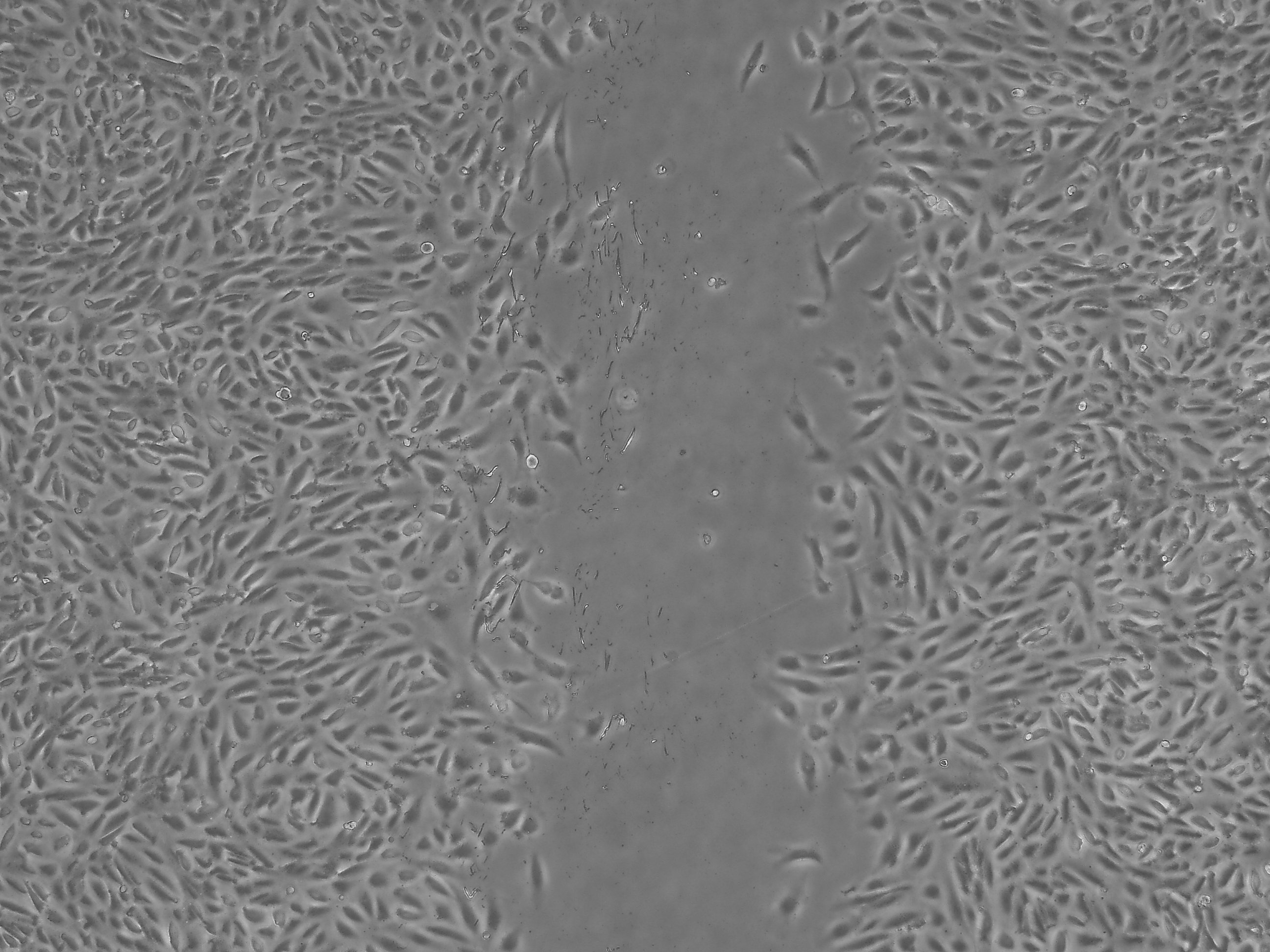

Supplement: Original Image for Figure 7A 36h 900 nM_3.tif [file IENZ_A_2423875_SM5346.tif]

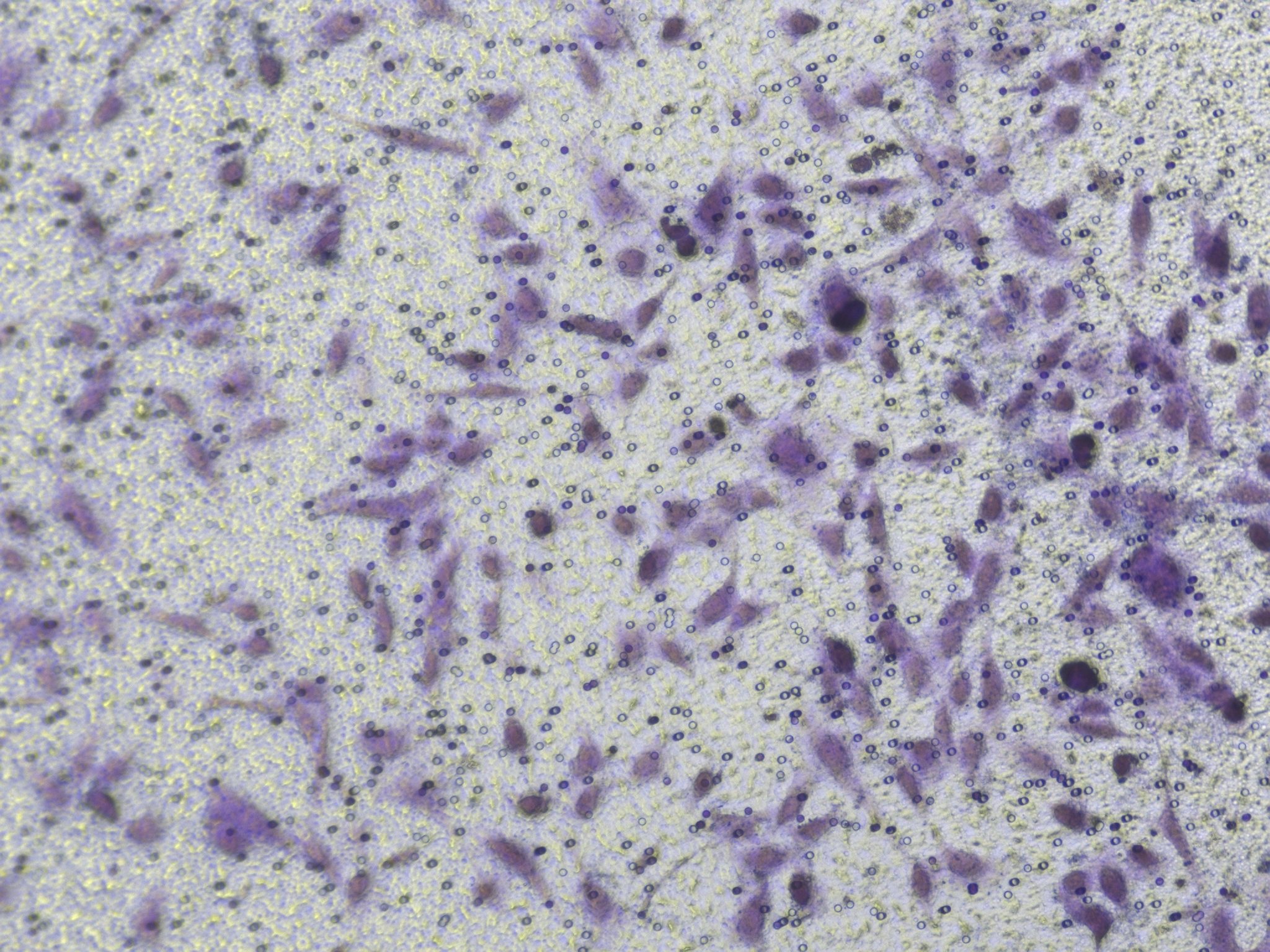

Supplement: Original Image for Figure 7C 600 nM.TIF [file IENZ_A_2423875_SM5345.tif]

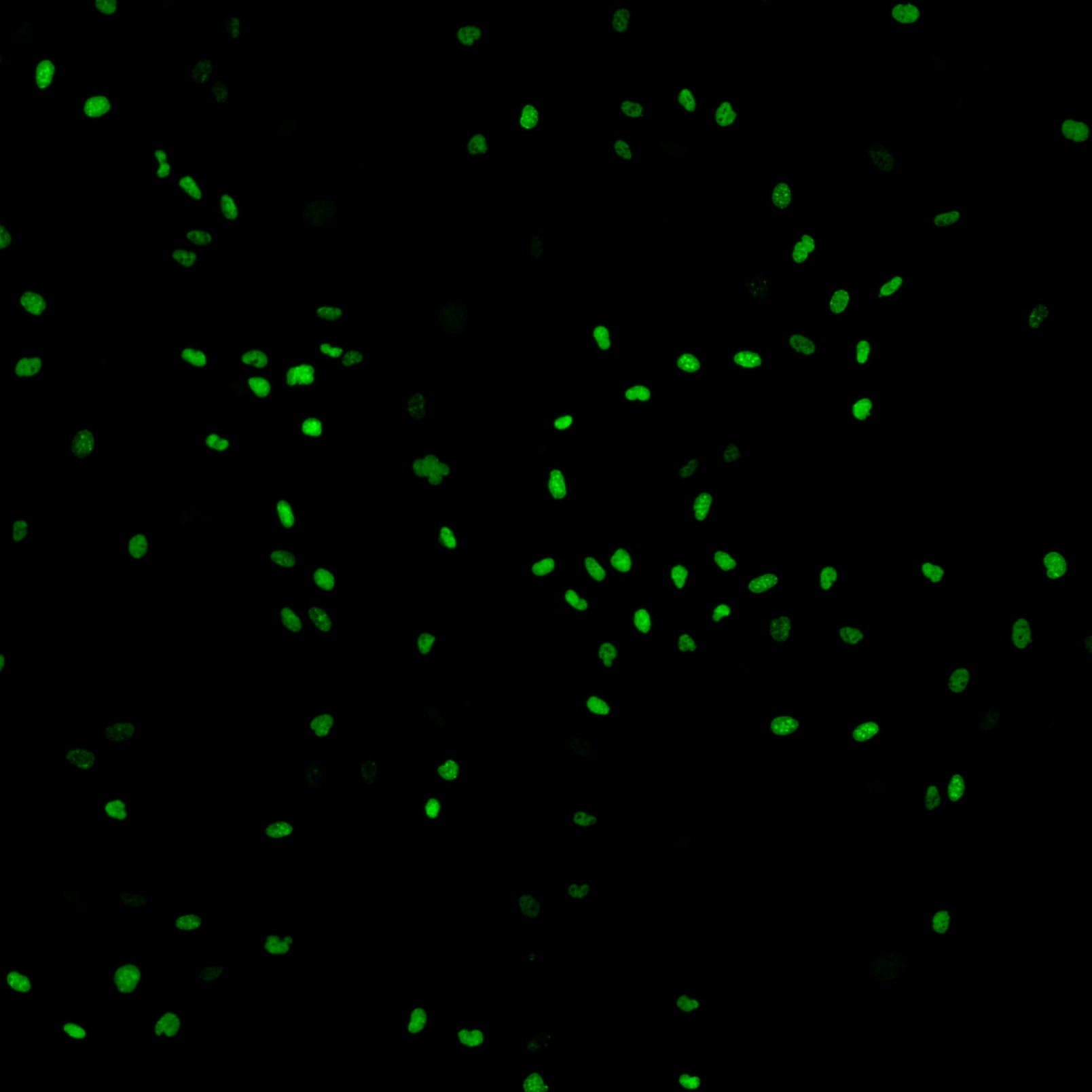

Supplement: Original Image for Figure 6A 300 nM_EdU.tif [file IENZ_A_2423875_SM5344.tif]

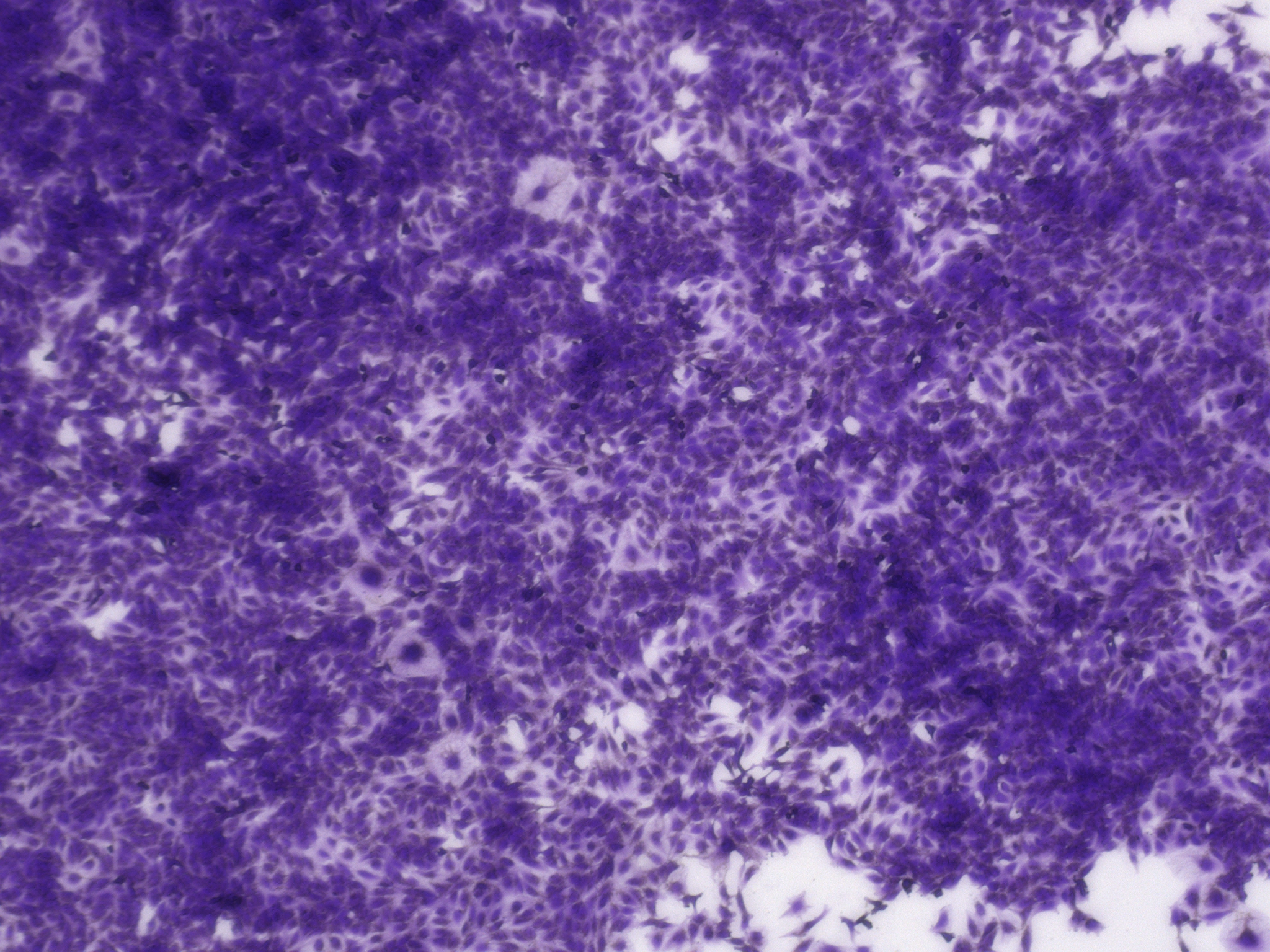

Supplement: Original Image for Figure 6B Control.TIF [file IENZ_A_2423875_SM5343.tif]

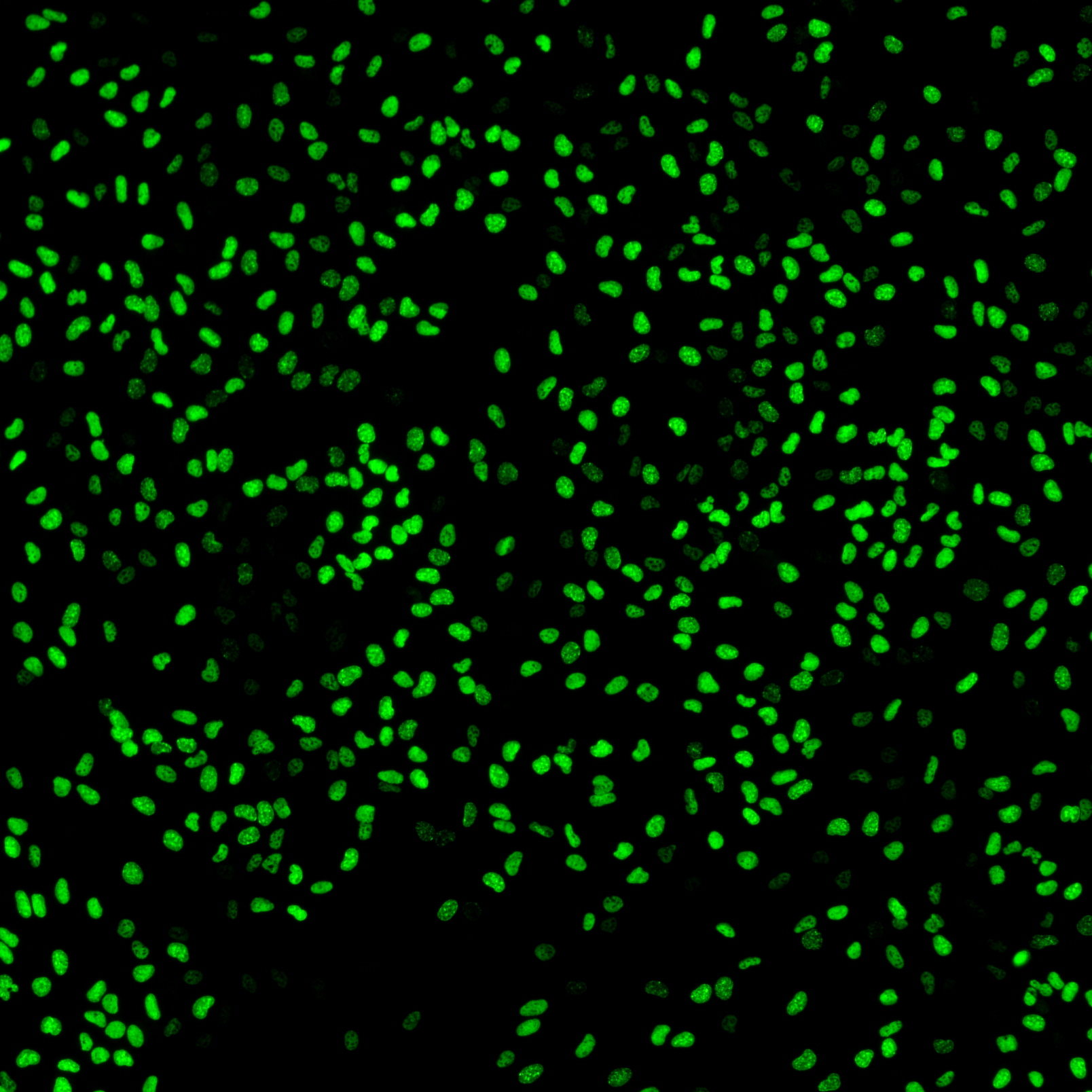

Supplement: Original Image for Figure 6A control_EdU.tif [file IENZ_A_2423875_SM5342.tif]

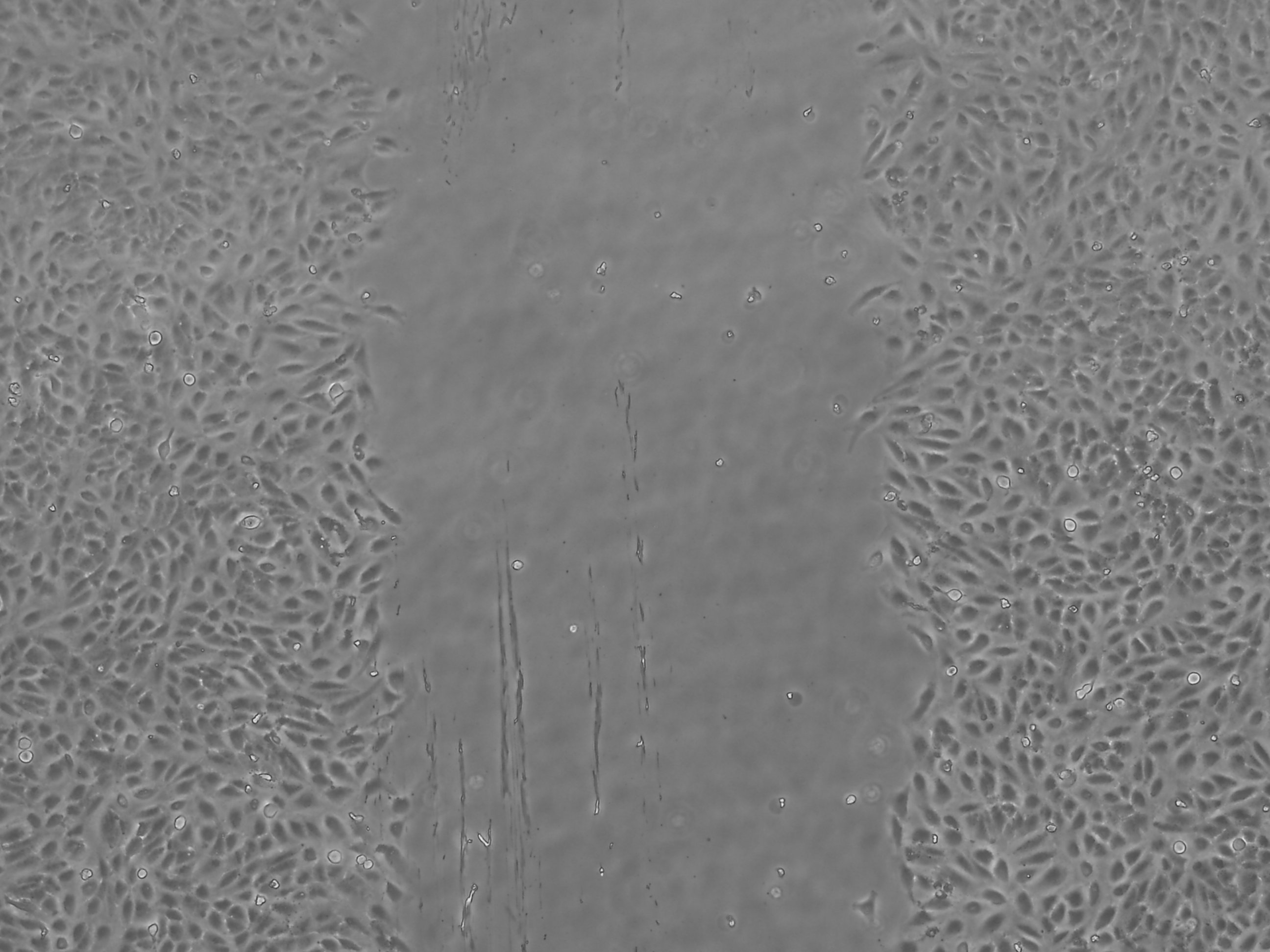

Supplement: Original Image for Figure 7A 12h 900 nM_1.tif [file IENZ_A_2423875_SM5341.tif]

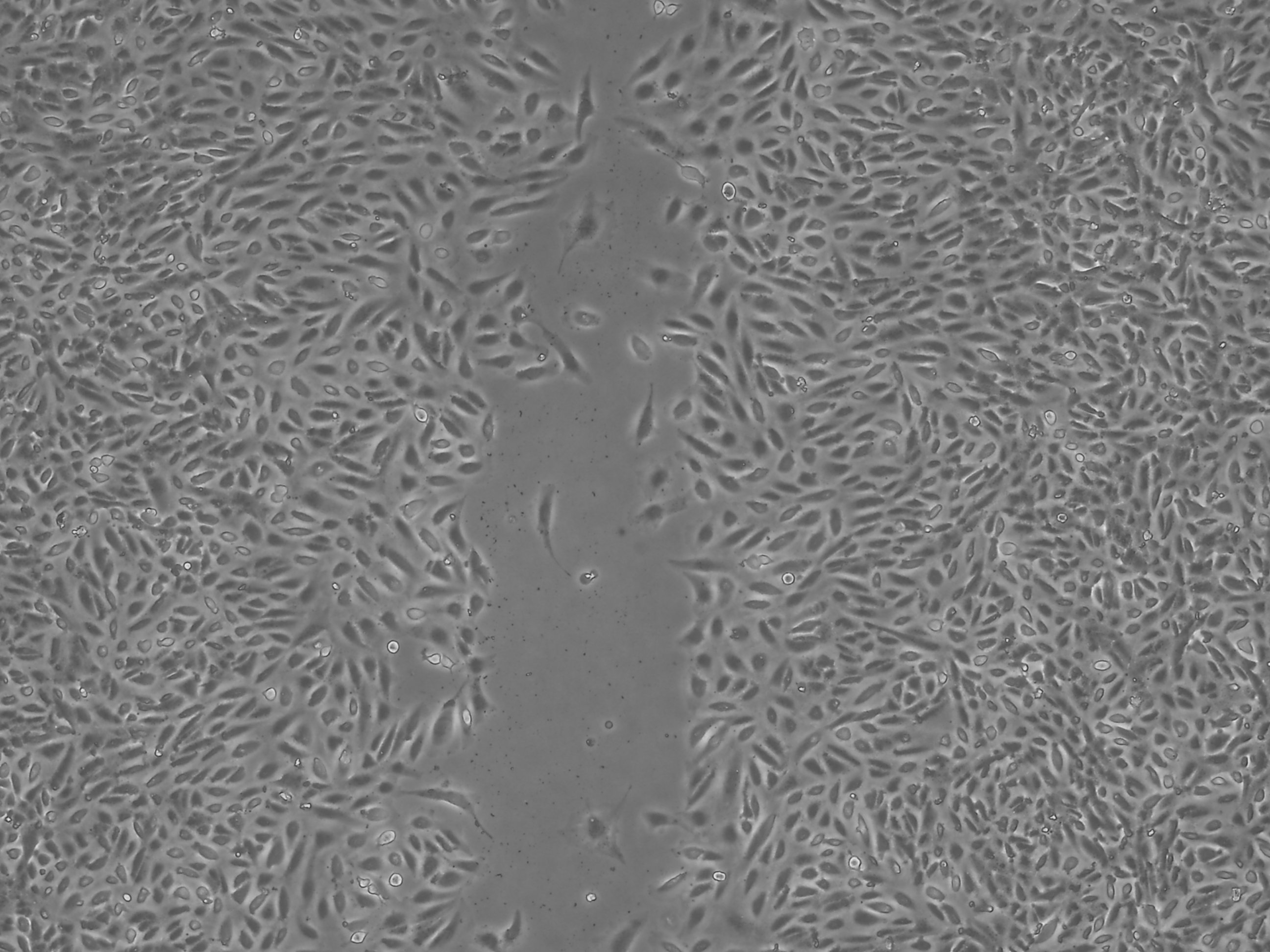

Supplement: Original Image for Figure 7A 36h 300 nM_1.tif [file IENZ_A_2423875_SM5340.tif]

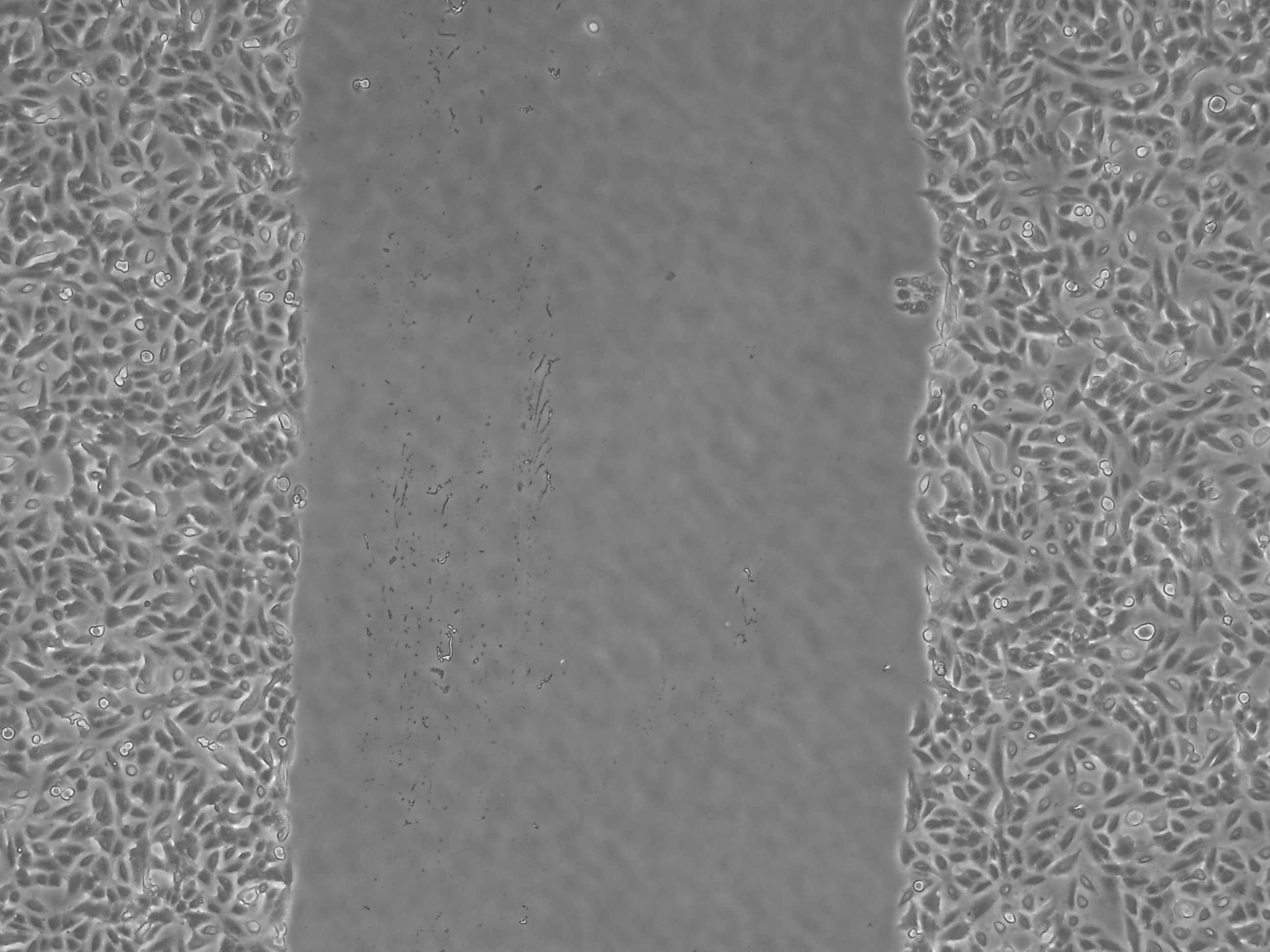

Supplement: Original Image for Figure 7A 0h 600 nM_3.tif [file IENZ_A_2423875_SM5339.tif]

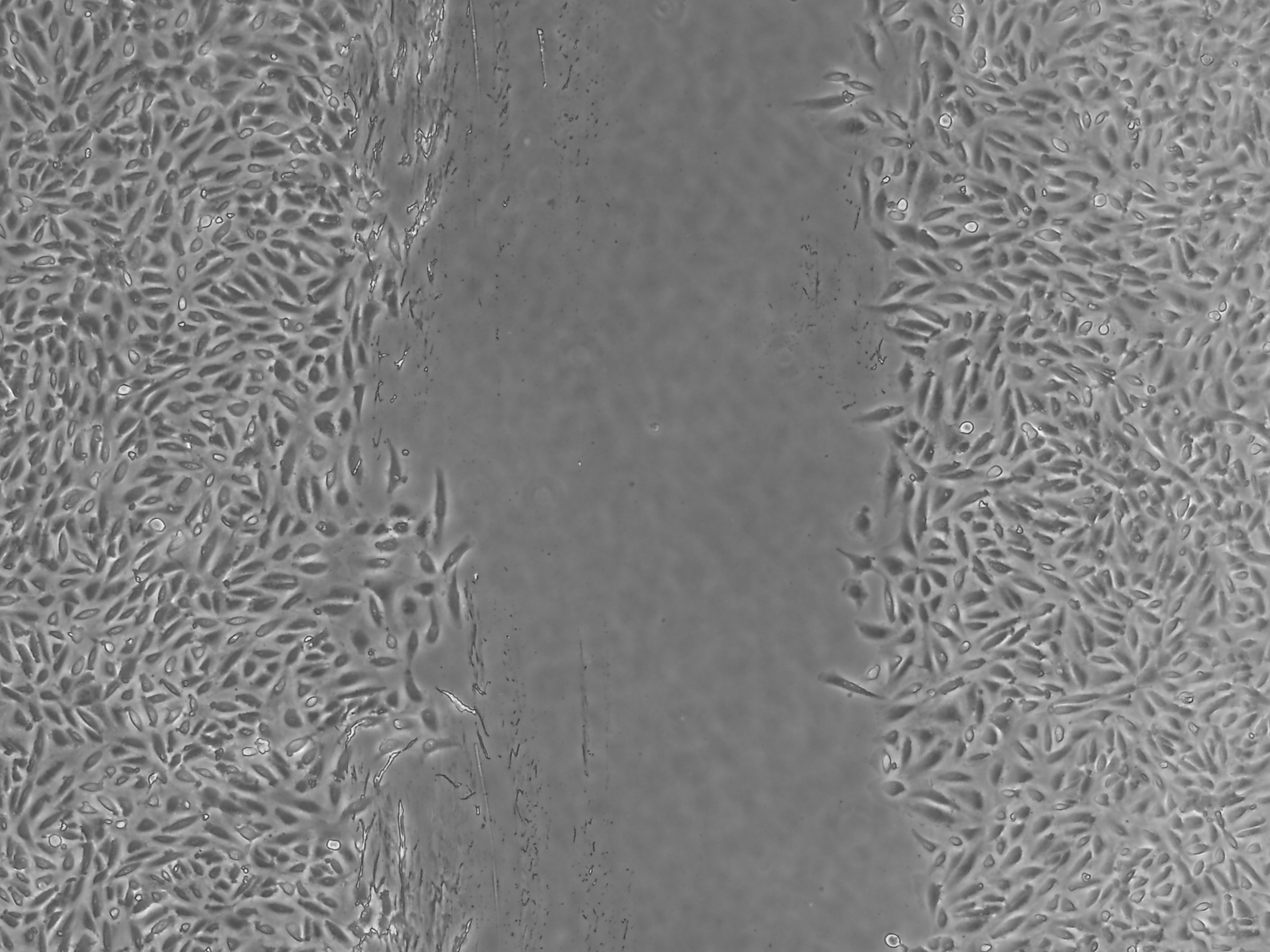

Supplement: Original Image for Figure 7A 24h 600 nM_1.tif [file IENZ_A_2423875_SM5338.tif]

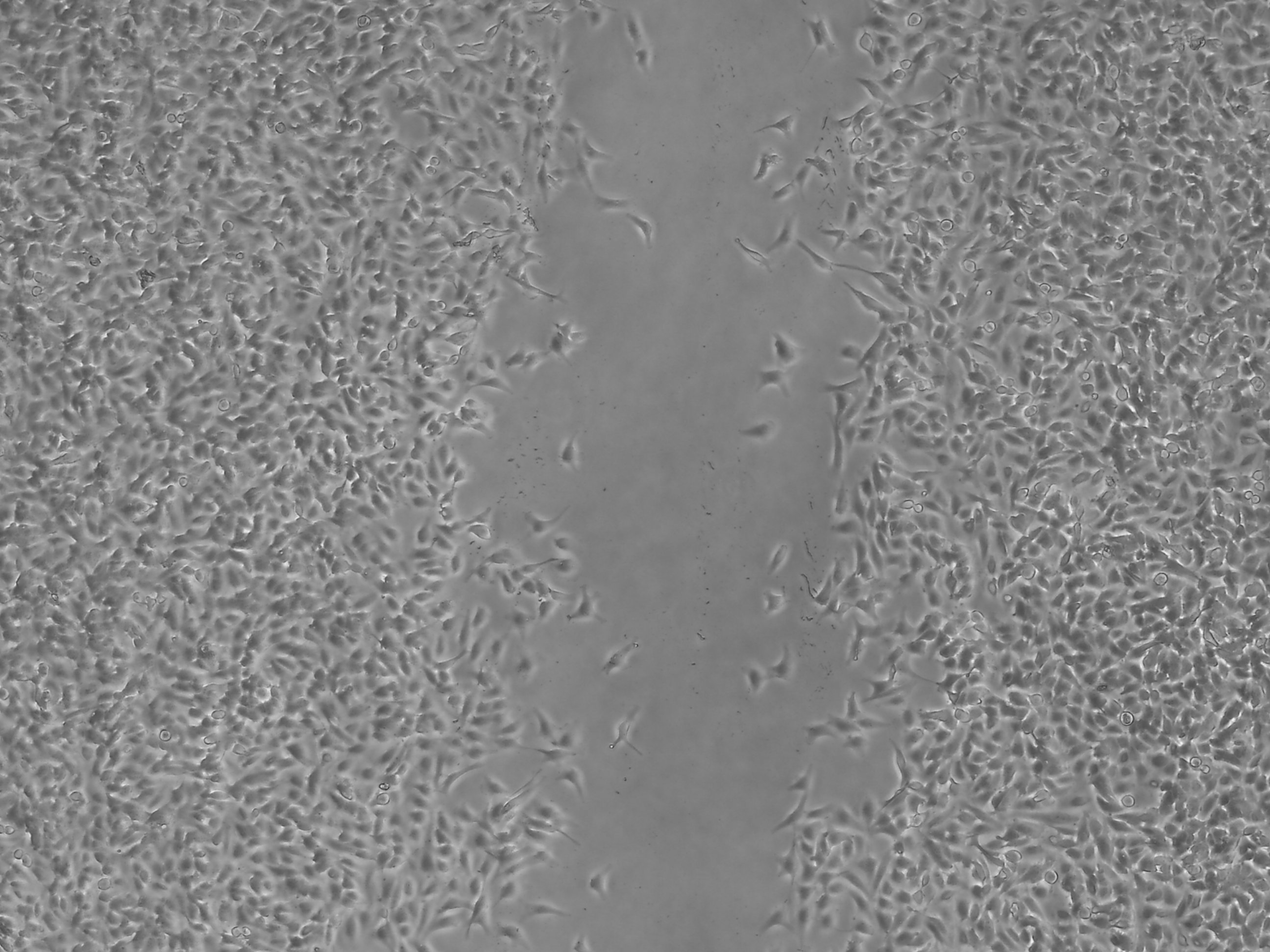

Supplement: Original Image for Figure 7A 24h Control_1.tif [file IENZ_A_2423875_SM5337.tif]

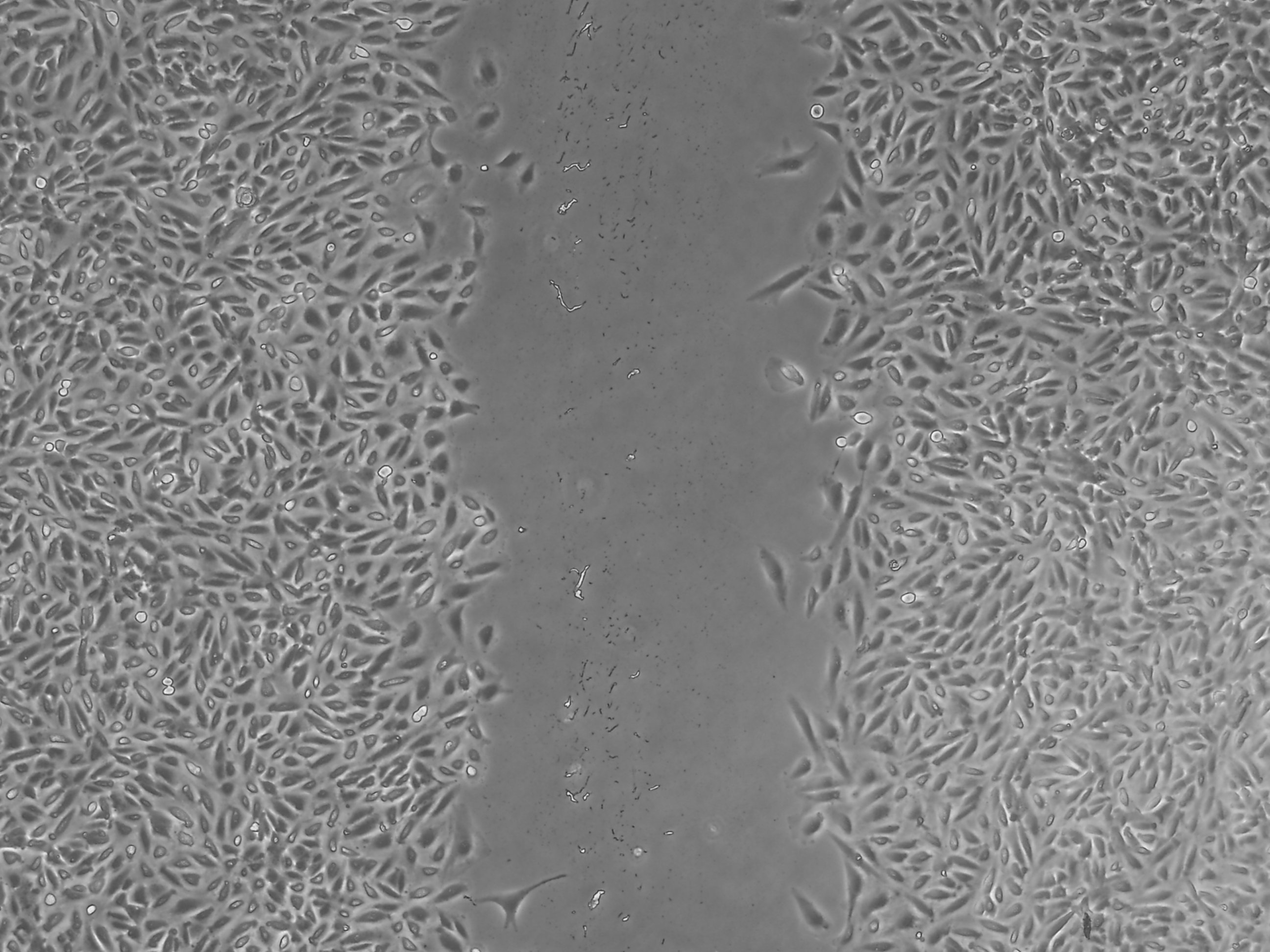

Supplement: Original Image for Figure 7A 24h 300 nM_3.tif [file IENZ_A_2423875_SM5336.tif]

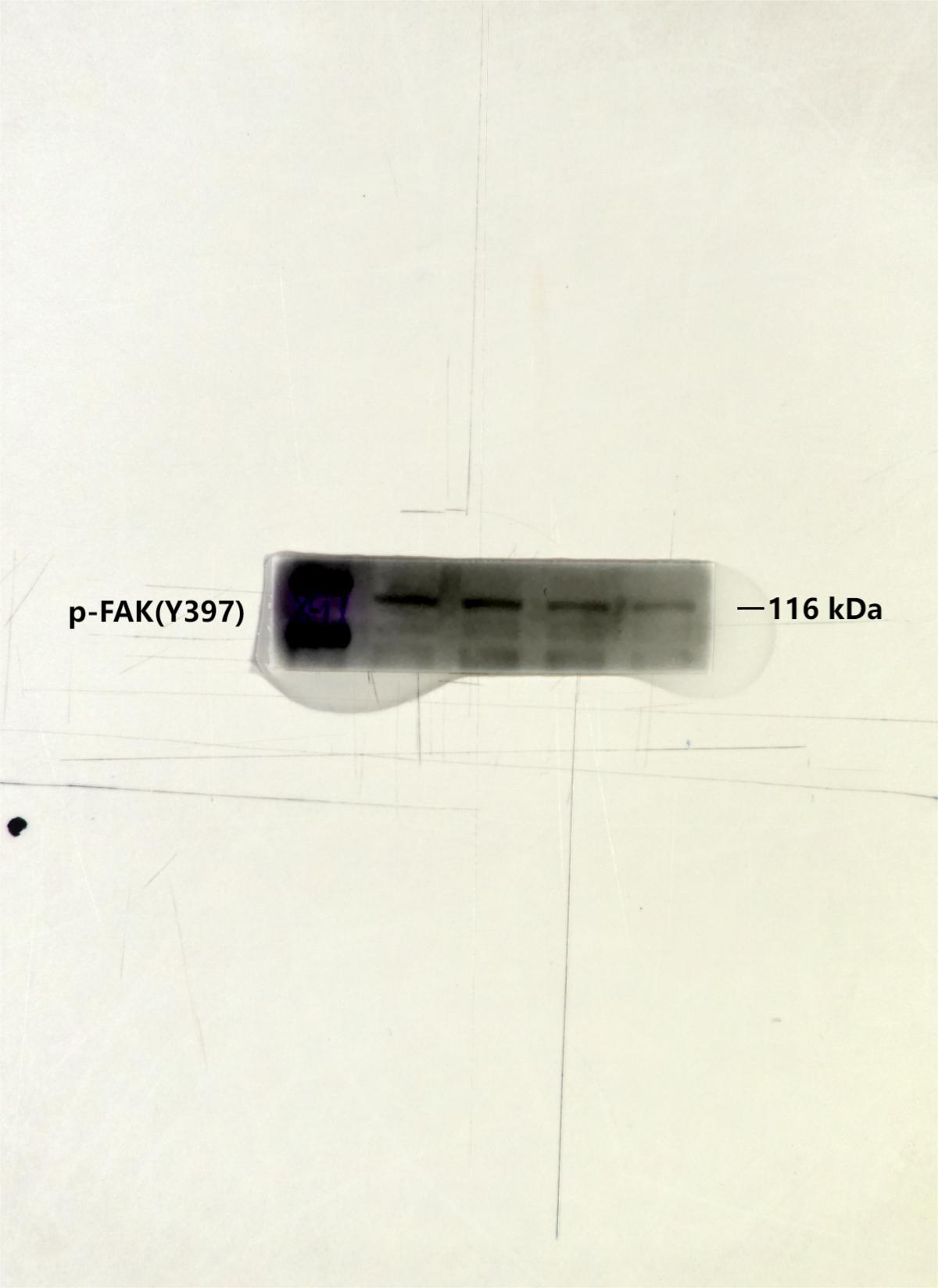

Supplement: Original Image for Fig 5B_p_FAK Y397.tif [file IENZ_A_2423875_SM5335.tif]

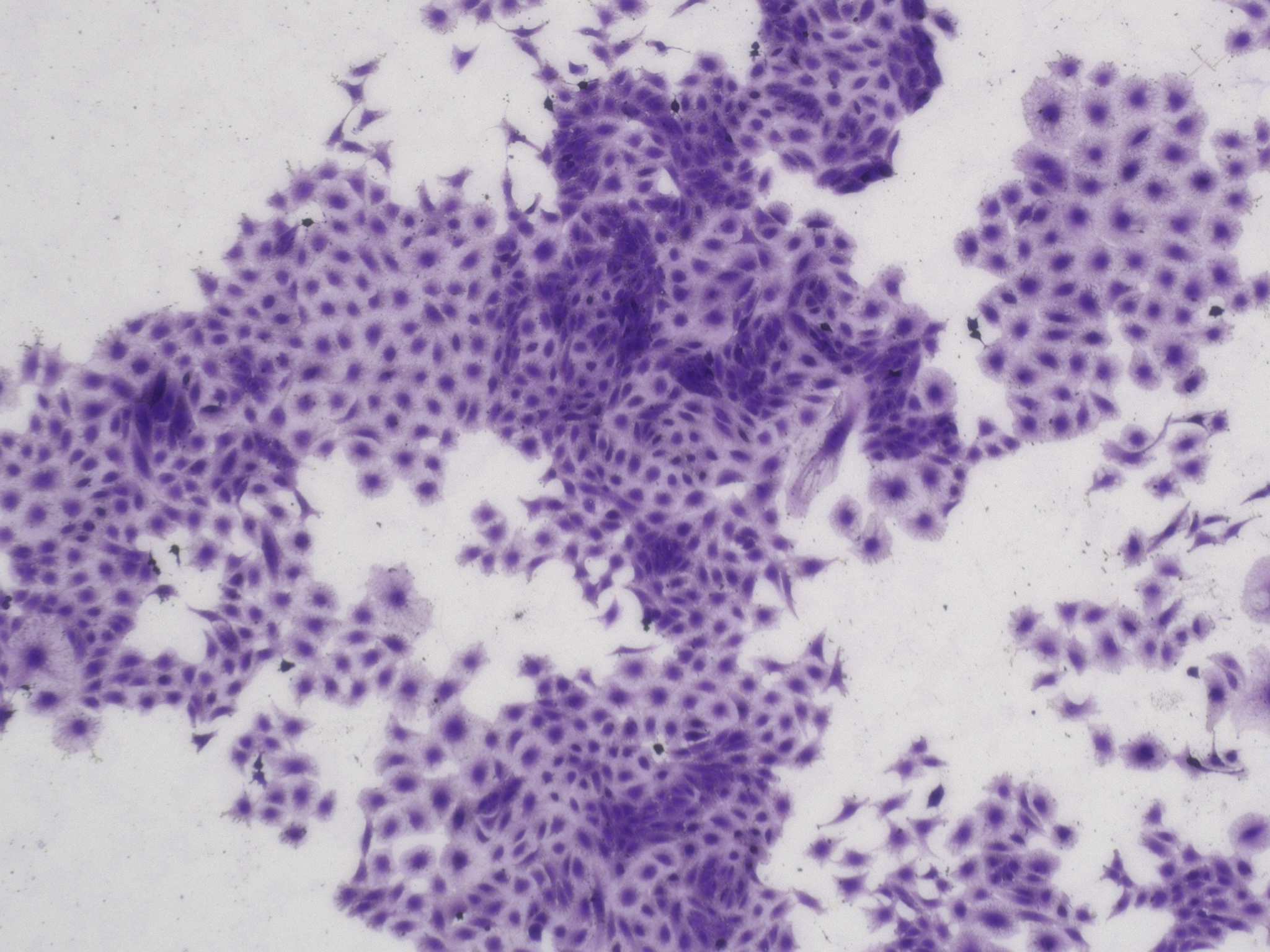

Supplement: Original Image for Figure 6B 500 nM.TIF [file IENZ_A_2423875_SM5334.tif]

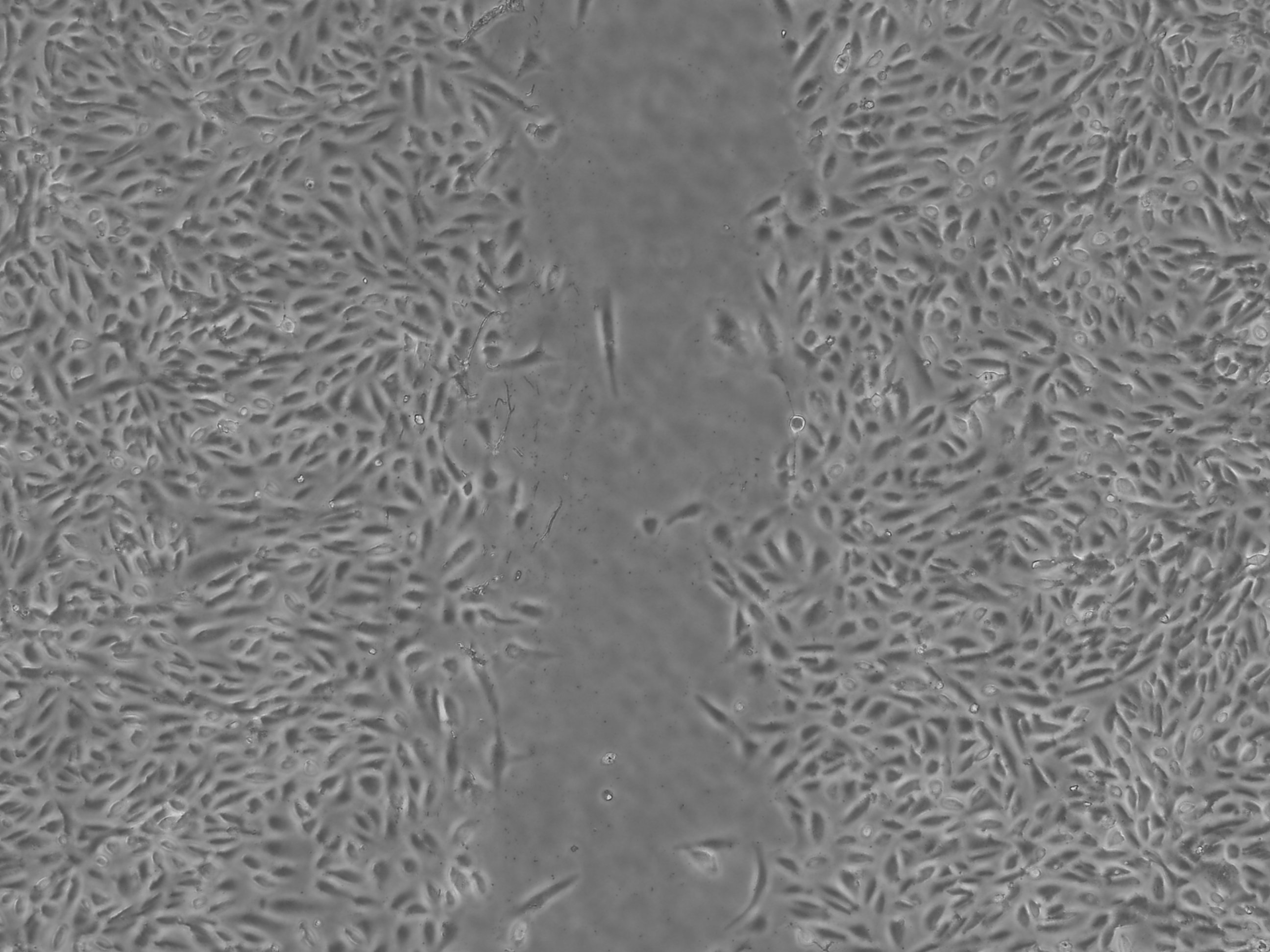

Supplement: Original Image for Figure 7A 48h 600 nM_1.tif [file IENZ_A_2423875_SM5333.tif]

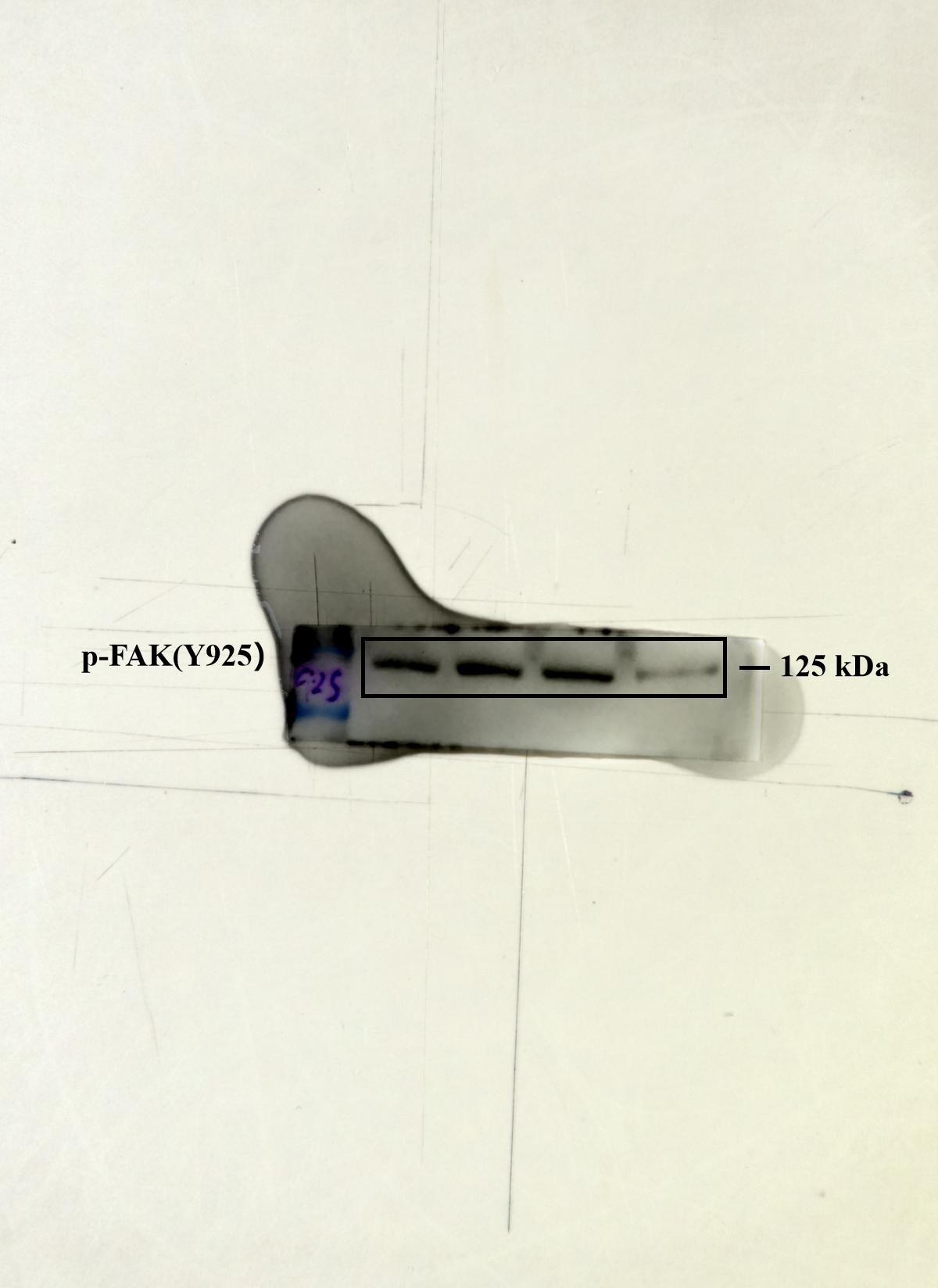

Supplement: Original Image for Fig 5B_p_FAK Y925.tif [file IENZ_A_2423875_SM5332.tif]

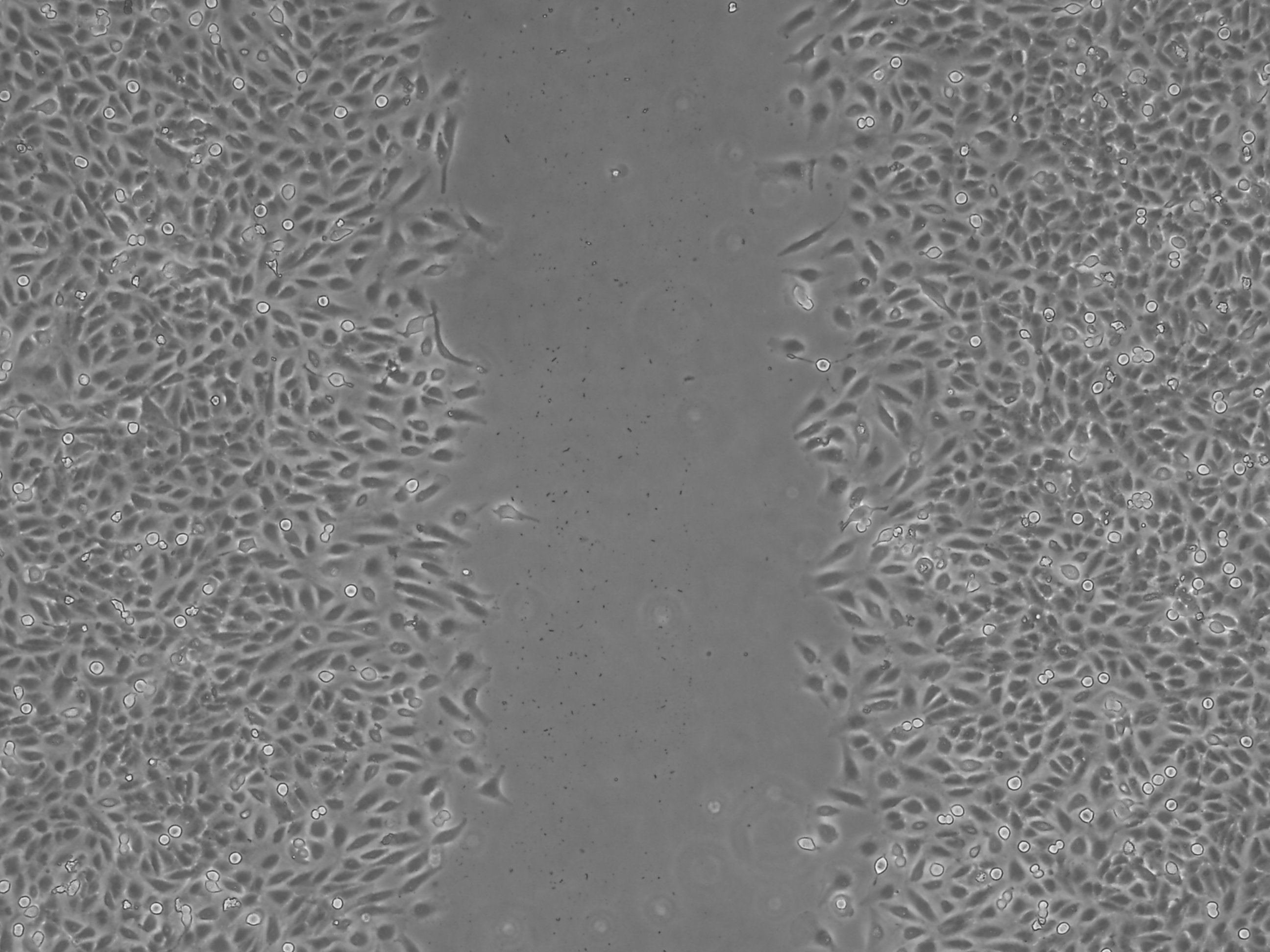

Supplement: Original Image for Figure 7A 12h 300 nM_1.tif [file IENZ_A_2423875_SM5331.tif]

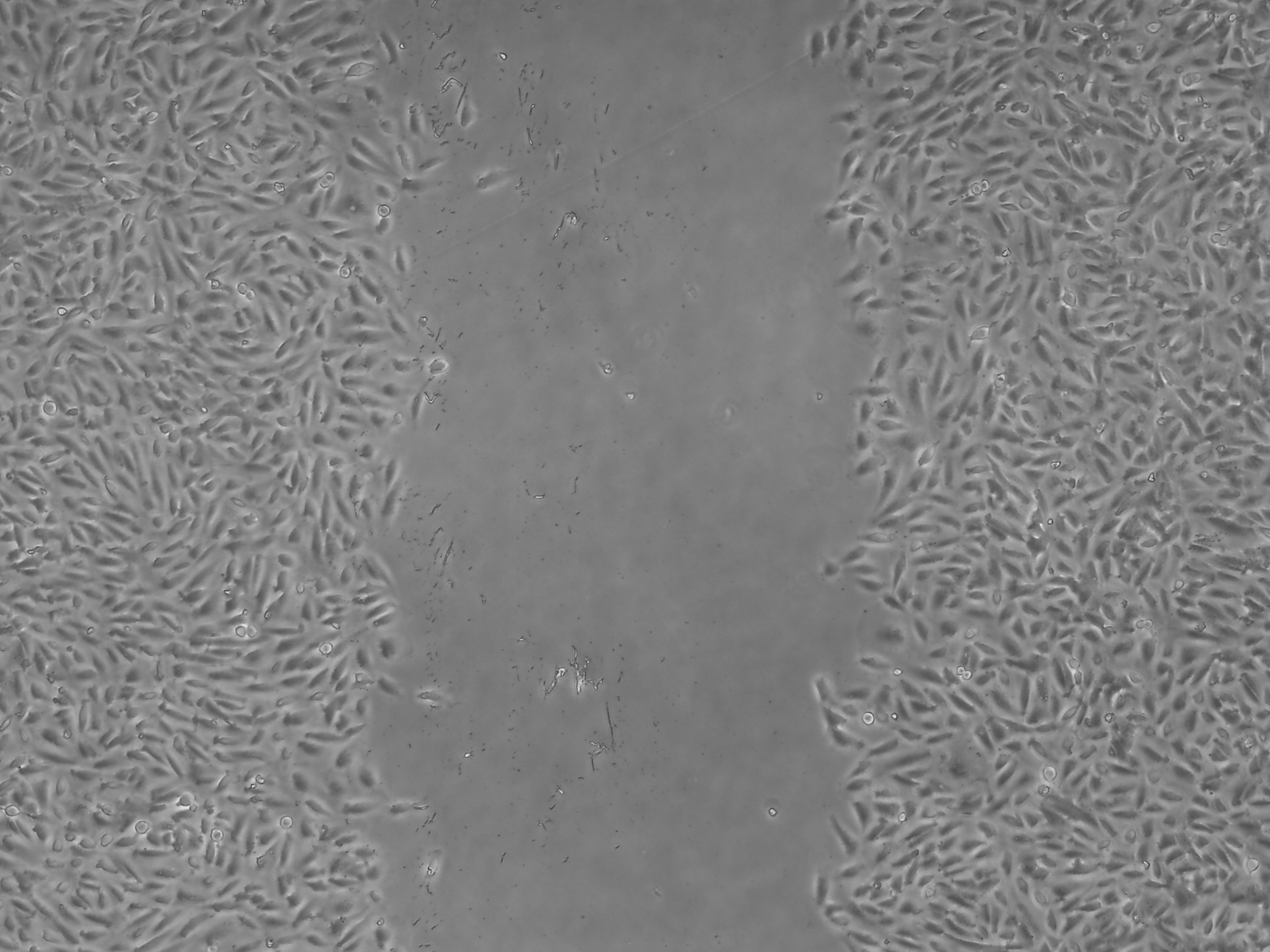

Supplement: Original Image for Figure 7A 24h 900 nM_1.tif [file IENZ_A_2423875_SM5330.tif]

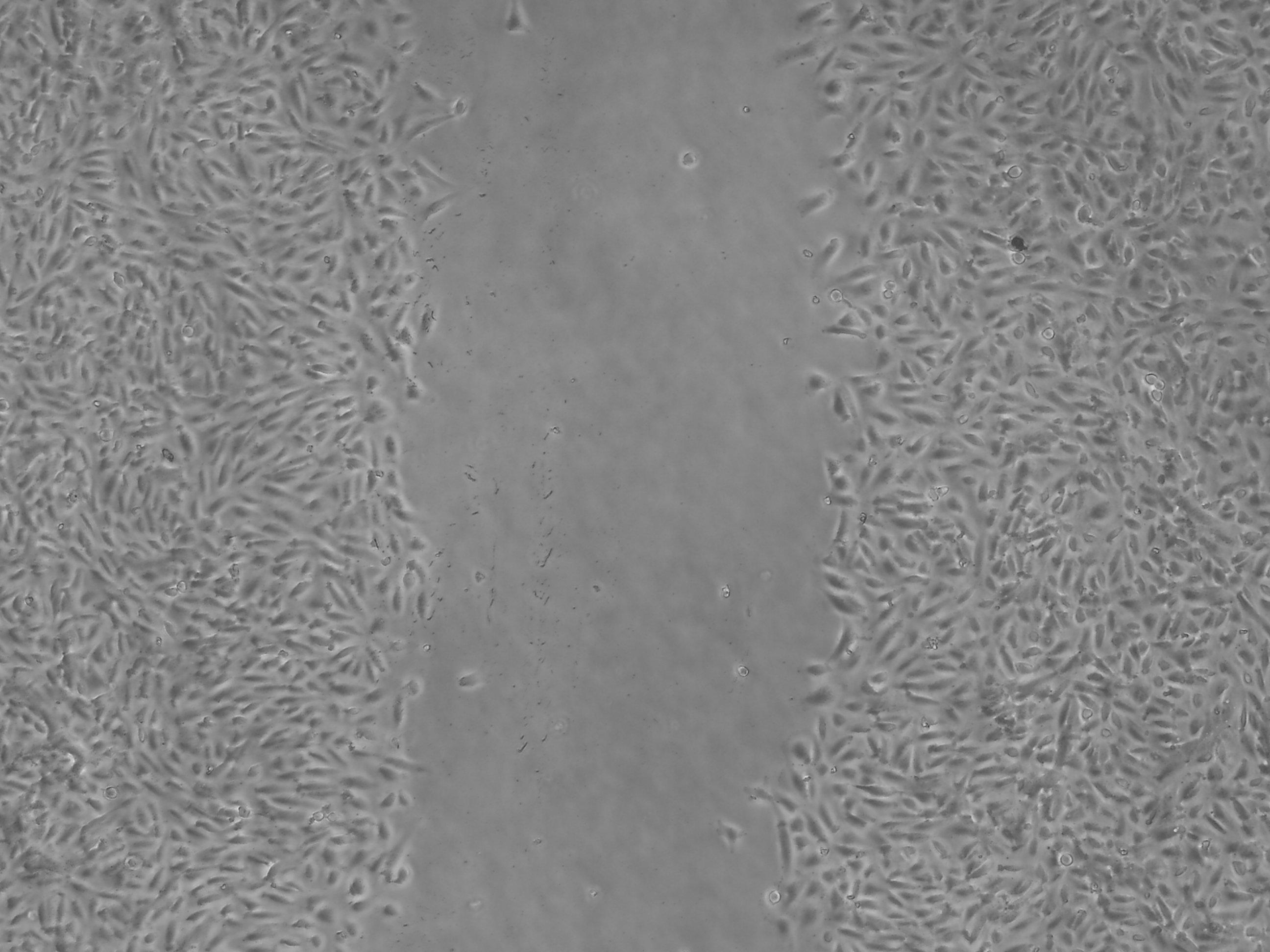

Supplement: Original Image for Figure 7A 24h 600 nM_2.tif [file IENZ_A_2423875_SM5329.tif]

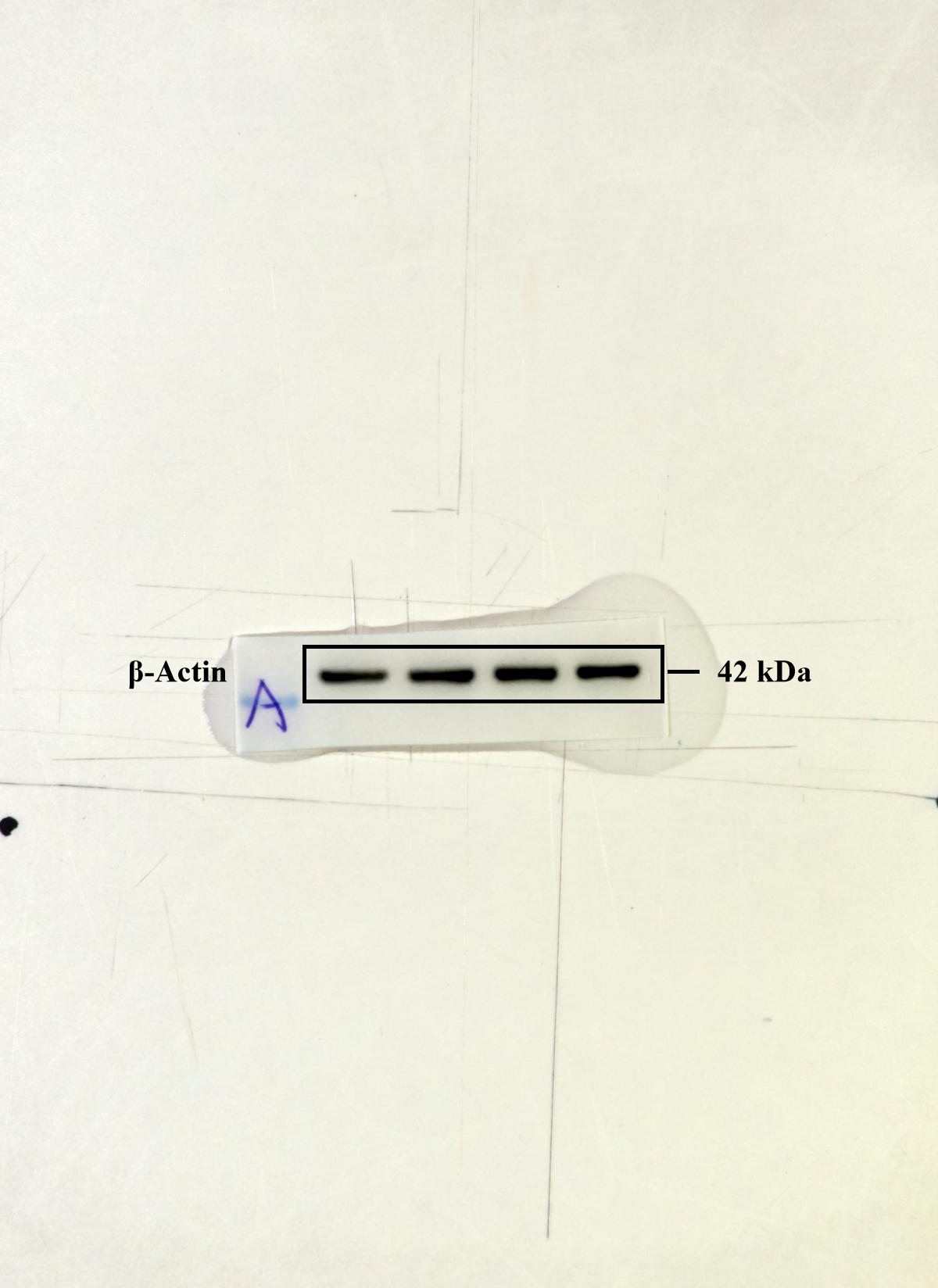

Supplement: Original Image for Fig 5B_β_actin.tif [file IENZ_A_2423875_SM5328.tif]

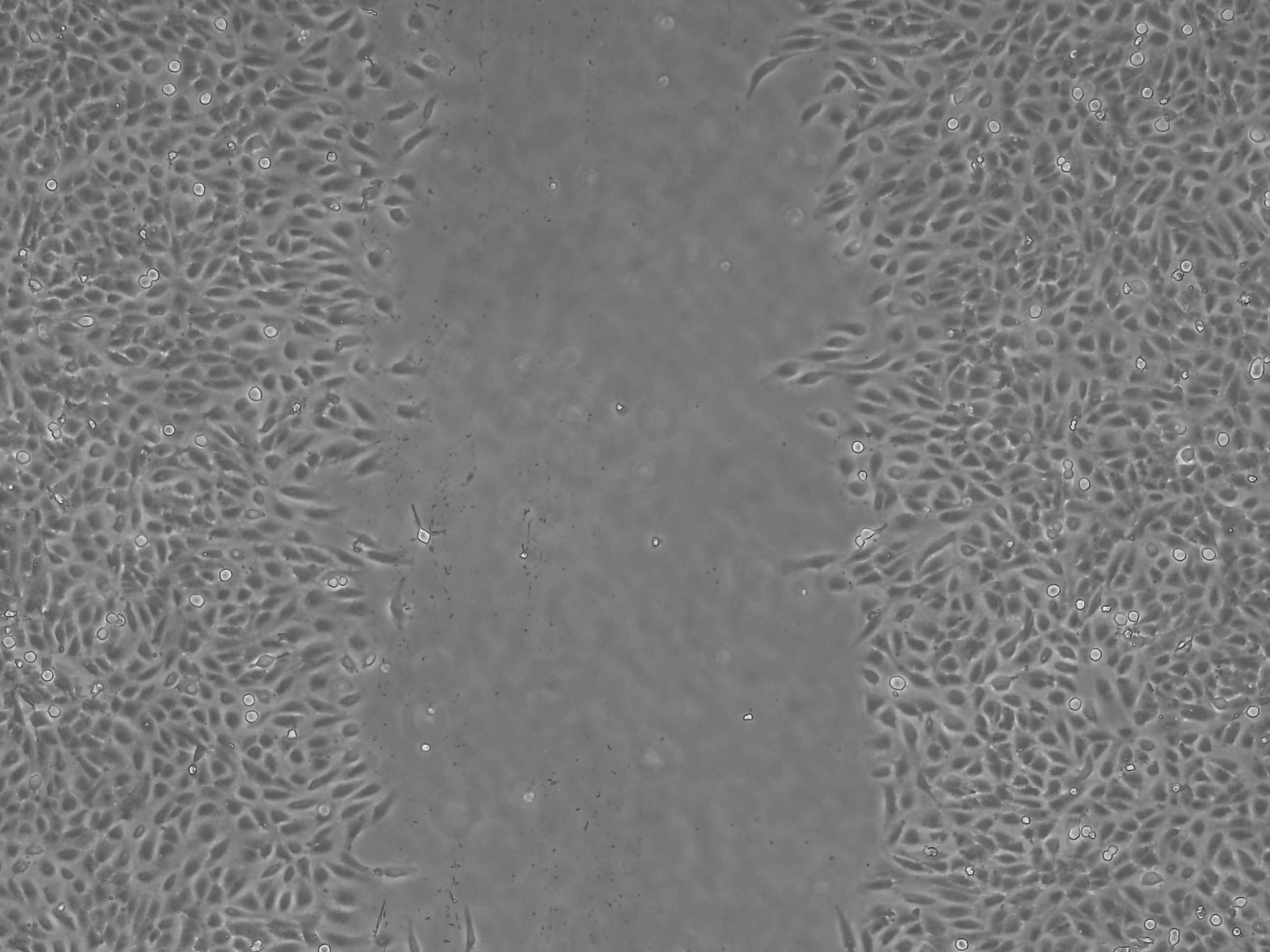

Supplement: Original Image for Figure 7A 12h 600 nM_1.tif [file IENZ_A_2423875_SM5327.tif]

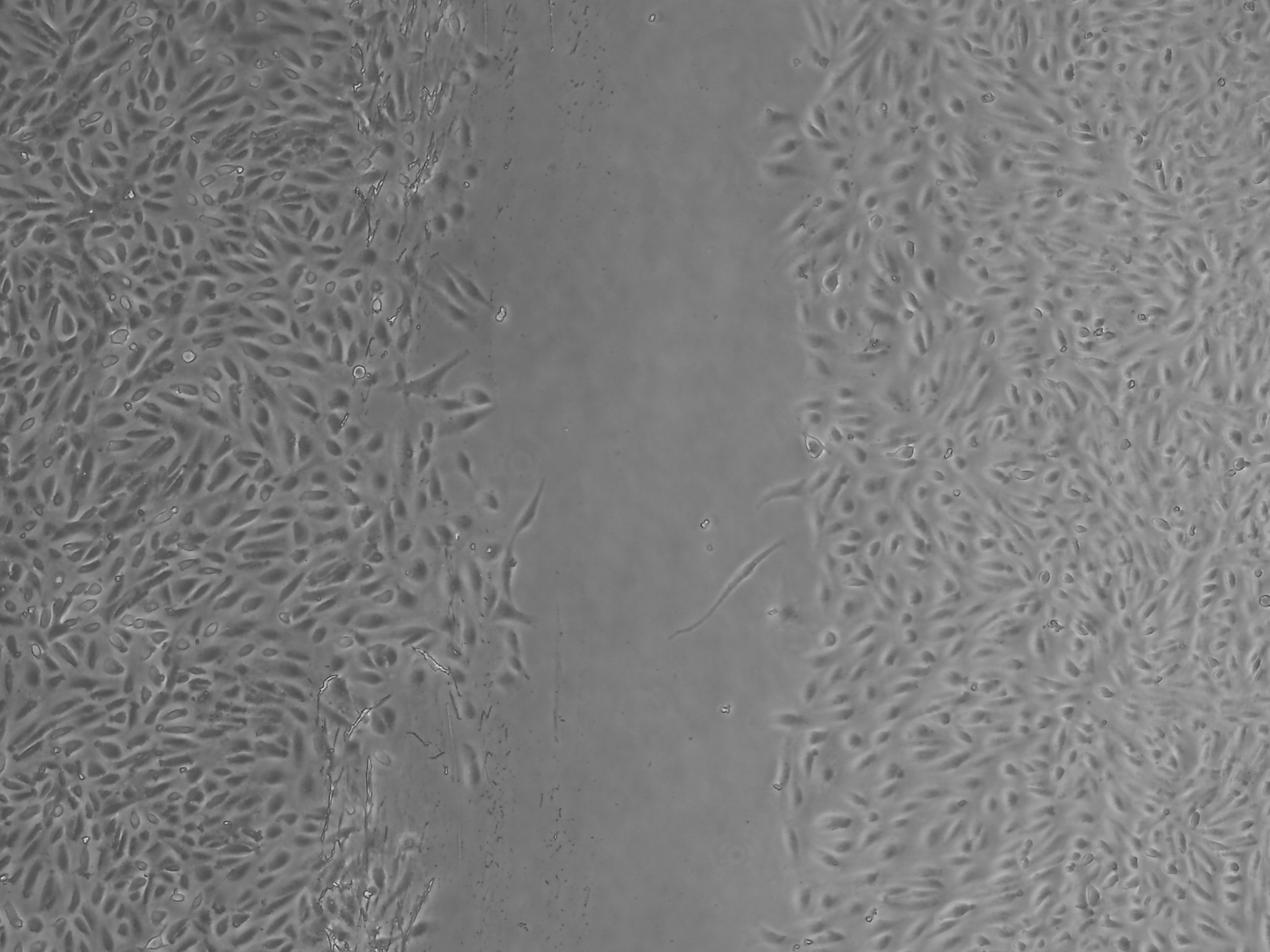

Supplement: Original Image for Figure 7A 36h 600 nM_1.tif [file IENZ_A_2423875_SM5326.tif]

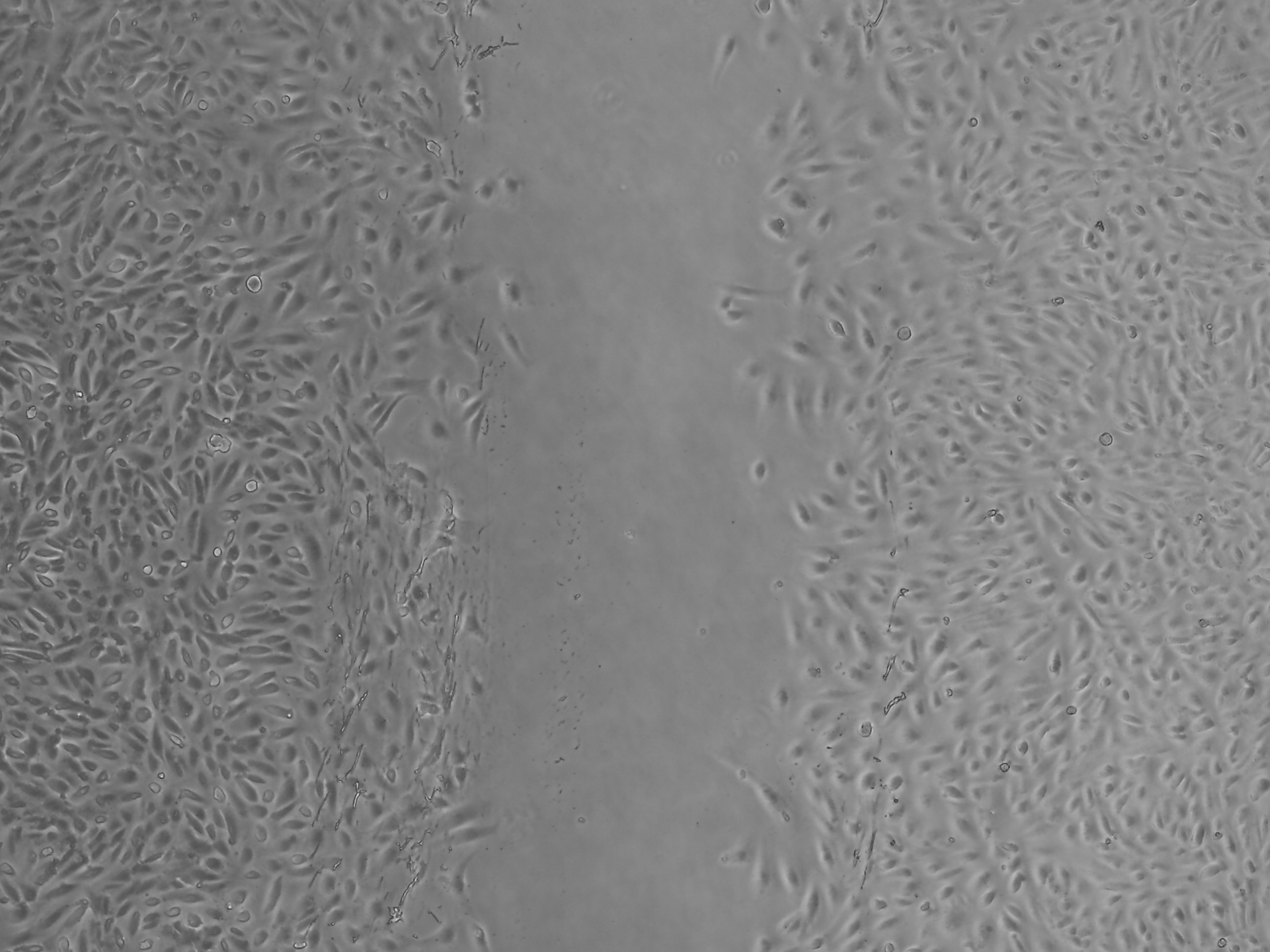

Supplement: Original Image for Figure 7A 36h 600 nM_2.tif [file IENZ_A_2423875_SM5325.tif]

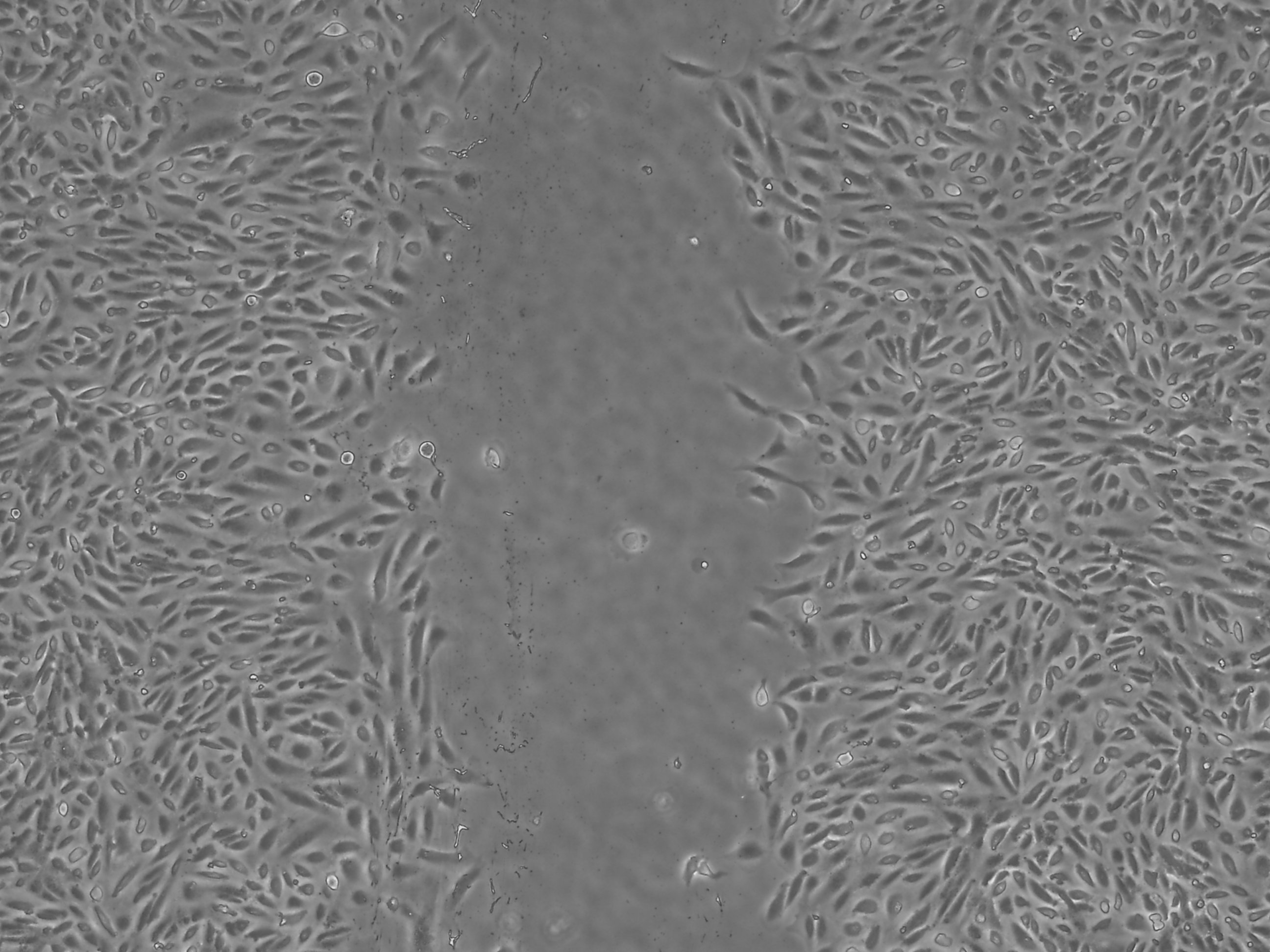

Supplement: Original Image for Figure 7A 36h 600 nM_3.tif [file IENZ_A_2423875_SM5324.tif]

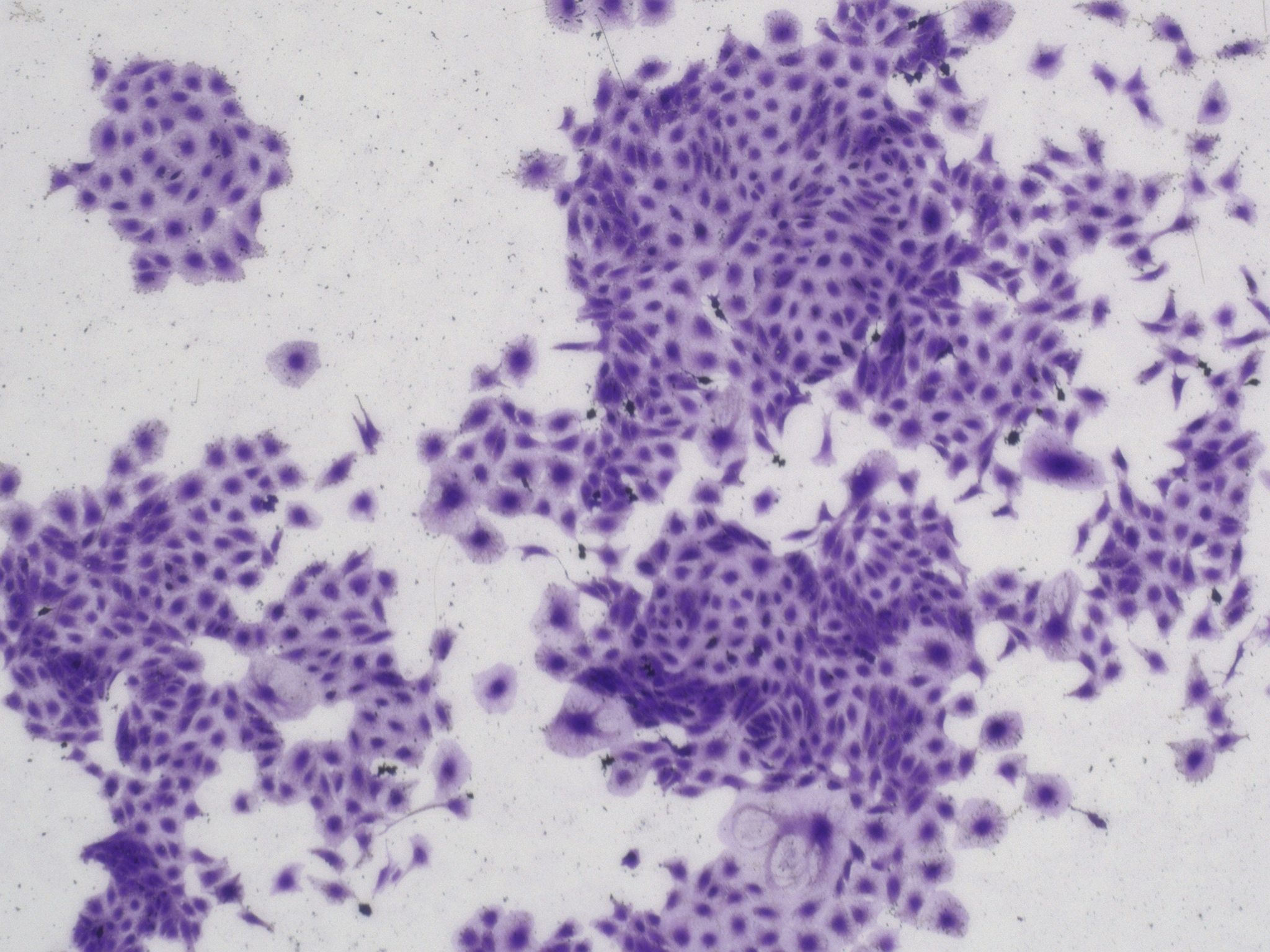

Supplement: Original Image for Figure 6B 700 nM.TIF [file IENZ_A_2423875_SM5323.tif]

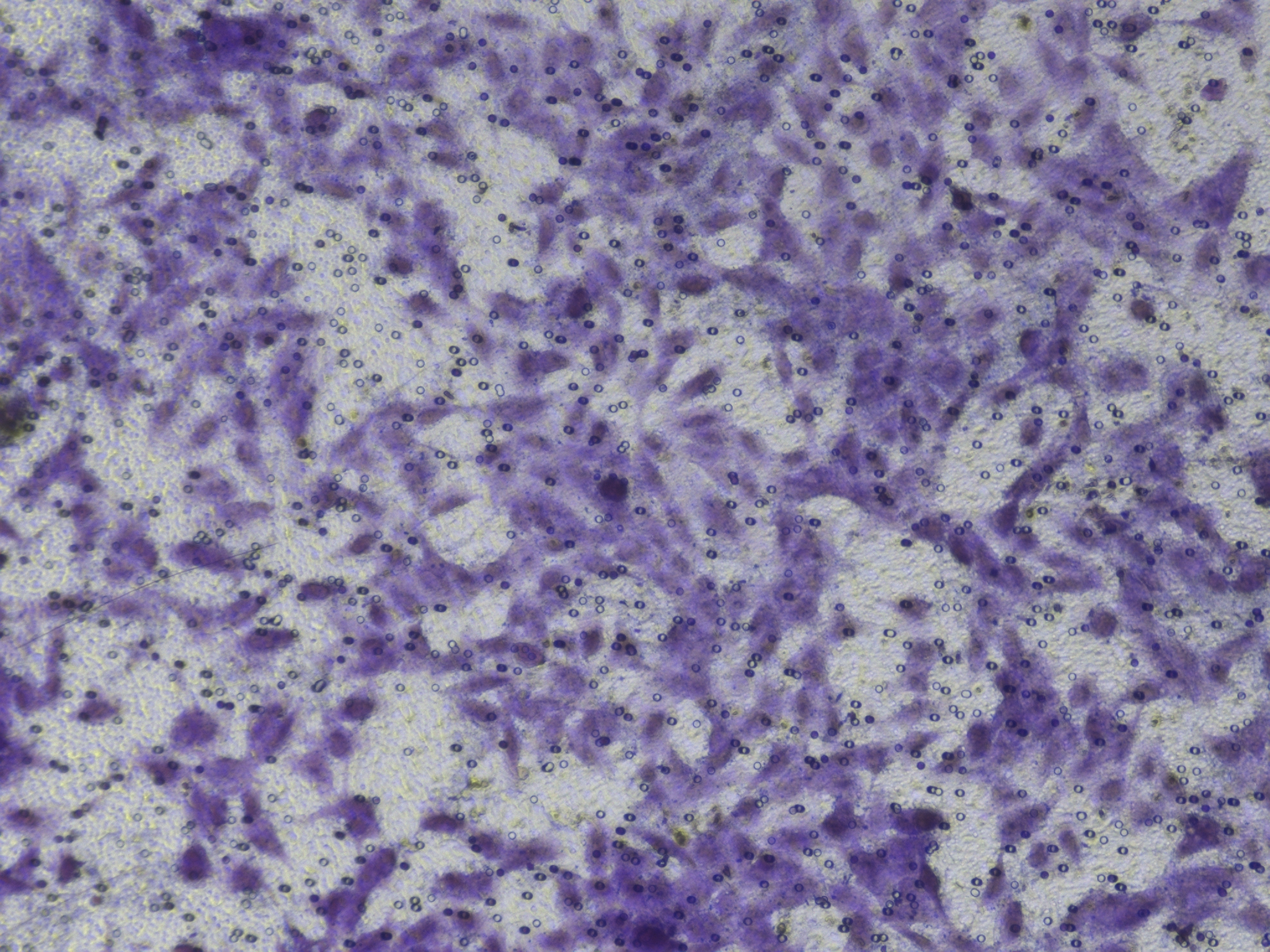

Supplement: Original Image for Figure 7C Control.TIF [file IENZ_A_2423875_SM5322.tif]

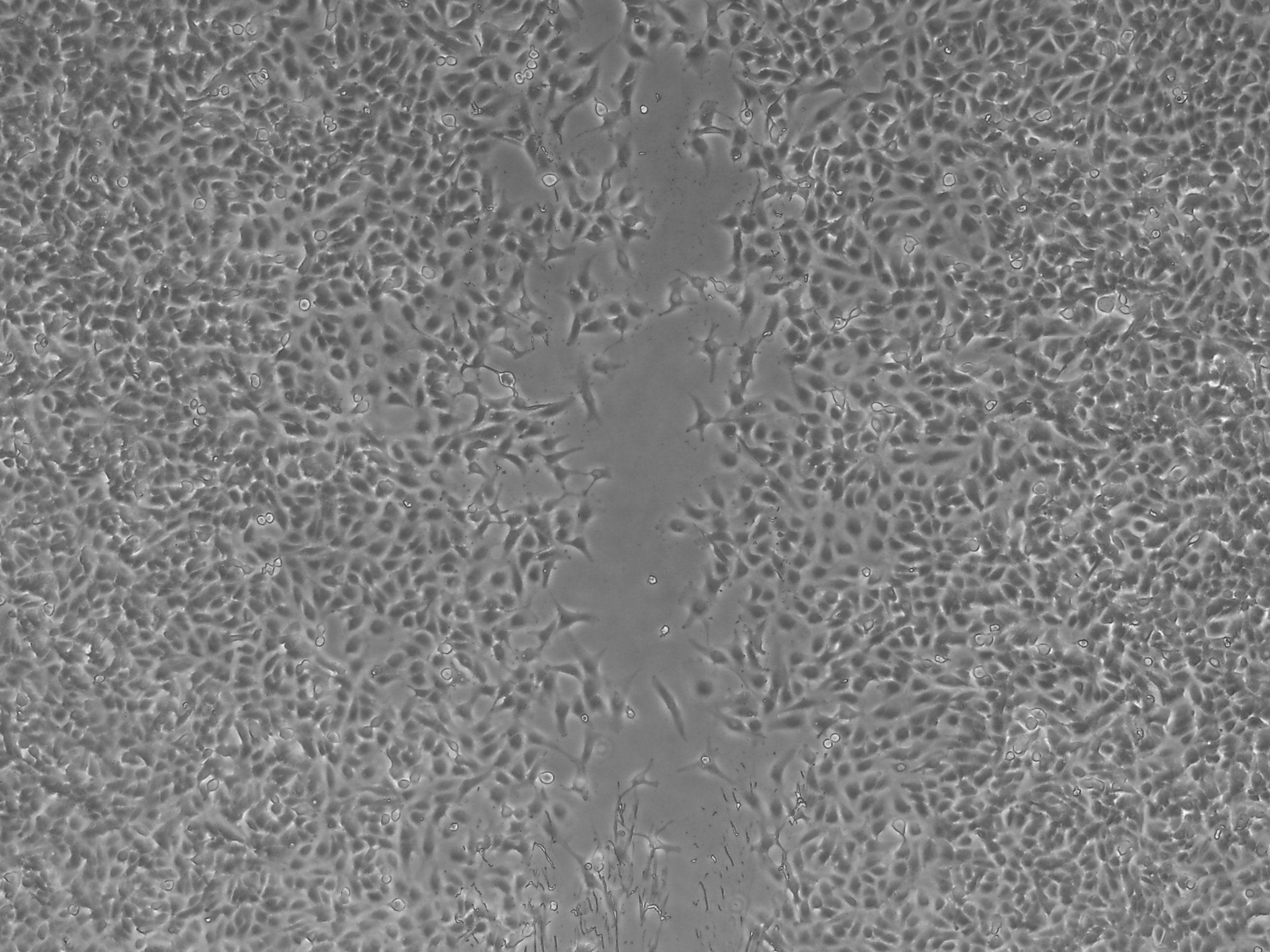

Supplement: Original Image for Figure 7A 36h Control_3.tif [file IENZ_A_2423875_SM5321.tif]

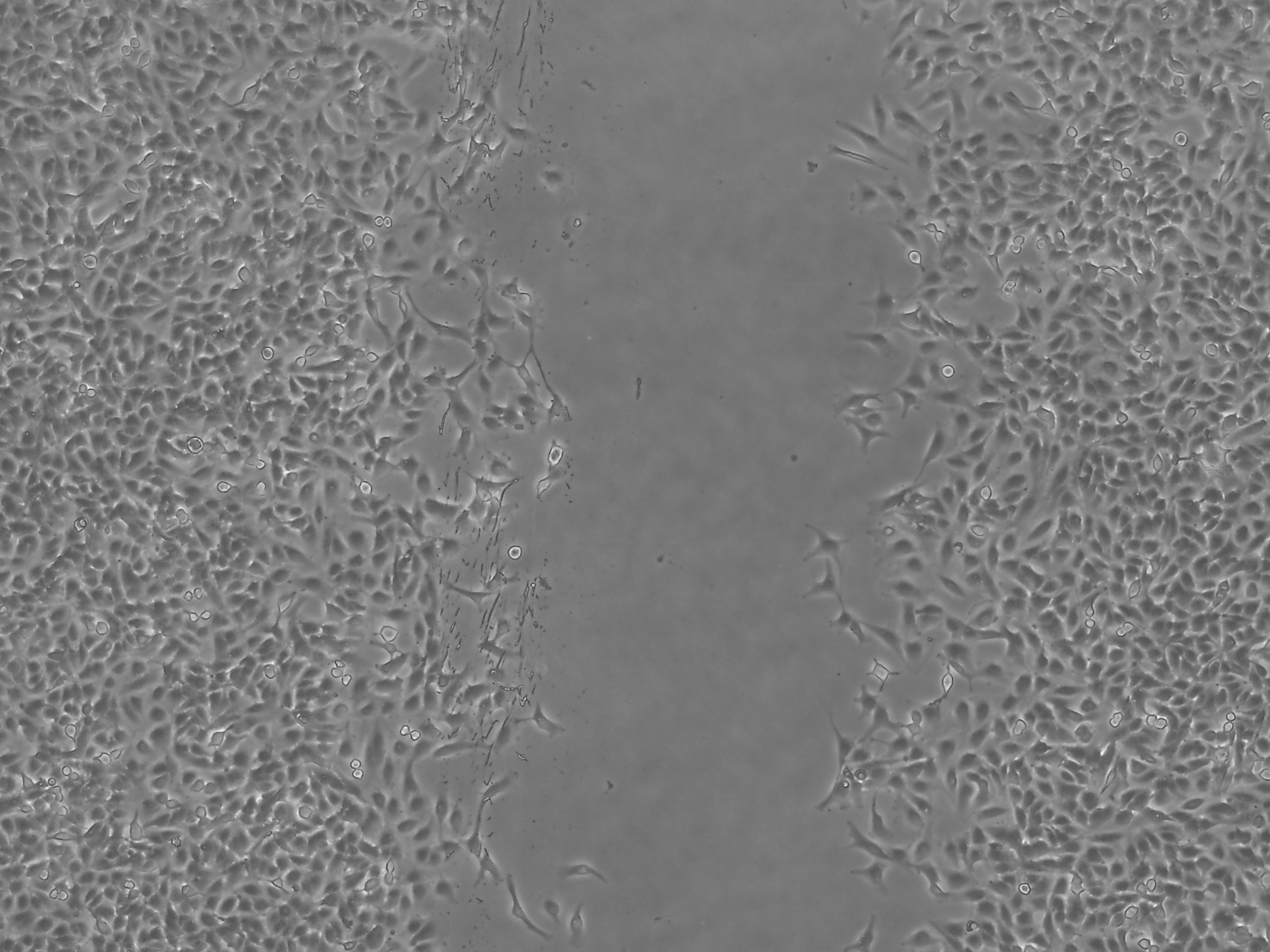

Supplement: Original Image for Figure 7A 12h Control_3.tif [file IENZ_A_2423875_SM5320.tif]

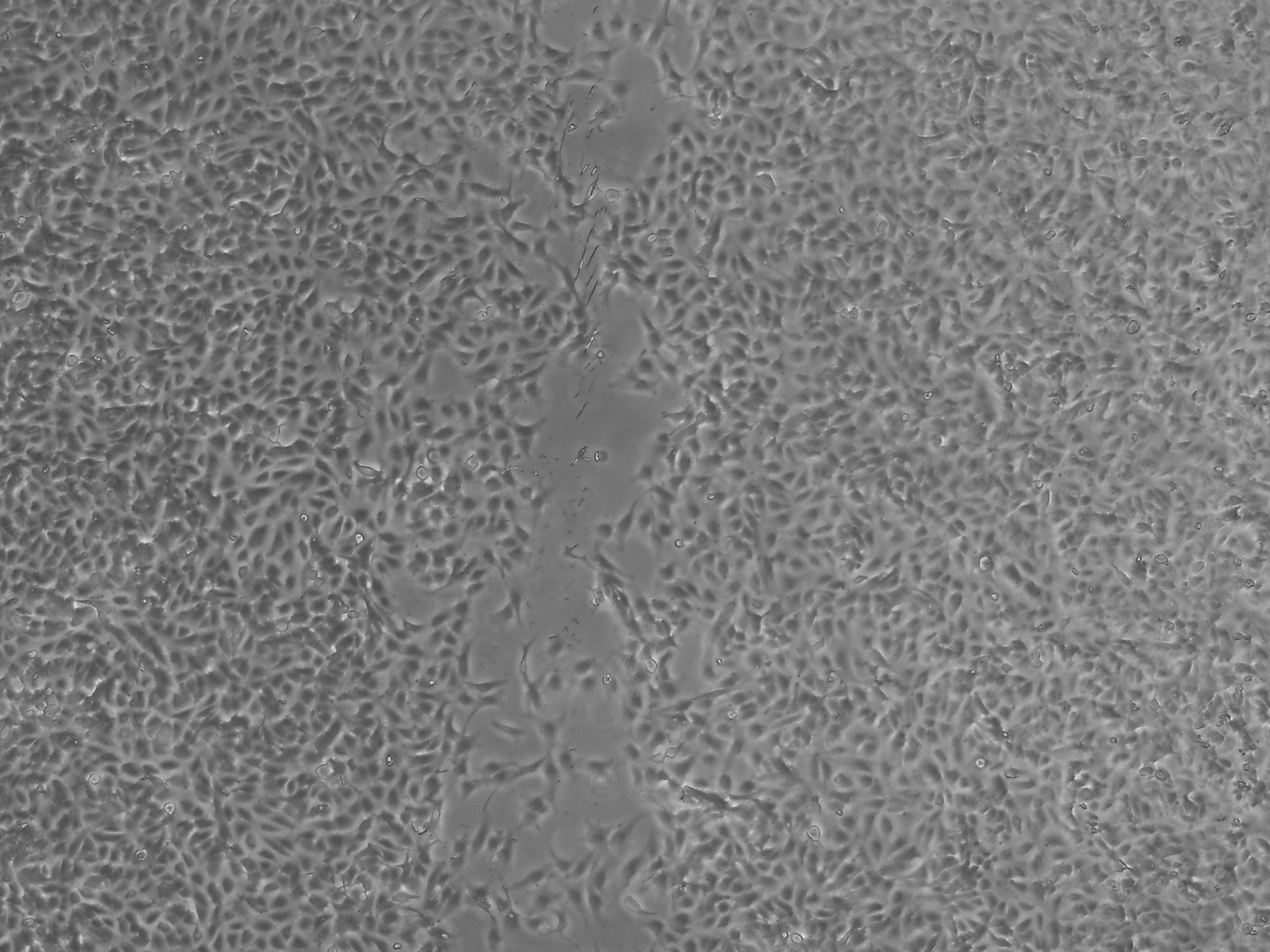

Supplement: Original Image for Figure 7A 48h Control_3.tif [file IENZ_A_2423875_SM5319.tif]

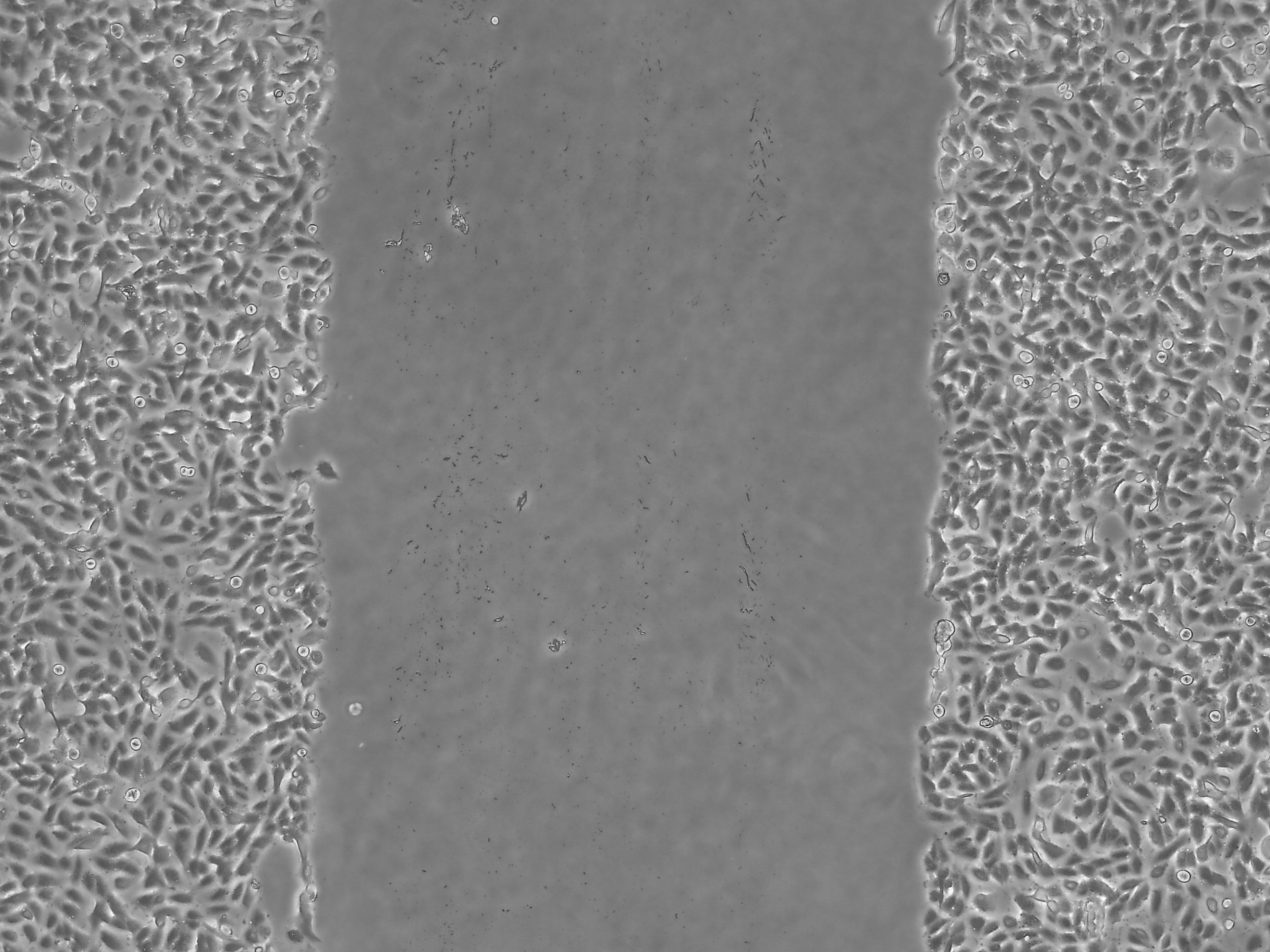

Supplement: Original Image for Figure 7A 0h Control_1.tif [file IENZ_A_2423875_SM5318.tif]

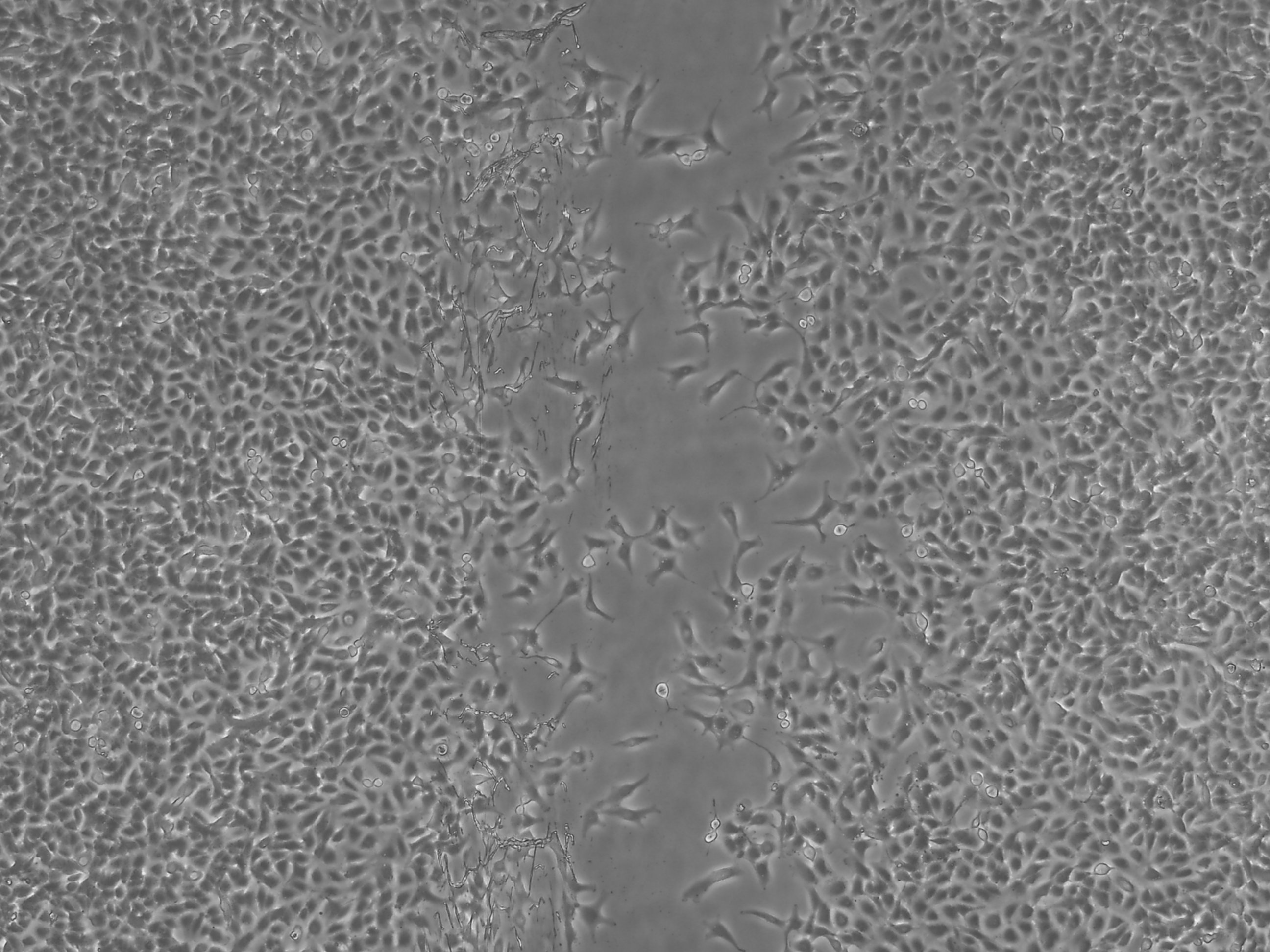

Supplement: Original Image for Figure 7A 36h Control_1.tif [file IENZ_A_2423875_SM5317.tif]

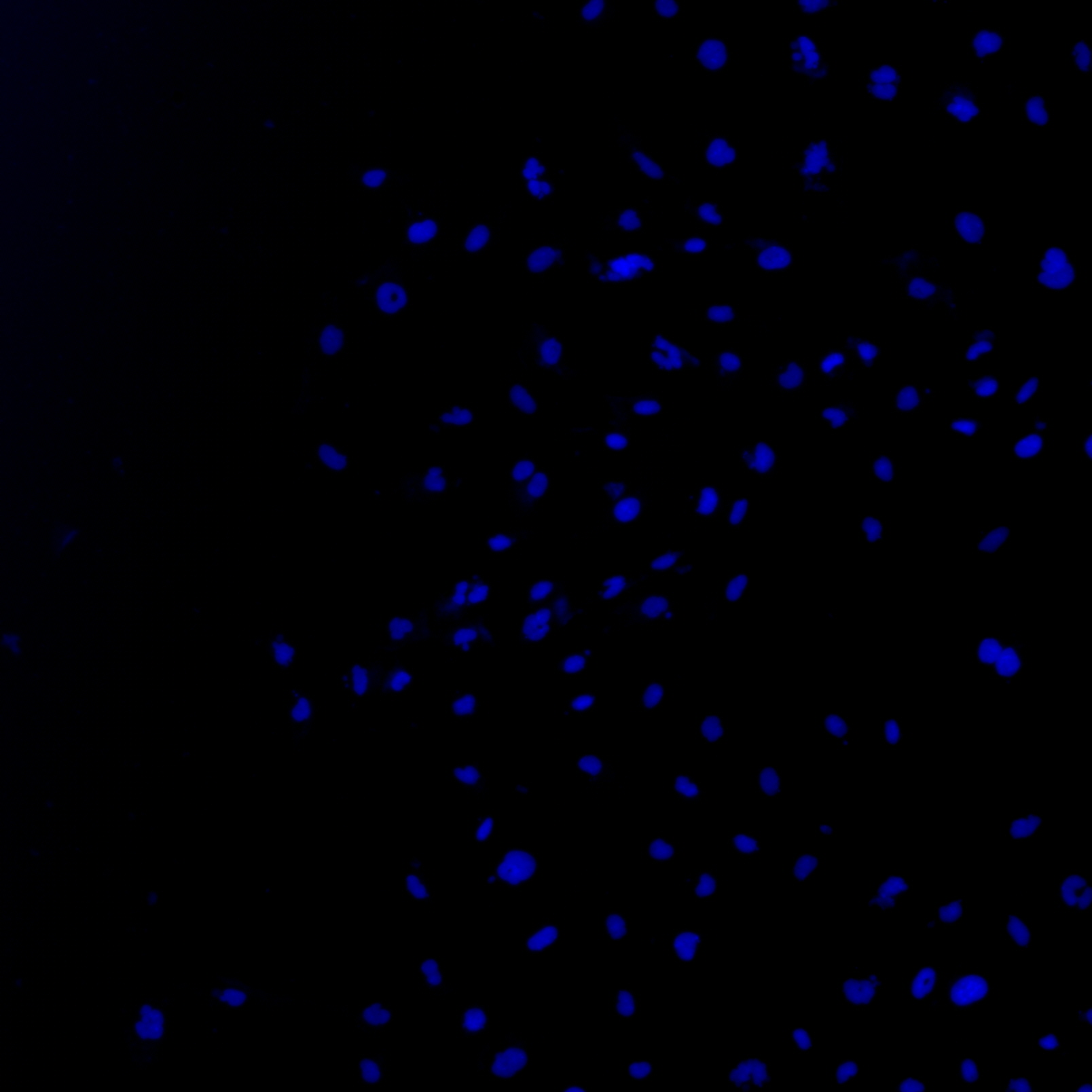

Supplement: Original Image for Figure 6A 600 nM_Hoechst.tif [file IENZ_A_2423875_SM5316.tif]

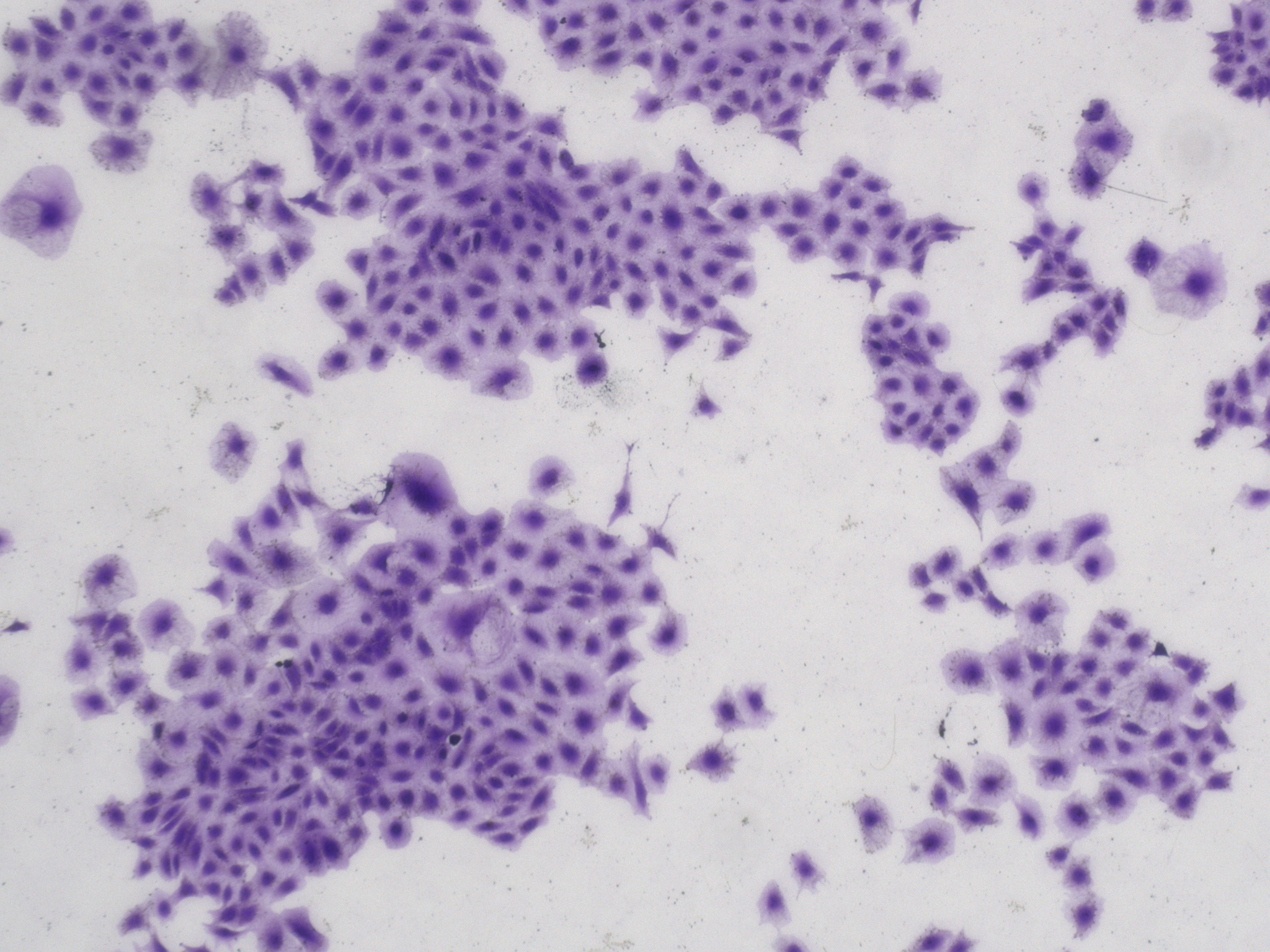

Supplement: Original Image for Figure 6B 900 nM.TIF [file IENZ_A_2423875_SM5315.tif]

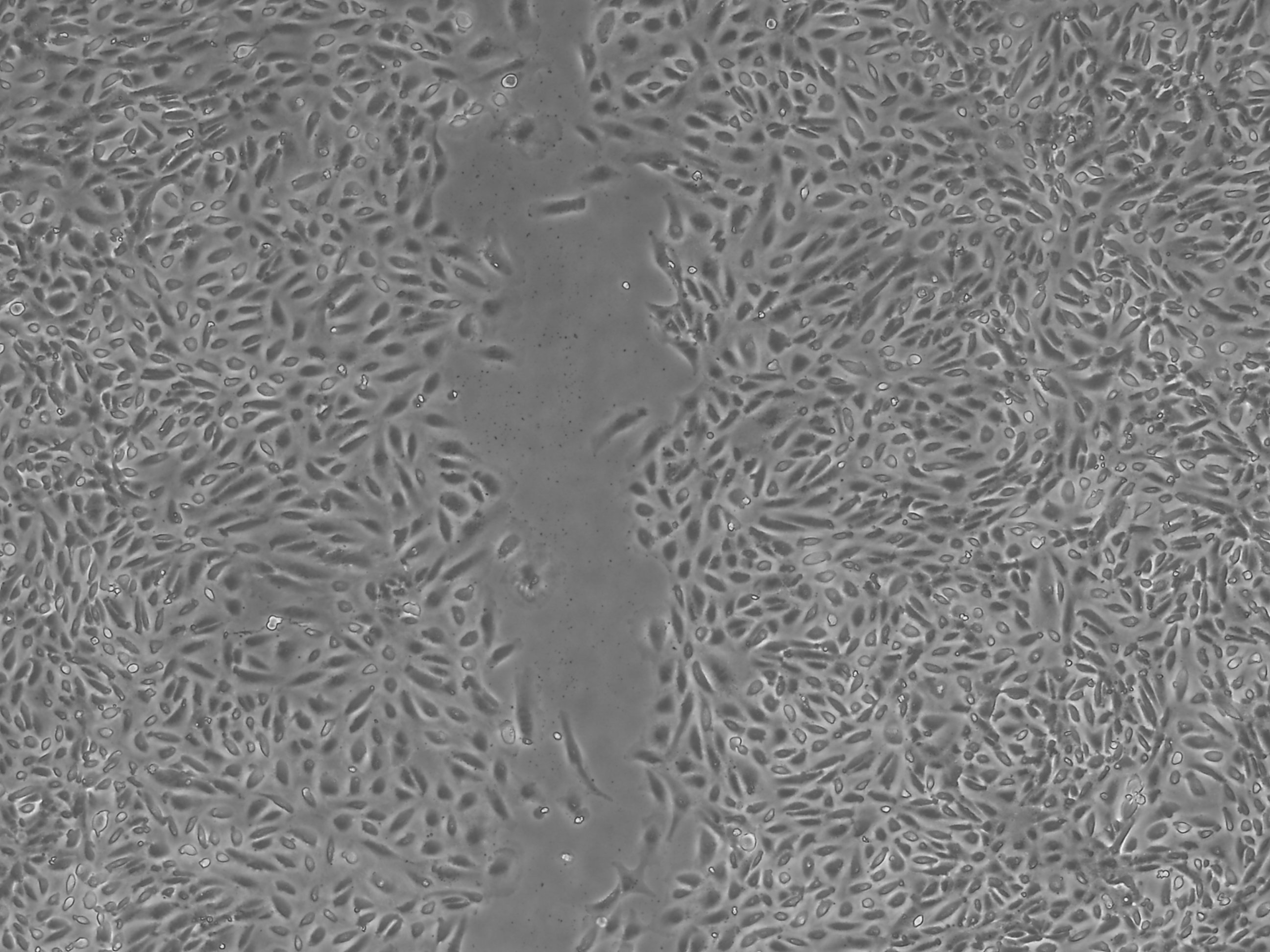

Supplement: Original Image for Figure 7A 48h 300 nM_1.tif [file IENZ_A_2423875_SM5314.tif]

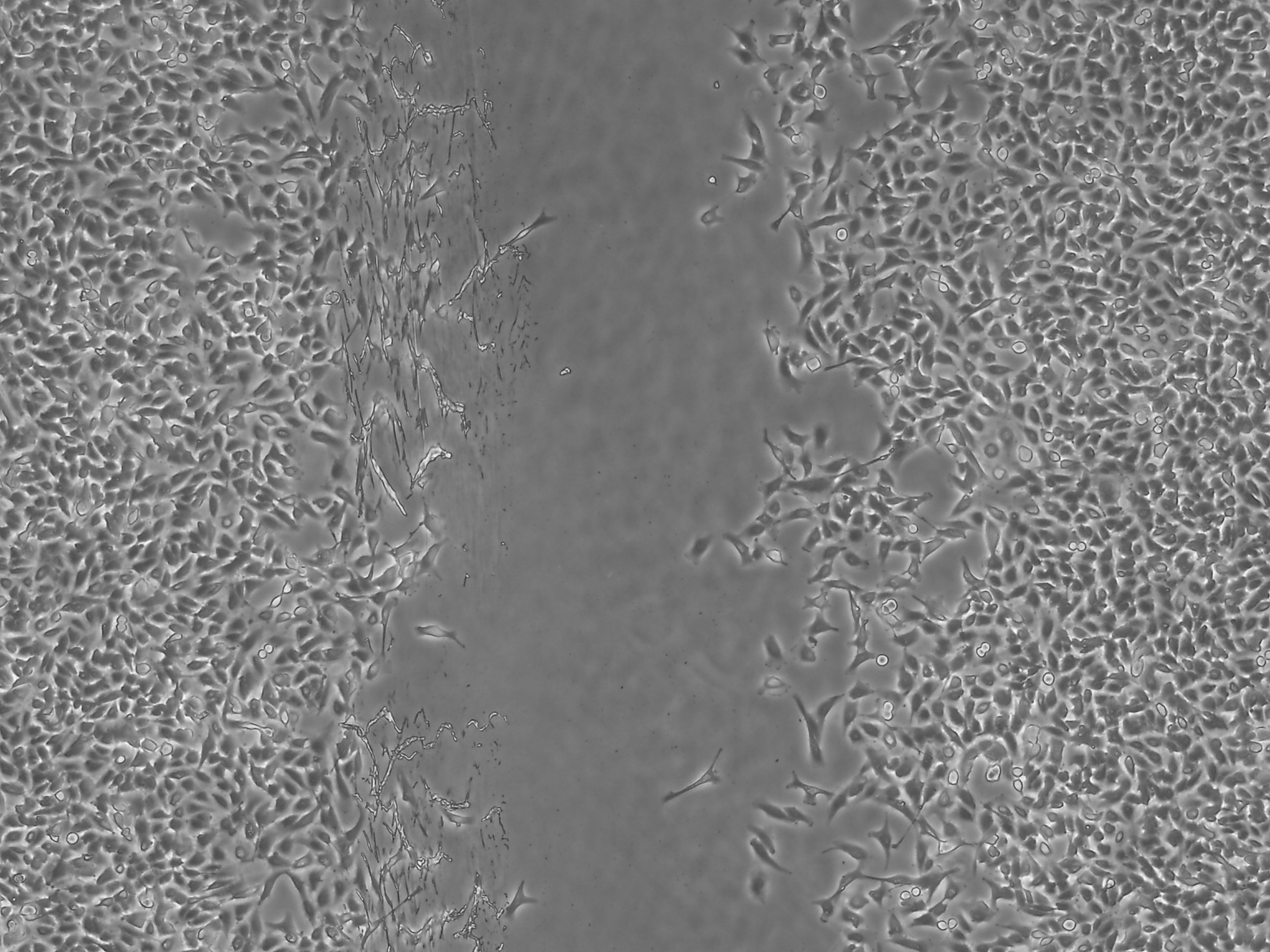

Supplement: Original Image for Figure 7A 24h Control_2.tif [file IENZ_A_2423875_SM5313.tif]

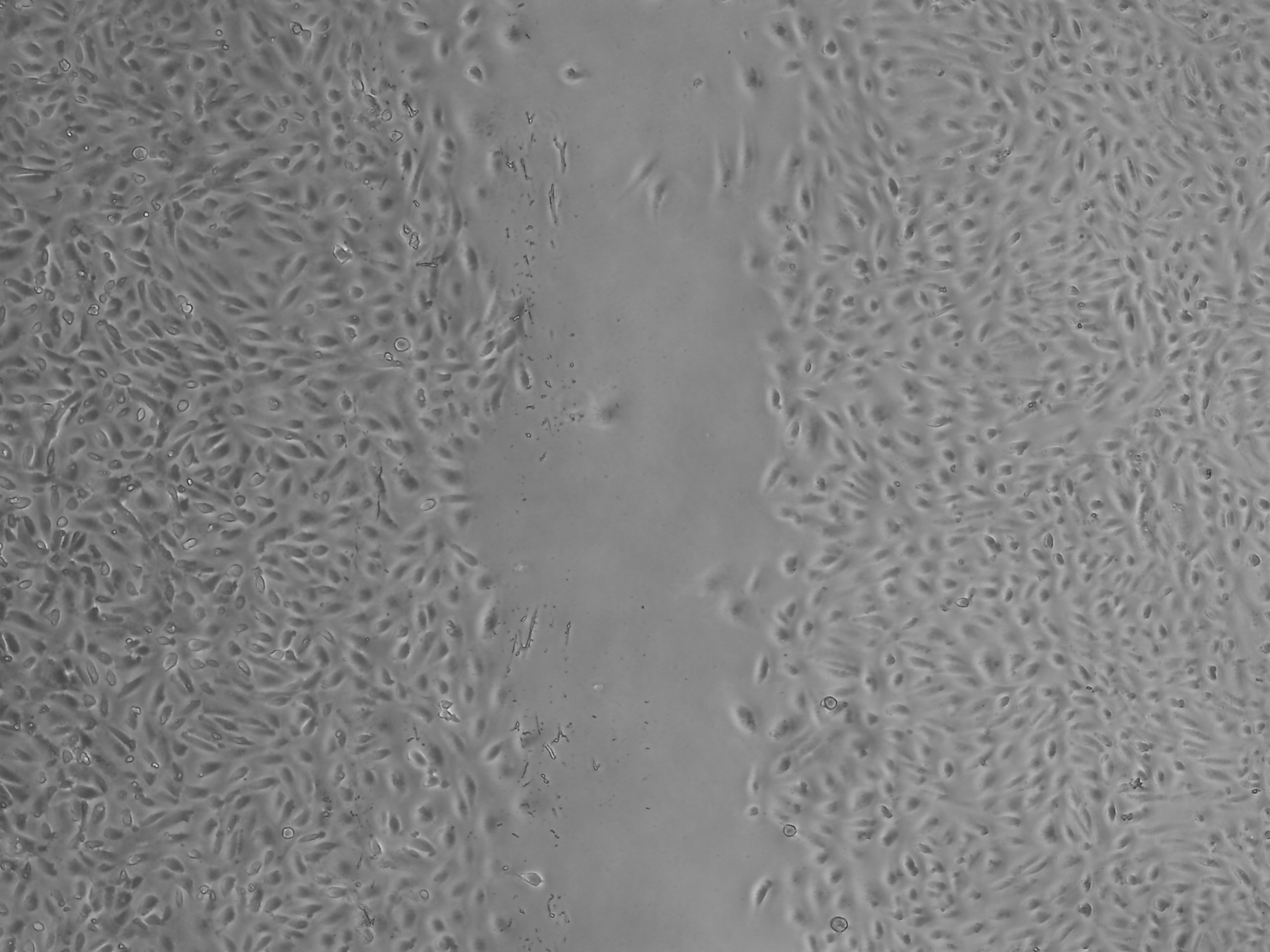

Supplement: Original Image for Figure 7A 48h 900 nM_2.tif [file IENZ_A_2423875_SM5312.tif]

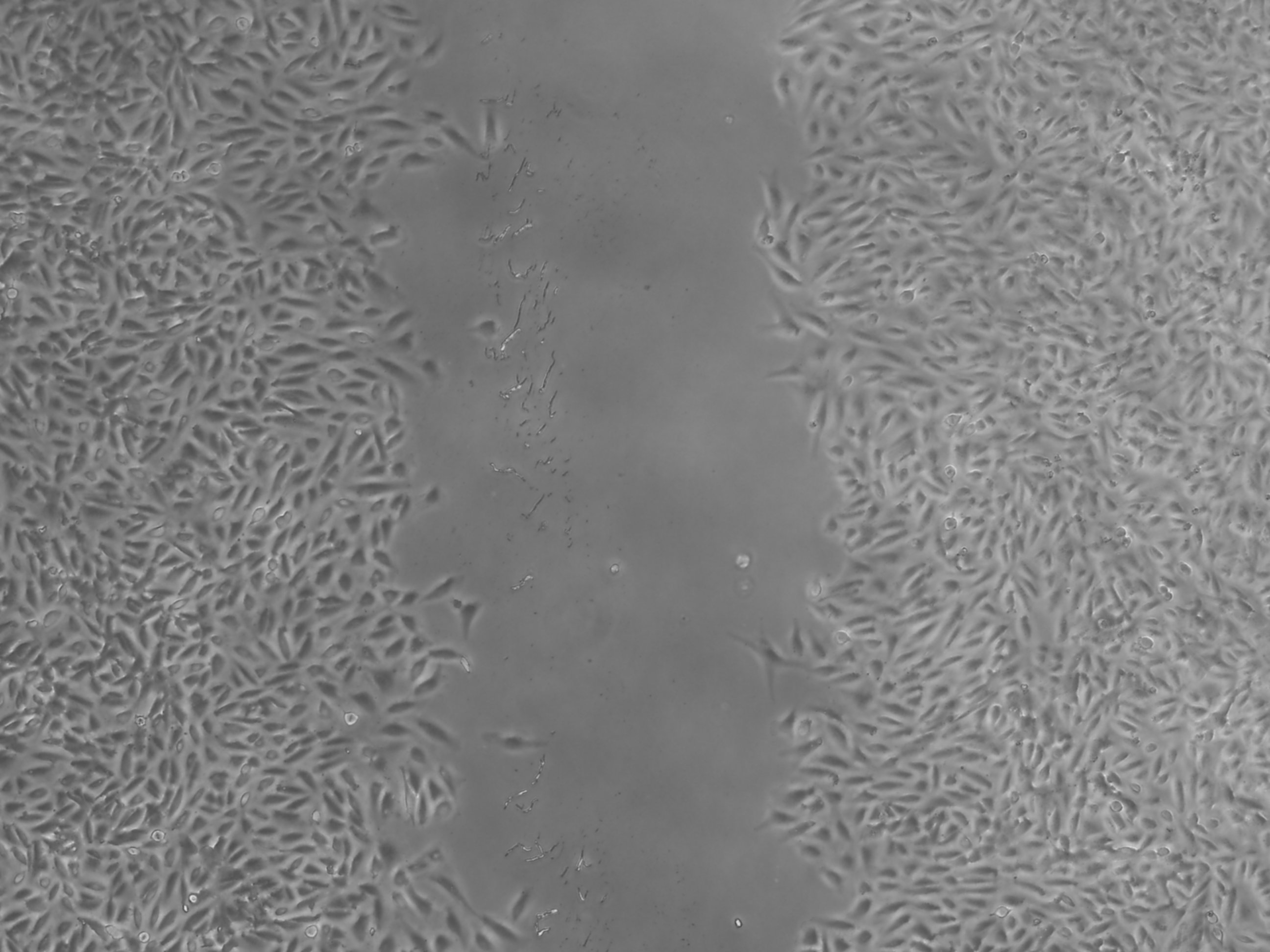

Supplement: Original Image for Figure 7A 24h 300 nM_2.tif [file IENZ_A_2423875_SM5311.tif]

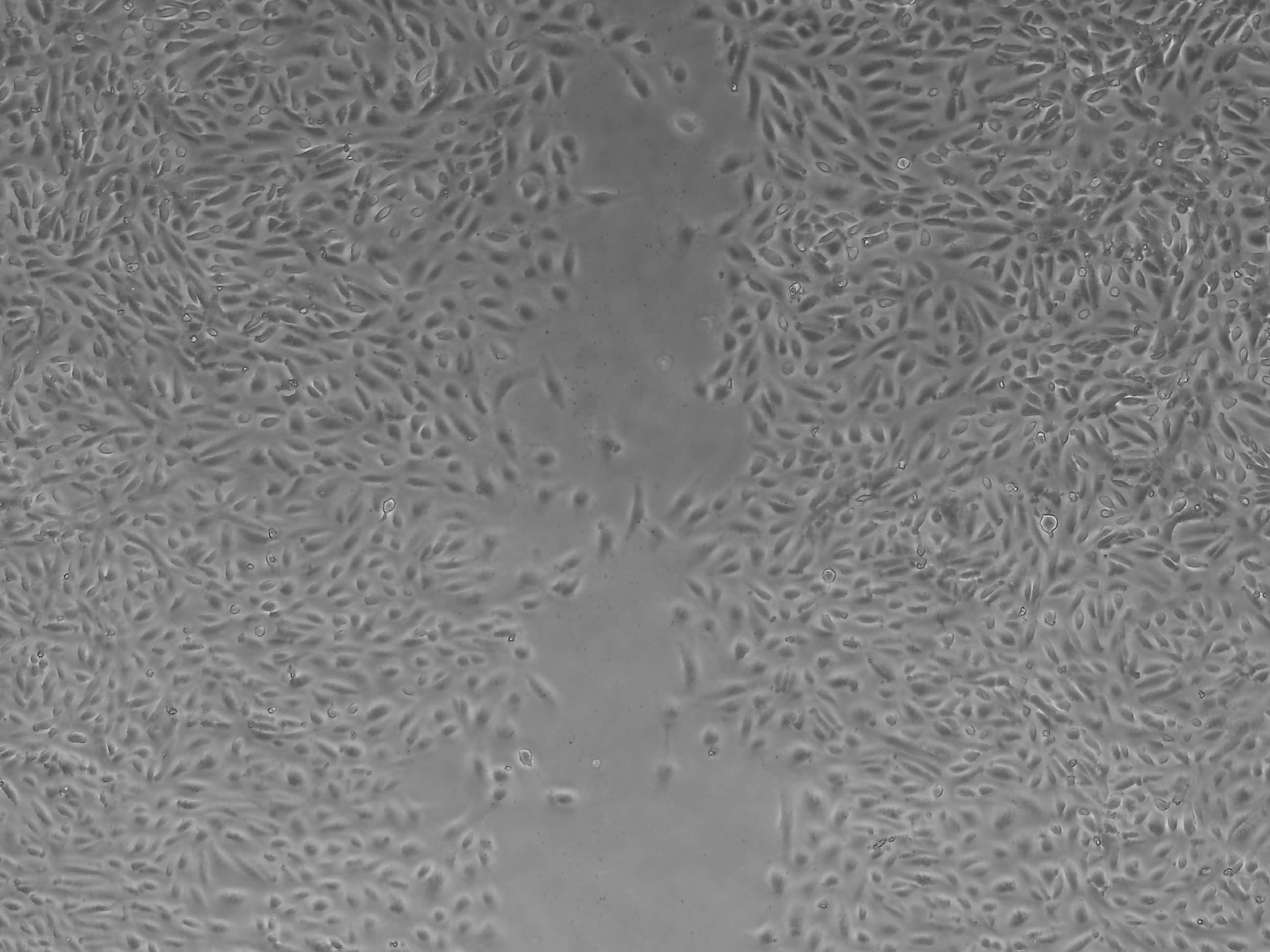

Supplement: Original Image for Figure 7A 48h 300 nM_3.tif [file IENZ_A_2423875_SM5310.tif]

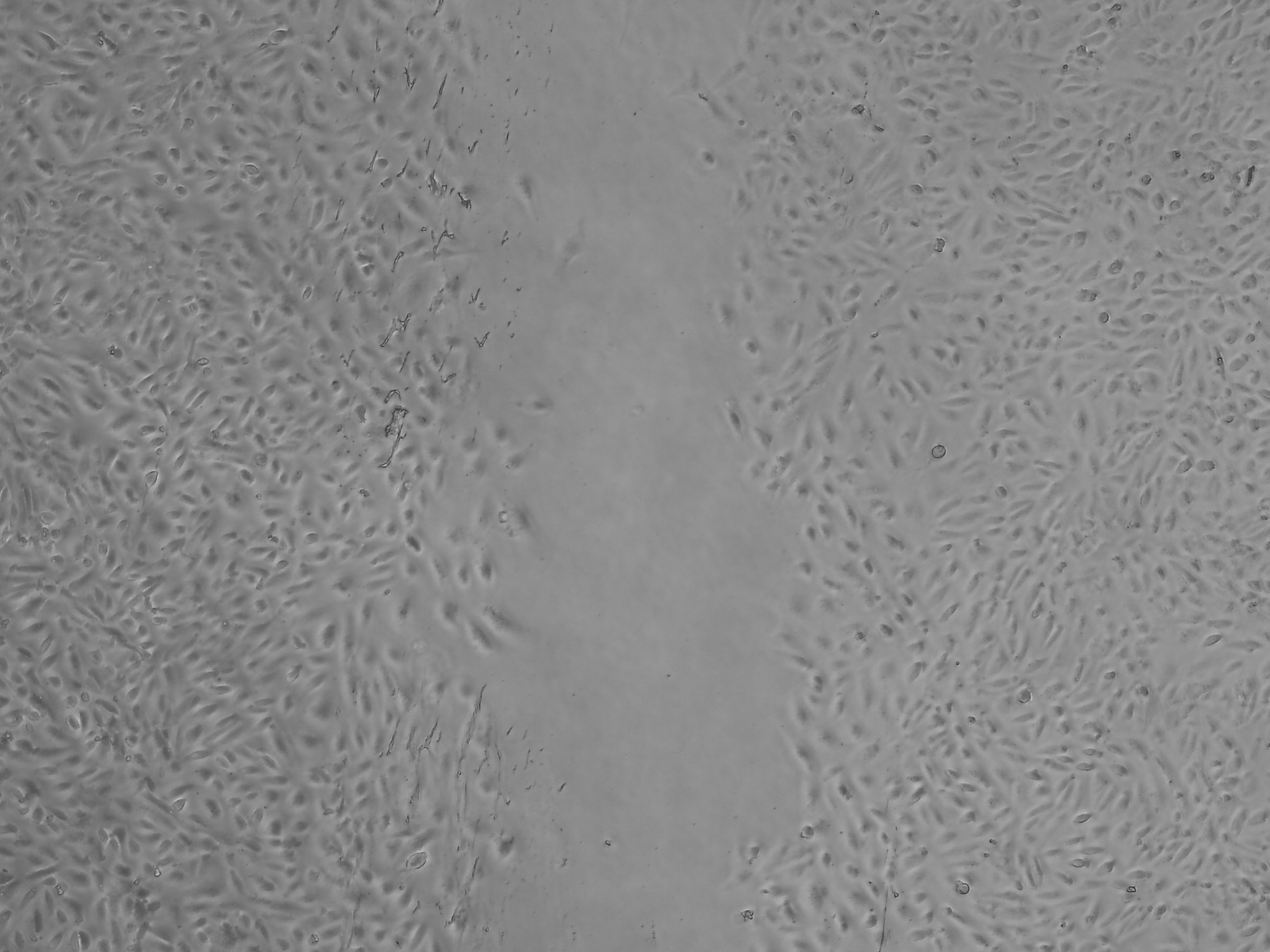

Supplement: Original Image for Figure 7A 48h 600 nM_3.tif [file IENZ_A_2423875_SM5309.tif]

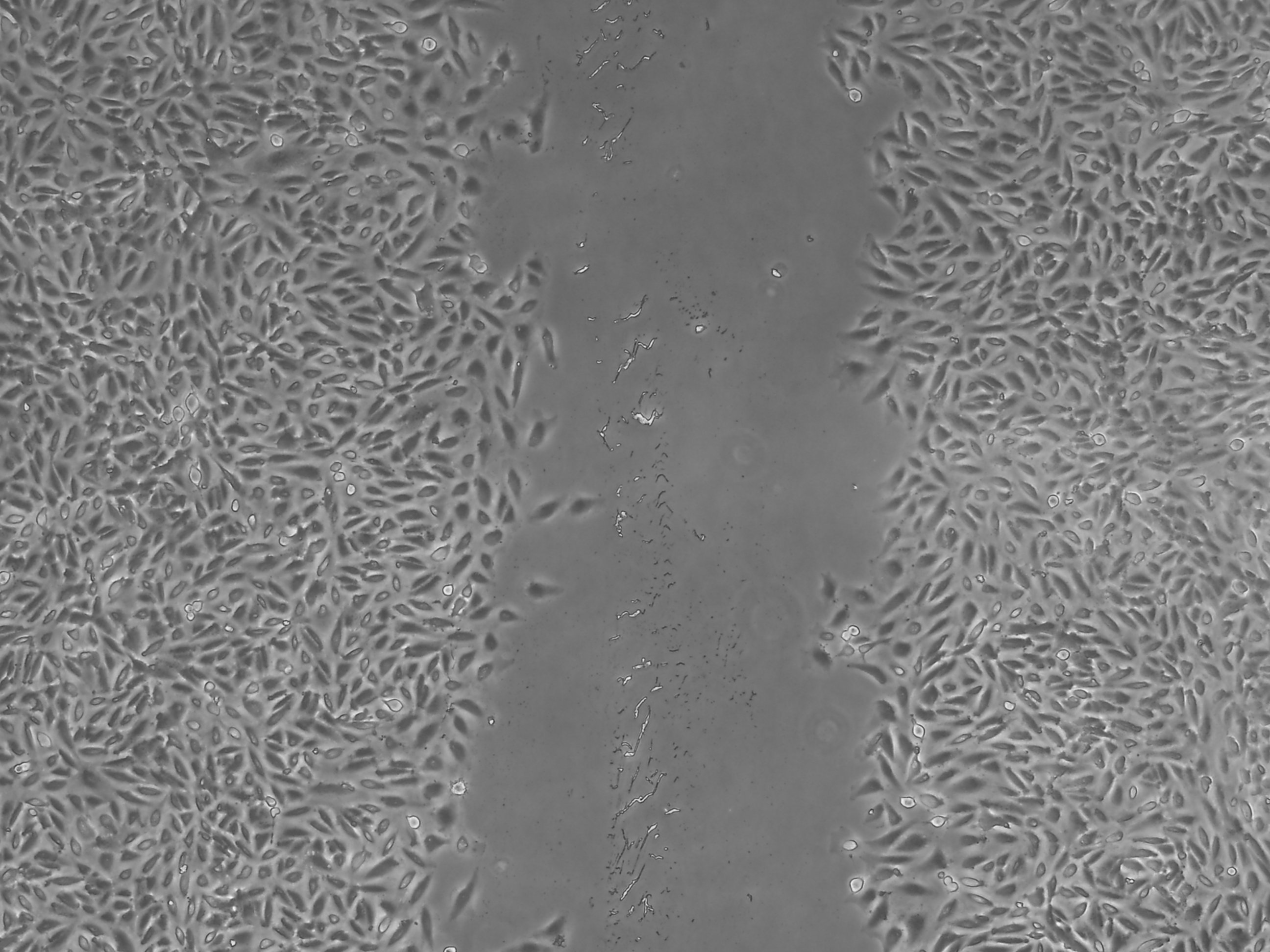

Supplement: Original Image for Figure 7A 24h 300 nM_1.tif [file IENZ_A_2423875_SM5308.tif]

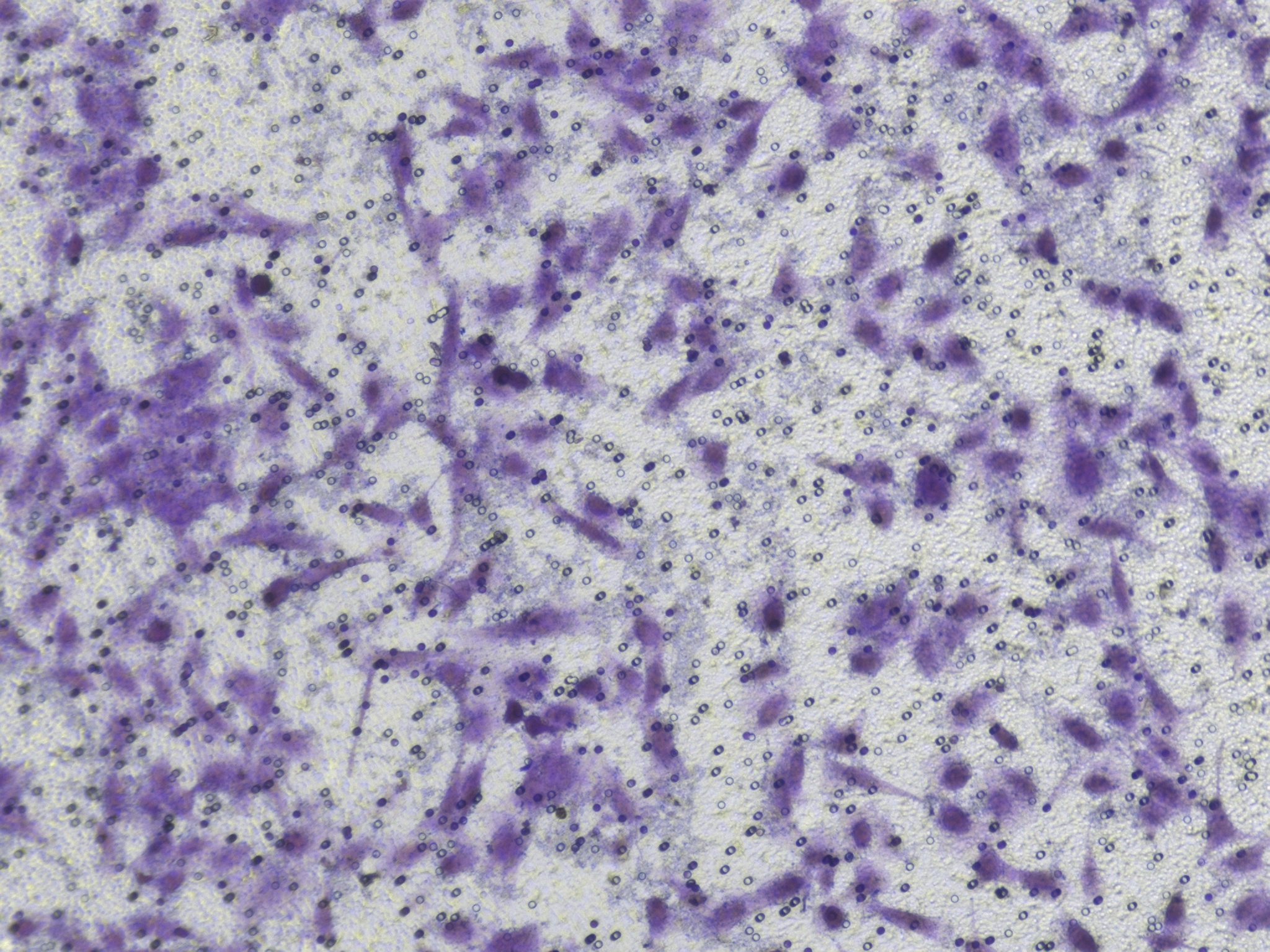

Supplement: Original Image for Figure 7C 300 nM.TIF [file IENZ_A_2423875_SM5307.tif]

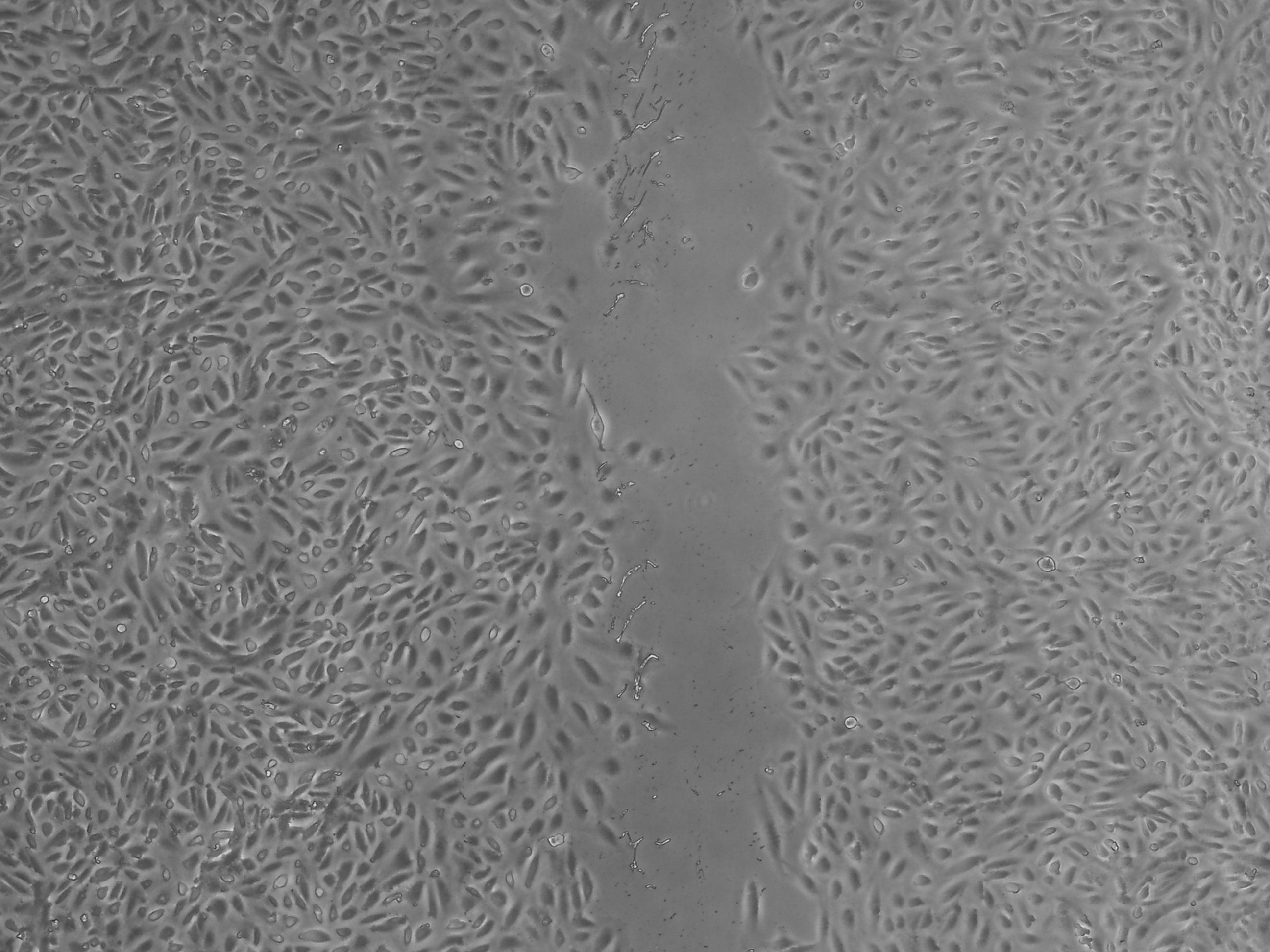

Supplement: Original Image for Figure 7A 48h 300 nM_2.tif [file IENZ_A_2423875_SM5306.tif]

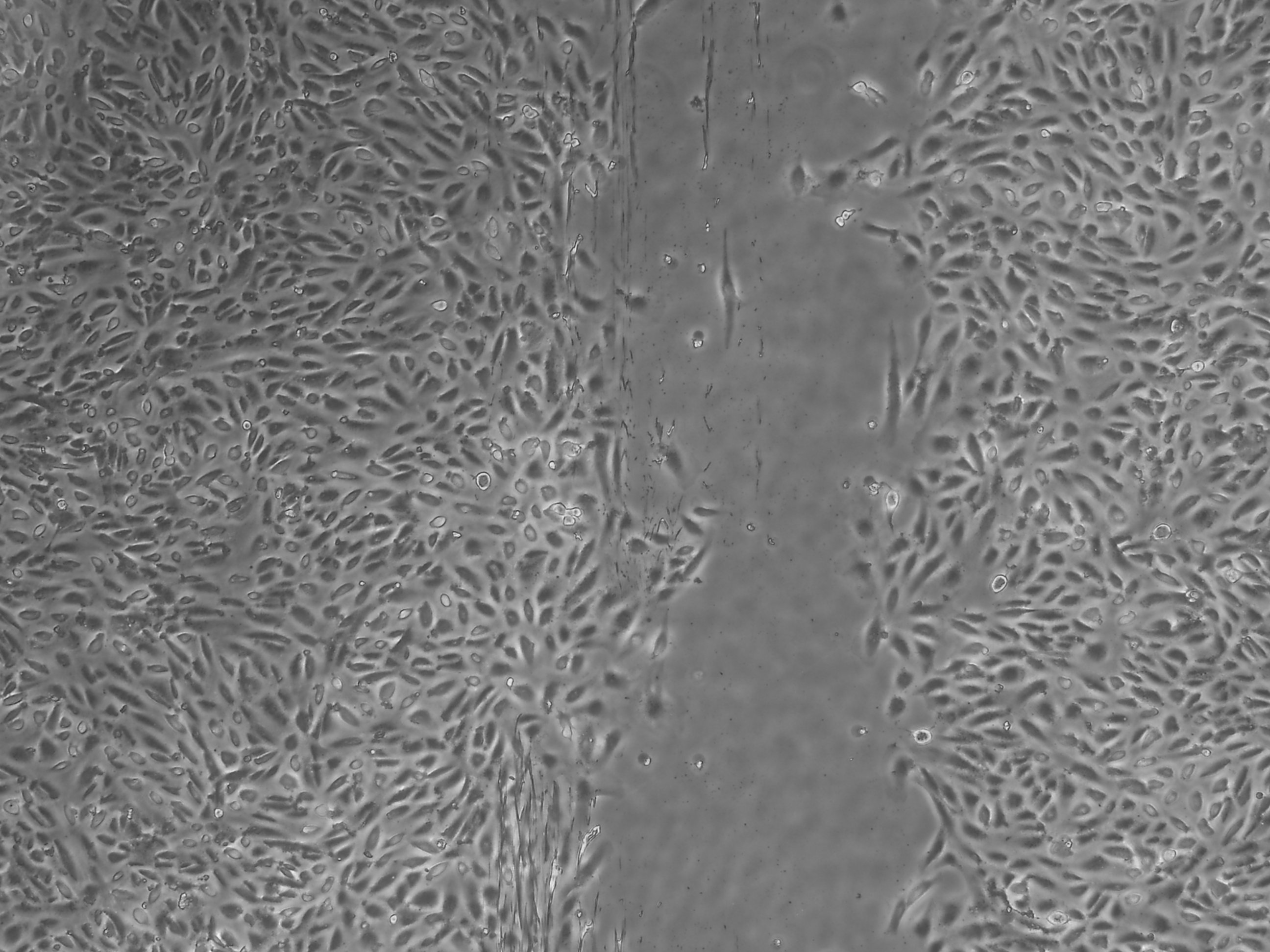

Supplement: Original Image for Figure 7A 48h 900 nM_3.tif [file IENZ_A_2423875_SM5305.tif]

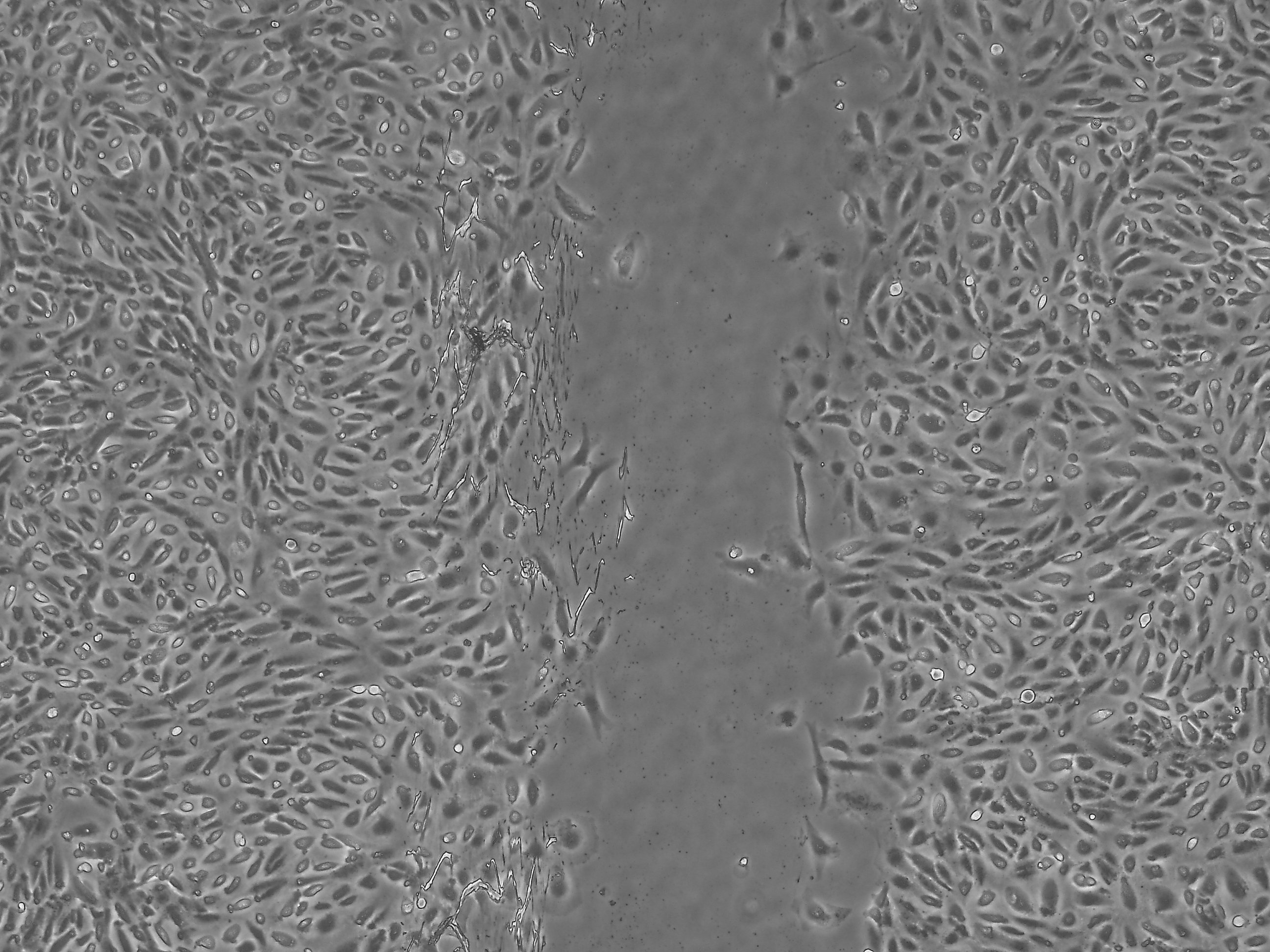

Supplement: Original Image for Figure 7A 48h 900 nM_1.tif [file IENZ_A_2423875_SM5304.tif]

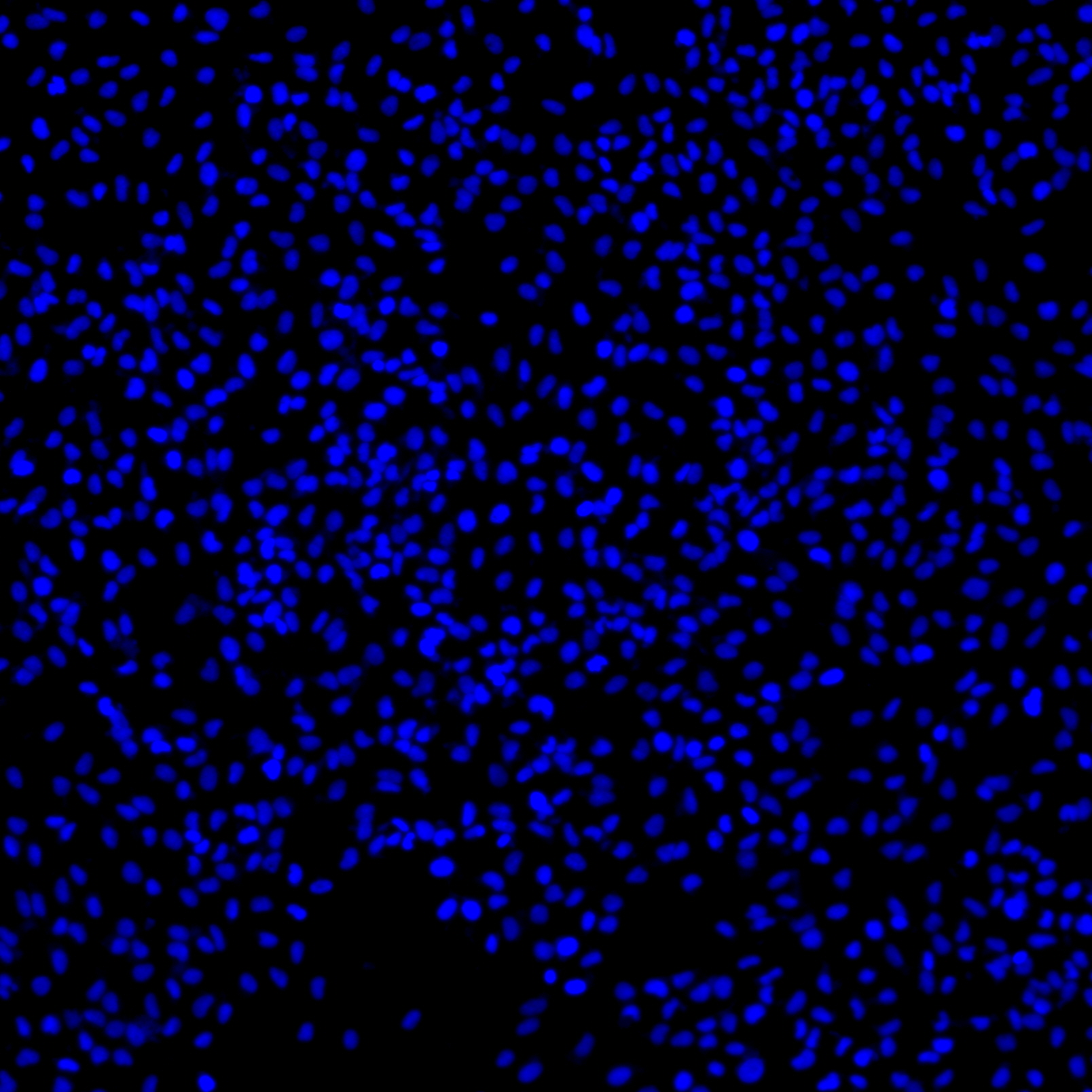

Supplement: Original Image for Figure 6A control_Hoechst.tif [file IENZ_A_2423875_SM5303.tif]

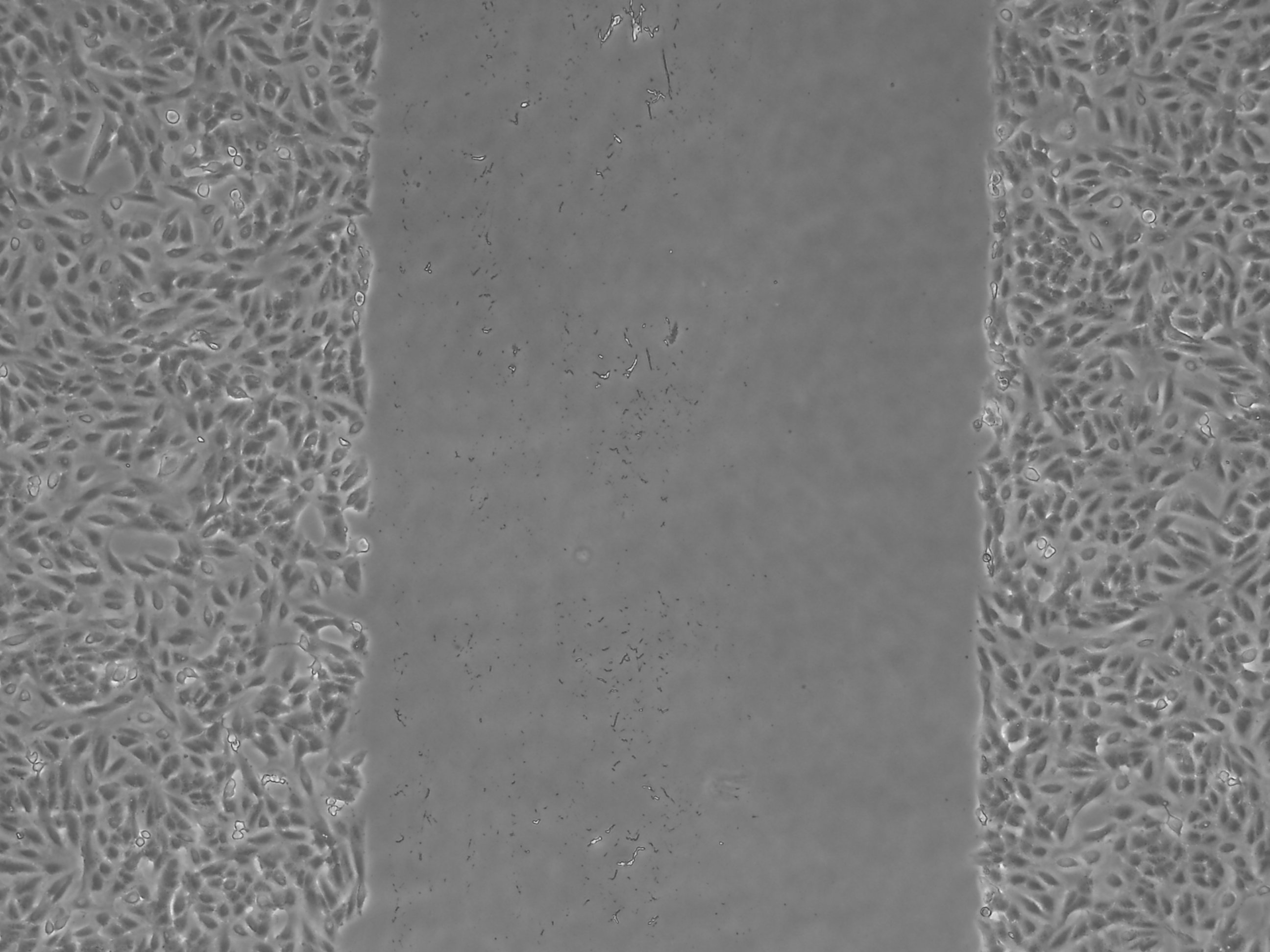

Supplement: Original Image for Figure 7A 0h 900 nM_1.tif [file IENZ_A_2423875_SM5302.tif]

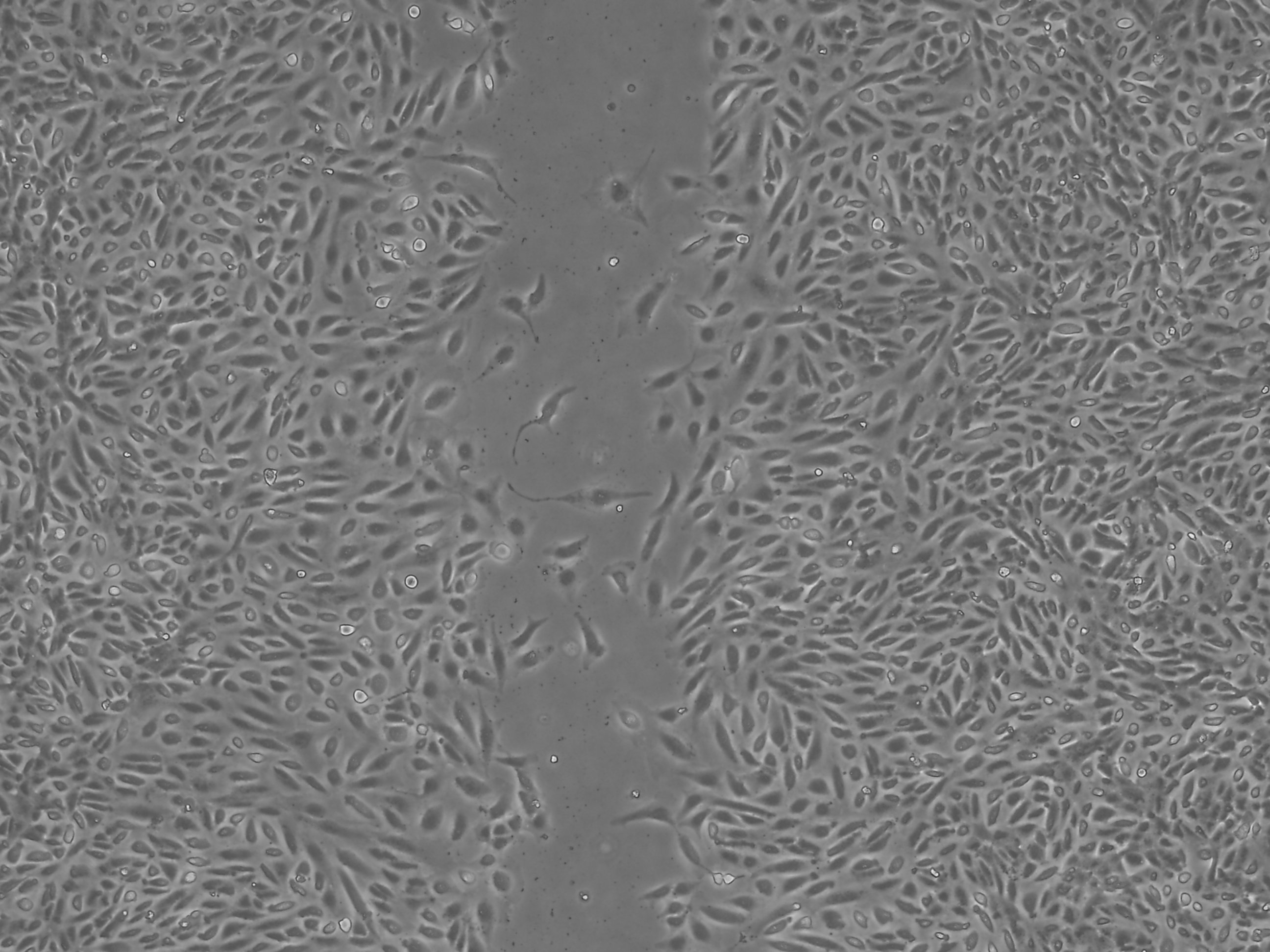

Supplement: Original Image for Figure 7A 36h 300 nM_2.tif [file IENZ_A_2423875_SM5301.tif]

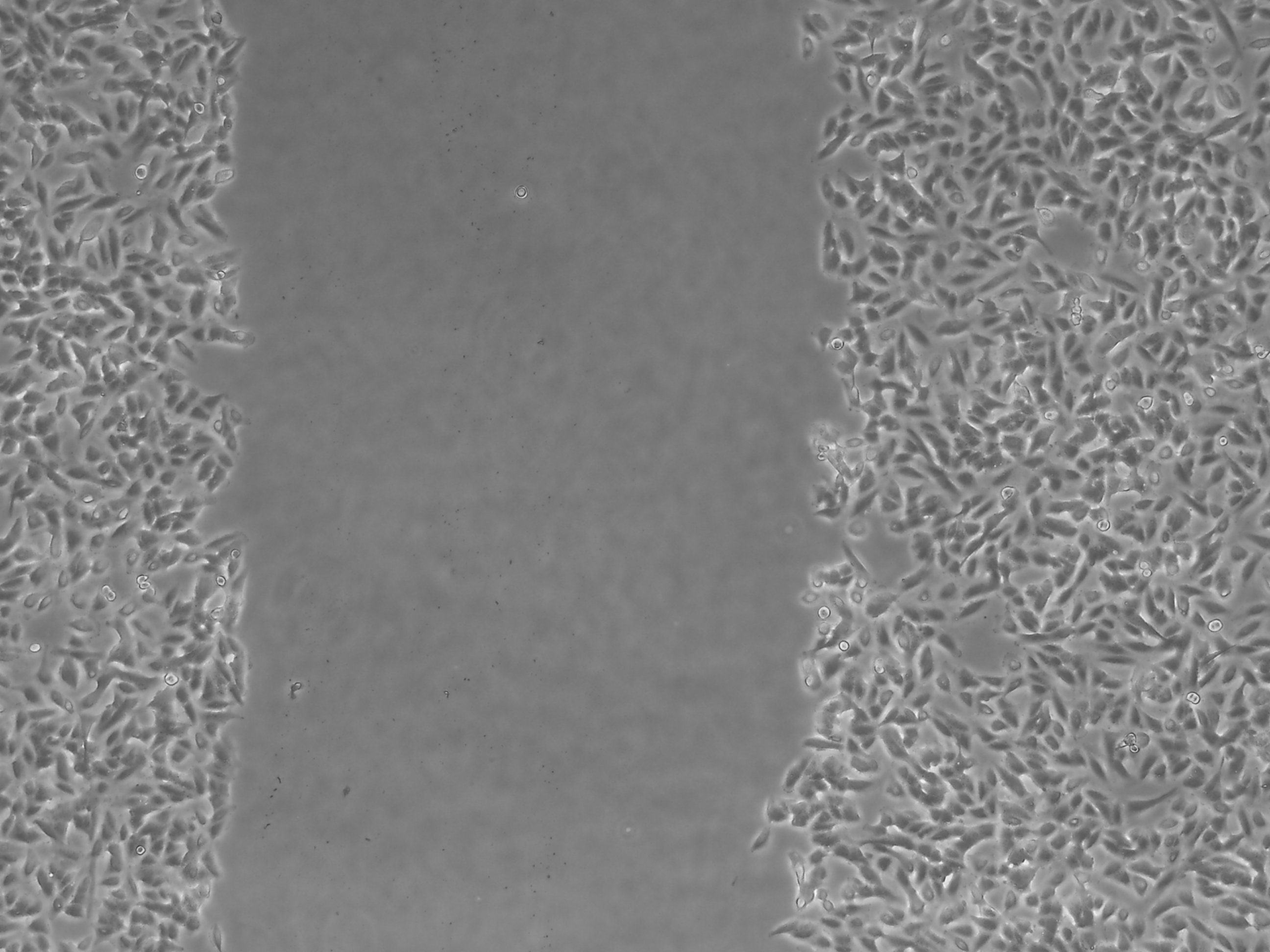

Supplement: Original Image for Figure 7A 0h 300 nM_3.tif [file IENZ_A_2423875_SM5300.tif]

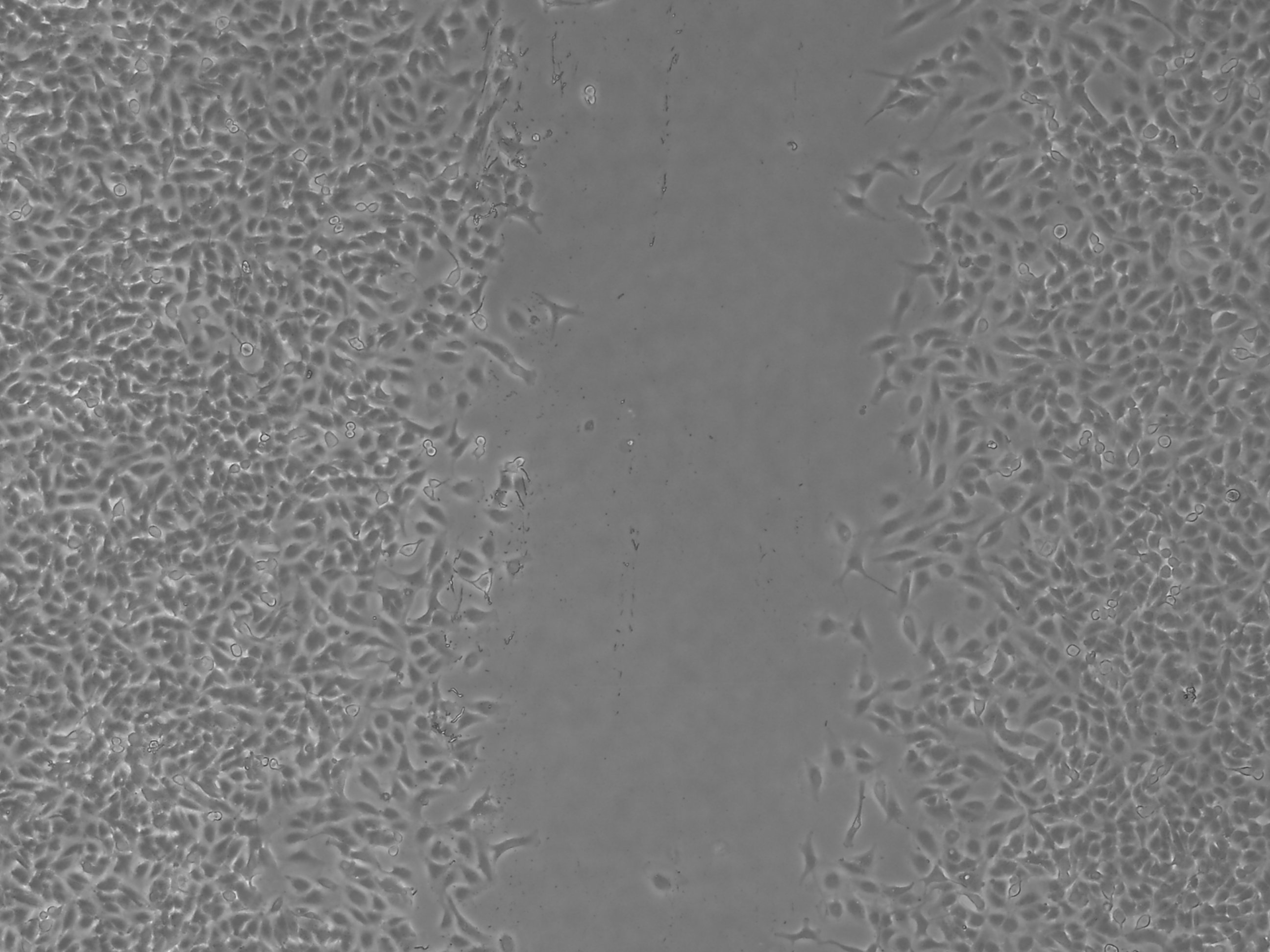

Supplement: Original Image for Figure 7A 12h Control_2.tif [file IENZ_A_2423875_SM5299.tif]

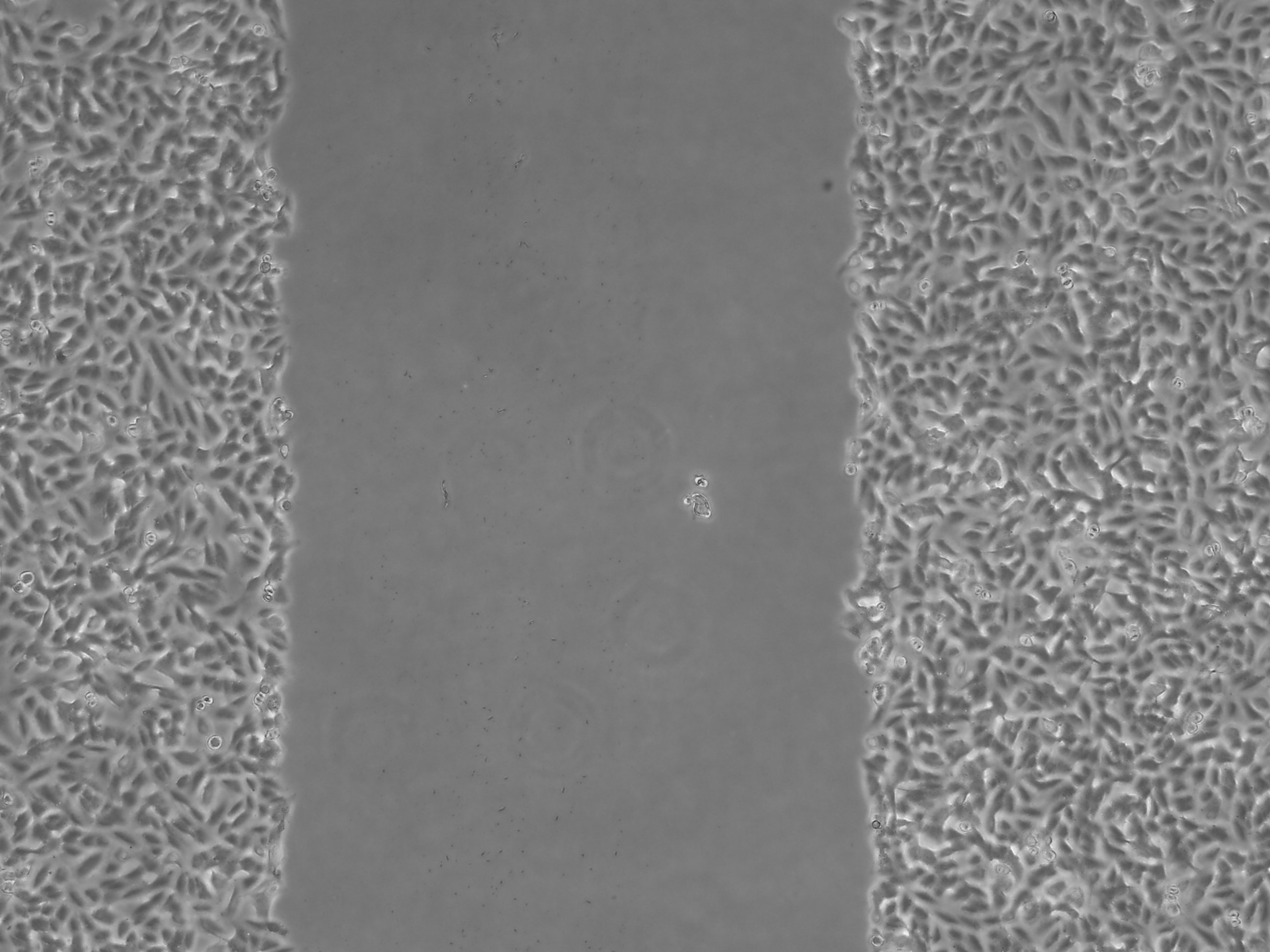

Supplement: Original Image for Figure 7A 0h 300 nM_2.tif [file IENZ_A_2423875_SM5298.tif]

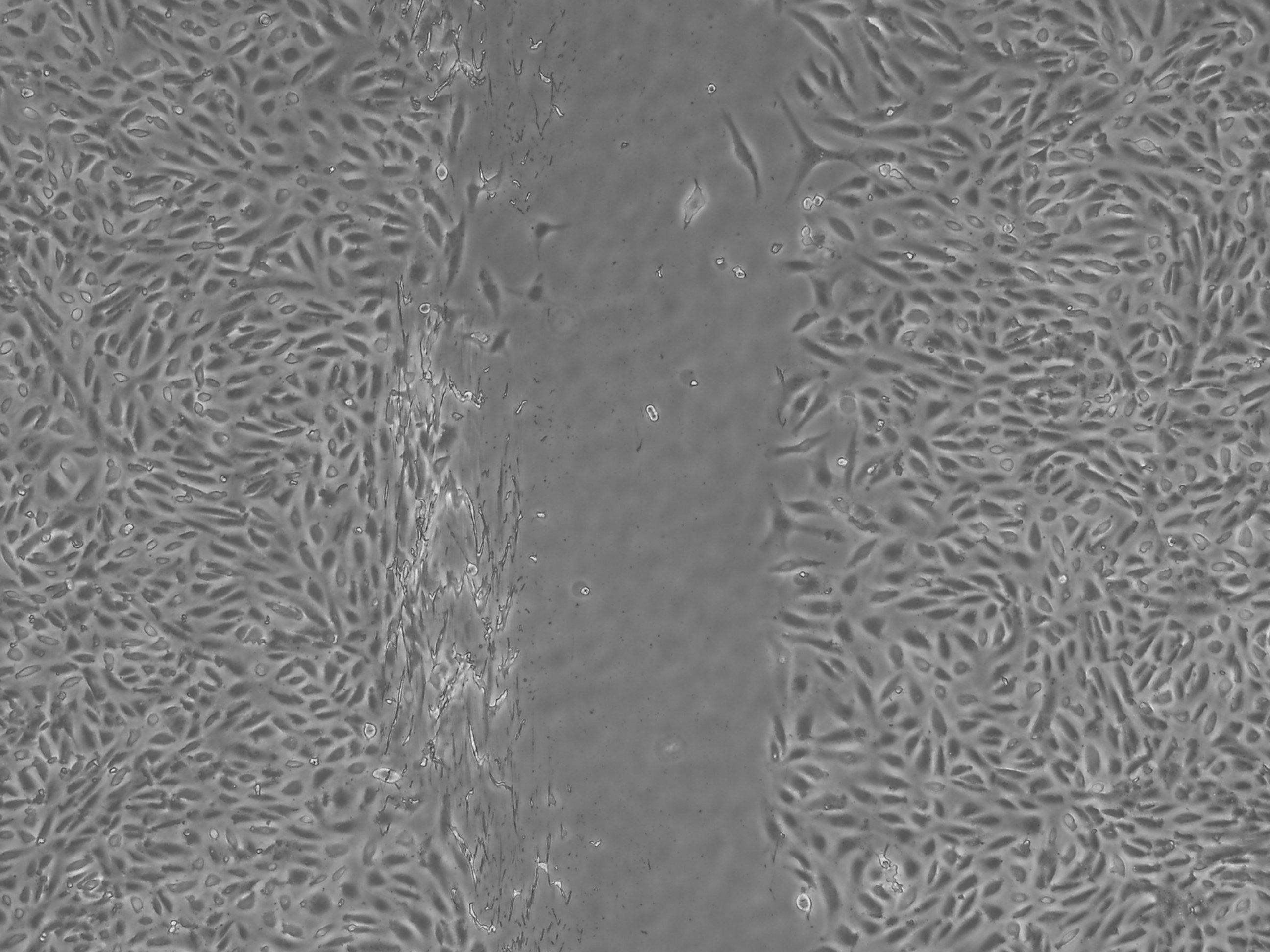

Supplement: Original Image for Figure 7A 36h 900 nM_1.tif [file IENZ_A_2423875_SM5297.tif]

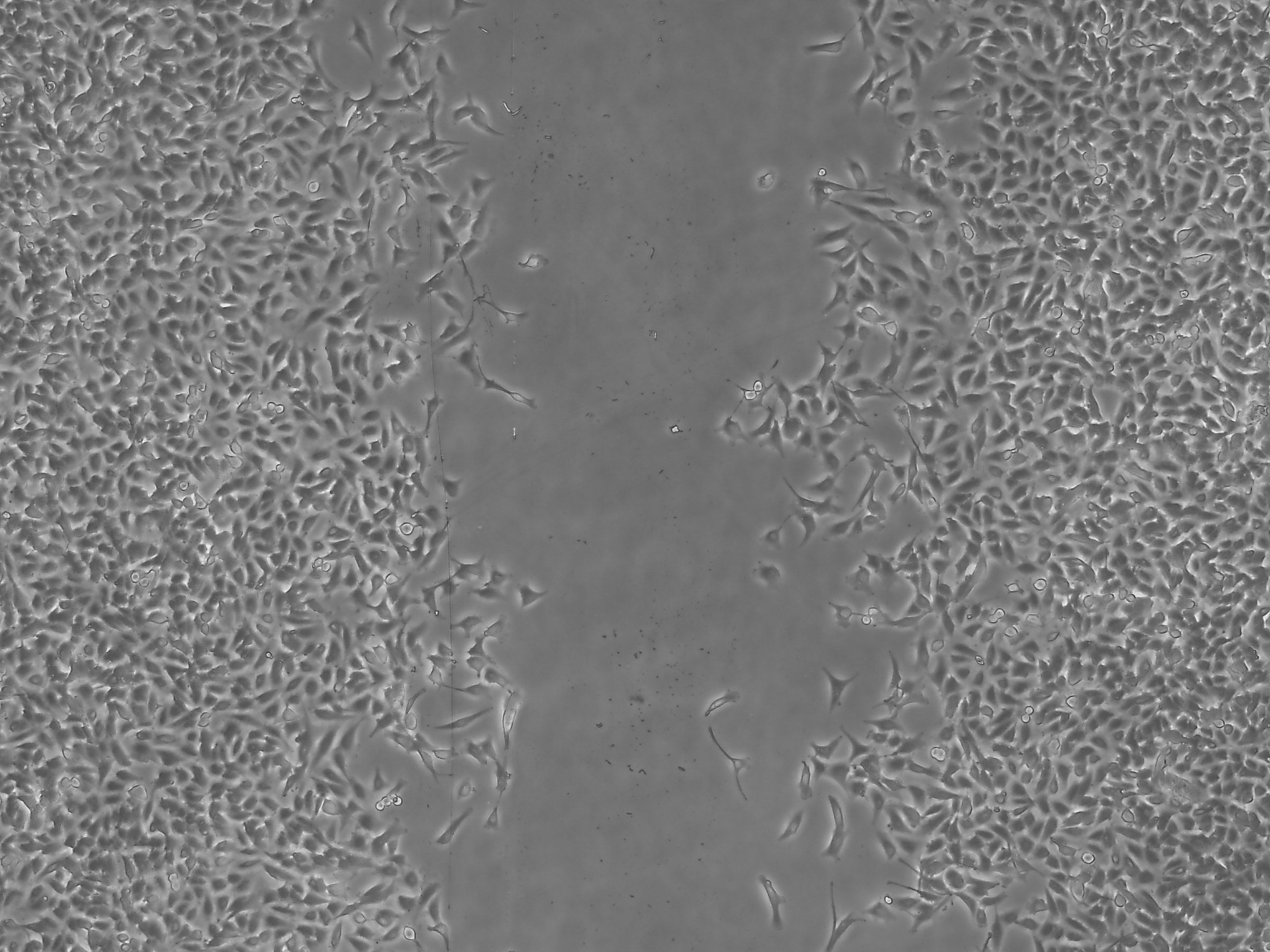

Supplement: Original Image for Figure 7A 24h Control_3.tif [file IENZ_A_2423875_SM5296.tif]

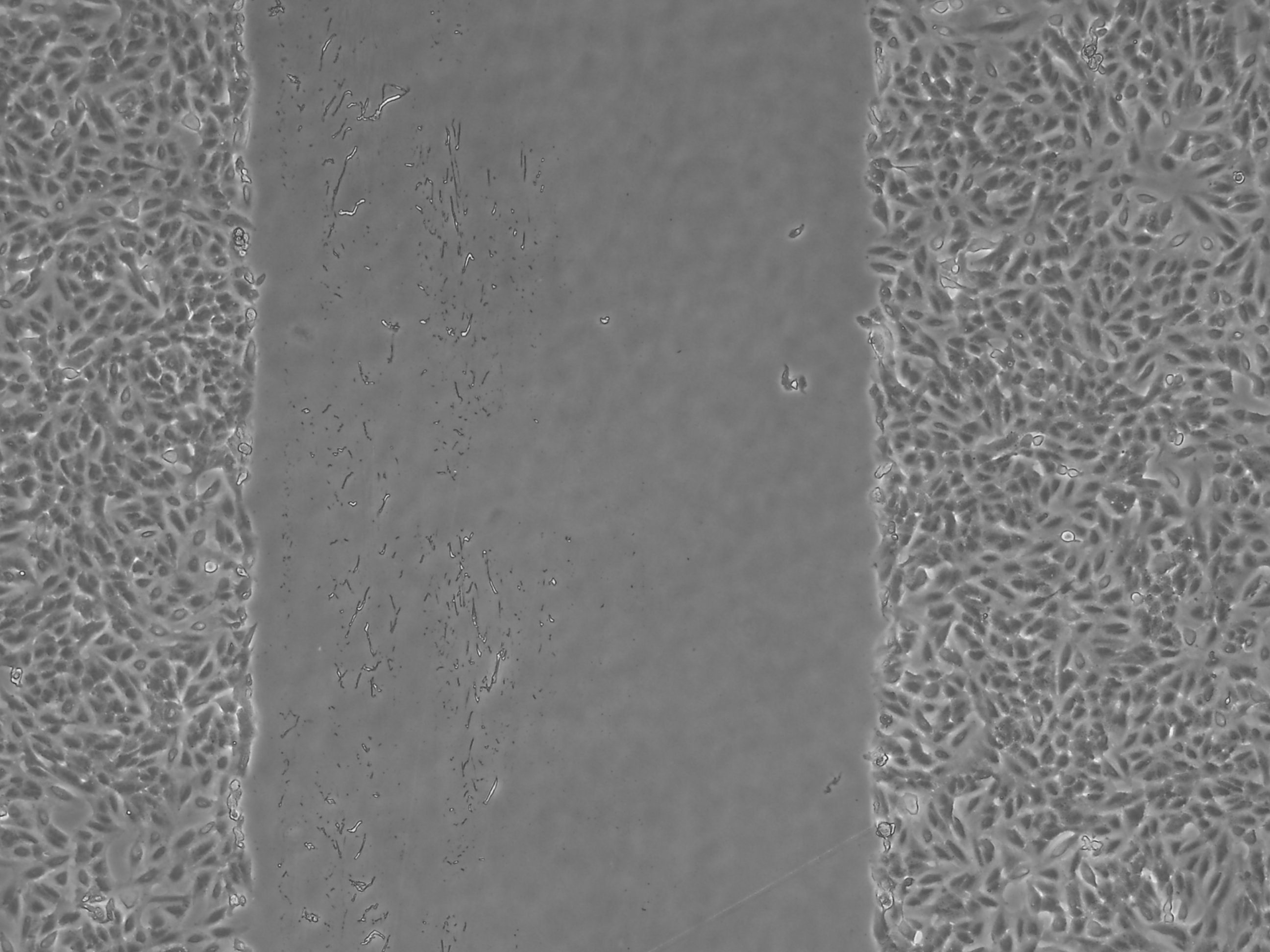

Supplement: Original Image for Figure 7A 0h 900 nM_3.tif [file IENZ_A_2423875_SM5295.tif]

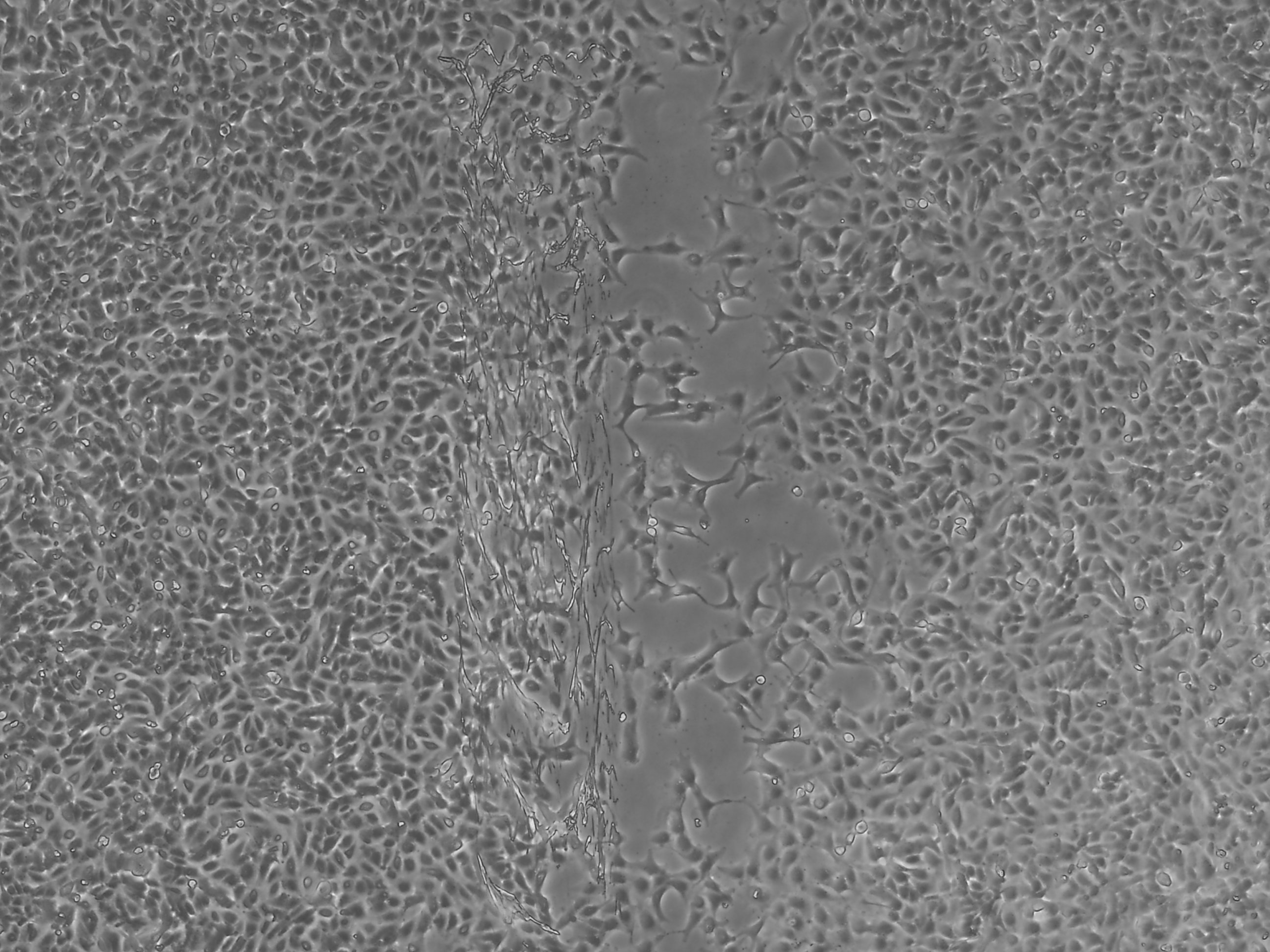

Supplement: Original Image for Figure 7A 48h Control_2.tif [file IENZ_A_2423875_SM5294.tif]

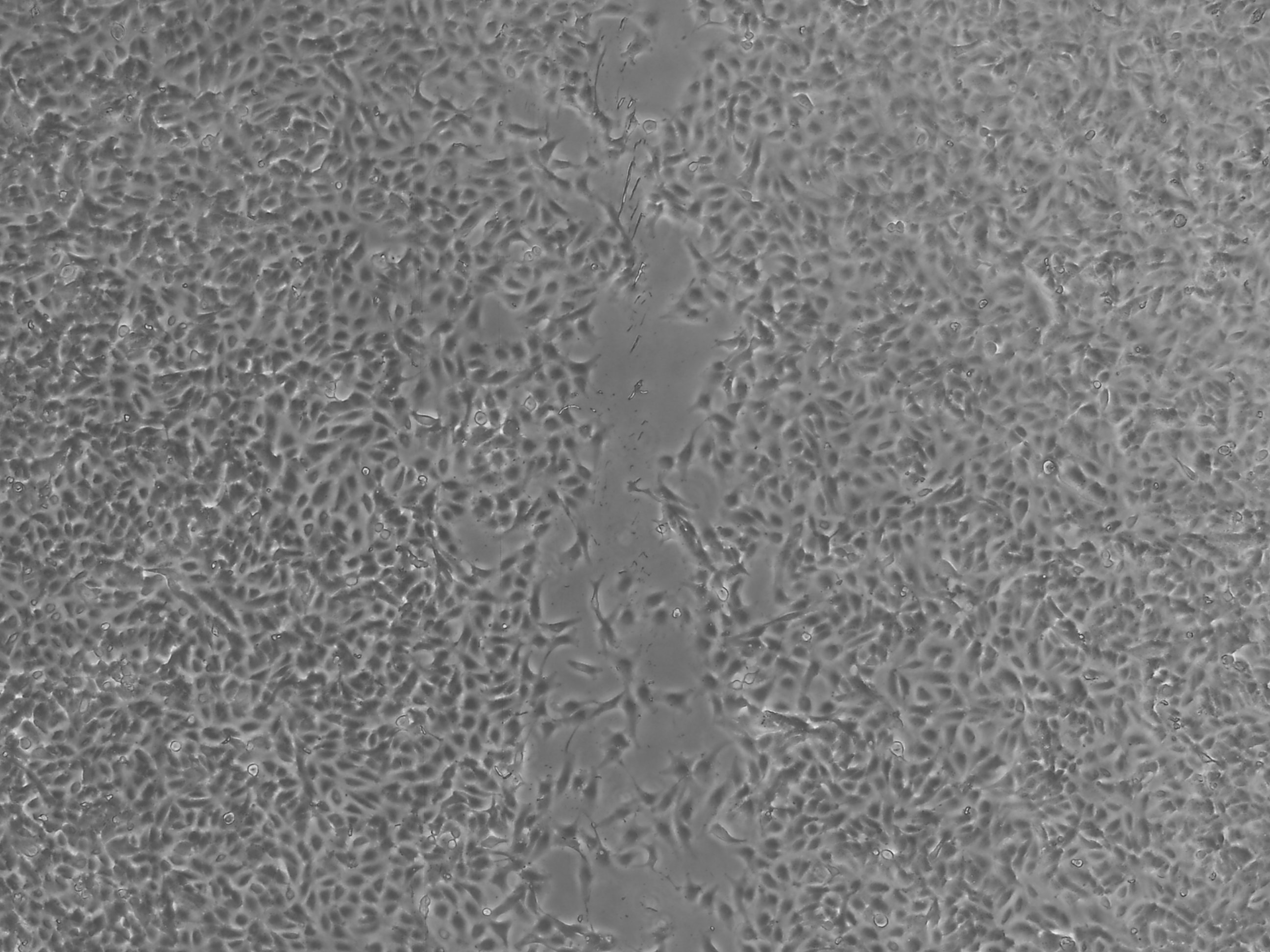

Supplement: Original Image for Figure 7A 48h Control_1.tif [file IENZ_A_2423875_SM5293.tif]

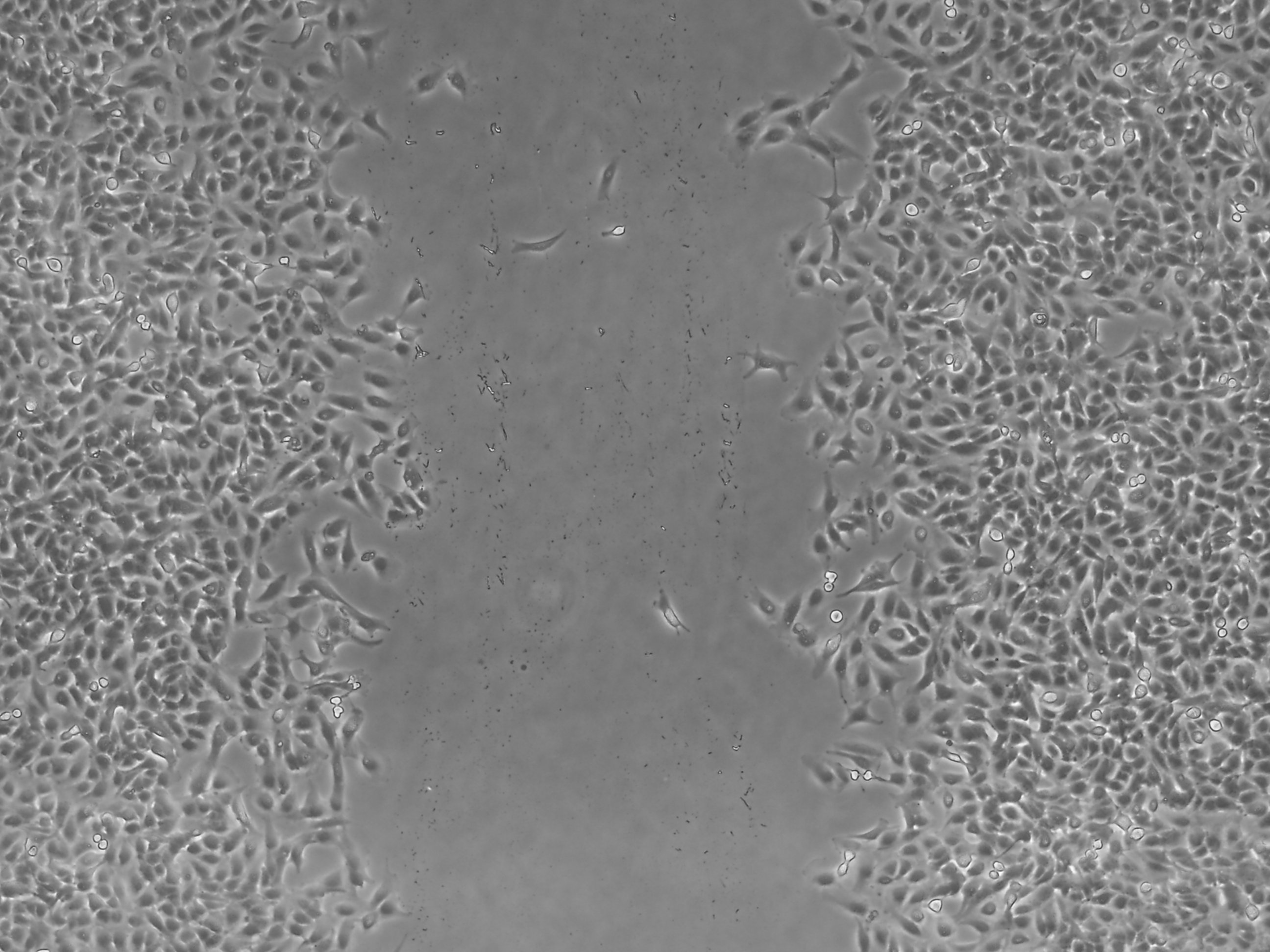

Supplement: Original Image for Figure 7A 12h Control_1.tif [file IENZ_A_2423875_SM5292.tif]

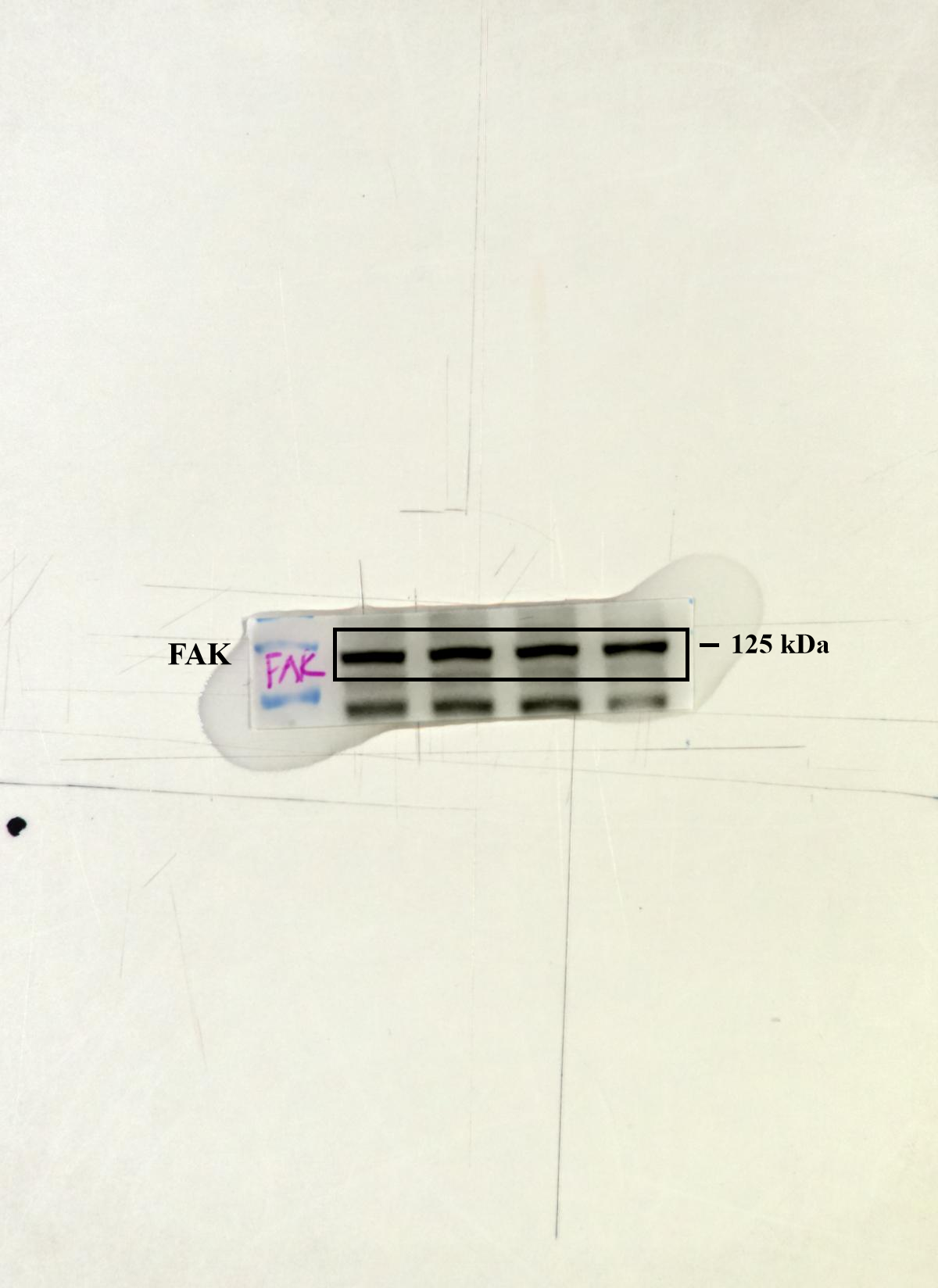

Supplement: Original Image for Fig 5B_FAK.tif [file IENZ_A_2423875_SM5291.tif]

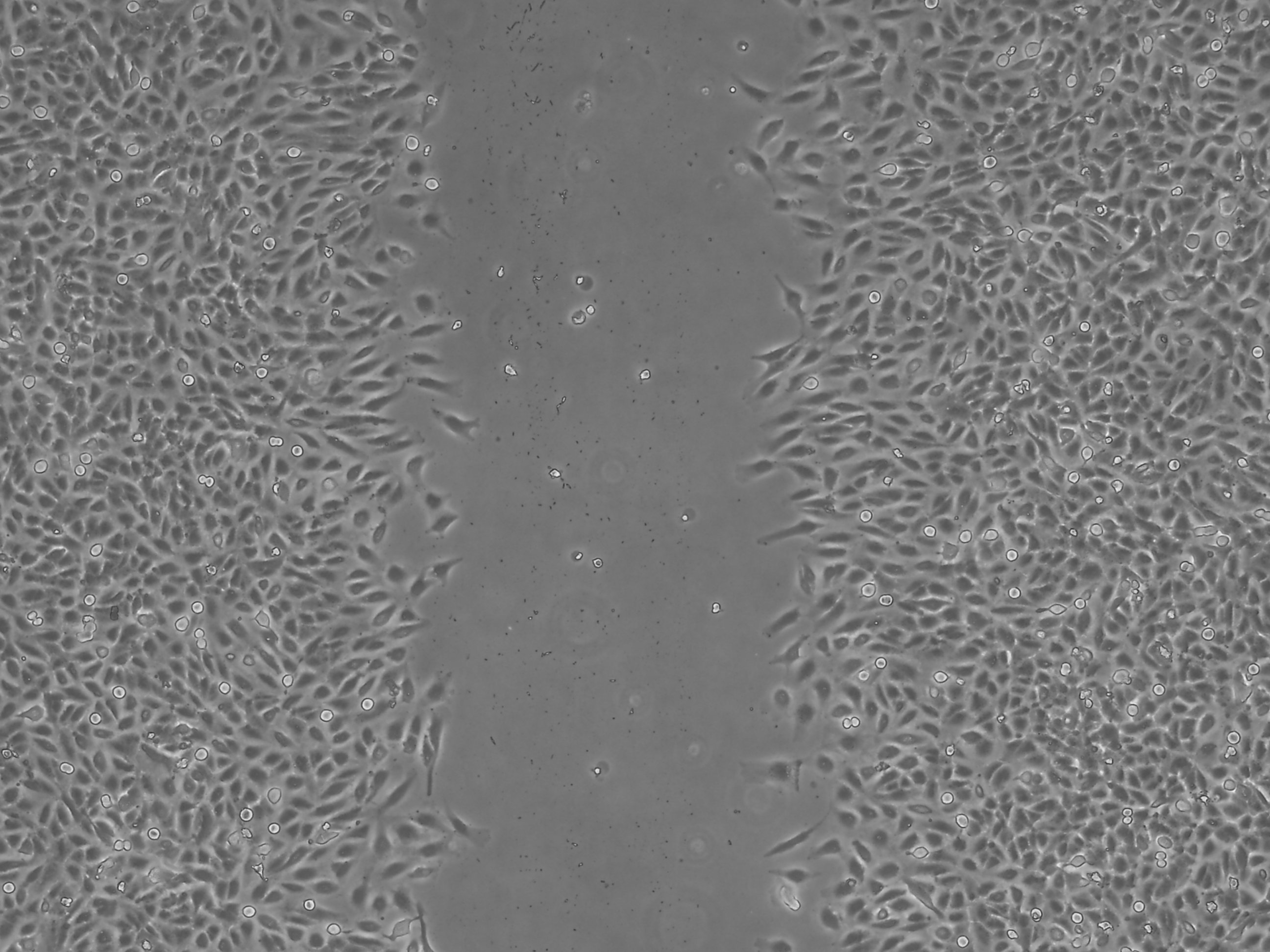

Supplement: Original Image for Figure 7A 12h 300 nM_2.tif [file IENZ_A_2423875_SM5290.tif]

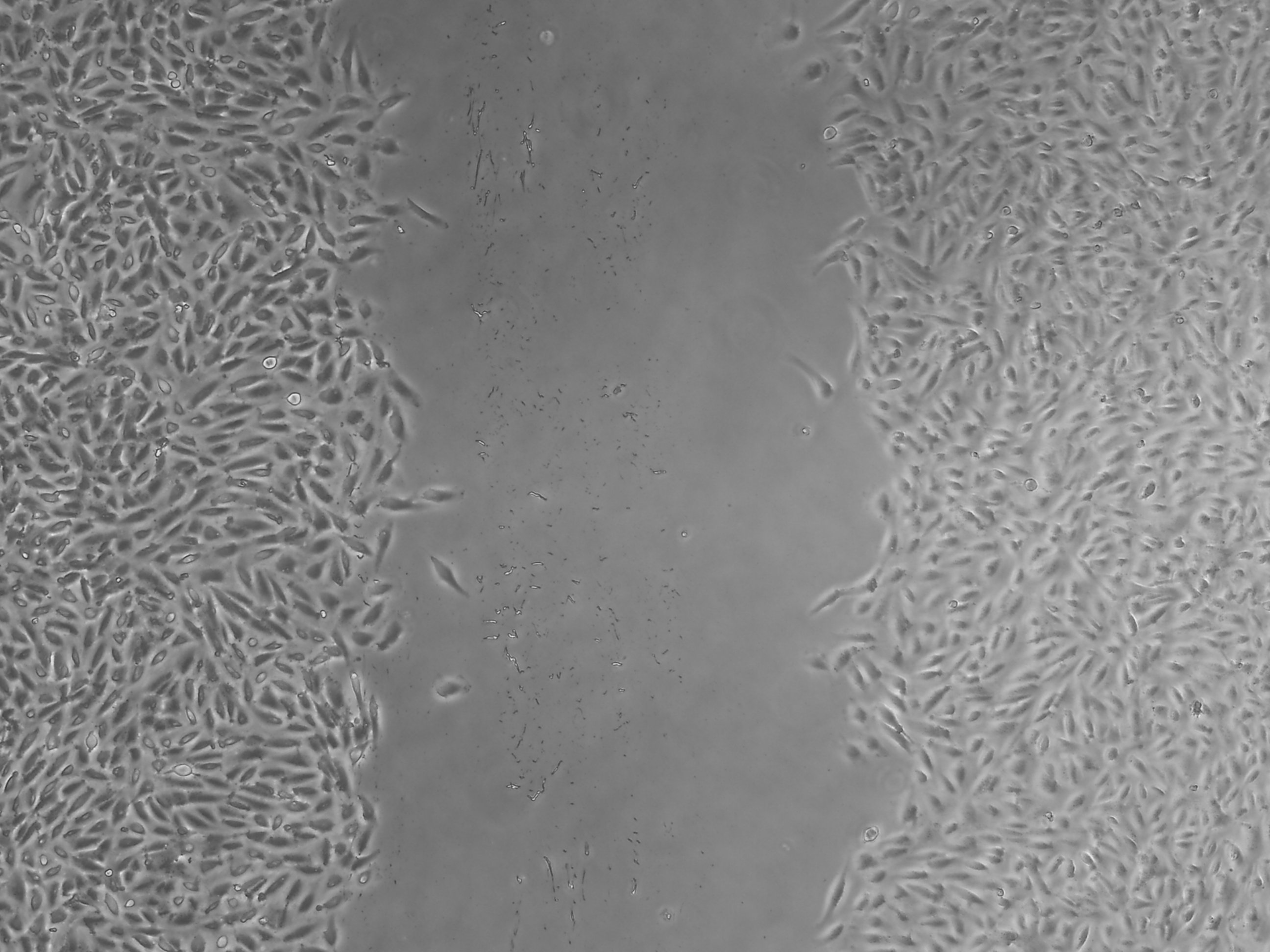

Supplement: Original Image for Figure 7A 24h 900 nM_3.tif [file IENZ_A_2423875_SM5289.tif]

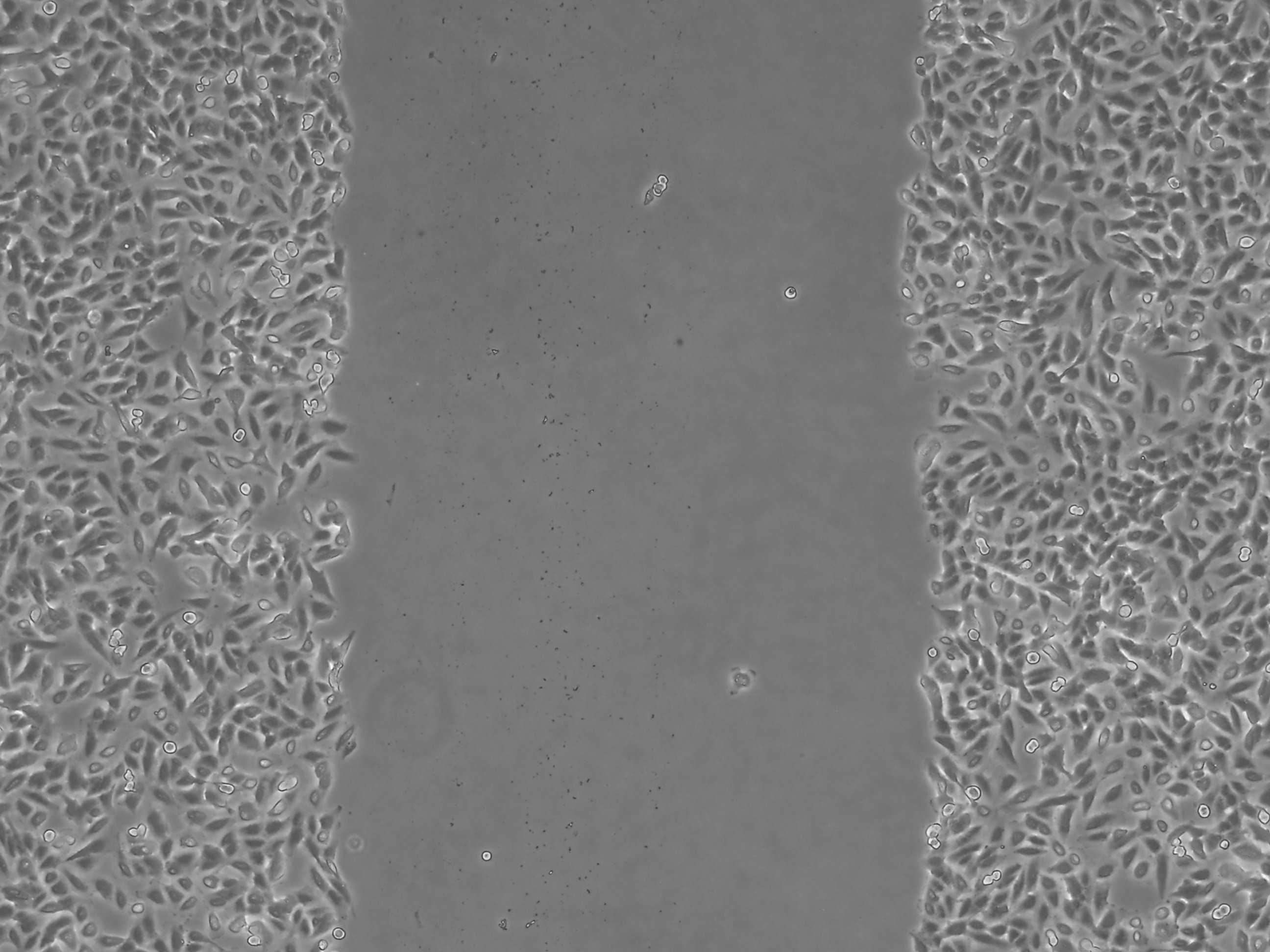

Supplement: Original Image for Figure 7A 0h 300 nM_1.tif [file IENZ_A_2423875_SM5288.tif]

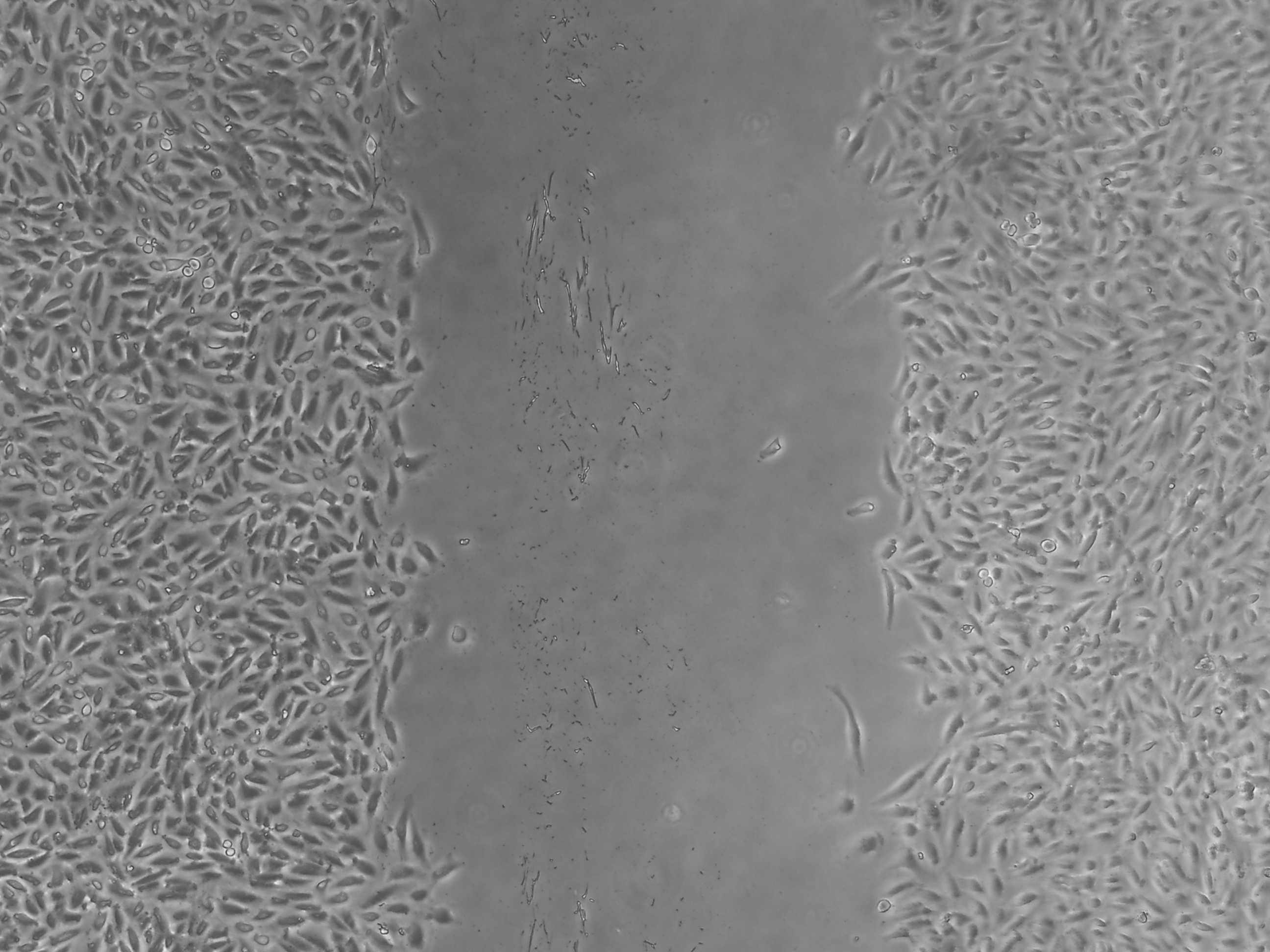

Supplement: Original Image for Figure 7A 24h 900 nM_2.tif [file IENZ_A_2423875_SM5287.tif]

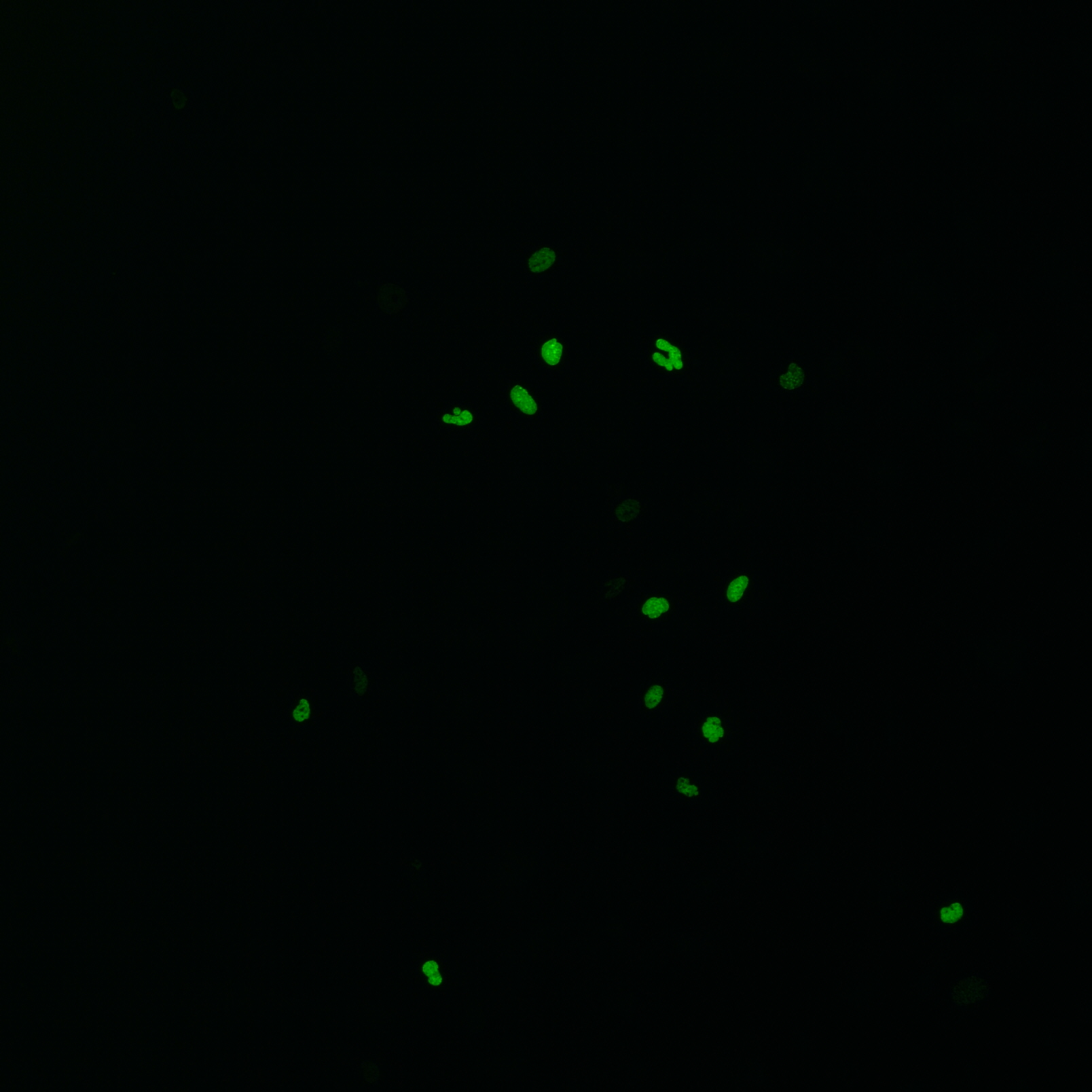

Supplement: Original Image for Figure 6A 600 nM_EdU.tif [file IENZ_A_2423875_SM5286.tif]

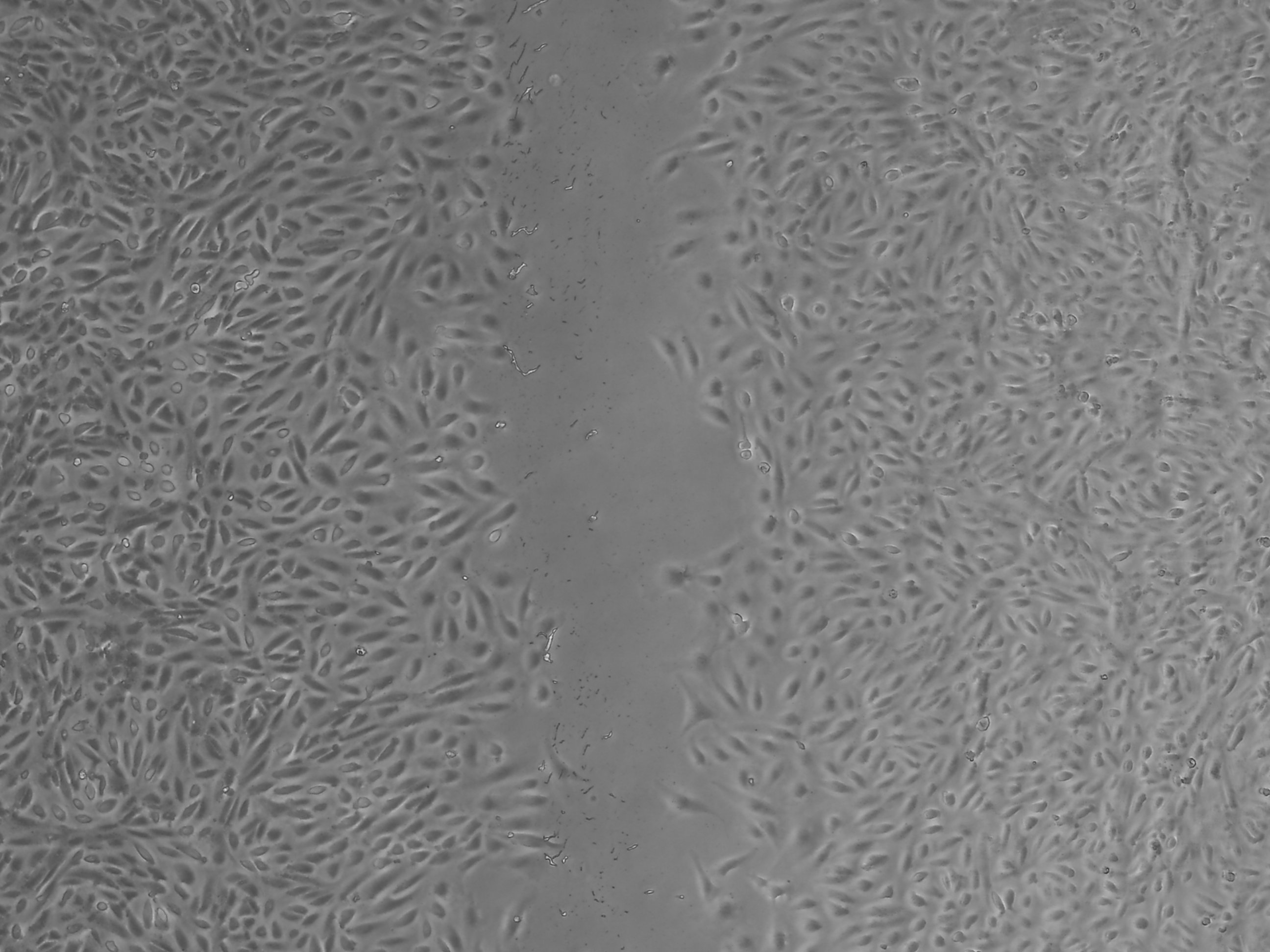

Supplement: Original Image for Figure 7A 36h 300 nM_3.tif [file IENZ_A_2423875_SM5285.tif]

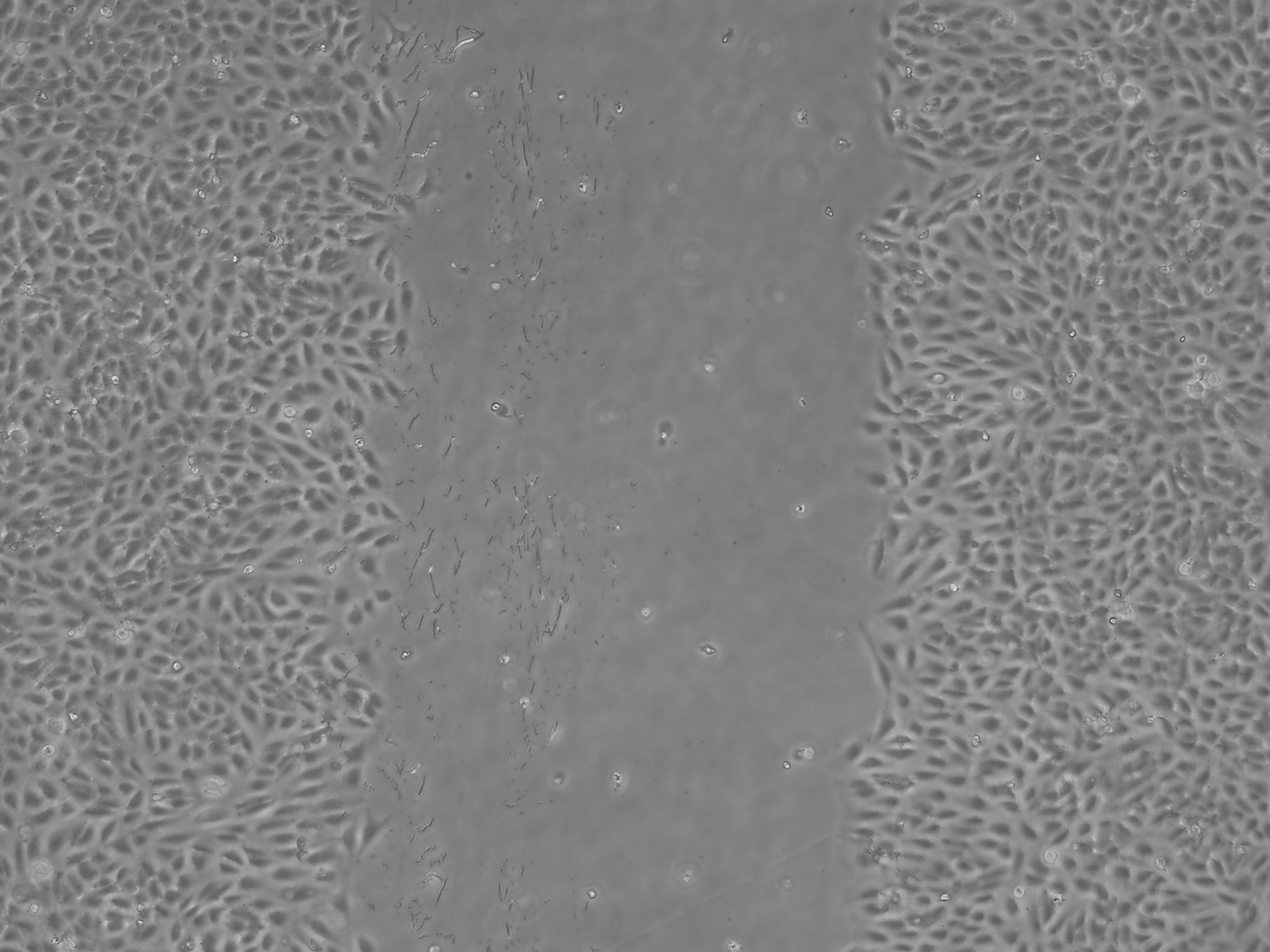

Supplement: Original Image for Figure 7A 12h 900 nM_3.tif [file IENZ_A_2423875_SM5284.tif]

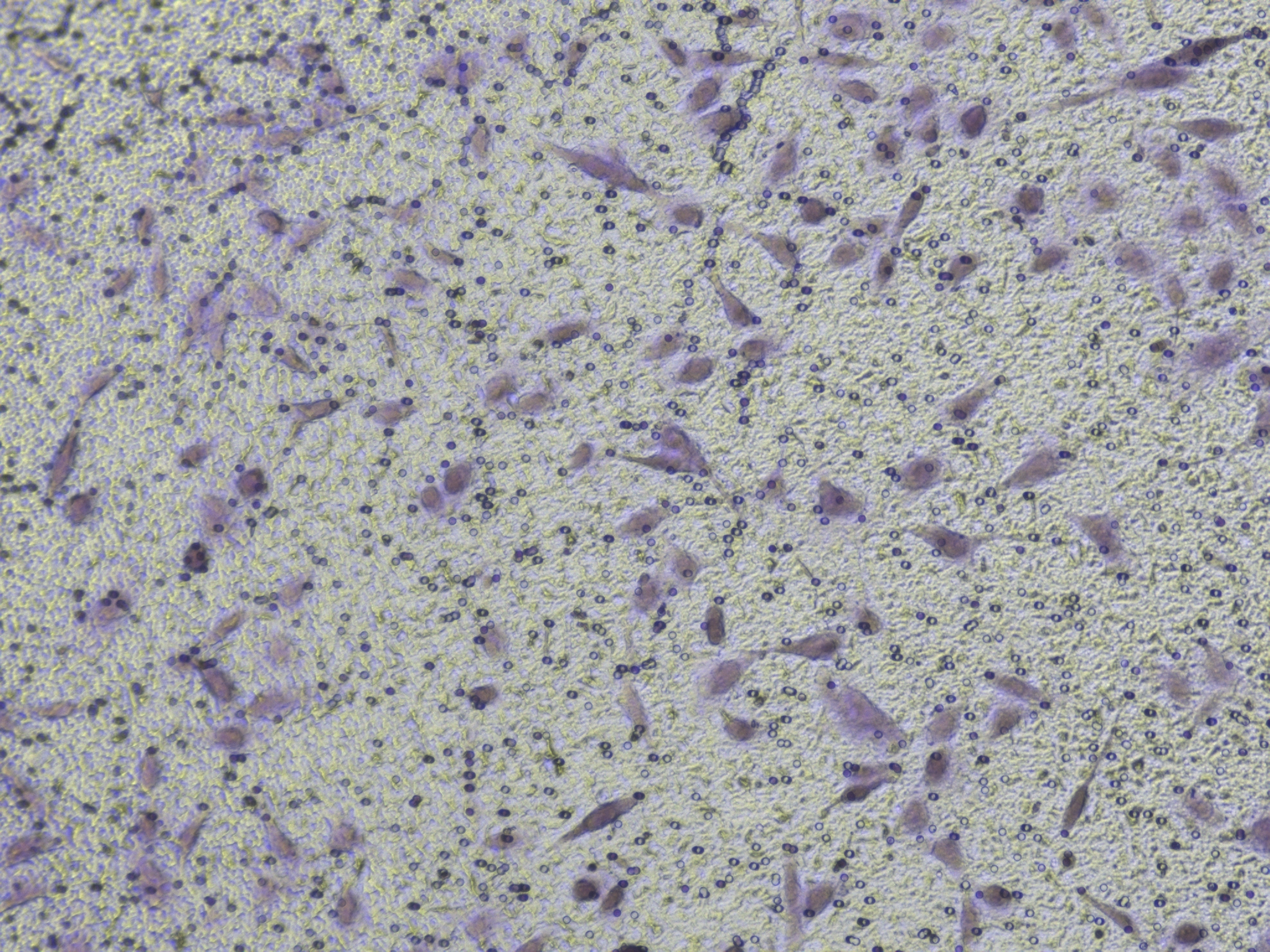

Supplement: Original Image for Figure 7C 900 nM.TIF [file IENZ_A_2423875_SM5283.tif]

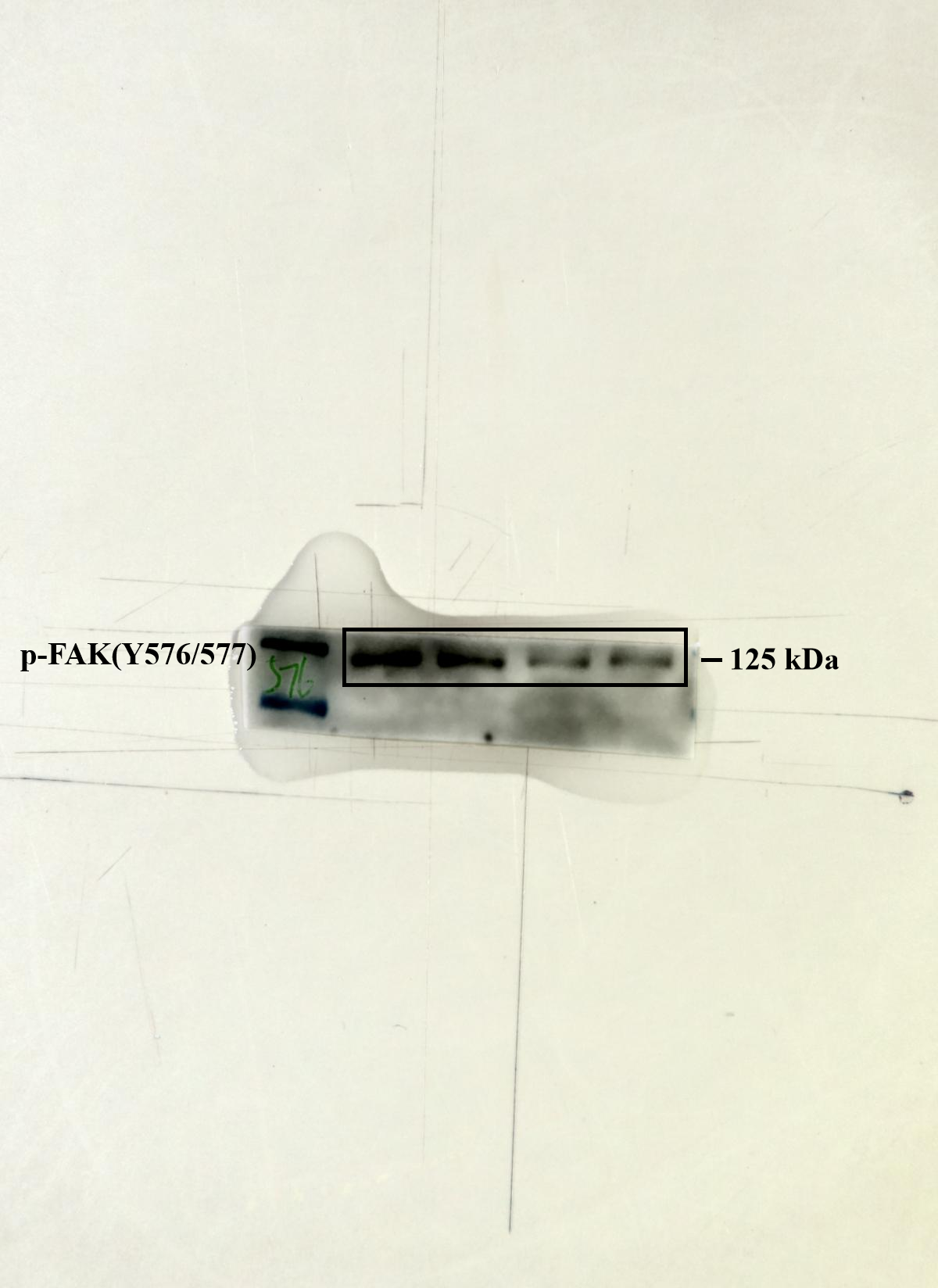

Supplement: Original Image for Fig 5B_p_FAK Y576 577.tif [file IENZ_A_2423875_SM5282.tif]

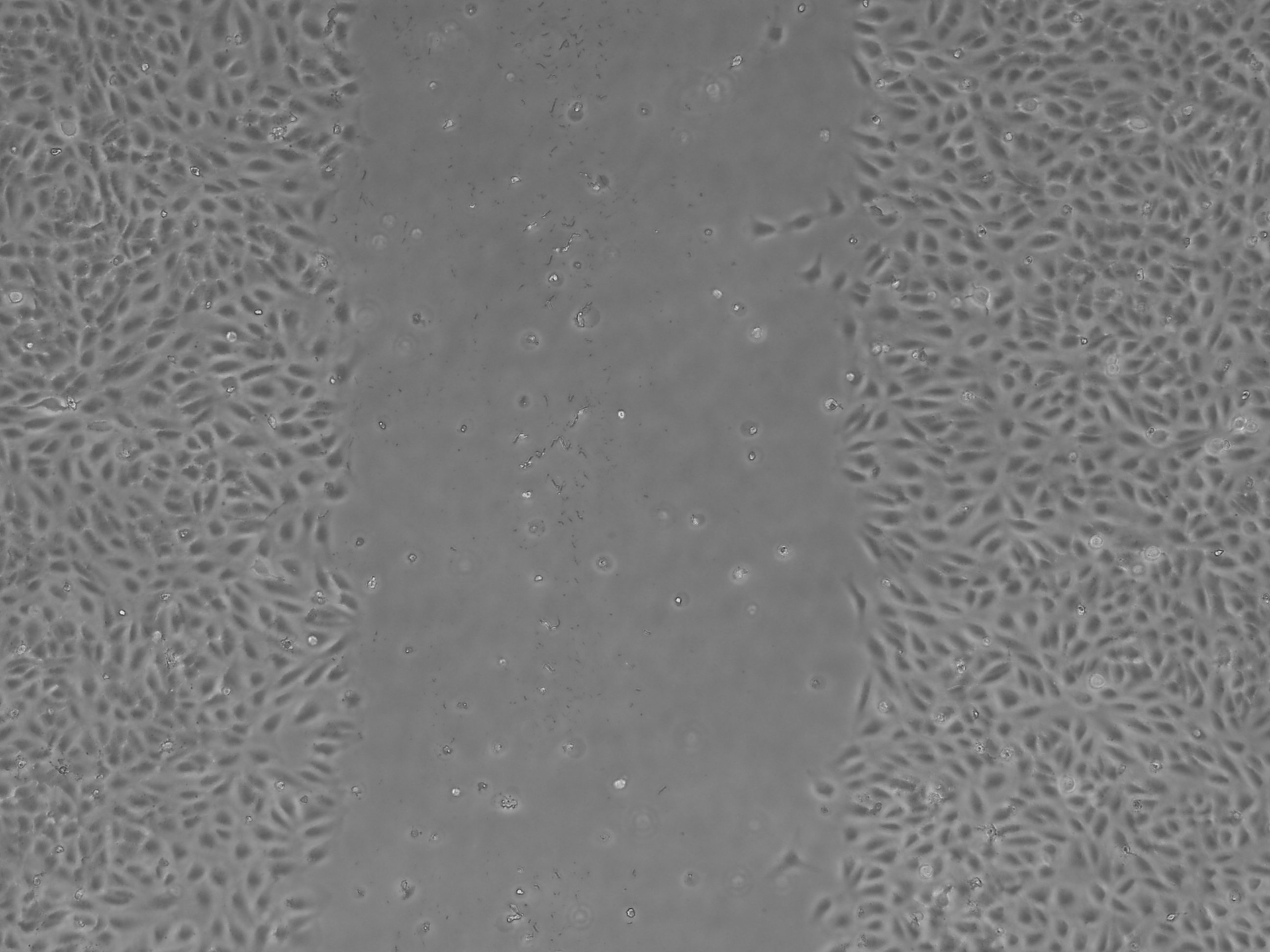

Supplement: Original Image for Figure 7A 12h 900 nM_2.tif [file IENZ_A_2423875_SM5281.tif]

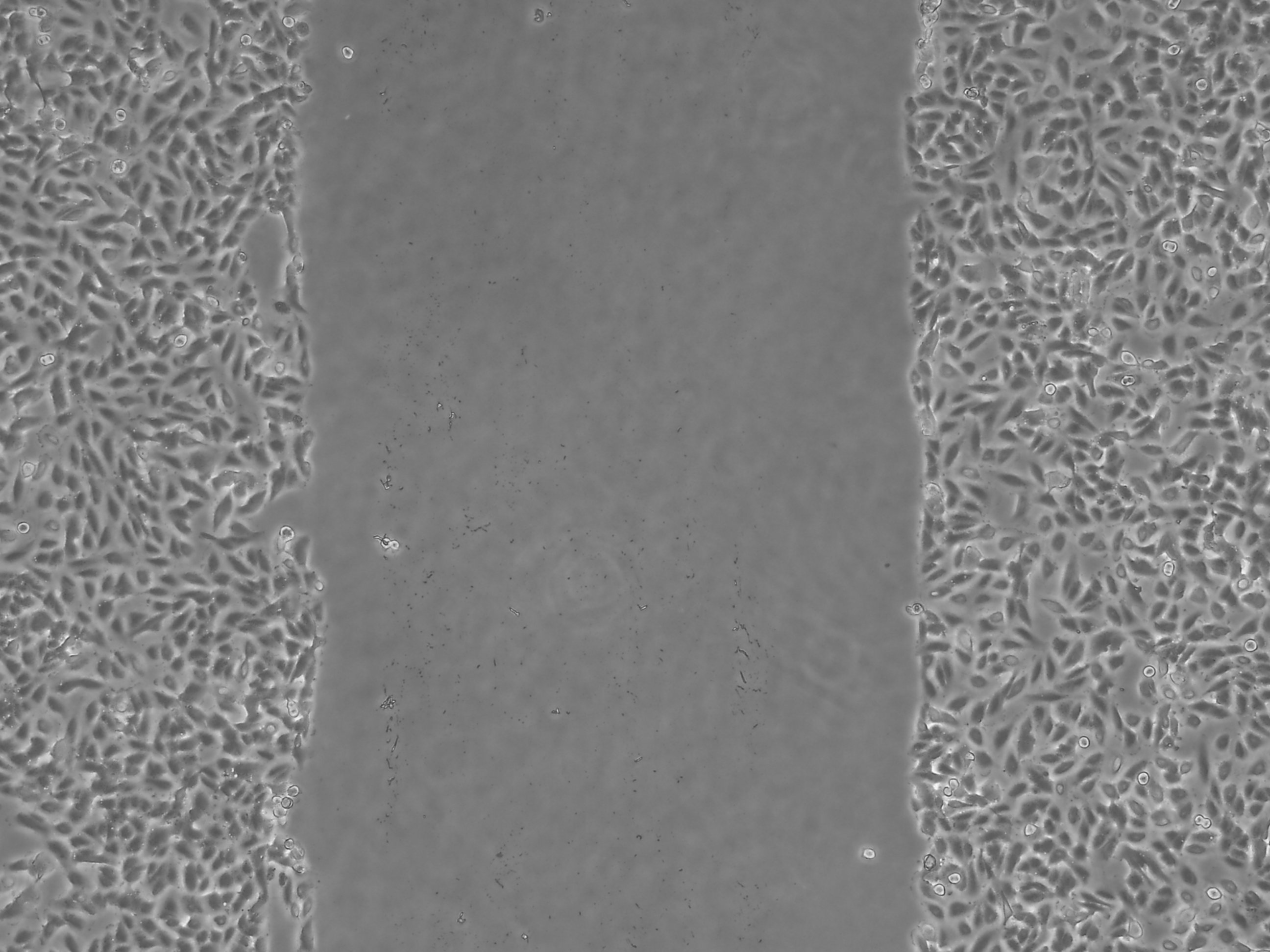

Supplement: Original Image for Figure 7A 0h Control_2.tif [file IENZ_A_2423875_SM5280.tif]
