# Supplementary material for: Discovery of novel 2,4-diarylaminopyrimidine hydrazone derivatives as potent anti-thyroid cancer agents capable of inhibiting FAK
Source: J Enzyme Inhib Med Chem. 2024 Nov 19;39(1):2423875. doi: 10.1080/14756366.2024.2423875 (PMC11578424; doi:10.1080/14756366.2024.2423875)
Supplement: Supporting information.docx [file IENZ_A_2423875_SM5355.docx]

**Discovery of novel 2,4-diarylaminopyrimidine hydrazone** **derivatives as potent anti-thyroid cancer agents capable of inhibiting FAK**

Hongting Li ^a,^ ^#,^ *, Mei-Qi Jia ^b, #^, Zhao-Long Qin ^c^, Changliang Lu, Weili Chu ^d^, Ze Zhang ^a^, Jinbo Niu ^a^, Jian Song ^b^, Sai-Yang Zhang ^b, d^, Lijun Fu*

1. Department of Thyroid Surgery, the First Affiliated Hospital of Zhengzhou University, the construction of east road, Erqi district, Zhengzhou,450052, Henan Province, China
2. School of Basic Medical Sciences, Zhengzhou University, Zhengzhou 450001, Henan Province, China
3. School of Pharmaceutical Sciences, Institute of Drug Discovery & Development Key, Laboratory of Advanced Drug Preparation Technologies (Ministry of Education), Zhengzhou University, Zhengzhou 450001, Henan Province, China
4. Zhengzhou Xingyuan Foreign Language High School, Zhengzhou 450045, Henan Province, China
5. Department of Respiratory and Critical Care Medicine, the First Affiliated Hospital of Zhengzhou University, the construction of east road, Erqi district, Zhengzhou,450052, Henan Province, China
6. The Third Affiliated Hospital of Zhengzhou University, Zhengzhou, 450052, China

* Corresponding author: Hong-Ting Li (tingting2003@126.com) and Lijun Fu (brease2003@163.com)

^#^ These authors contributed equally to this work

- ^1^H, ^13^C-NMR and HRMS of compound 14a


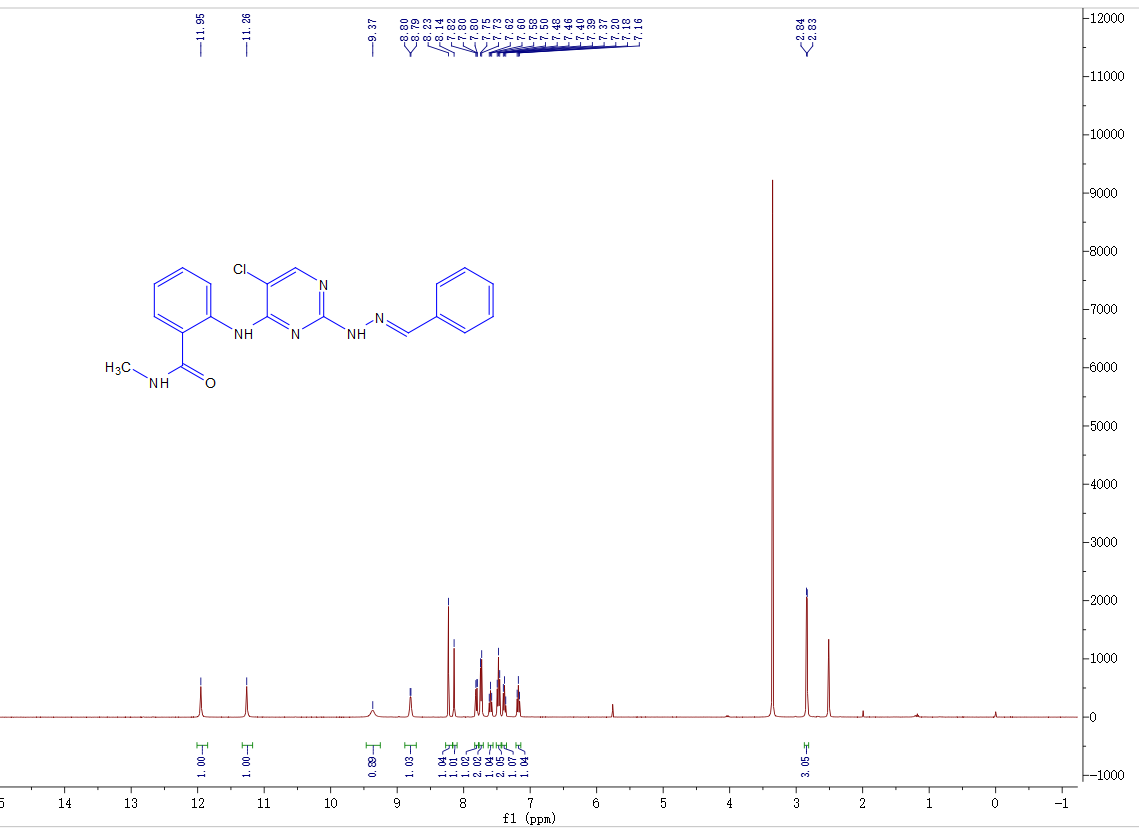


**Figure S1**. ^1^H NMR spectrum of compound 14a (400 MHz, DMSO-d6)


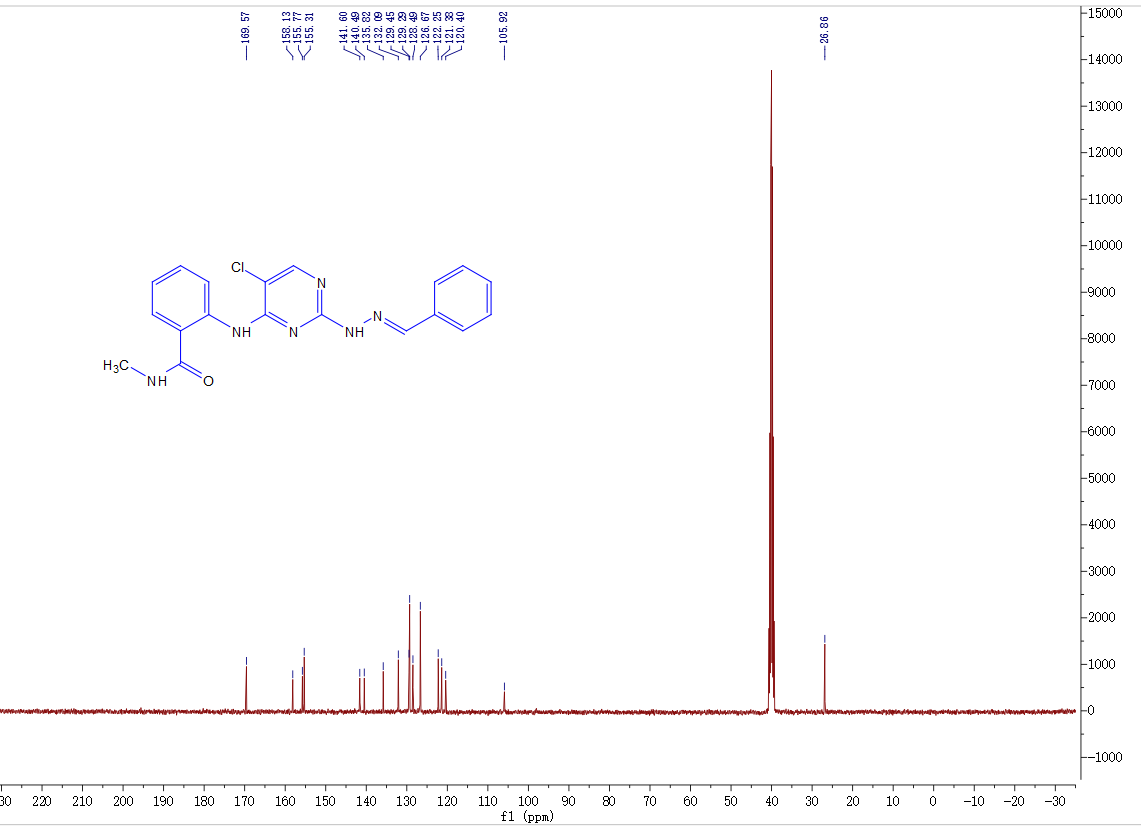


**Figure S2**. ^13^C NMR spectrum of compound 14a (100 MHz, DMSO-d6)


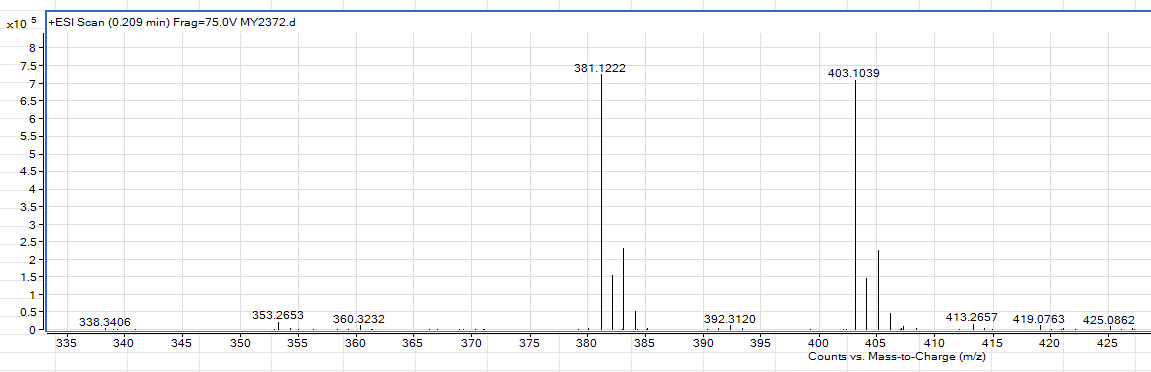


**Figure S3**. HRMS spectrum of compound 14a

- ^1^H, ^13^C-NMR and HRMS of compound 14b


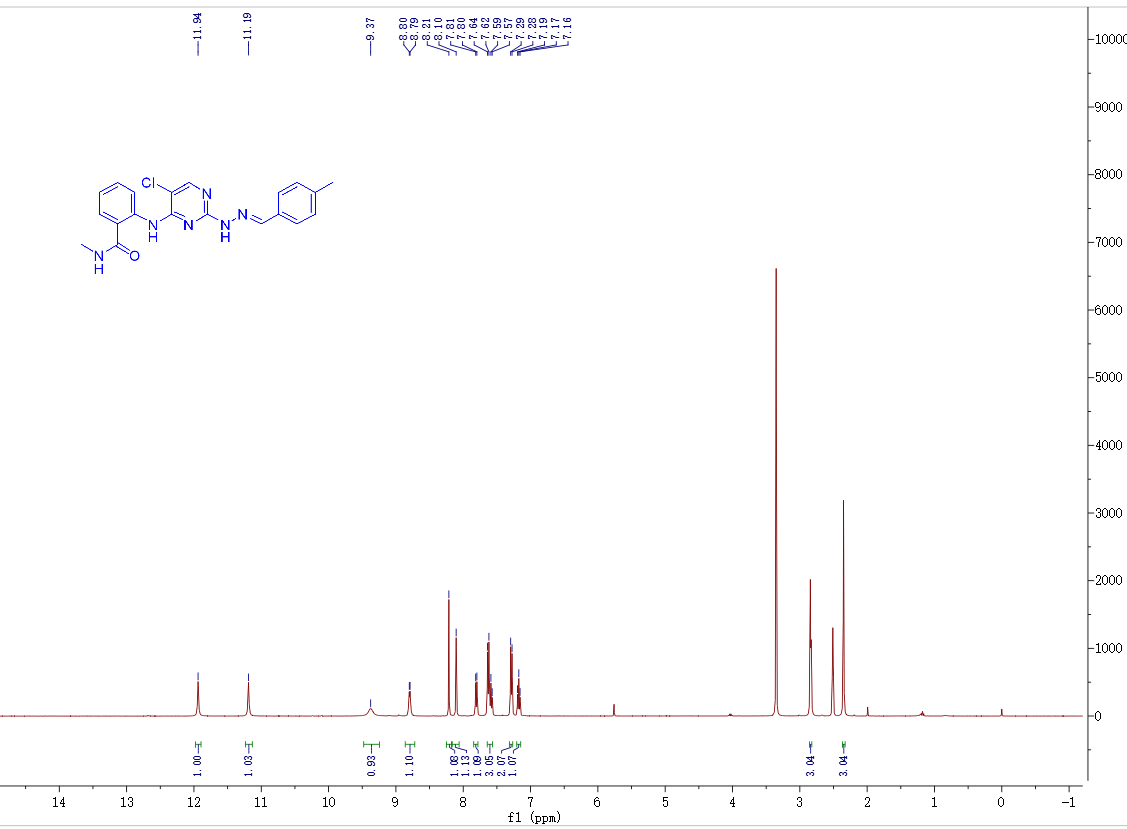


**Figure S4**. ^1^H NMR spectrum of compound 14b (400 MHz, DMSO-d6)


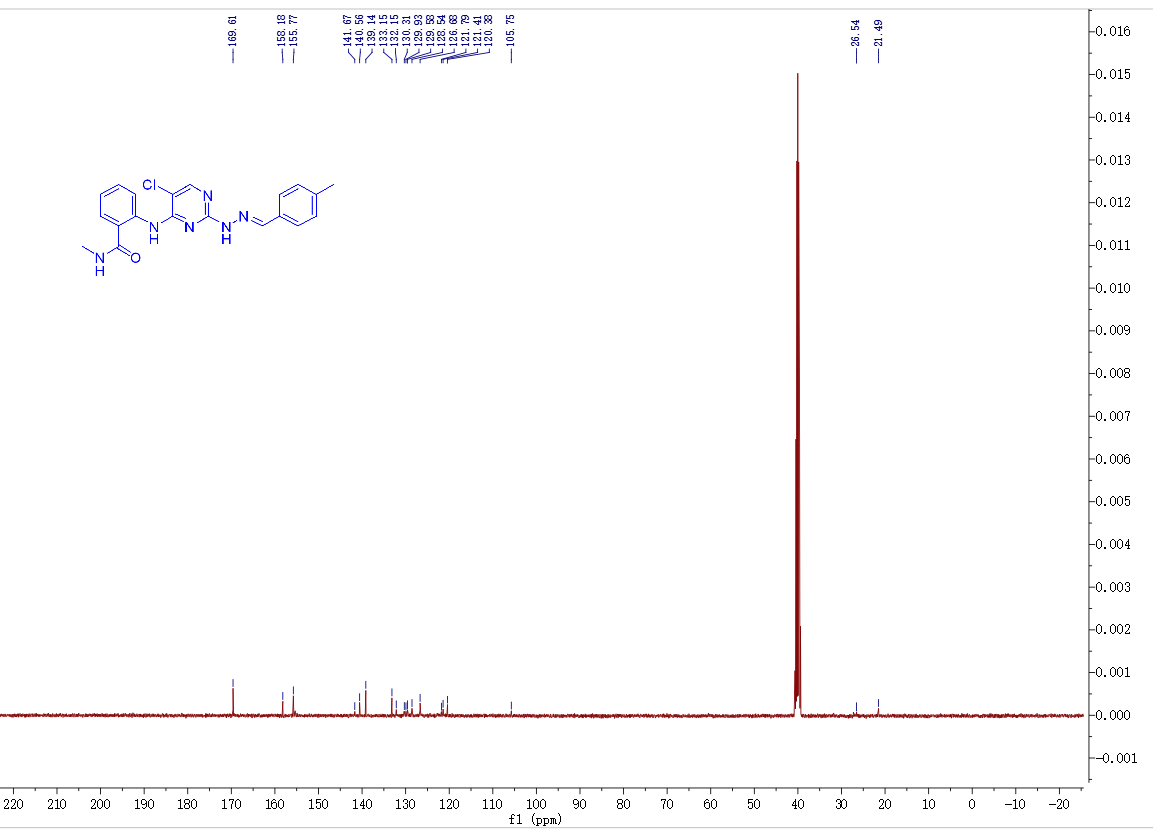


**Figure S5**. ^13^C NMR spectrum of compound 14b (100 MHz, DMSO-d6)


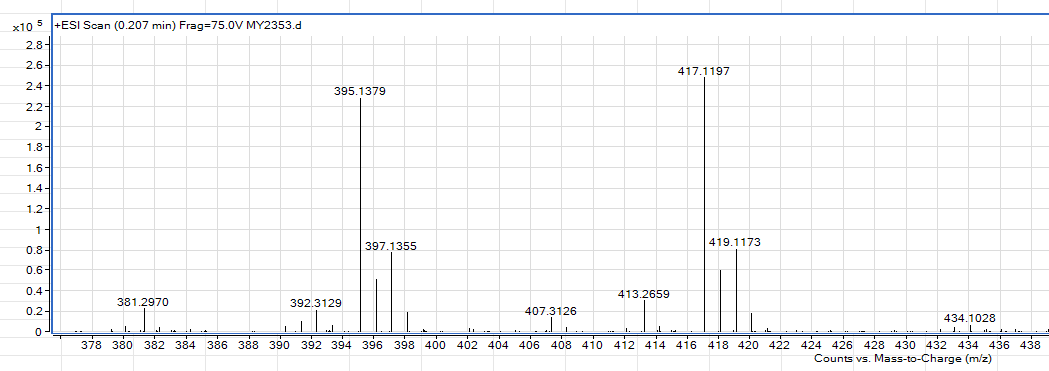


**Figure S6**. HRMS spectrum of compound 14b

- ^1^H, ^13^C-NMR and HRMS of compound 14c


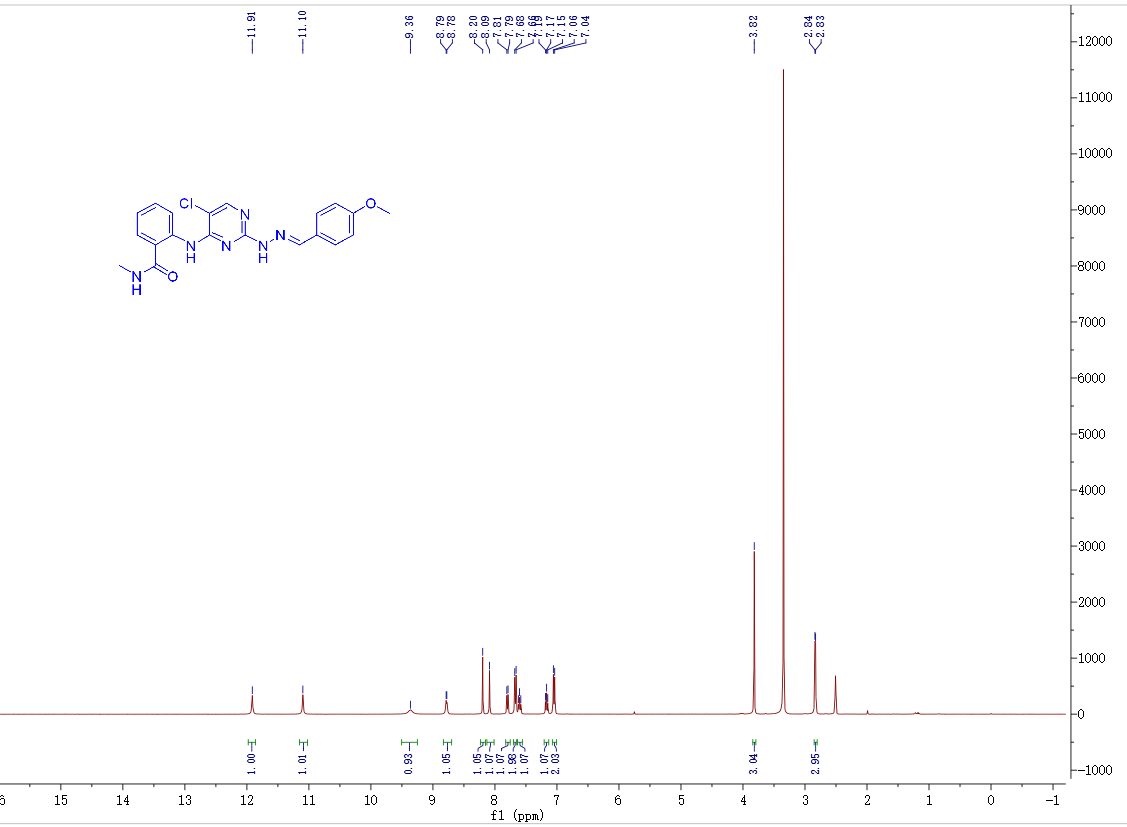


**Figure S7**. ^1^H NMR spectrum of compound 14c (400 MHz, DMSO-d6)


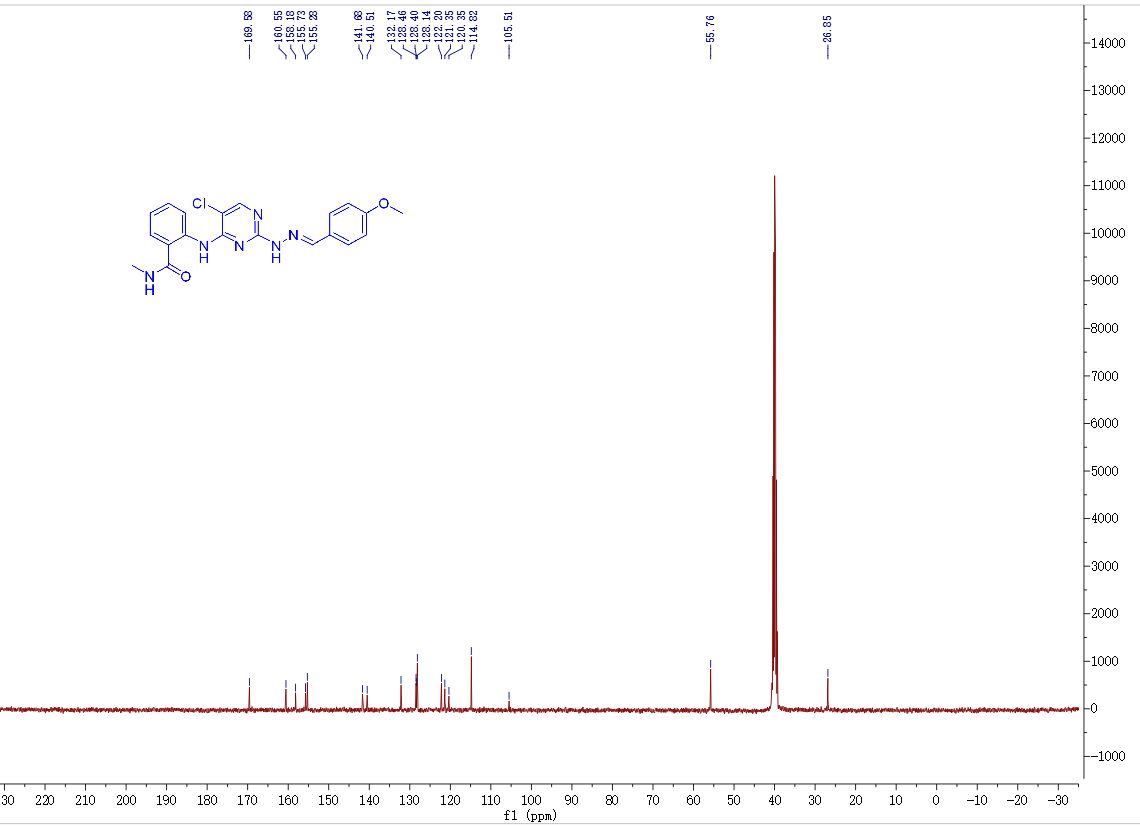


**Figure S8**. ^13^C NMR spectrum of compound 14c (100 MHz, DMSO-d6)


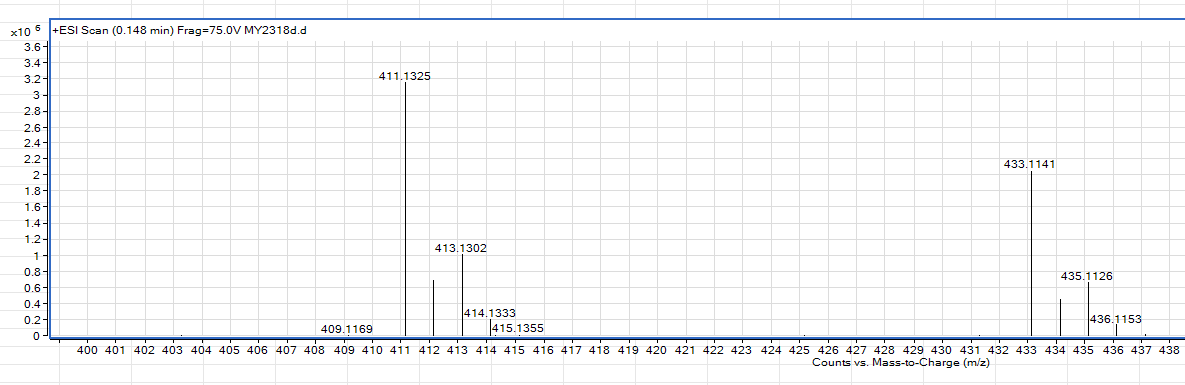


**Figure S9**. HRMS spectrum of compound 14c

- ^1^H, ^13^C-NMR and HRMS of compound 14d


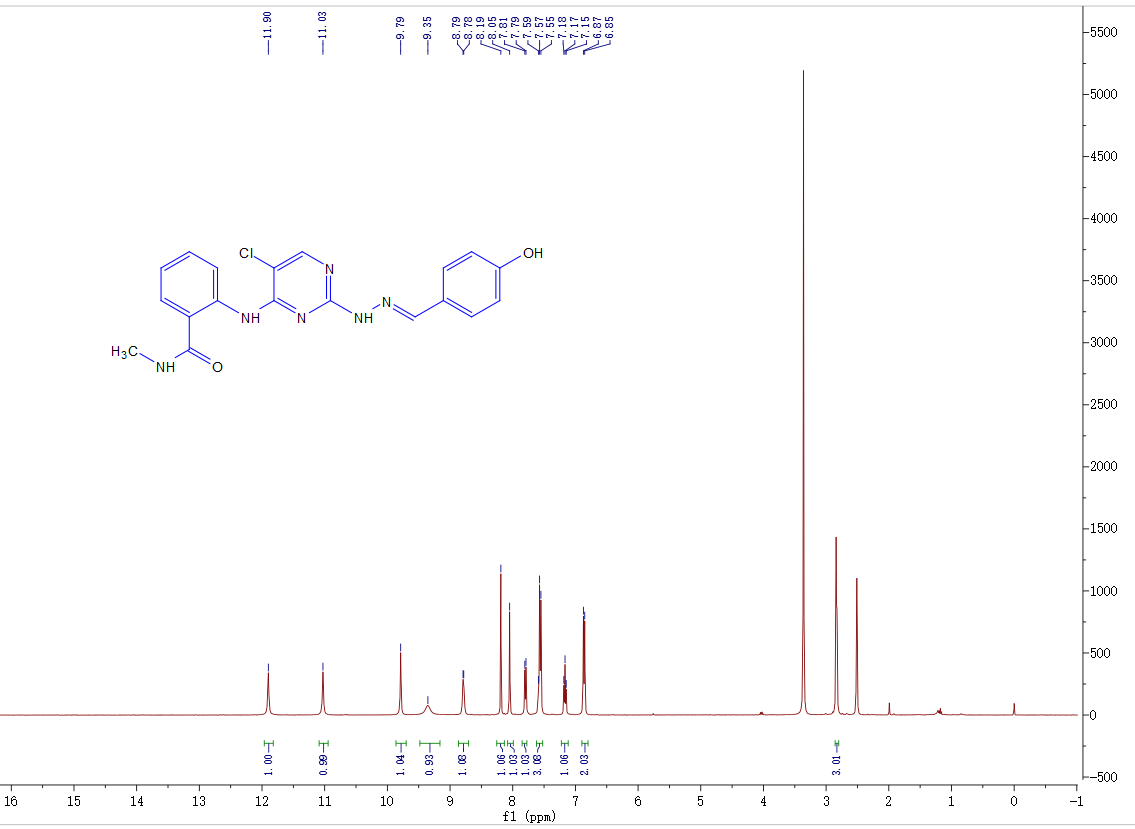


**Figure S10**. ^1^H NMR spectrum of compound 14d (400 MHz, DMSO-d6)


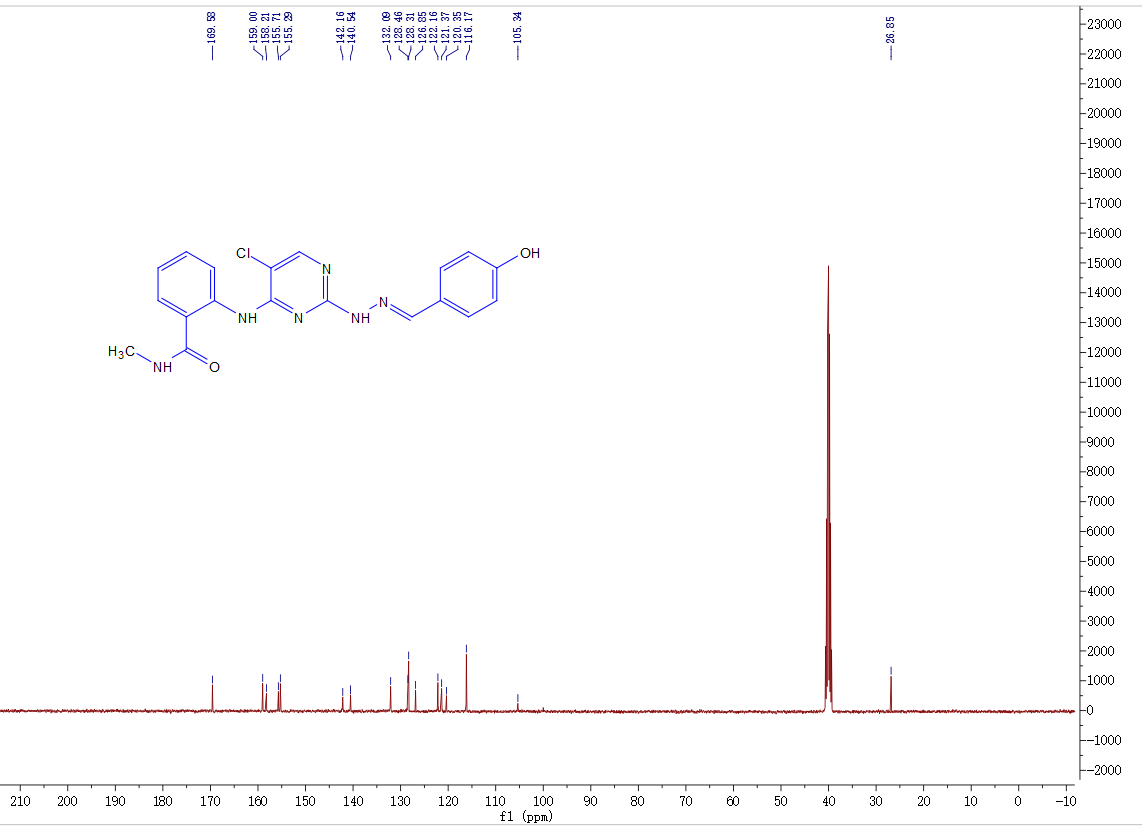


**Figure S11**. ^13^C NMR spectrum of compound 14d (100 MHz, DMSO-d6)


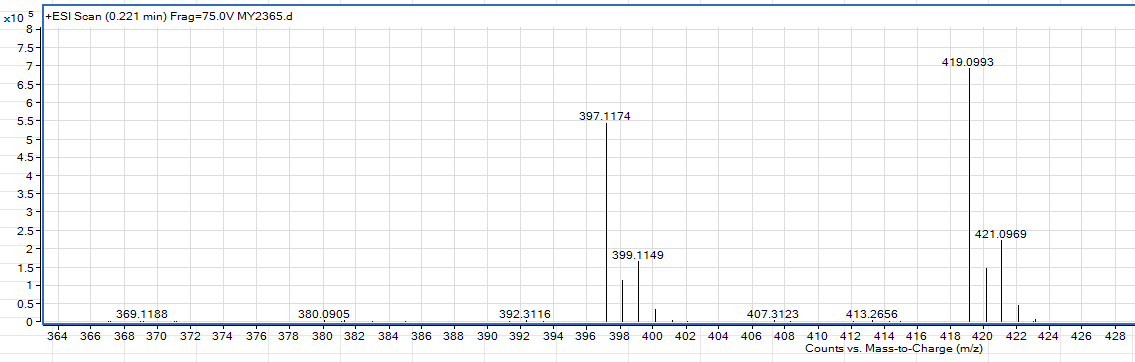


**Figure S12**. HRMS spectrum of compound 14d

- ^1^H, ^13^C-NMR and HRMS of compound 14e


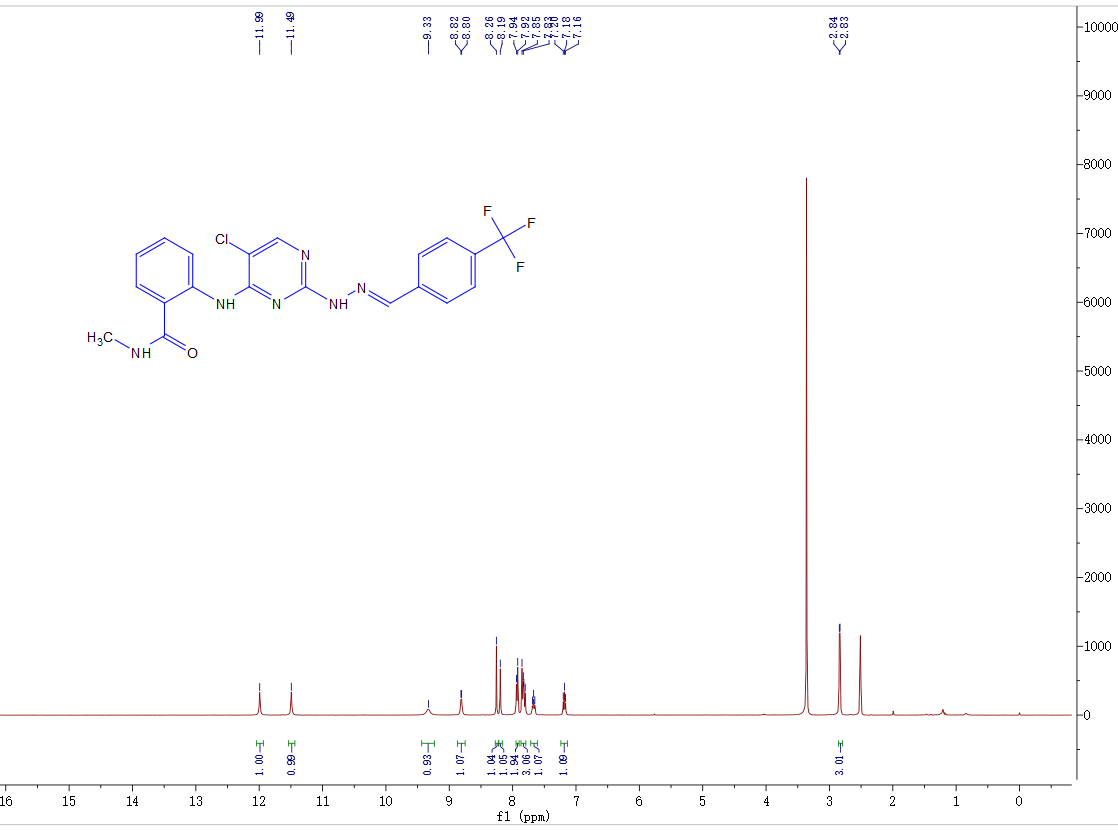


**Figure S13**. ^1^H NMR spectrum of compound 14e (400 MHz, DMSO-d6)


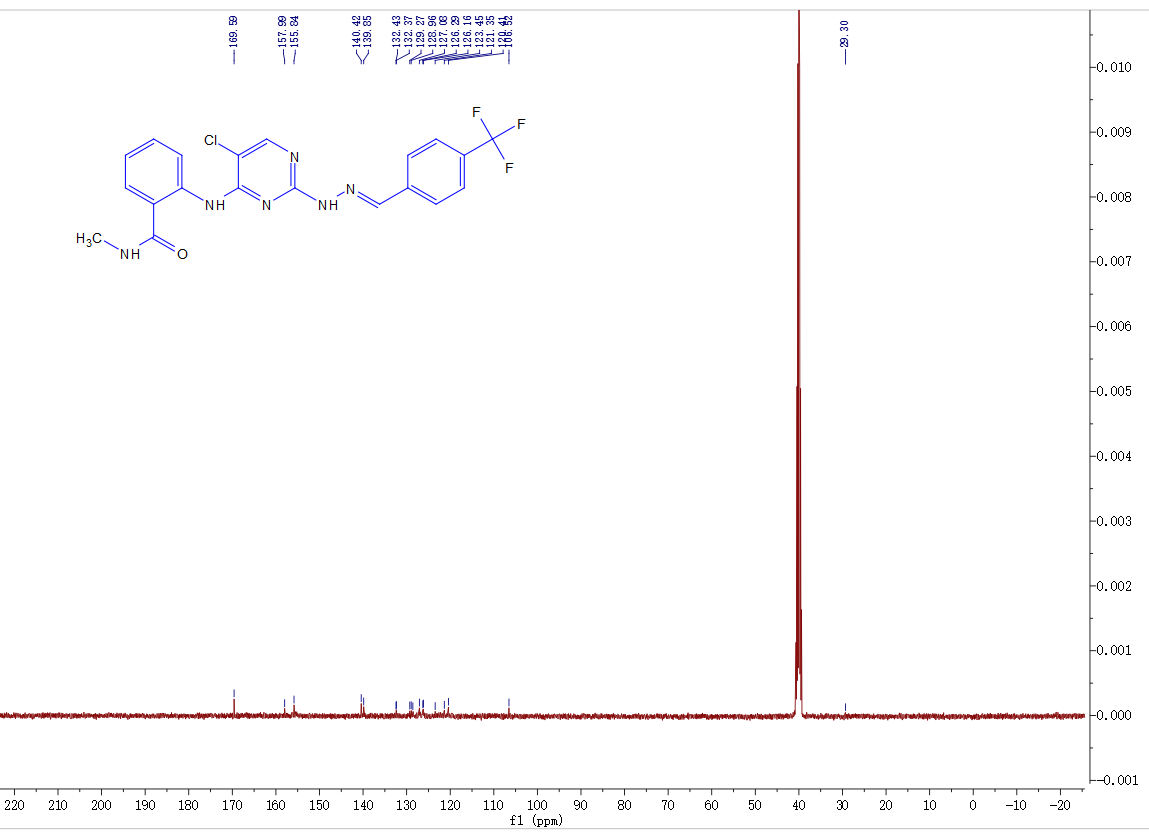


**Figure S14**. ^13^C NMR spectrum of compound 14e (100 MHz, DMSO-d6)


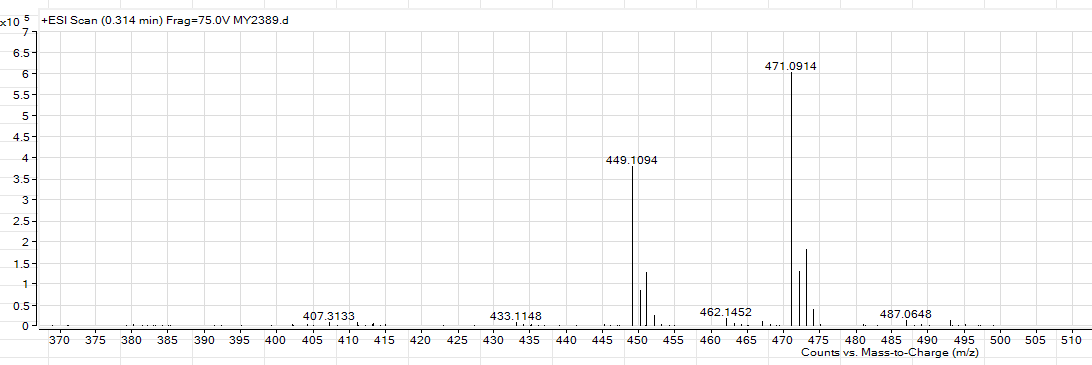


**Figure S15**. HRMS spectrum of compound 14e

- ^1^H, ^13^C-NMR and HRMS of compound 14f


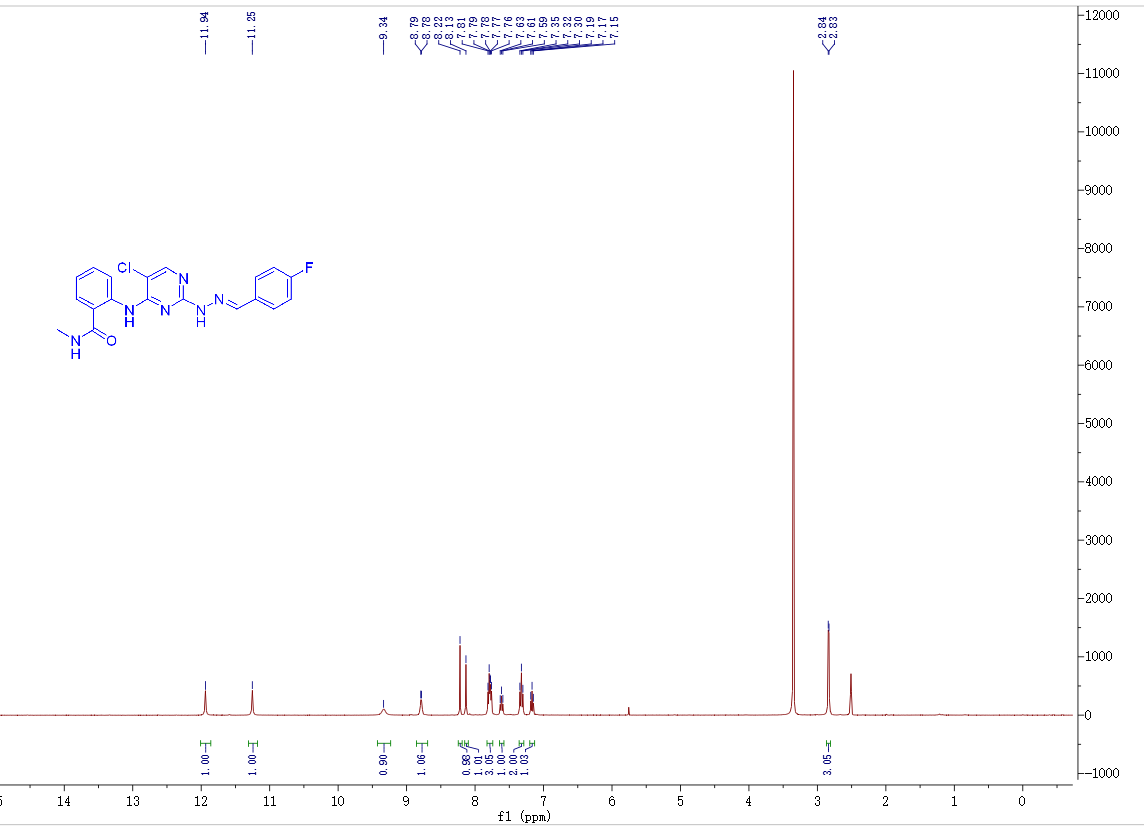


**Figure S16**. ^1^H NMR spectrum of compound 14f (400 MHz, DMSO-d6)


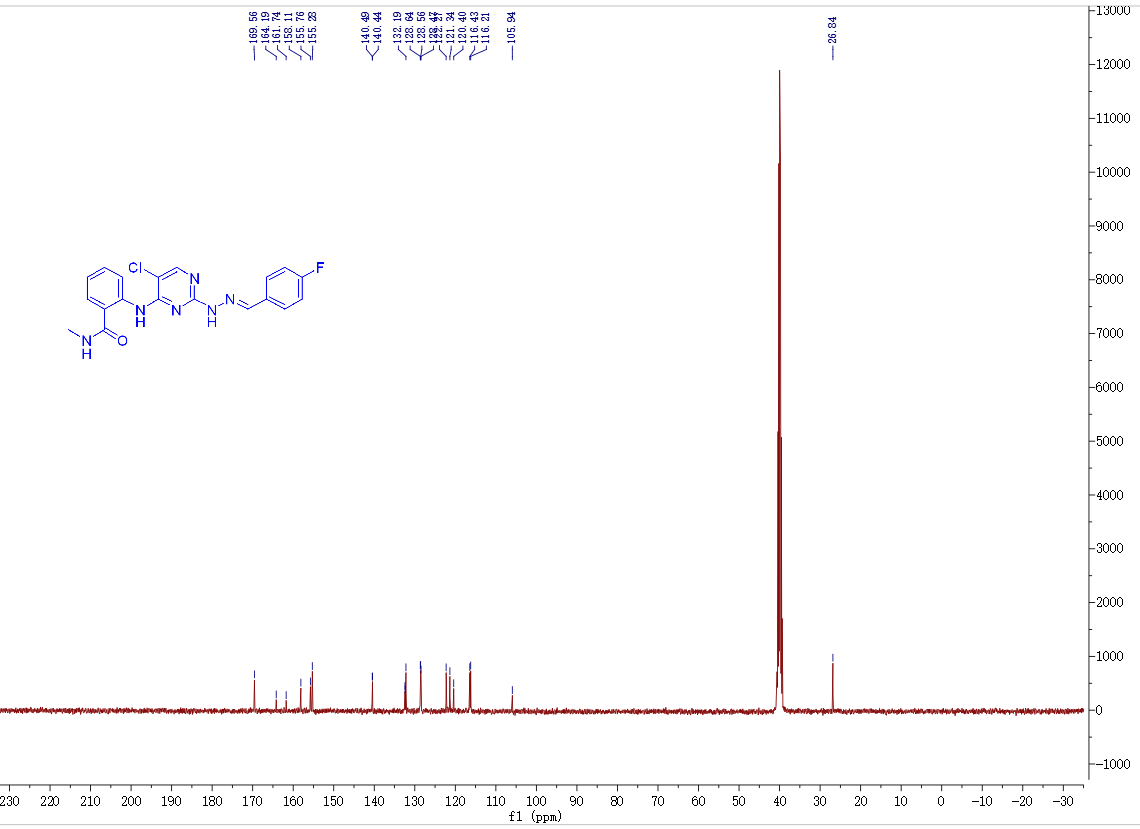


**Figure S17**. ^13^C NMR spectrum of compound 14f (100 MHz, DMSO-d6)


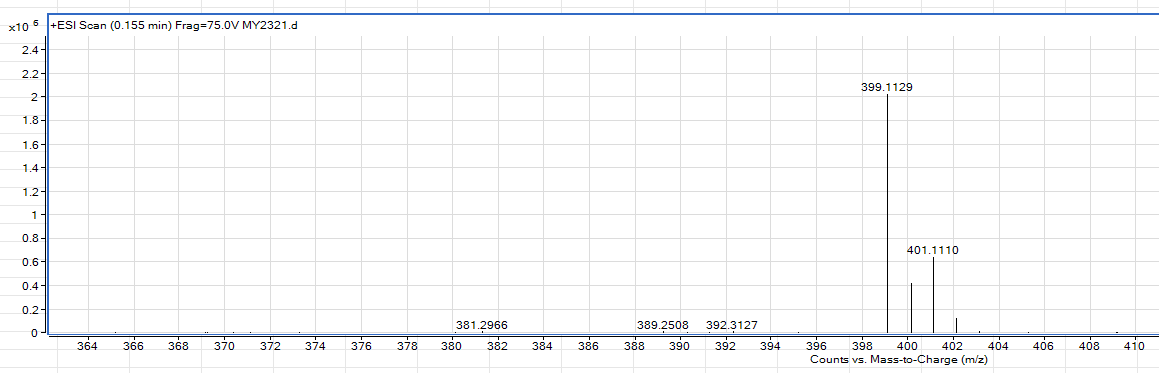


**Figure S18**. HRMS spectrum of compound 14f

- ^1^H, ^13^C-NMR and HRMS of compound 14g


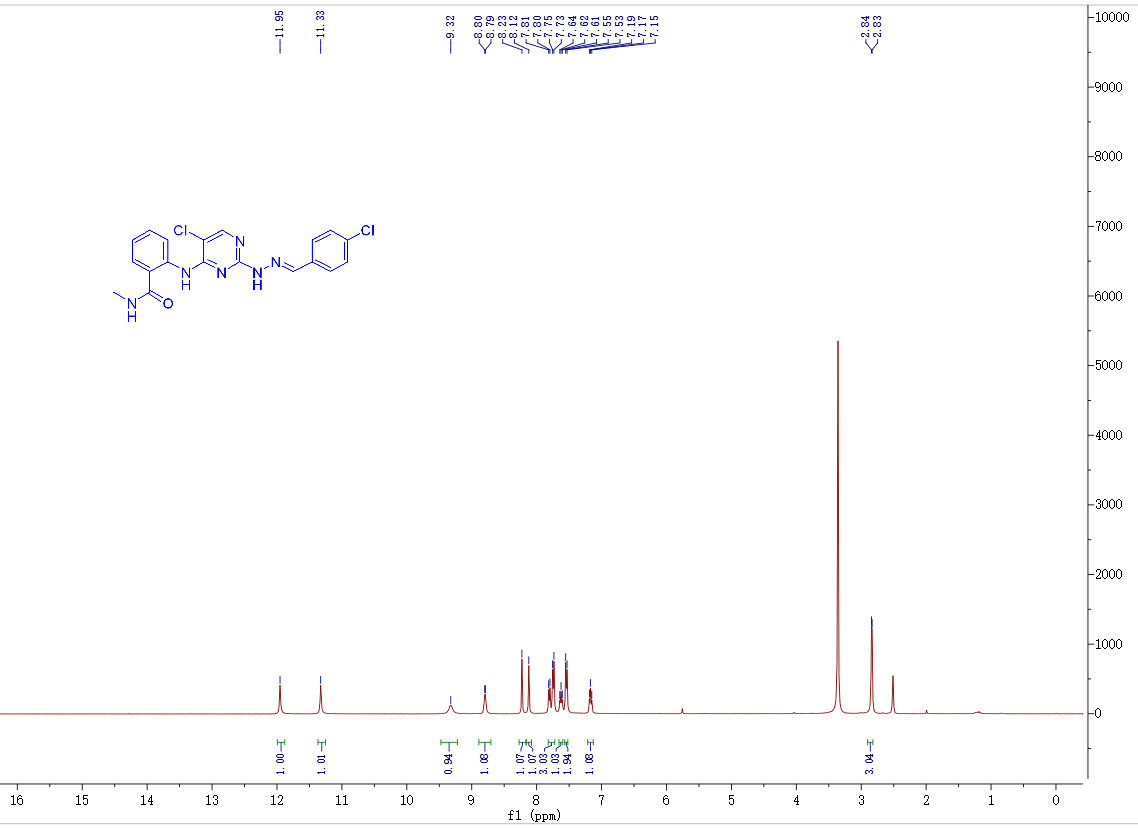


**Figure S19**. ^1^H NMR spectrum of compound 14g (400 MHz, DMSO-d6)


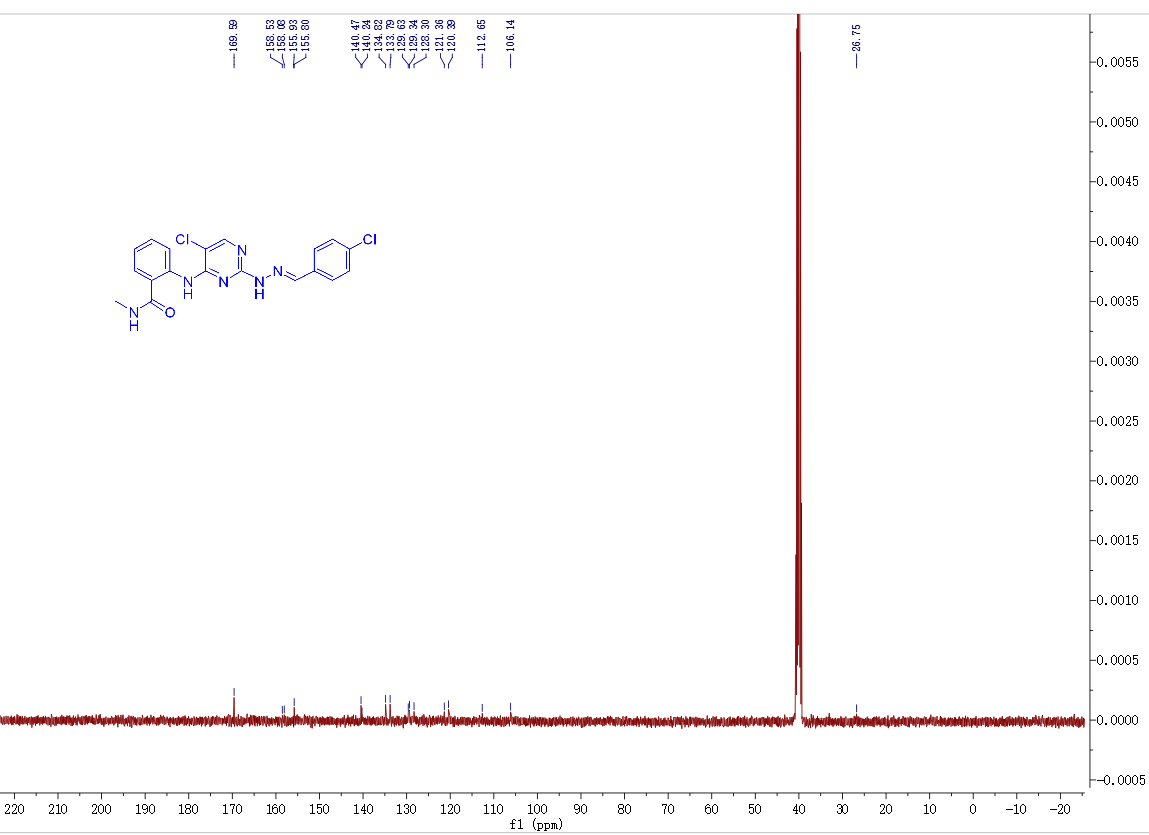


**Figure S20**. ^13^C NMR spectrum of compound 14g (100 MHz, DMSO-d6)


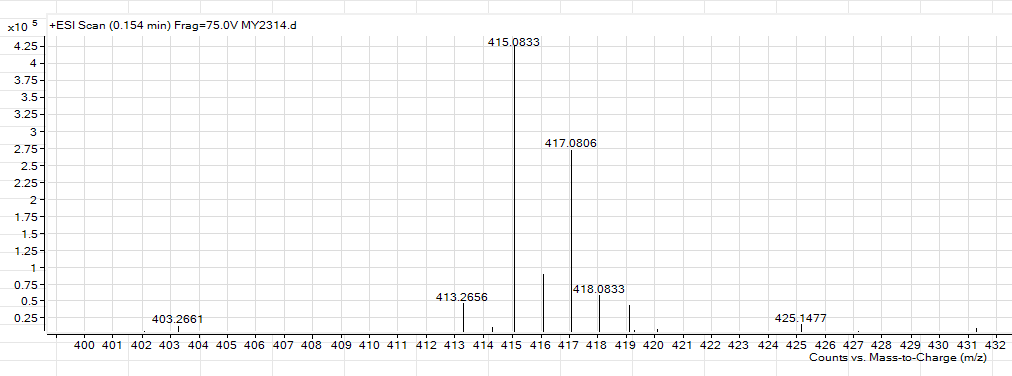


**Figure S21**. HRMS spectrum of compound 14g

- ^1^H, ^13^C-NMR and HRMS of compound 14h


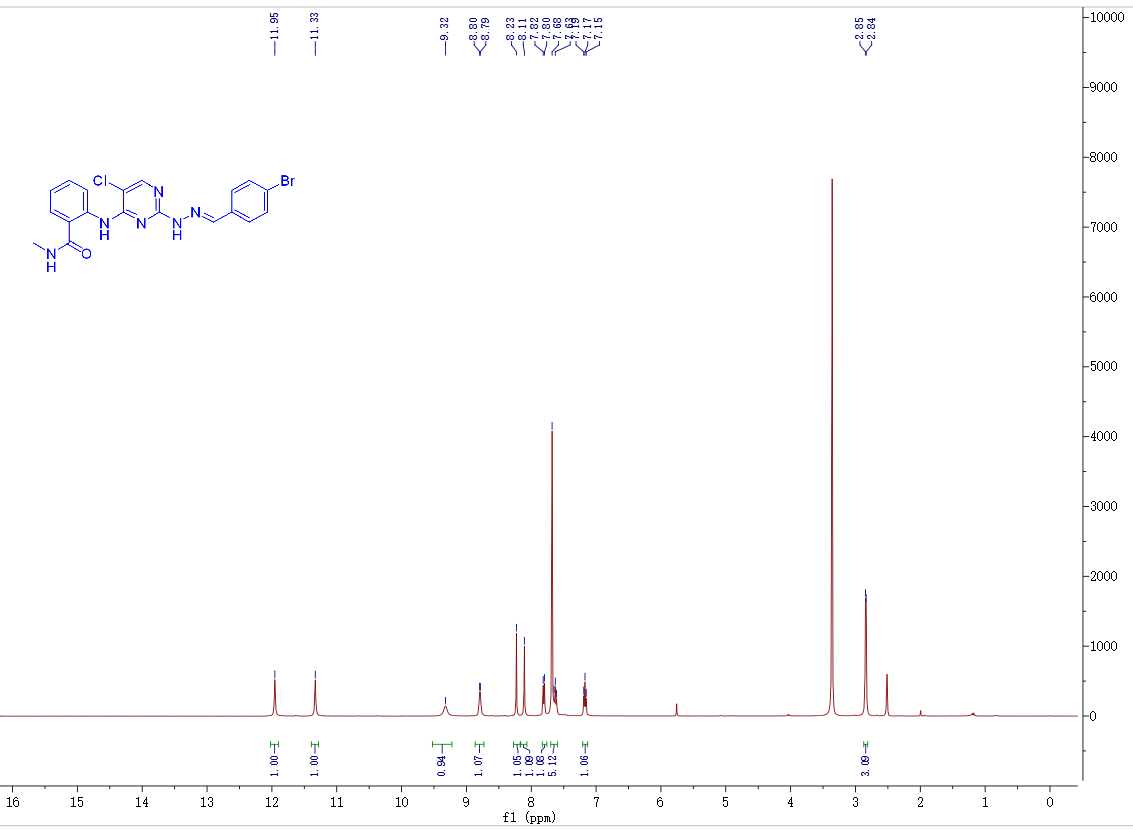


**Figure S22**. ^1^H NMR spectrum of compound 14h (400 MHz, DMSO-d6)


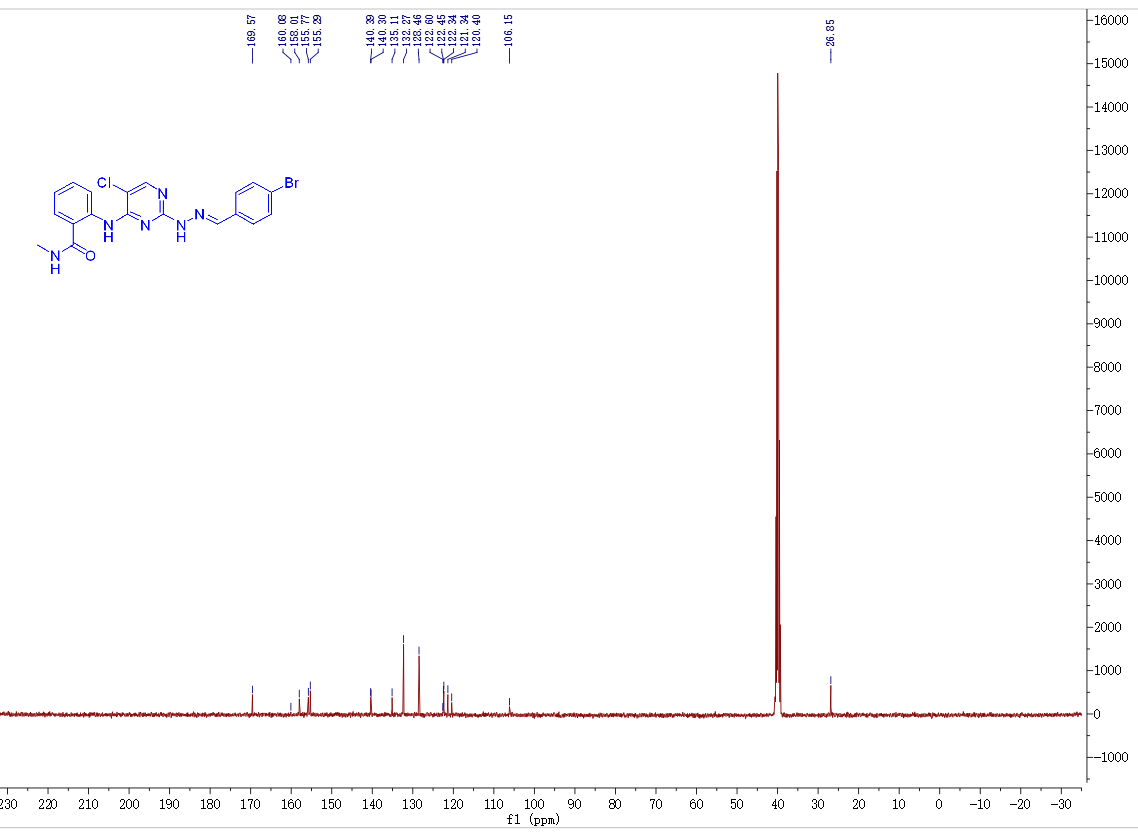


**Figure S23**. ^13^C NMR spectrum of compound 14h (100 MHz, DMSO-d6)


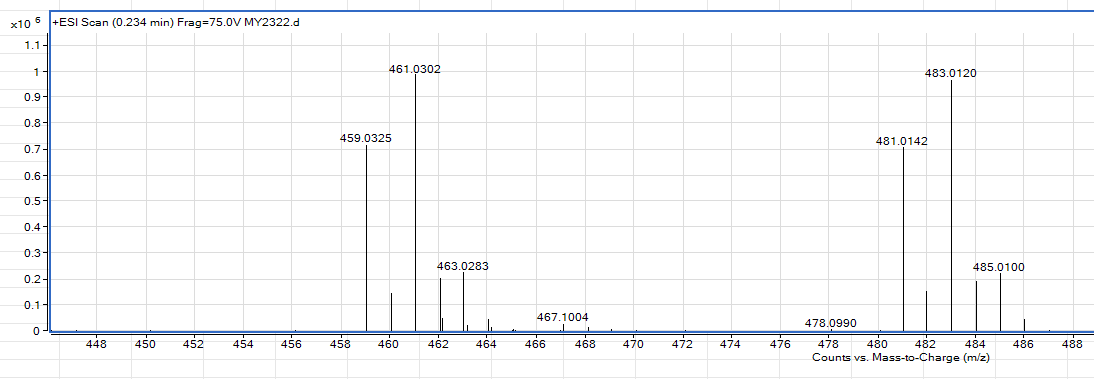


**Figure S24**. HRMS spectrum of compound 14h

- ^1^H, ^13^C-NMR and HRMS of compound 14i


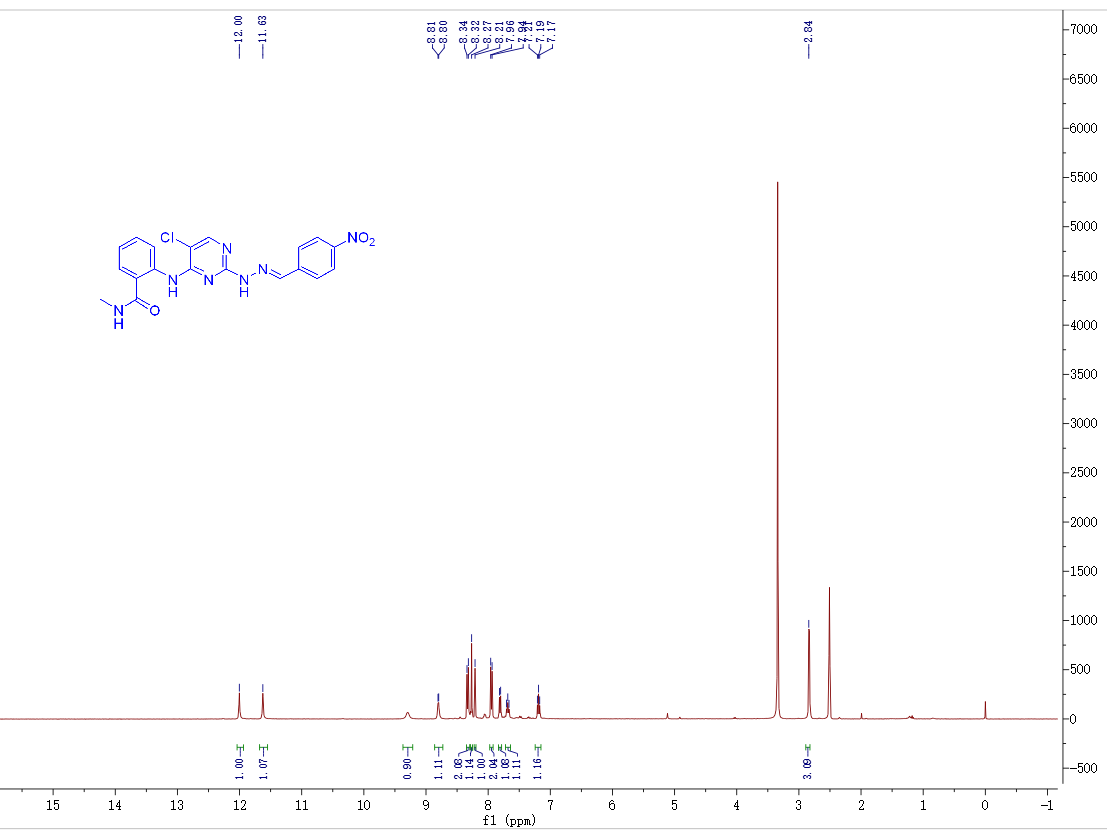


**Figure S25**. ^1^H NMR spectrum of compound 14i (400 MHz, DMSO-d6)


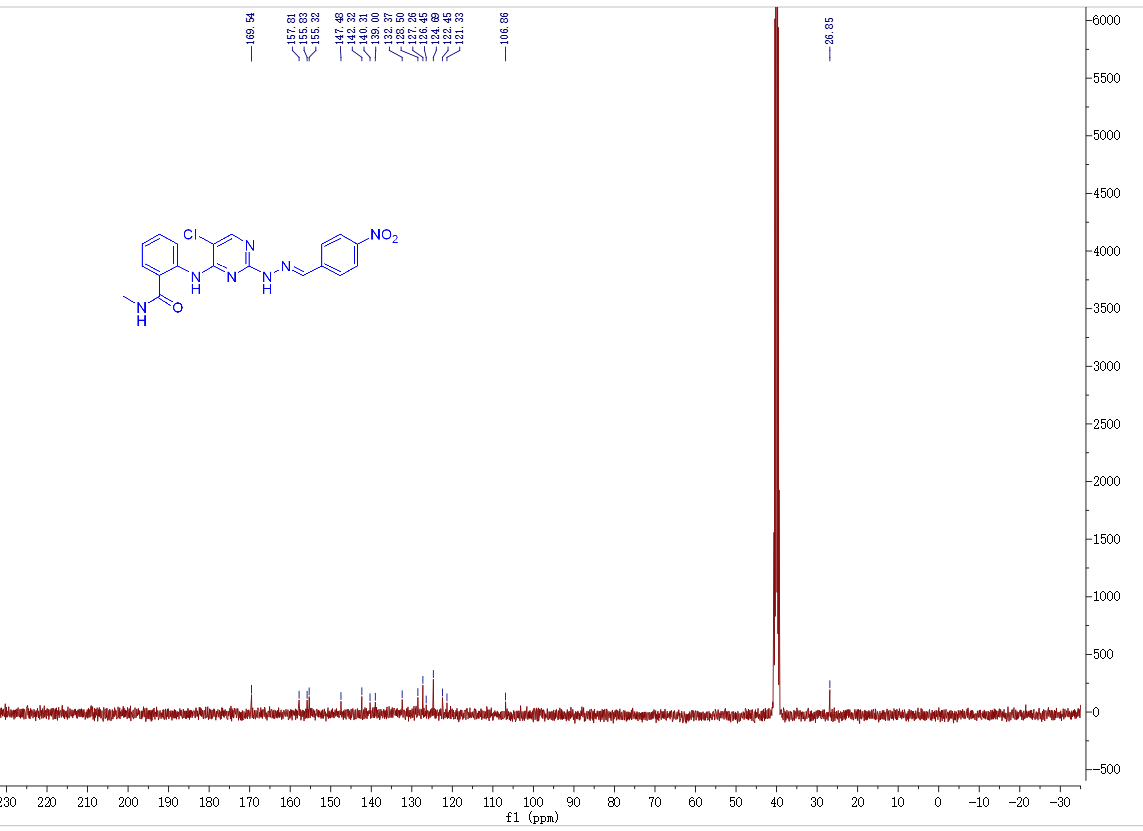


**Figure S26**. ^13^C NMR spectrum of compound 14i (100 MHz, DMSO-d6)


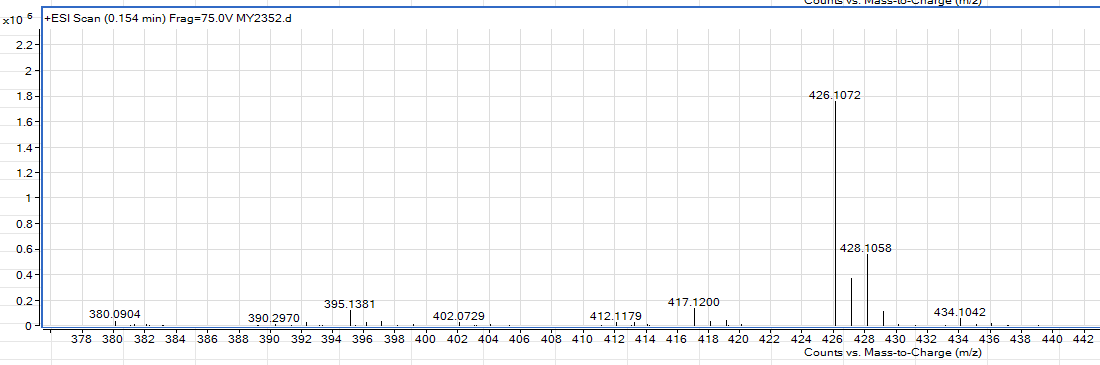


**Figure S27**. HRMS spectrum of compound 14i

- ^1^H, ^13^C-NMR and HRMS of compound 14j


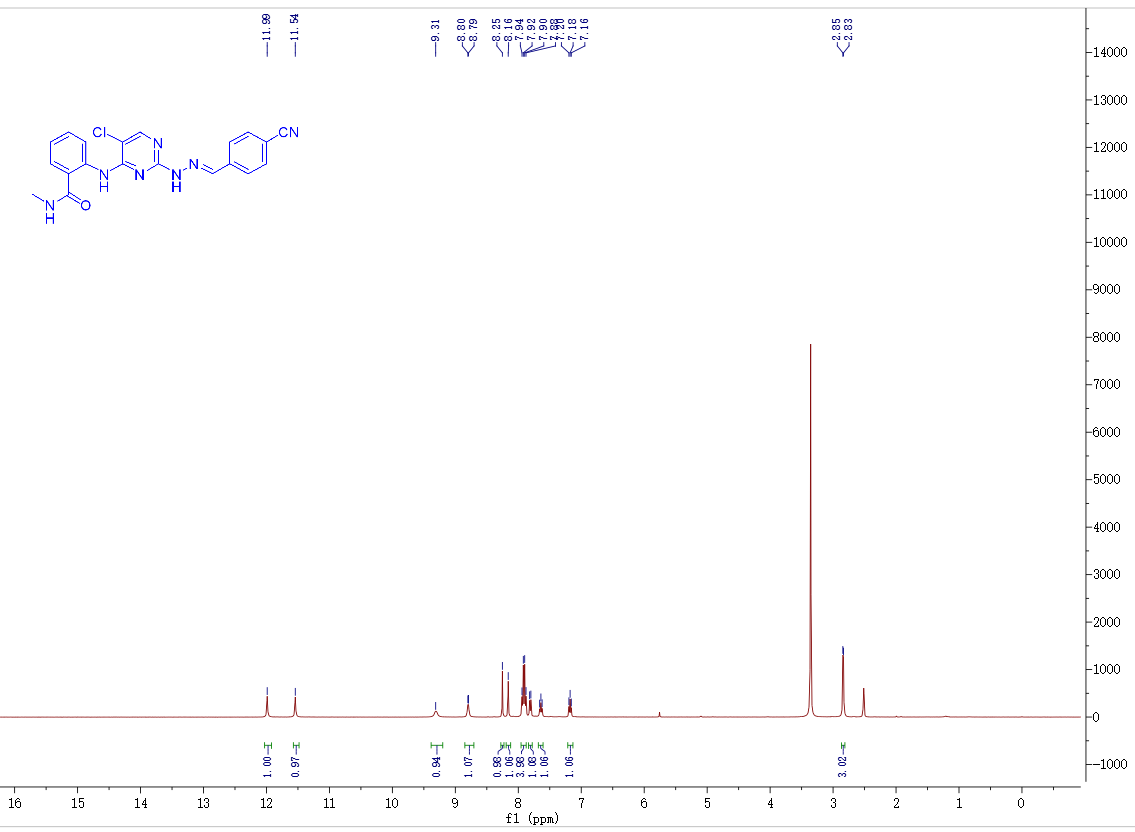


**Figure S28**. ^1^H NMR spectrum of compound 14j (400 MHz, DMSO-d6)


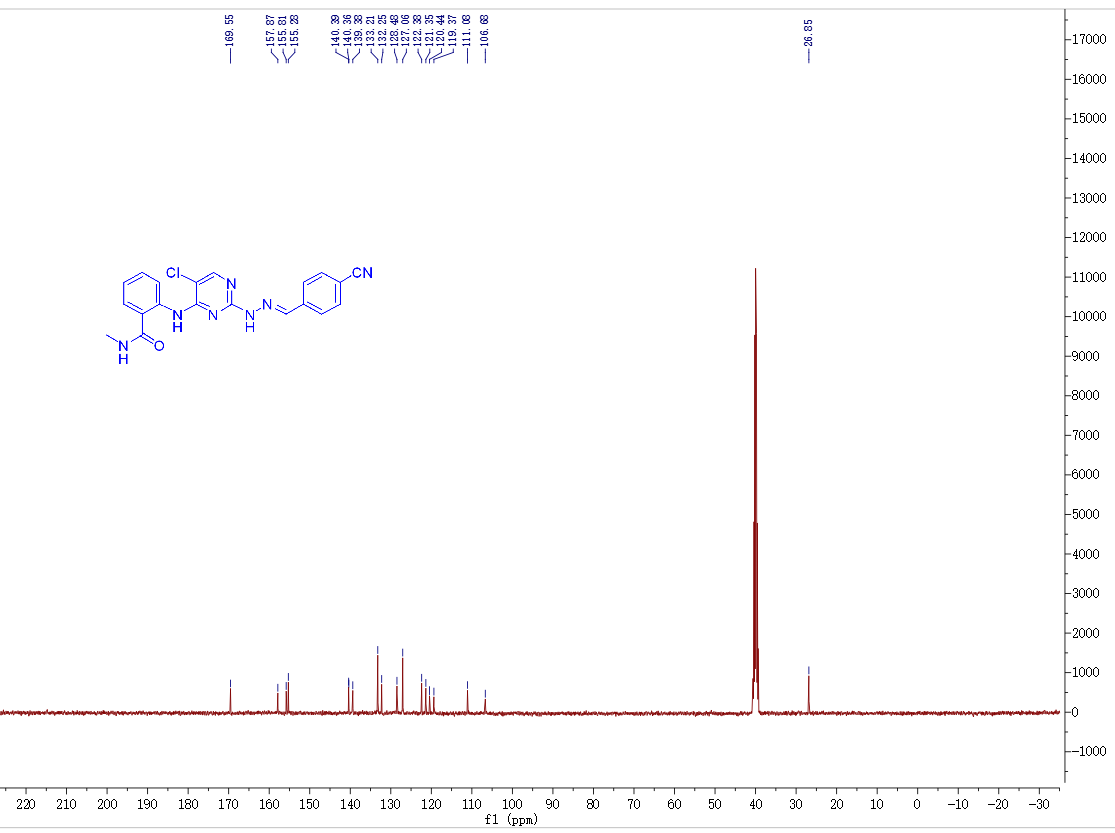


**Figure S29**. ^13^C NMR spectrum of compound 14j (100 MHz, DMSO-d6)


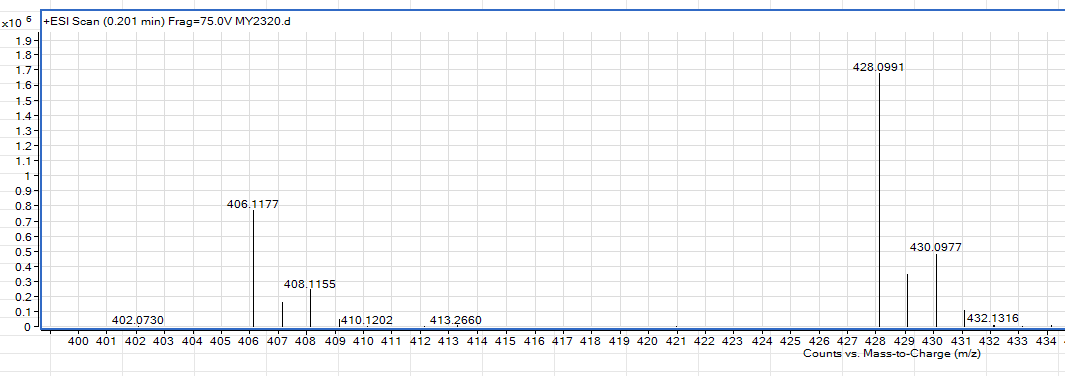


**Figure S30**. HRMS spectrum of compound 14j

- ^1^H, ^13^C-NMR and HRMS of compound 14k


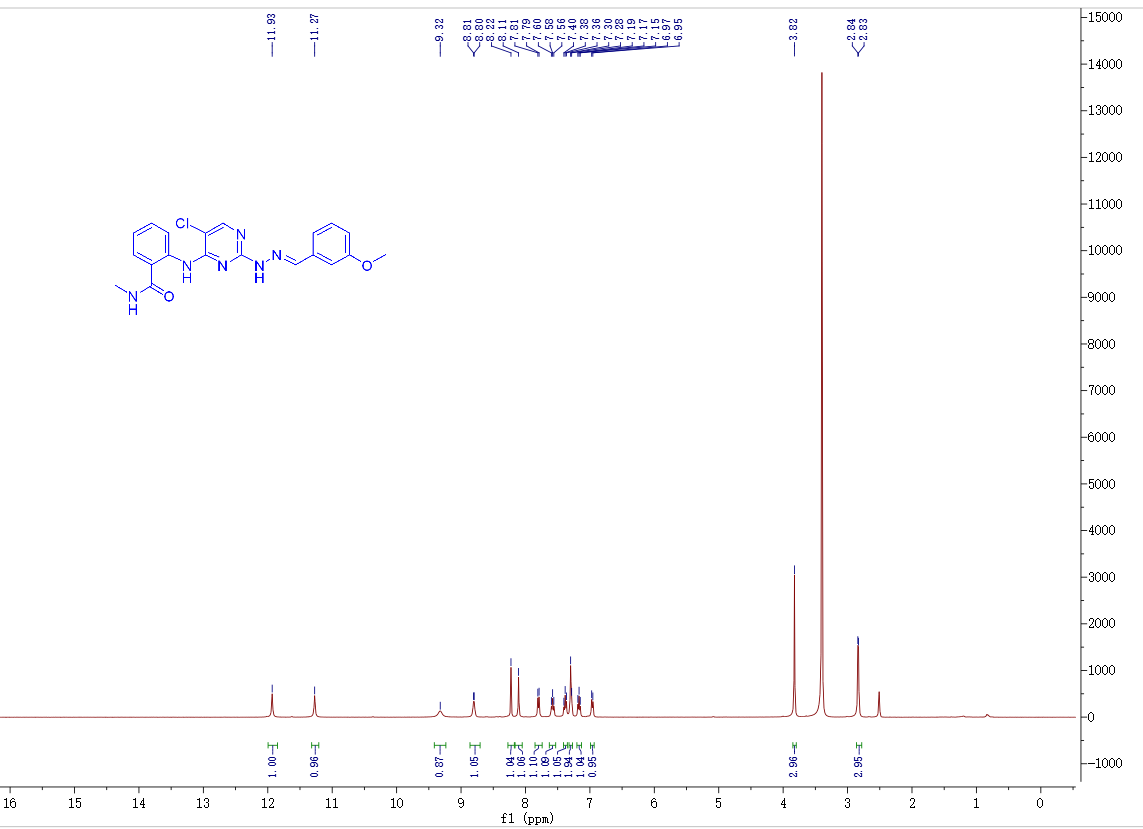


**Figure S31**. ^1^H NMR spectrum of compound 14k (400 MHz, DMSO-d6)


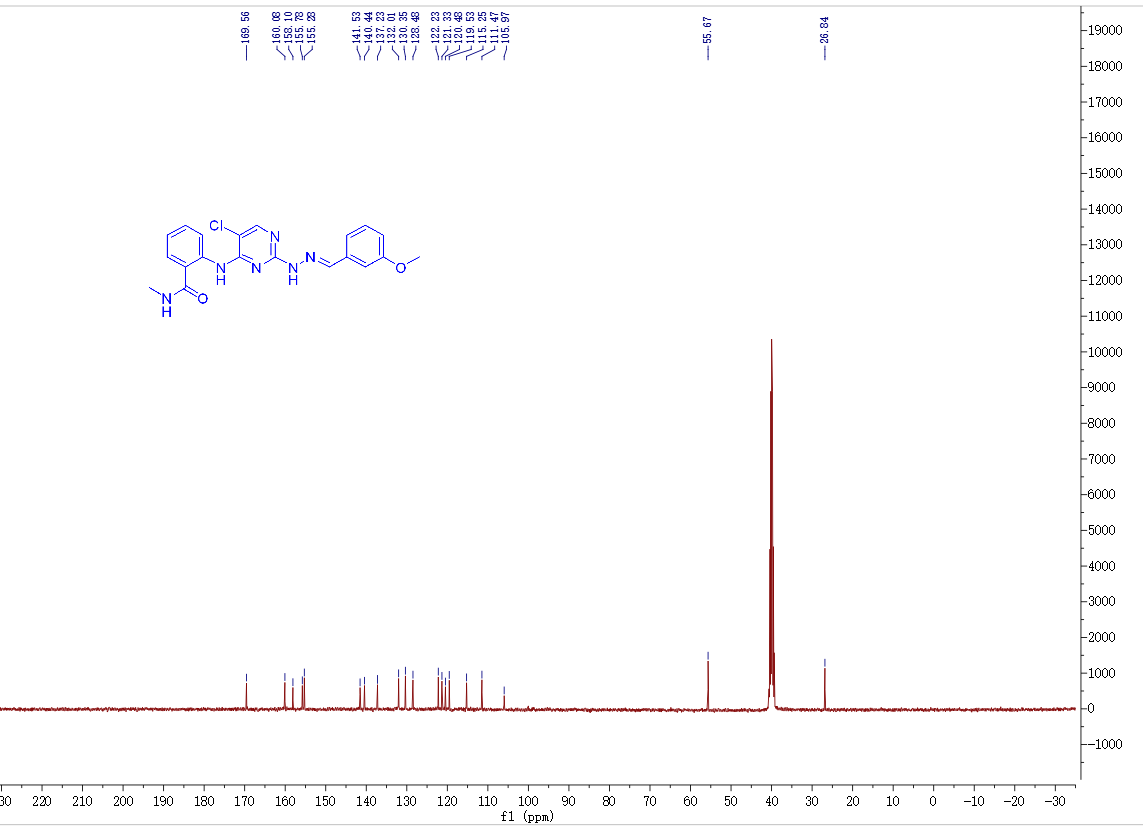


**Figure S32**. ^13^C NMR spectrum of compound 14k (100 MHz, DMSO-d6)


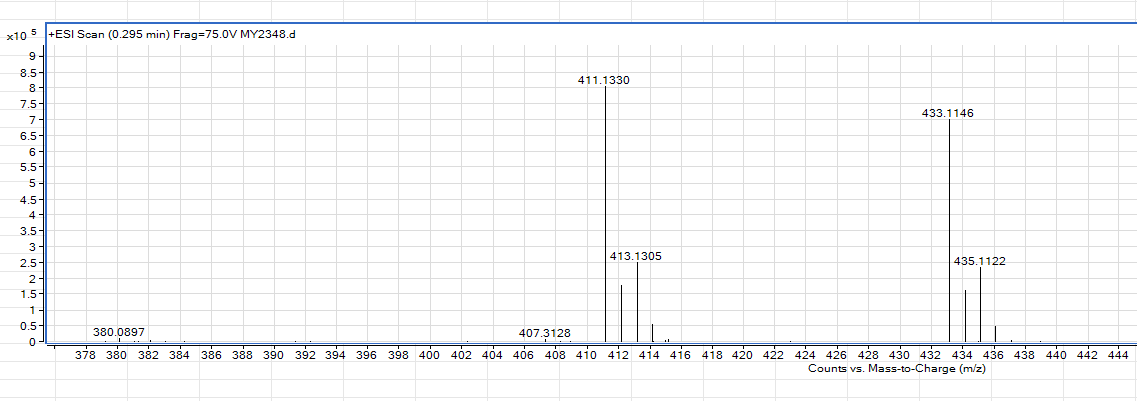


**Figure S33**. HRMS spectrum of compound 14k

- ^1^H, ^13^C-NMR and HRMS of compound 14l


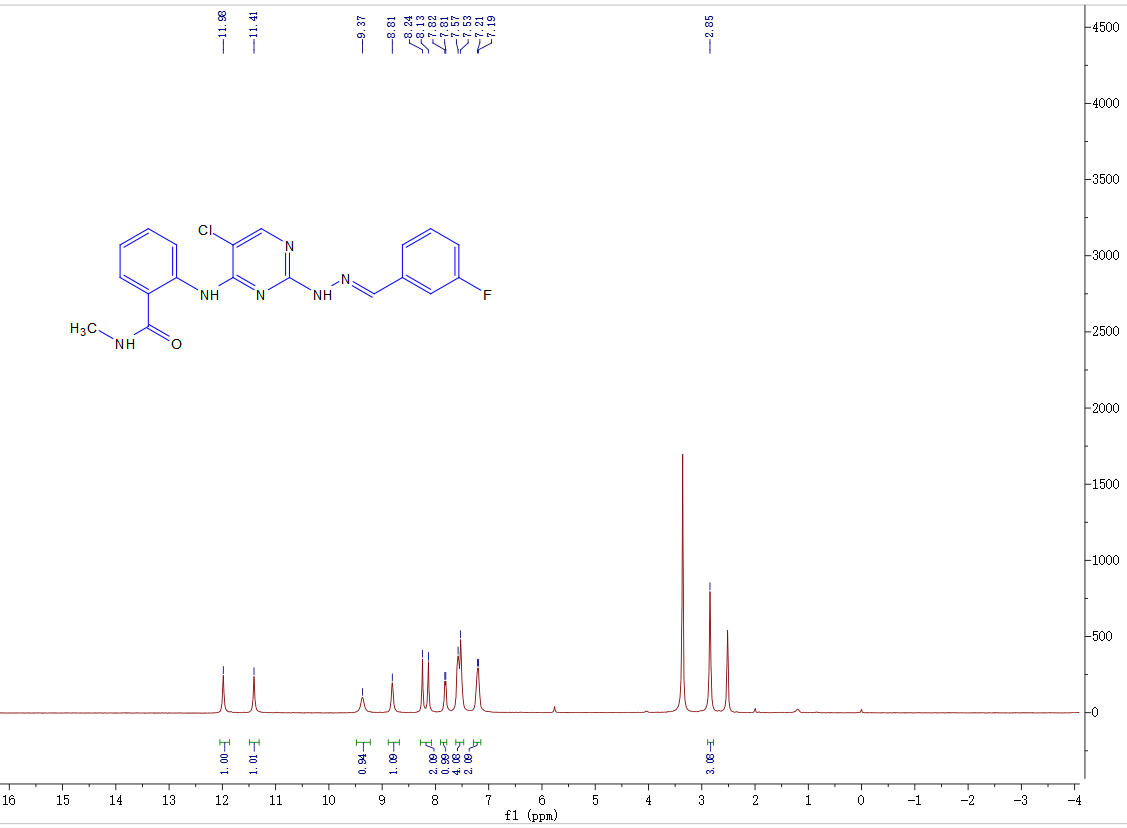


**Figure S34**. ^1^H NMR spectrum of compound 14l (400 MHz, DMSO-d6)


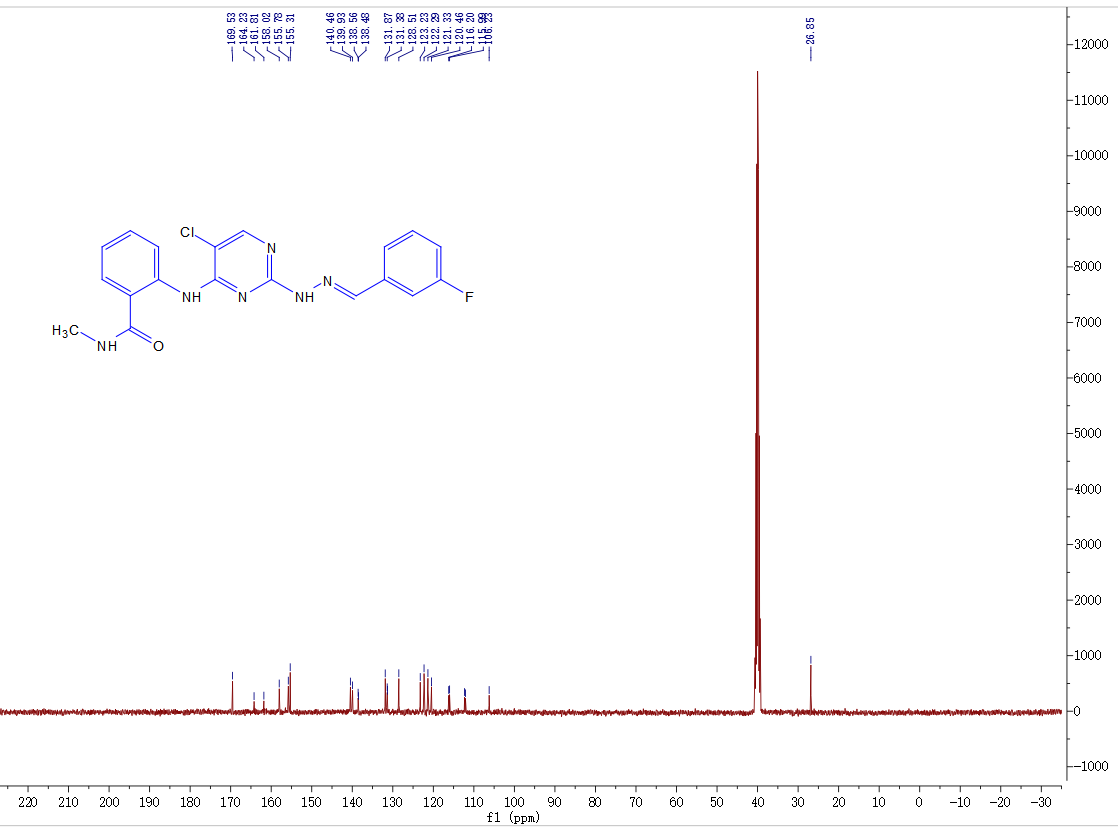


**Figure S35**. ^13^C NMR spectrum of compound 14l (100 MHz, DMSO-d6)


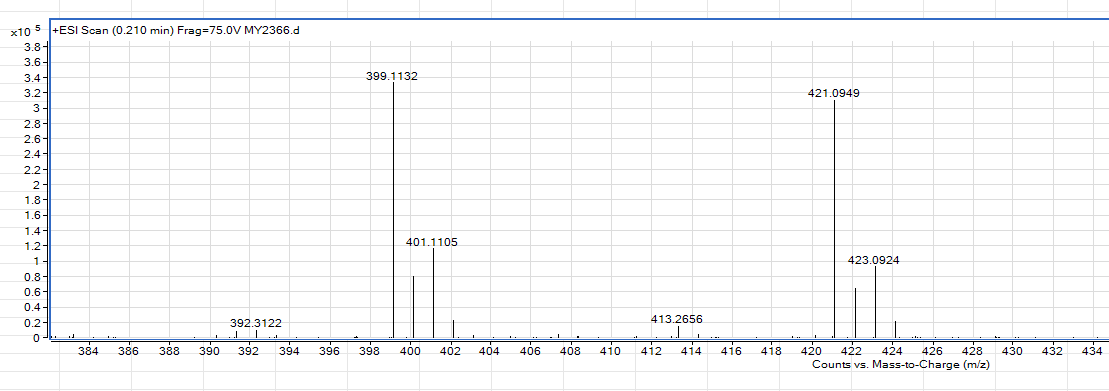


**Figure S36**. HRMS spectrum of compound 14l

- ^1^H, ^13^C-NMR and HRMS of compound 14m


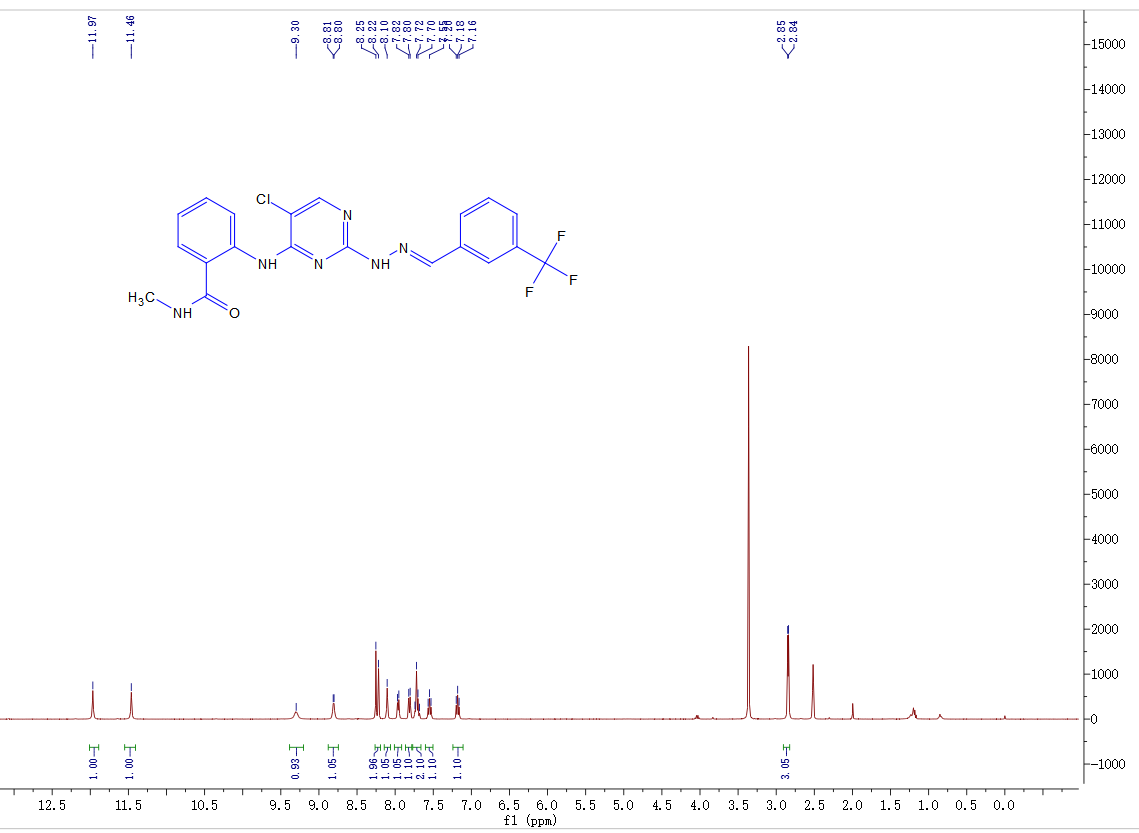


**Figure S37**. ^1^H NMR spectrum of compound 14m (400 MHz, DMSO-d6)


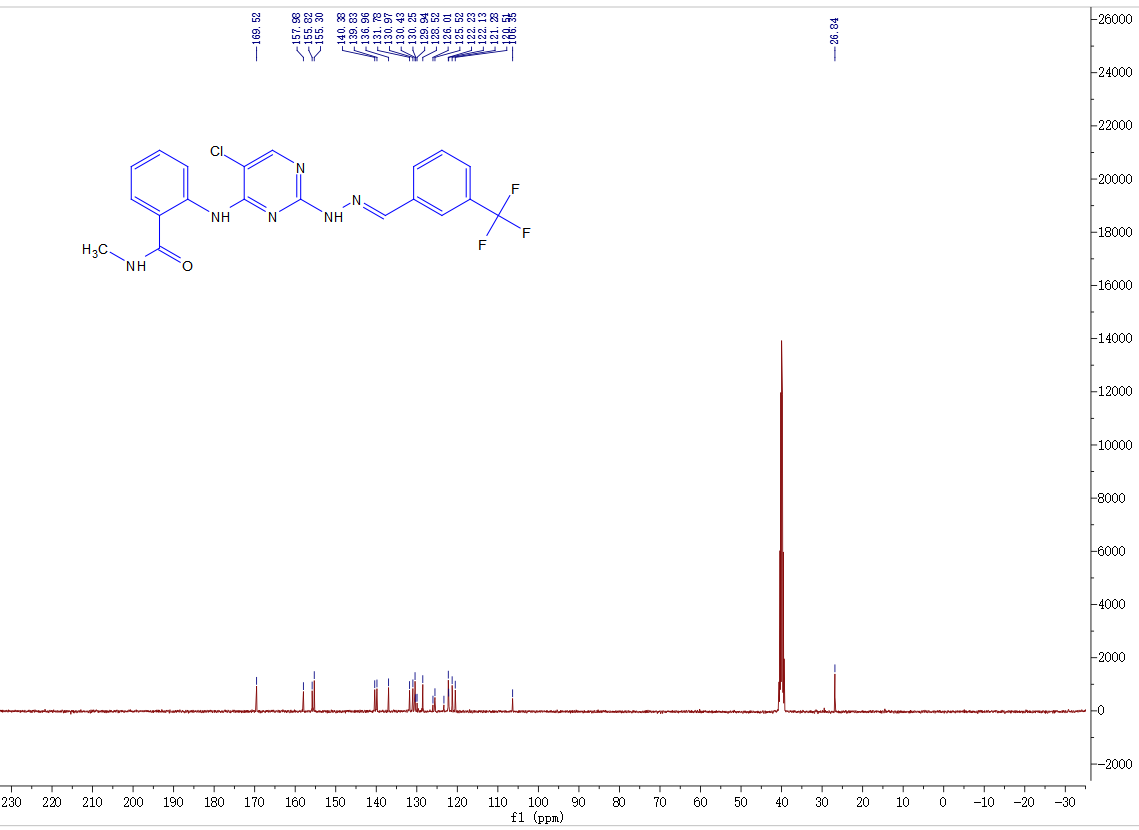


**Figure S38**. ^13^C NMR spectrum of compound 14m (100 MHz, DMSO-d6)


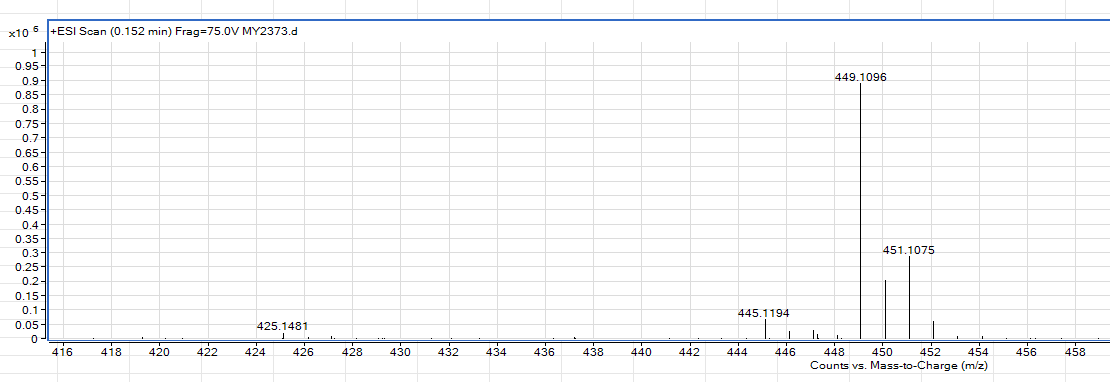


**Figure S39**. HRMS spectrum of compound 14m

- ^1^H, ^13^C-NMR and HRMS of compound 14n


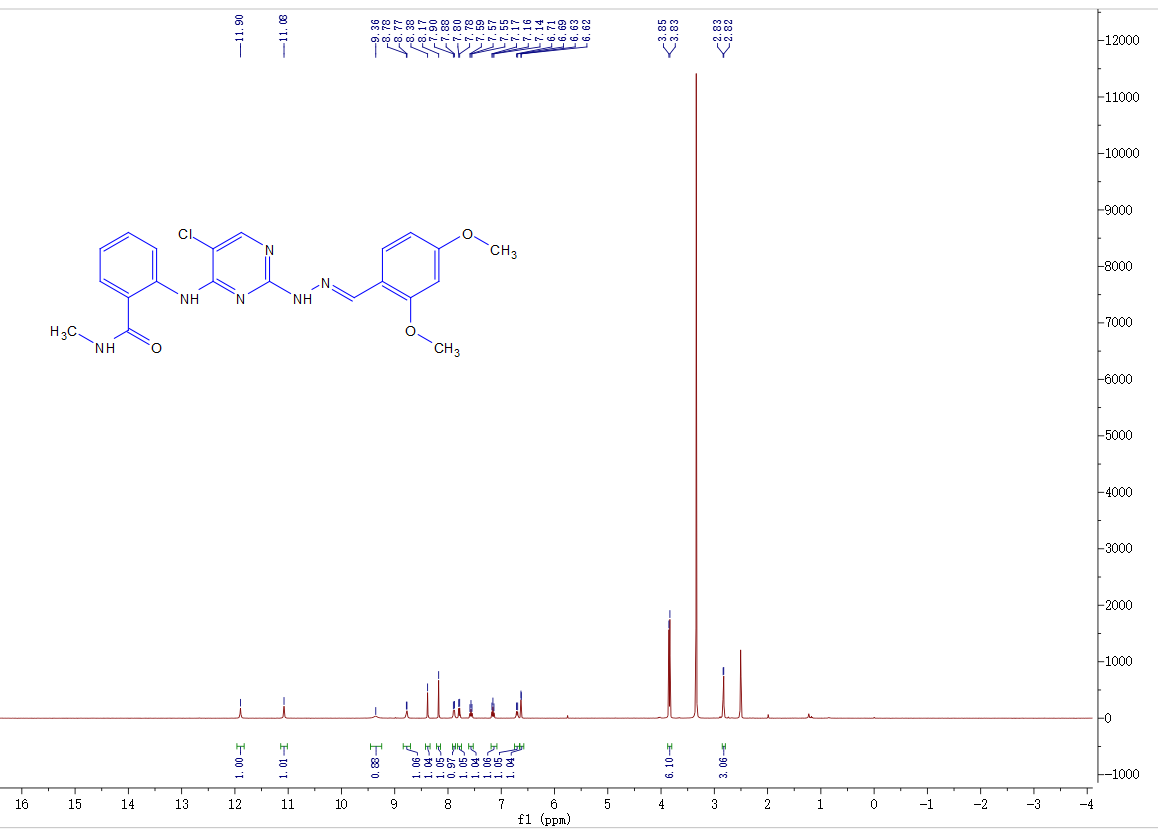


**Figure S40**. ^1^H NMR spectrum of compound 14n (400 MHz, DMSO-d6)


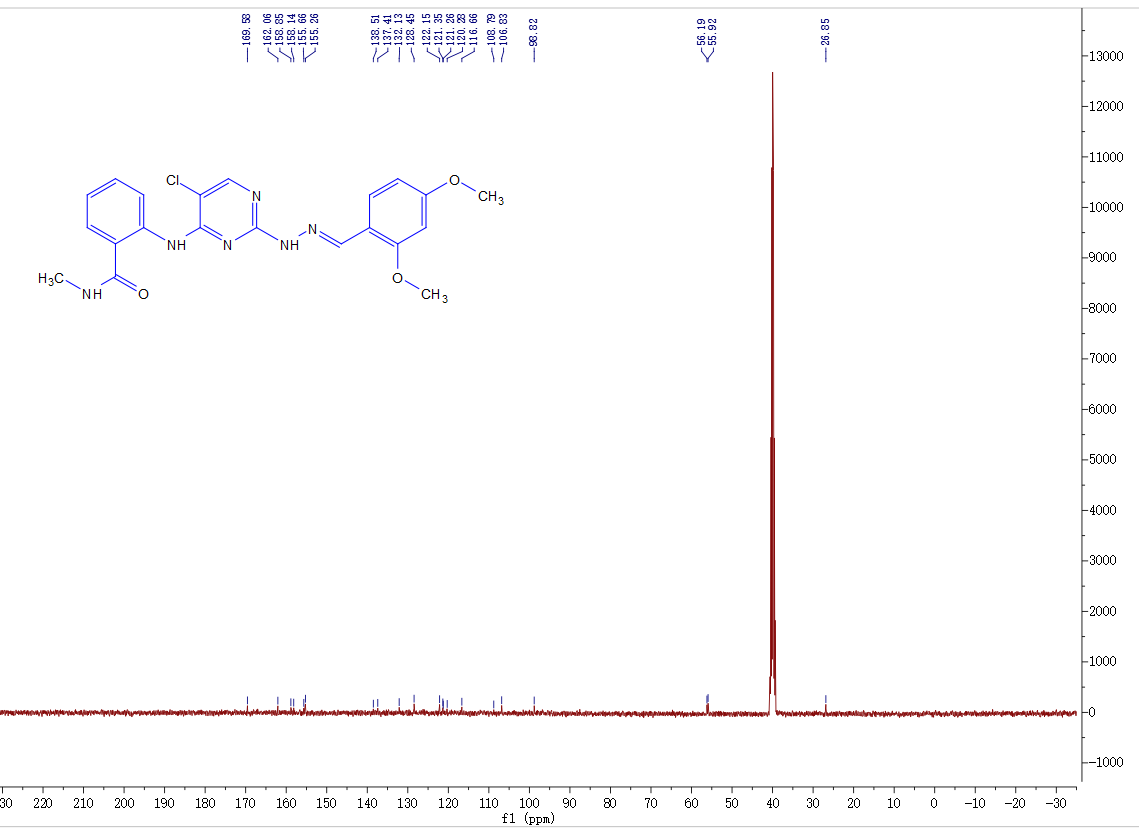


**Figure S41**. ^13^C NMR spectrum of compound 14n (100 MHz, DMSO-d6)


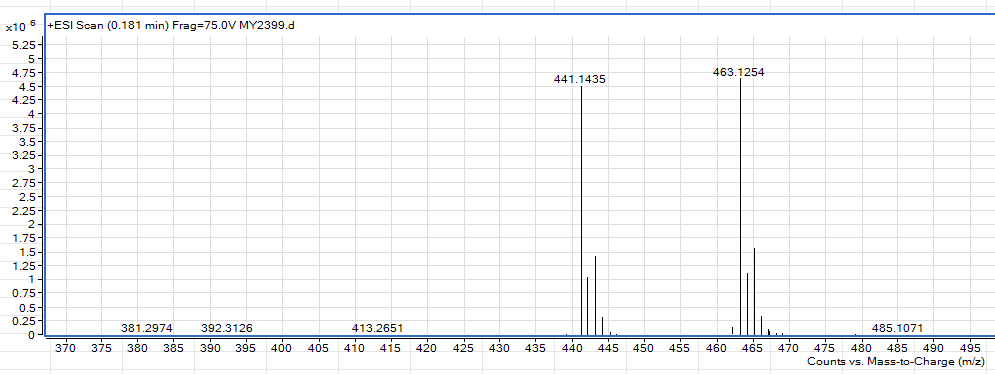


**Figure S42**. HRMS spectrum of compound 14n

- ^1^H, ^13^C-NMR and HRMS of compound 14o


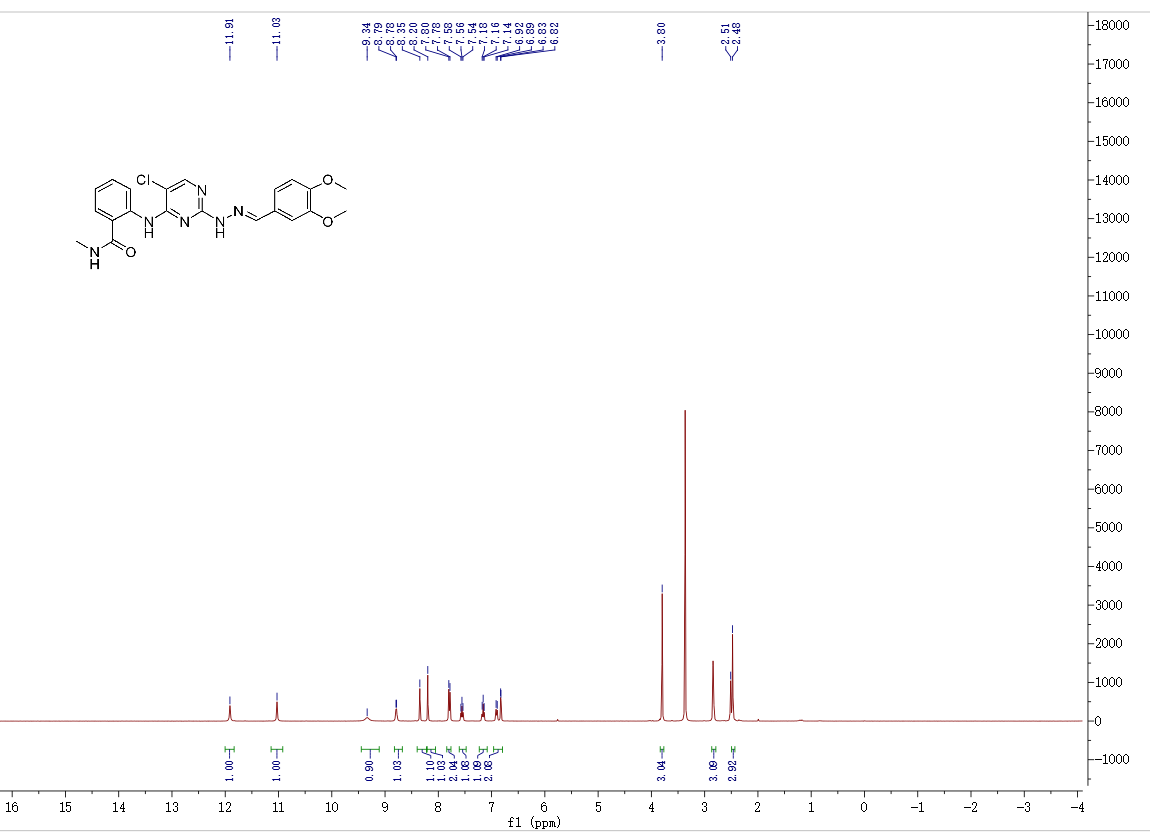


**Figure S43**. ^1^H NMR spectrum of compound 14o (400 MHz, DMSO-d6)


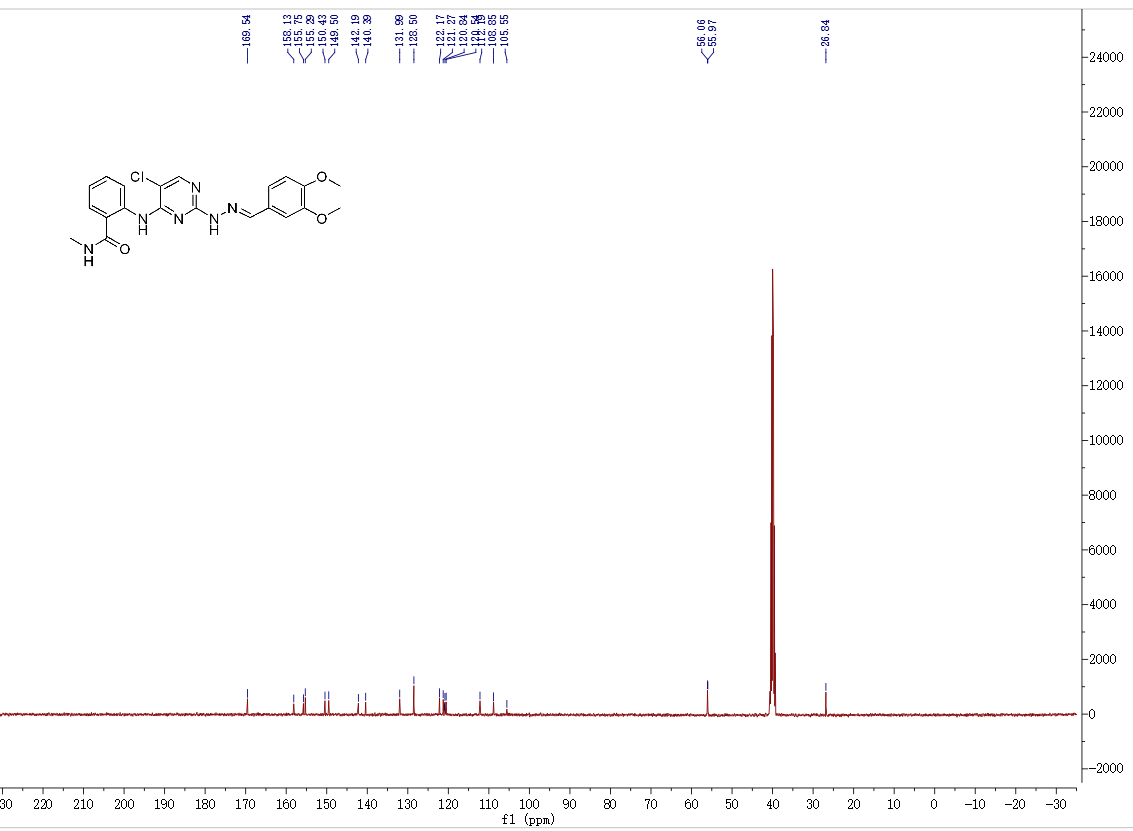


**Figure S44**. ^13^C NMR spectrum of compound 14o (100 MHz, DMSO-d6)


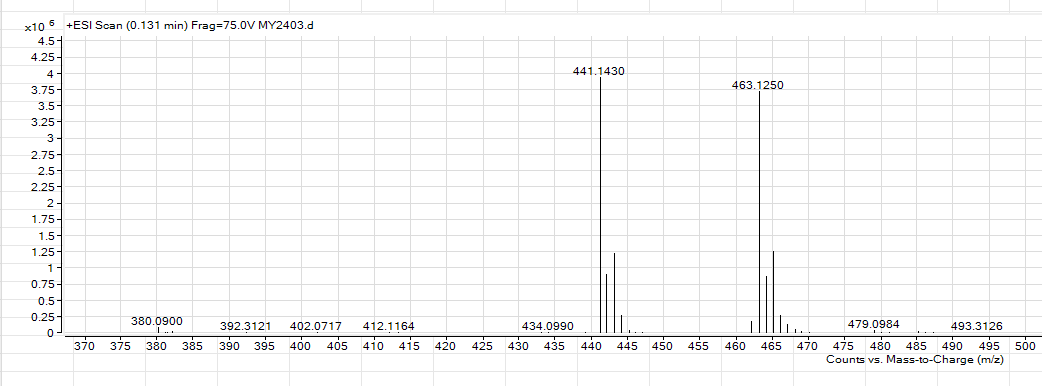


**Figure S45**. HRMS spectrum of compound 14o

- ^1^H, ^13^C-NMR and HRMS of compound 14p


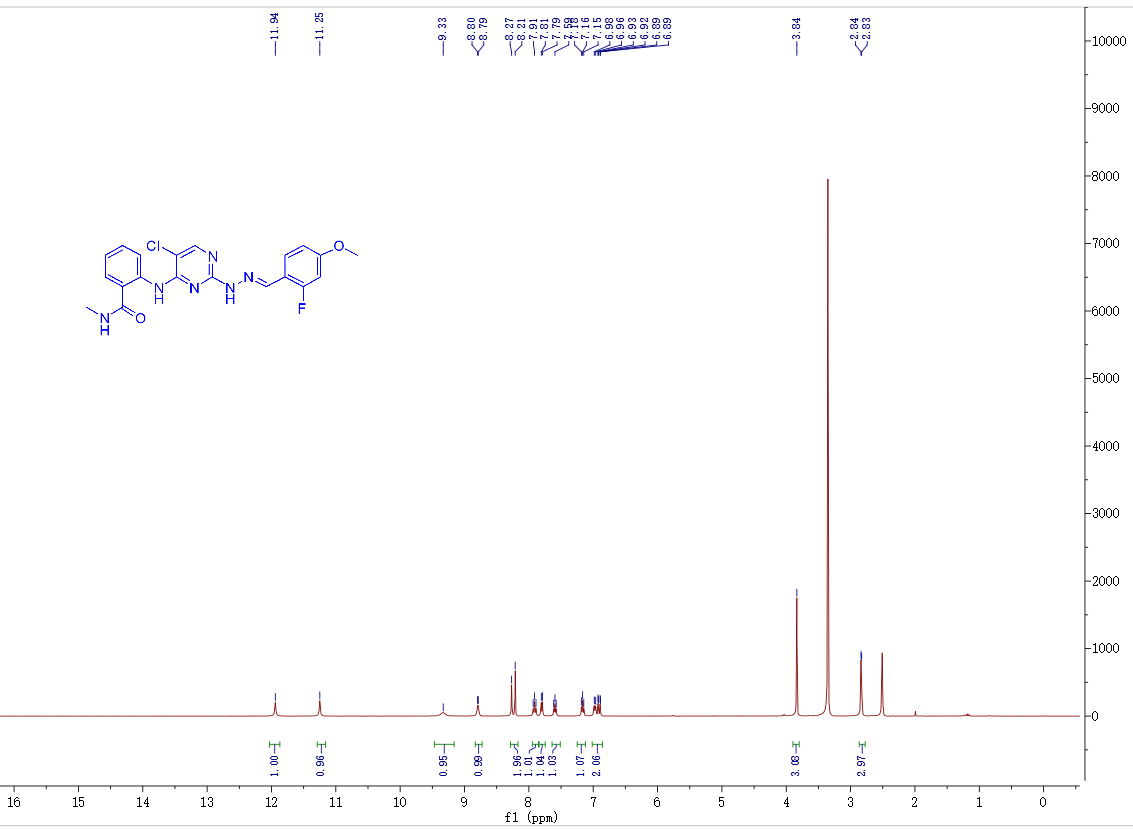


**Figure S46**. ^1^H NMR spectrum of compound 14p (400 MHz, DMSO-d6)


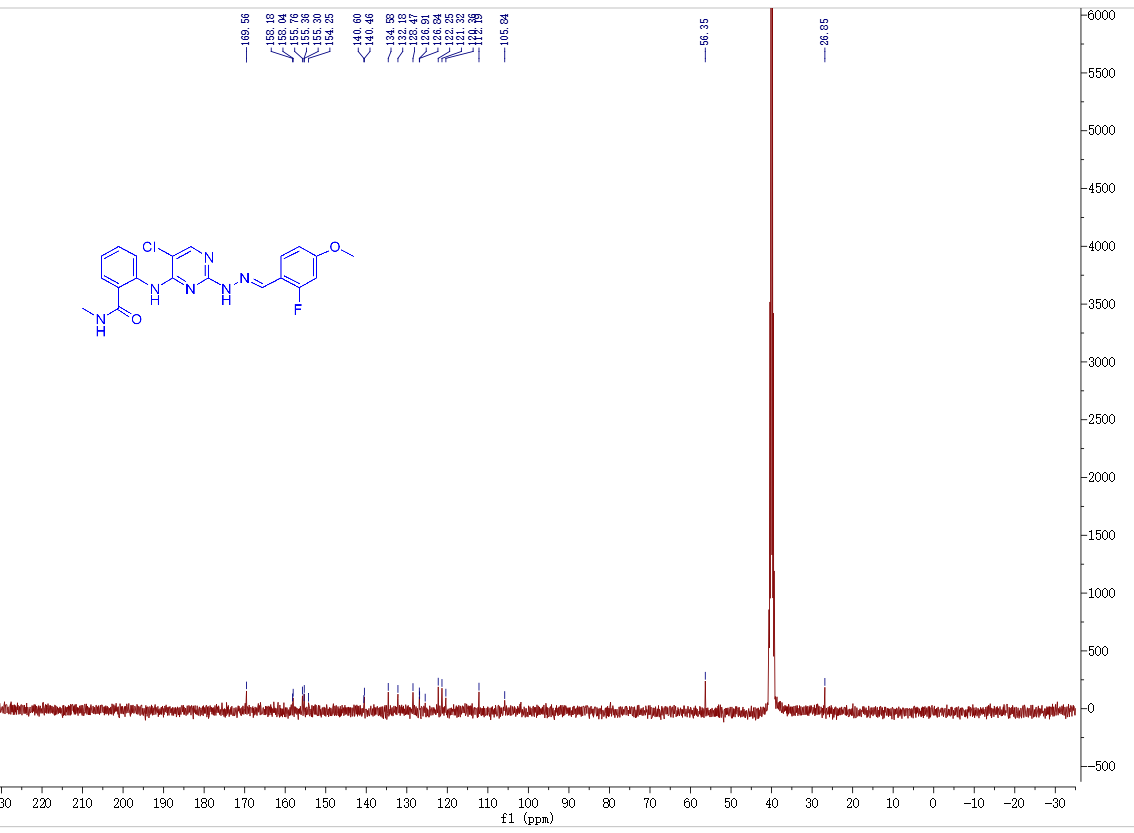


**Figure S47**. ^13^C NMR spectrum of compound 14p (100 MHz, DMSO-d6)


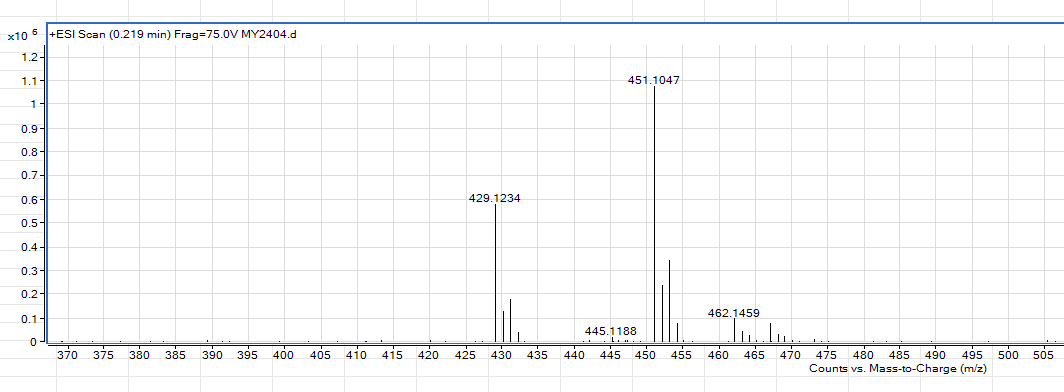


**Figure S48**. HRMS spectrum of compound 14p

- ^1^H, ^13^C-NMR and HRMS of compound 14q


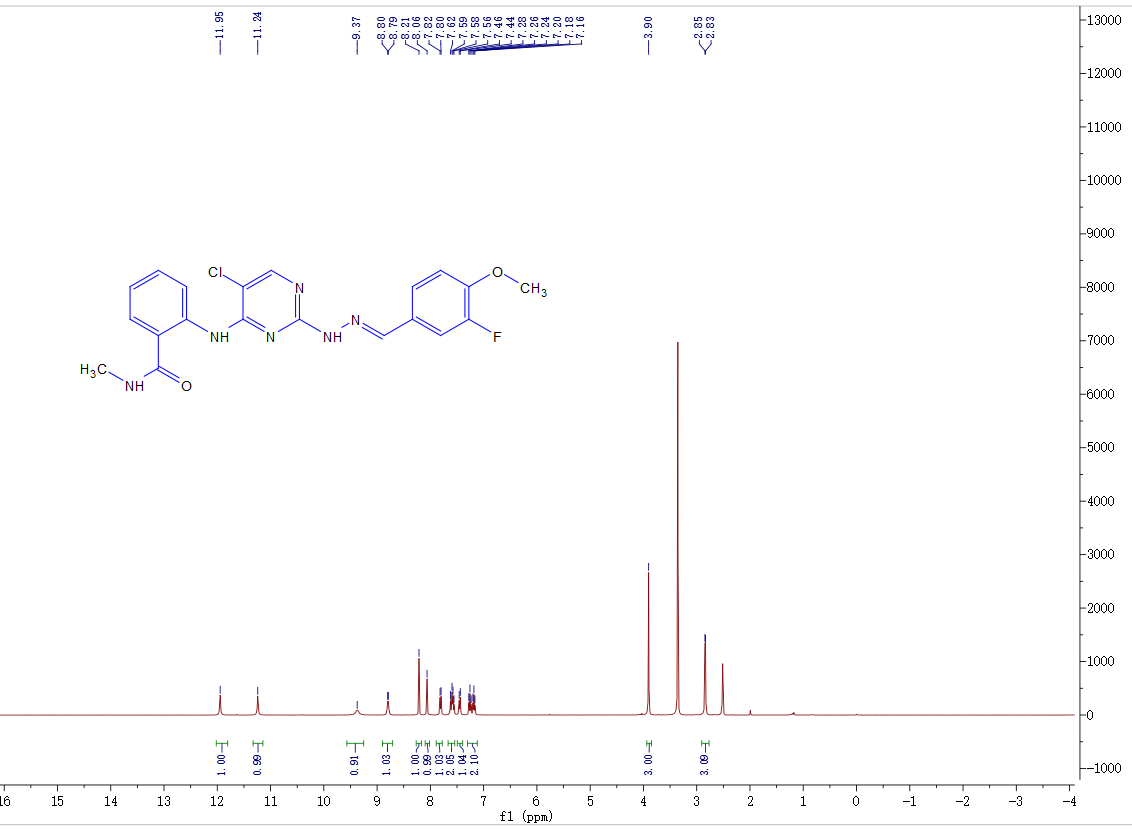


**Figure S49**. ^1^H NMR spectrum of compound 14q (400 MHz, DMSO-d6)


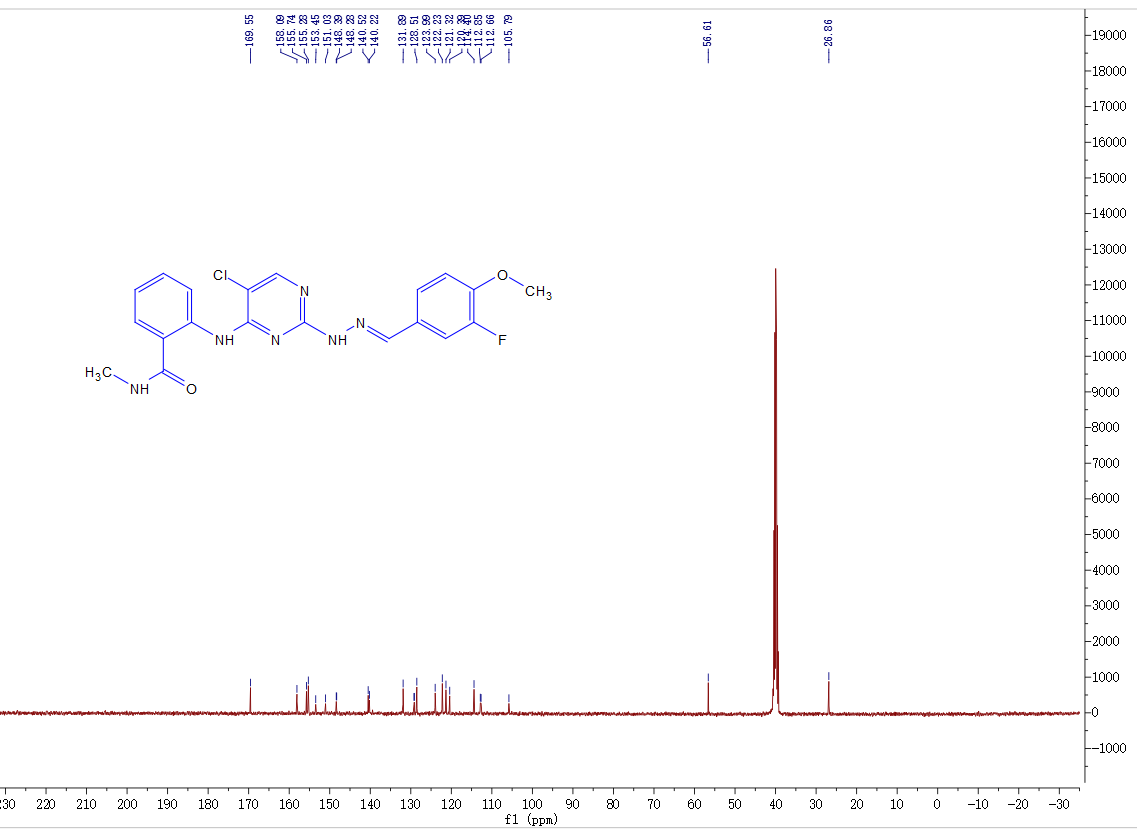


**Figure S50**. ^13^C NMR spectrum of compound 14q (100 MHz, DMSO-d6)


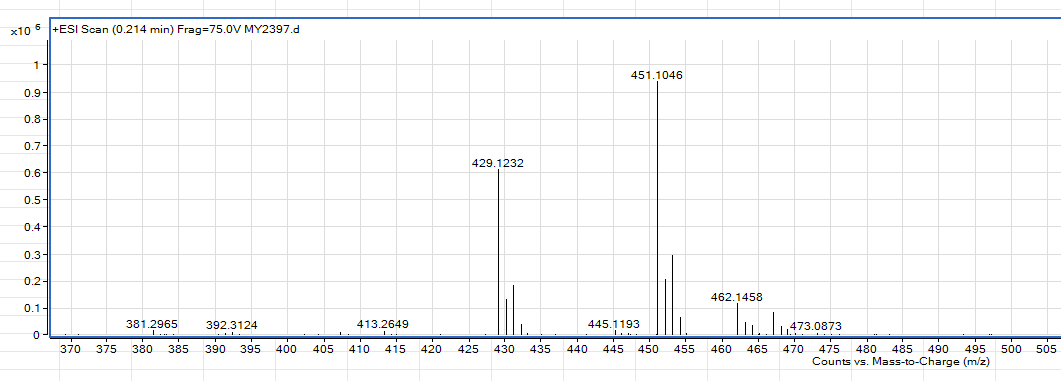


**Figure S51**. HRMS spectrum of compound 14q

- ^1^H, ^13^C-NMR and HRMS of compound 14r


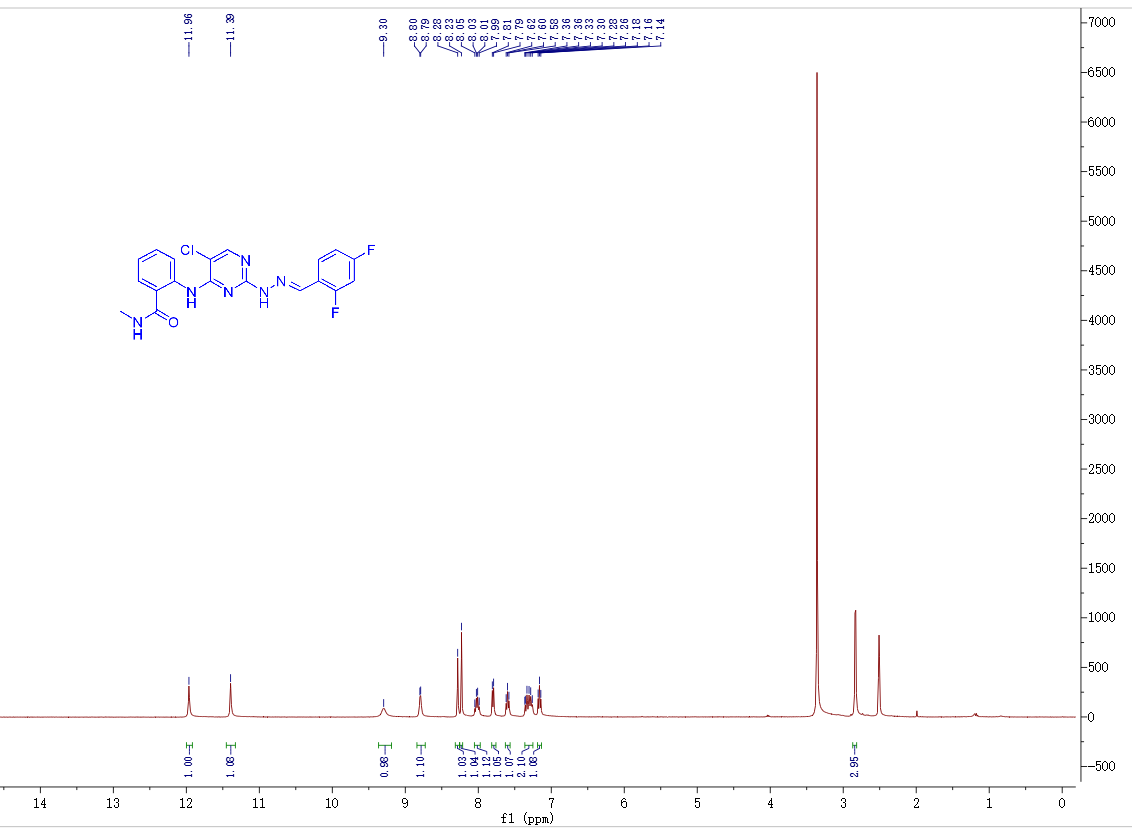


**Figure S52**. ^1^H NMR spectrum of compound 14r (400 MHz, DMSO-d6)


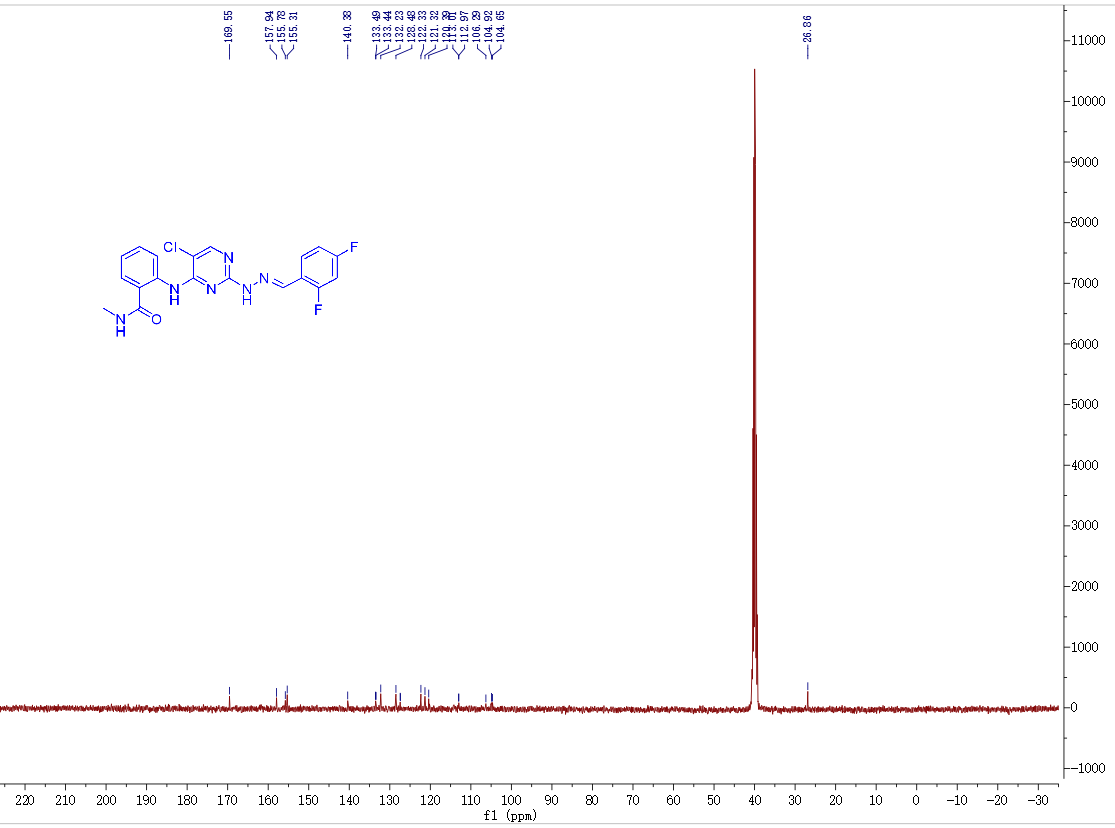


**Figure S53**. ^13^C NMR spectrum of compound 14r (100 MHz, DMSO-d6)


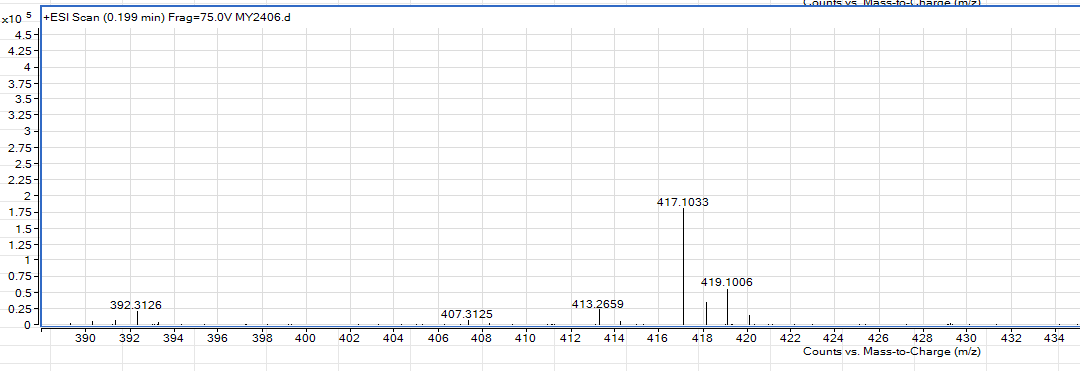


**Figure S54**. HRMS spectrum of compound 14r

- ^1^H, ^13^C-NMR and HRMS of compound 14s


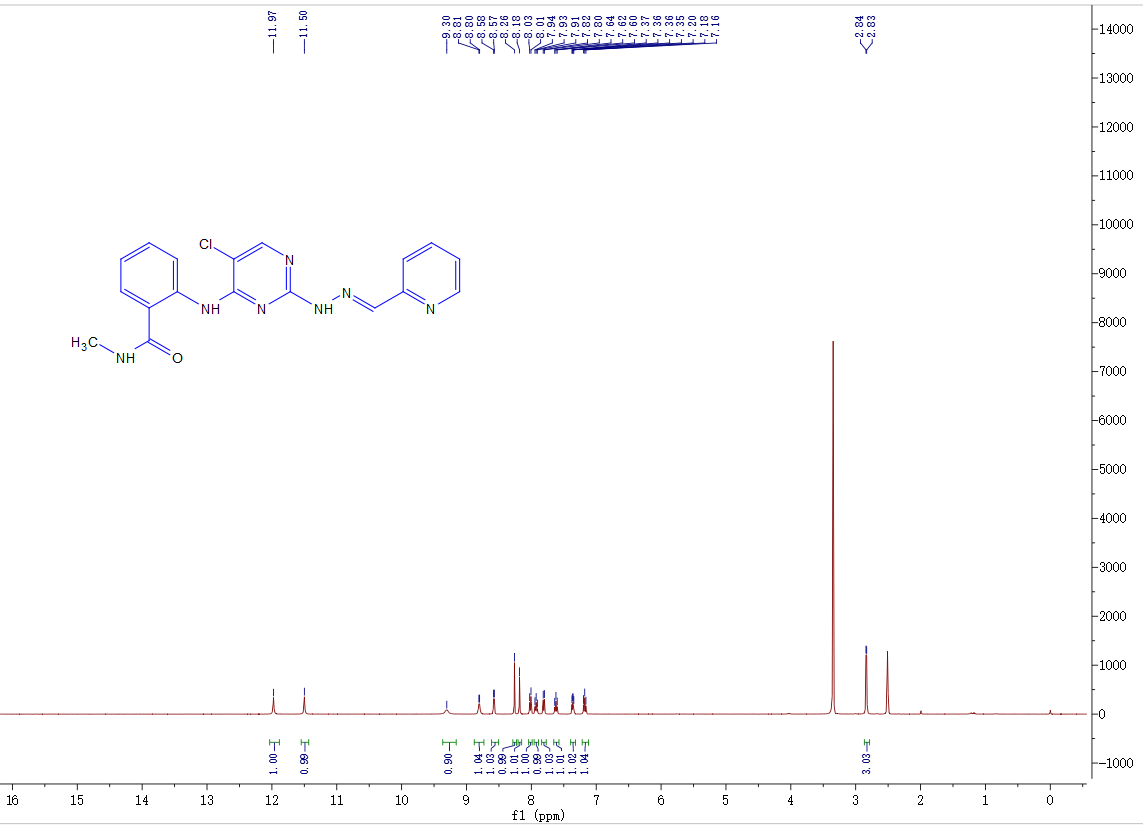


**Figure S55**. ^1^H NMR spectrum of compound 14s (400 MHz, DMSO-d6)


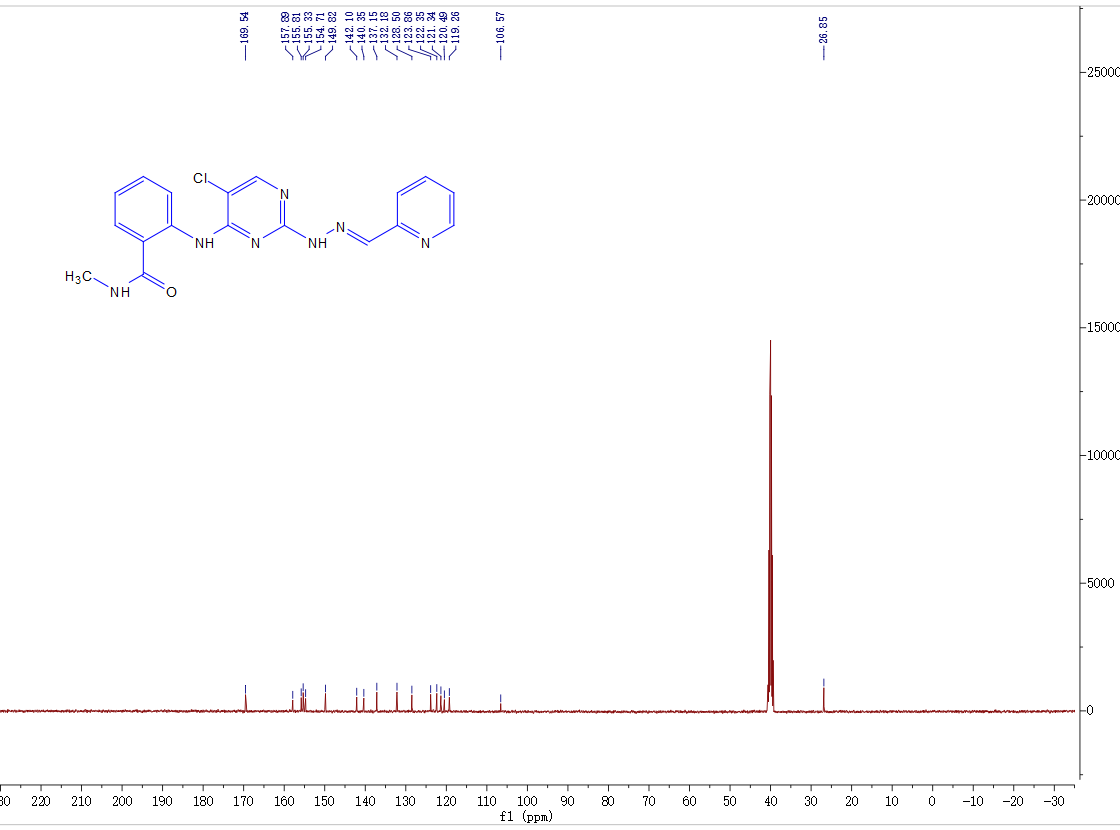


**Figure S56**. ^13^C NMR spectrum of compound 14s (100 MHz, DMSO-d6)


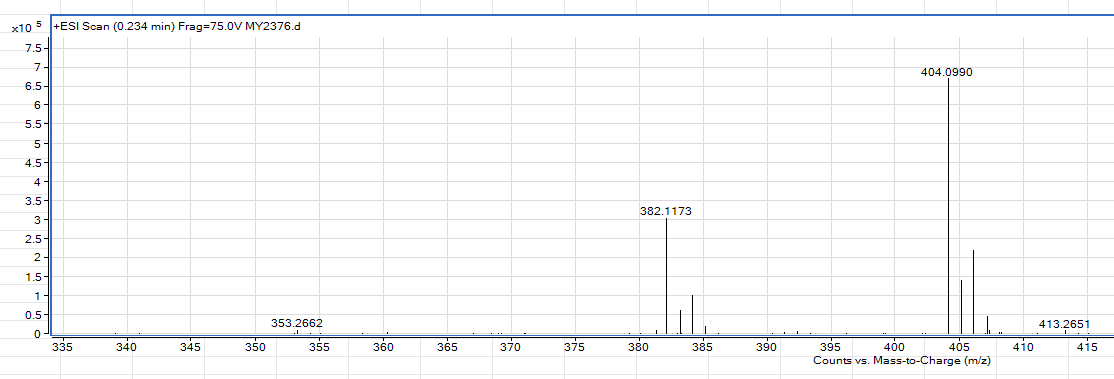


**Figure S57**. HRMS spectrum of compound 14s

- ^1^H, ^13^C-NMR and HRMS of compound 14t


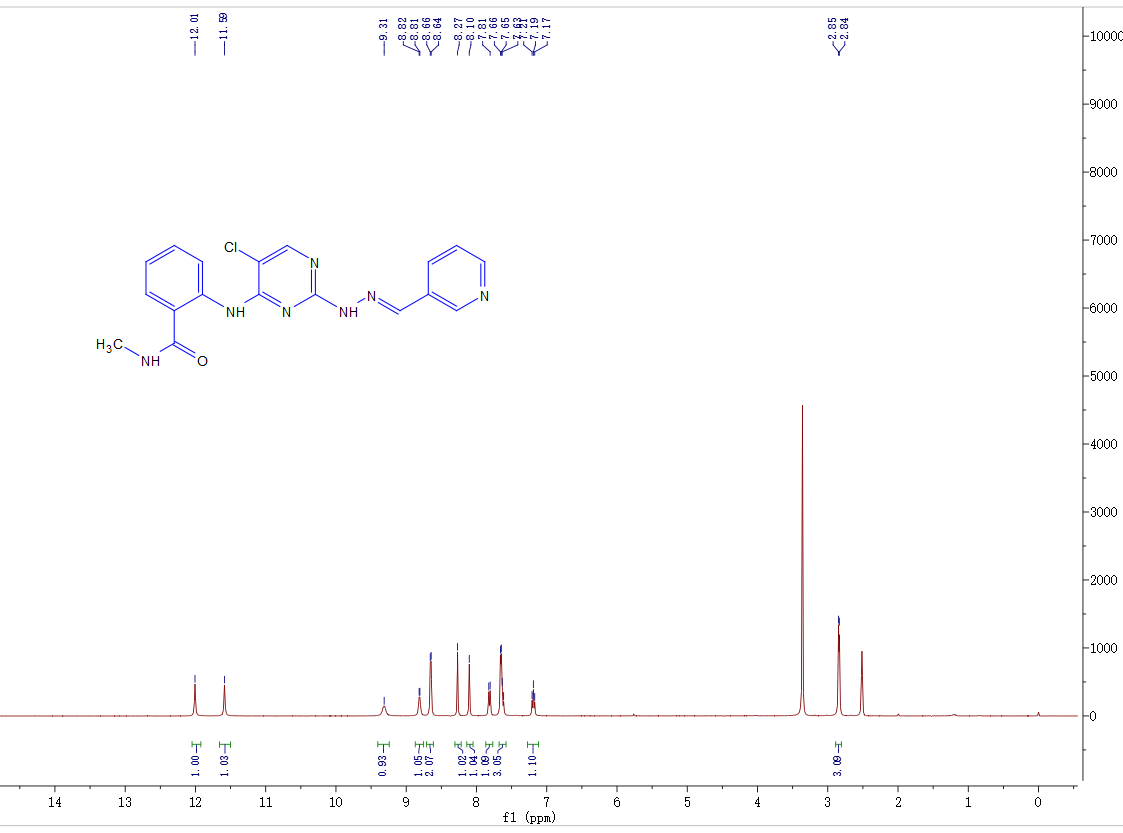


**Figure S58**. ^1^H NMR spectrum of compound 14t (400 MHz, DMSO-d6)


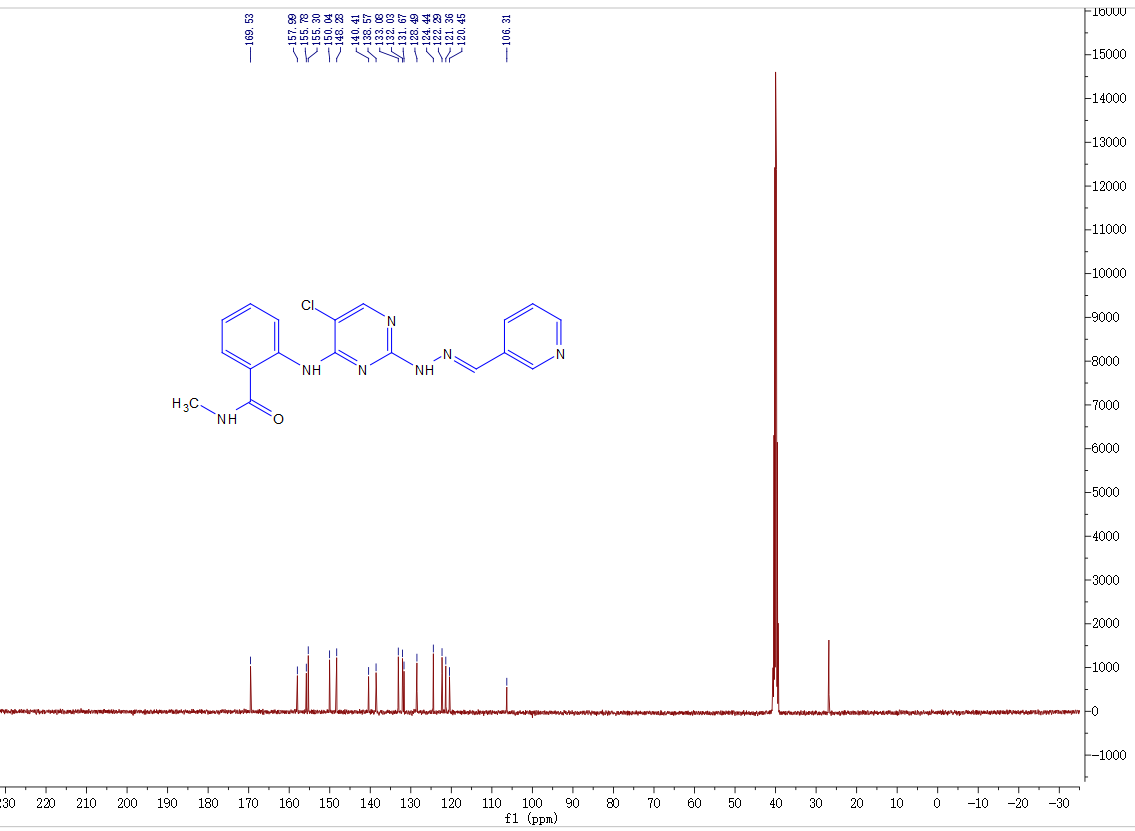


**Figure S59**. ^13^C NMR spectrum of compound 14t (100 MHz, DMSO-d6)


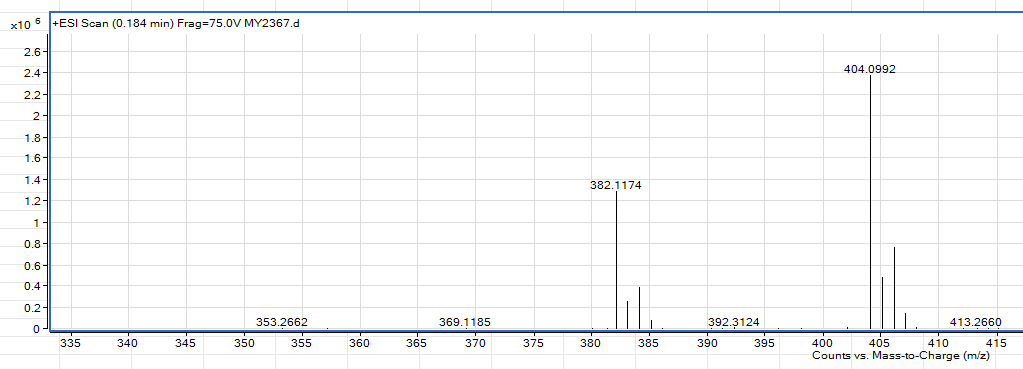


**Figure S60**. HRMS spectrum of compound 14t

- ^1^H and ^13^C-NMR of compound 14u


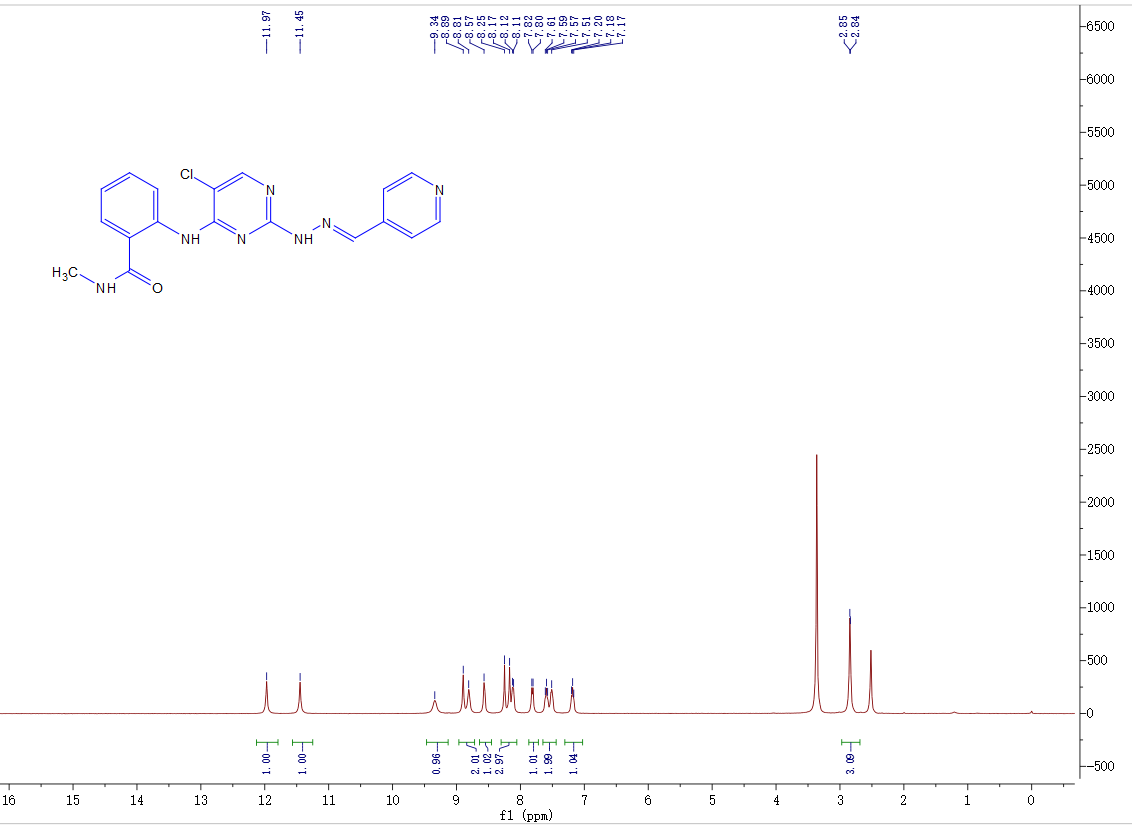


**Figure S61**. ^1^H NMR spectrum of compound 14u (400 MHz, DMSO-d6)
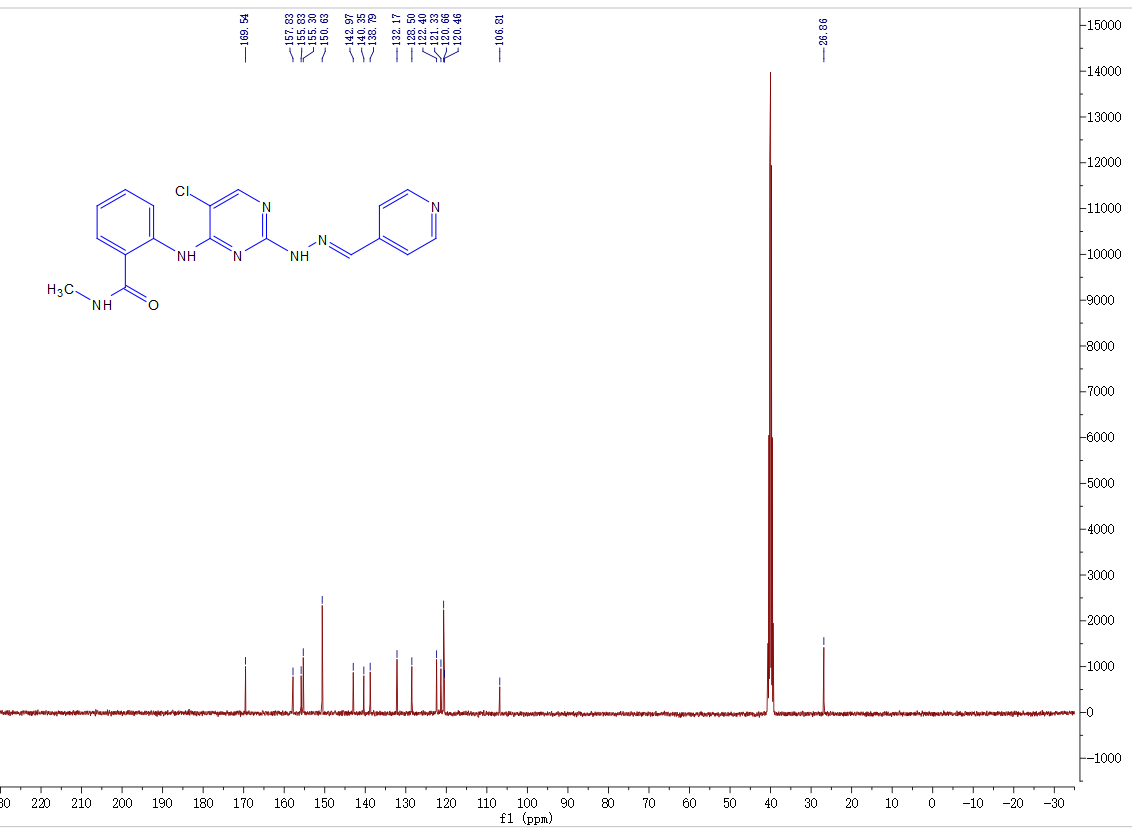


**Figure S62**. ^13^C NMR spectrum of compound 14u (100 MHz, DMSO-d6)

- ^1^H, ^13^C-NMR and HRMS of compound 14v


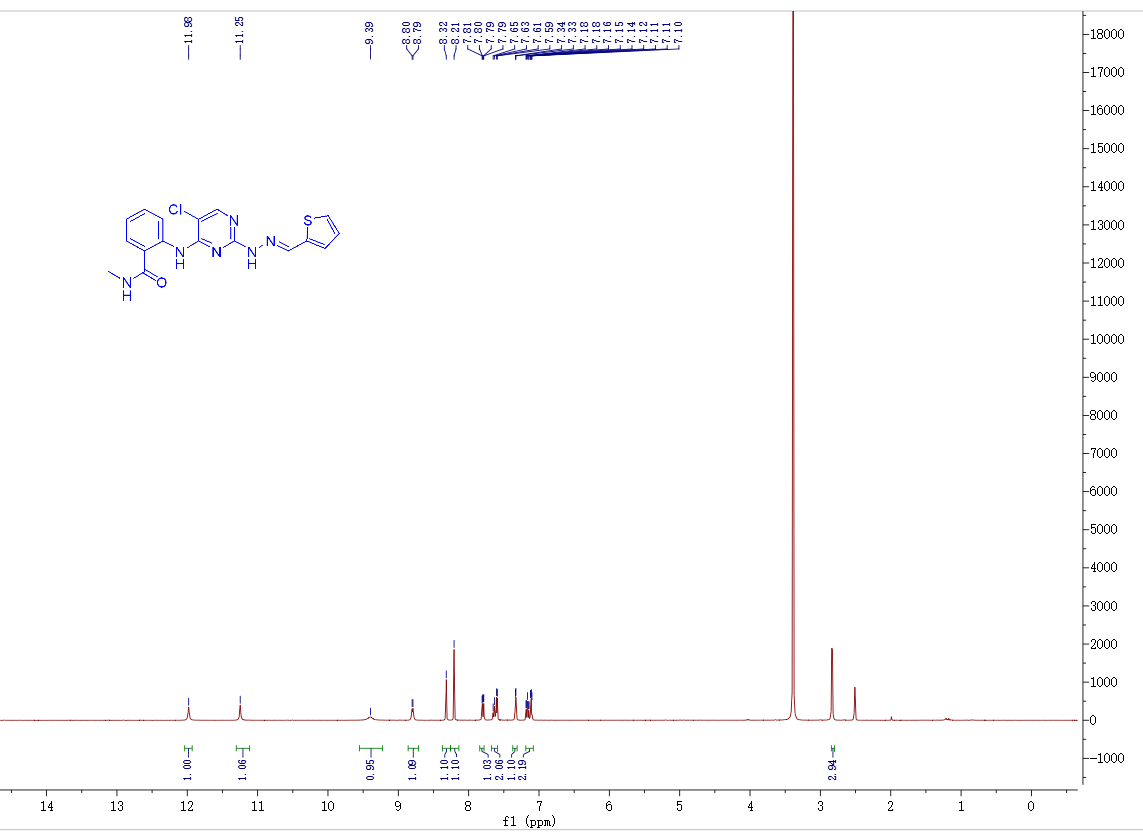


**Figure S63**. ^1^H NMR spectrum of compound 14v (400 MHz, DMSO-d6)


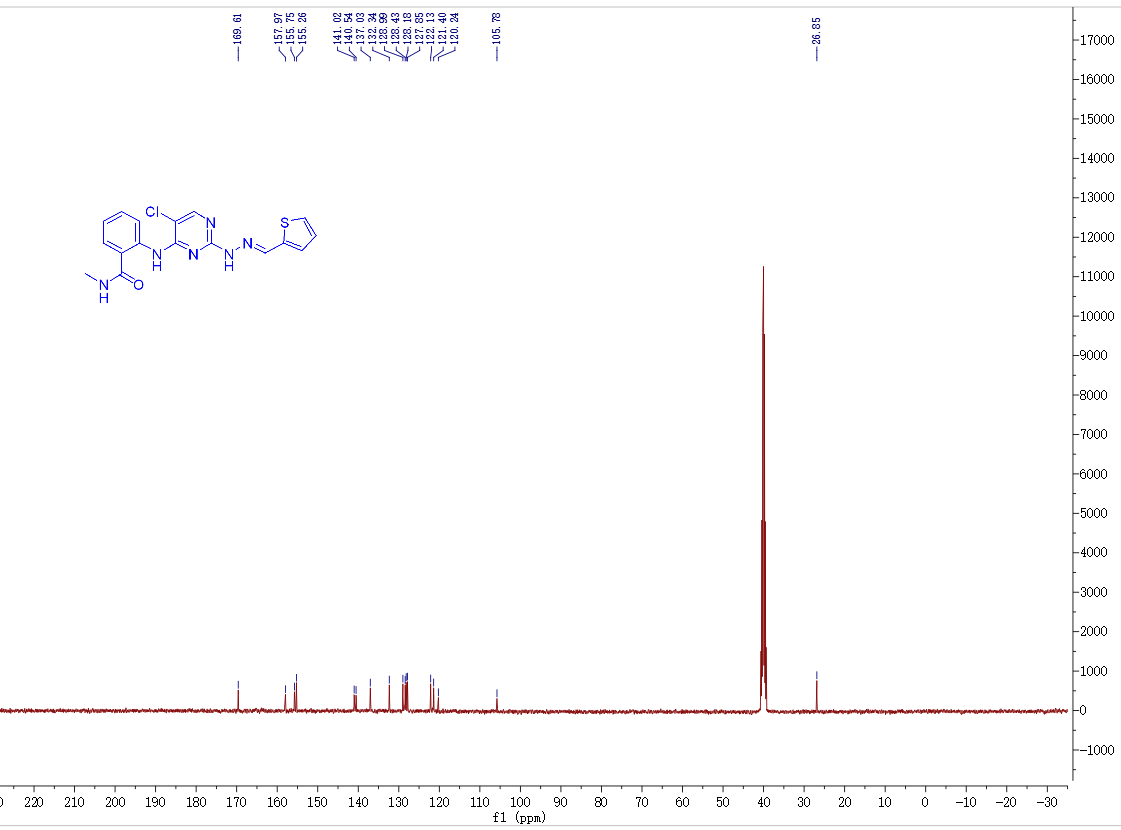


**Figure S64**. ^13^C NMR spectrum of compound 14v (100 MHz, DMSO-d6)


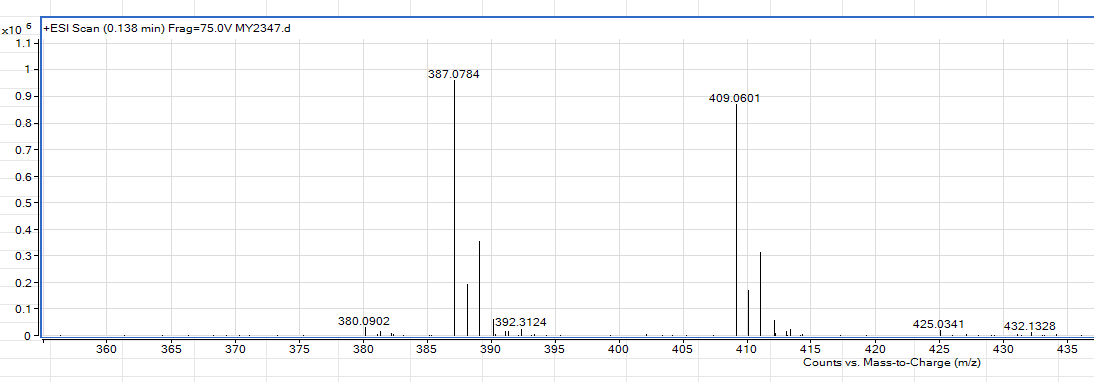


**Figure S65**. HRMS spectrum of compound 14v

- ^1^H, ^13^C-NMR and HRMS of compound 14w


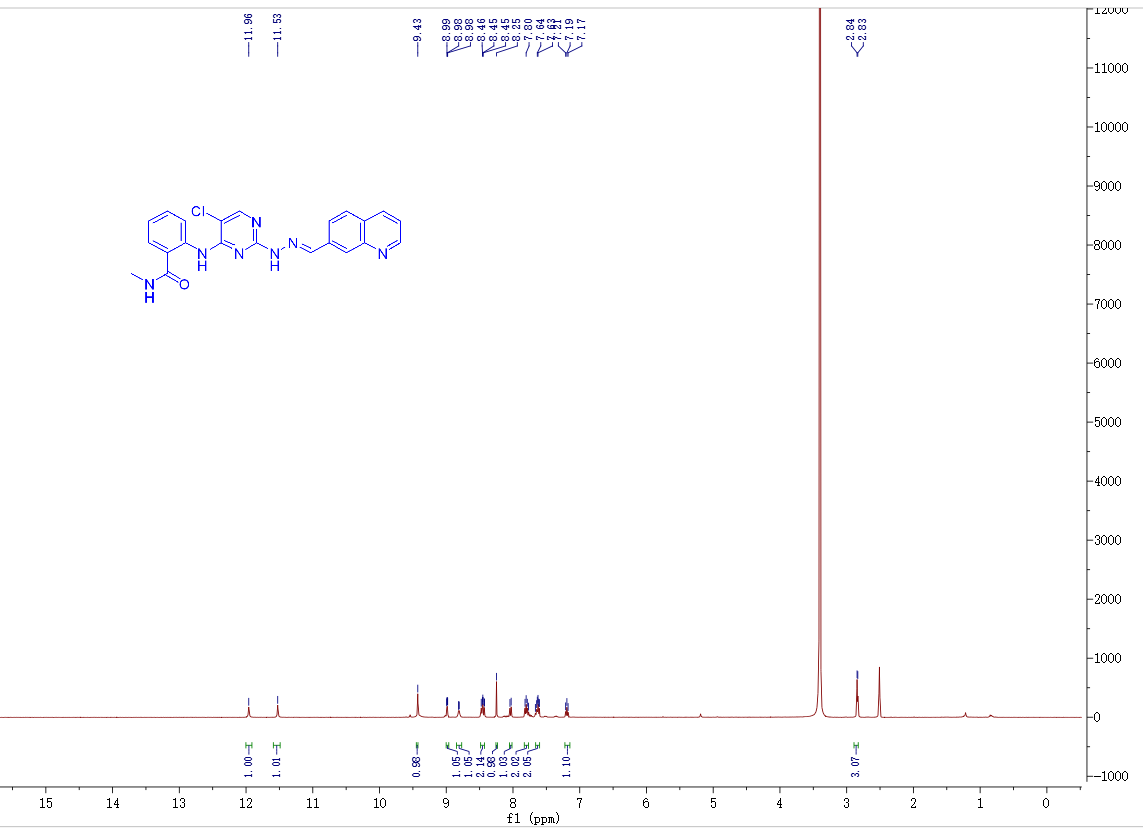


**Figure S66**. ^1^H NMR spectrum of compound 14w (400 MHz, DMSO-d6)


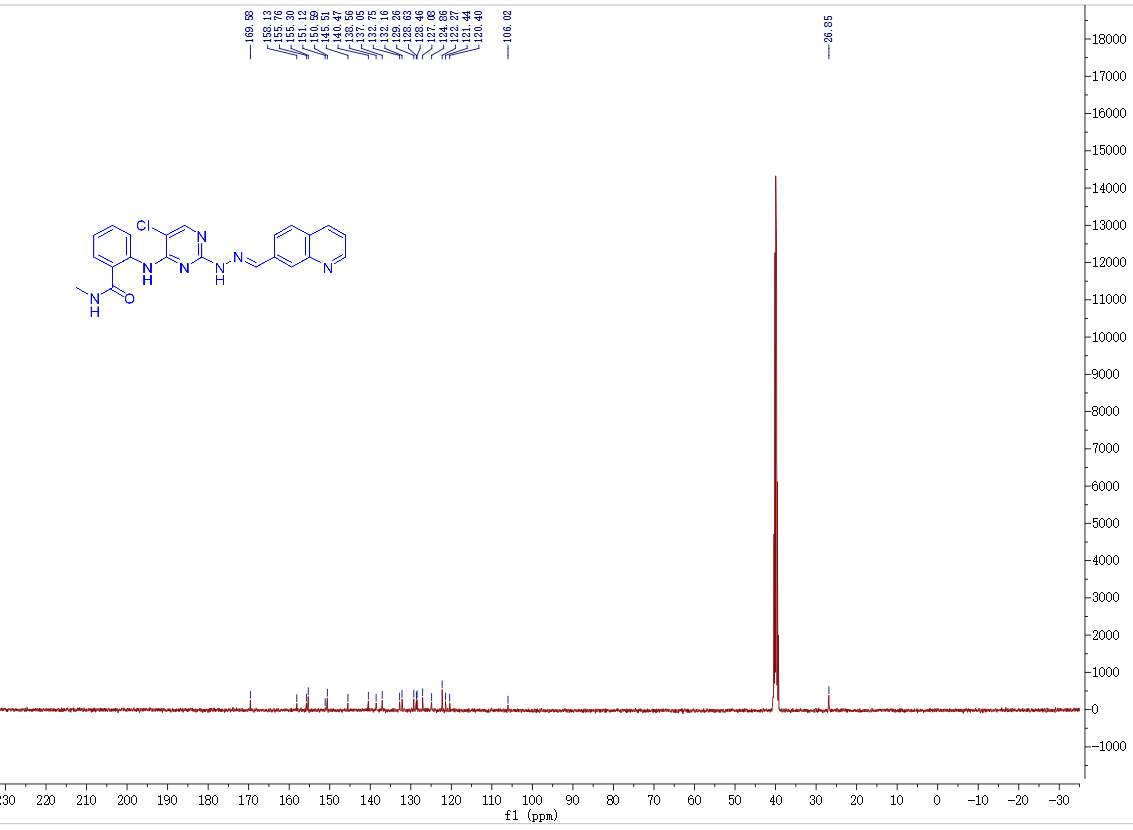


**Figure S67**. ^13^C NMR spectrum of compound 14w (100 MHz, DMSO-d6)


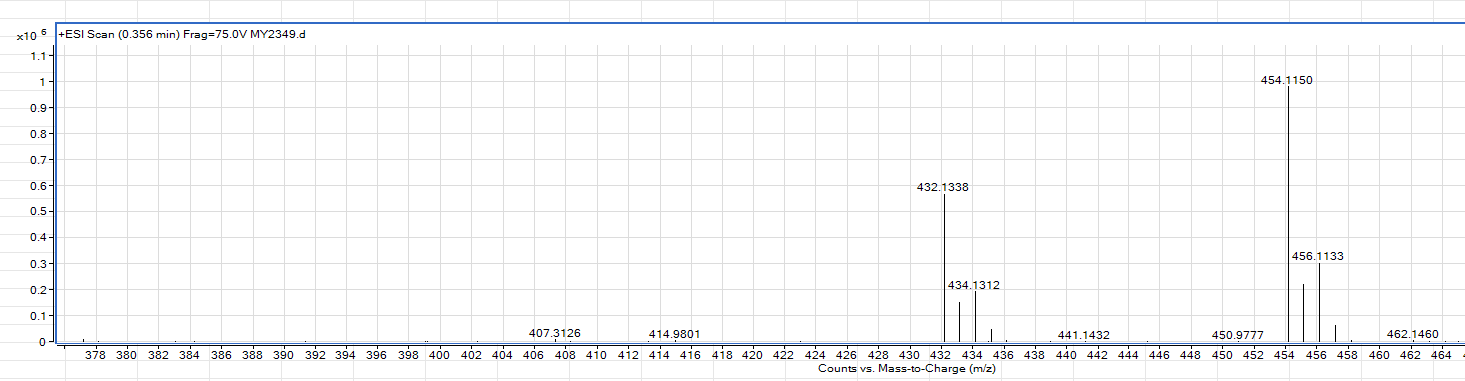


**Figure S68**. HRMS spectrum of compound 14w

- ^1^H, ^13^C-NMR and HRMS of compound 14x


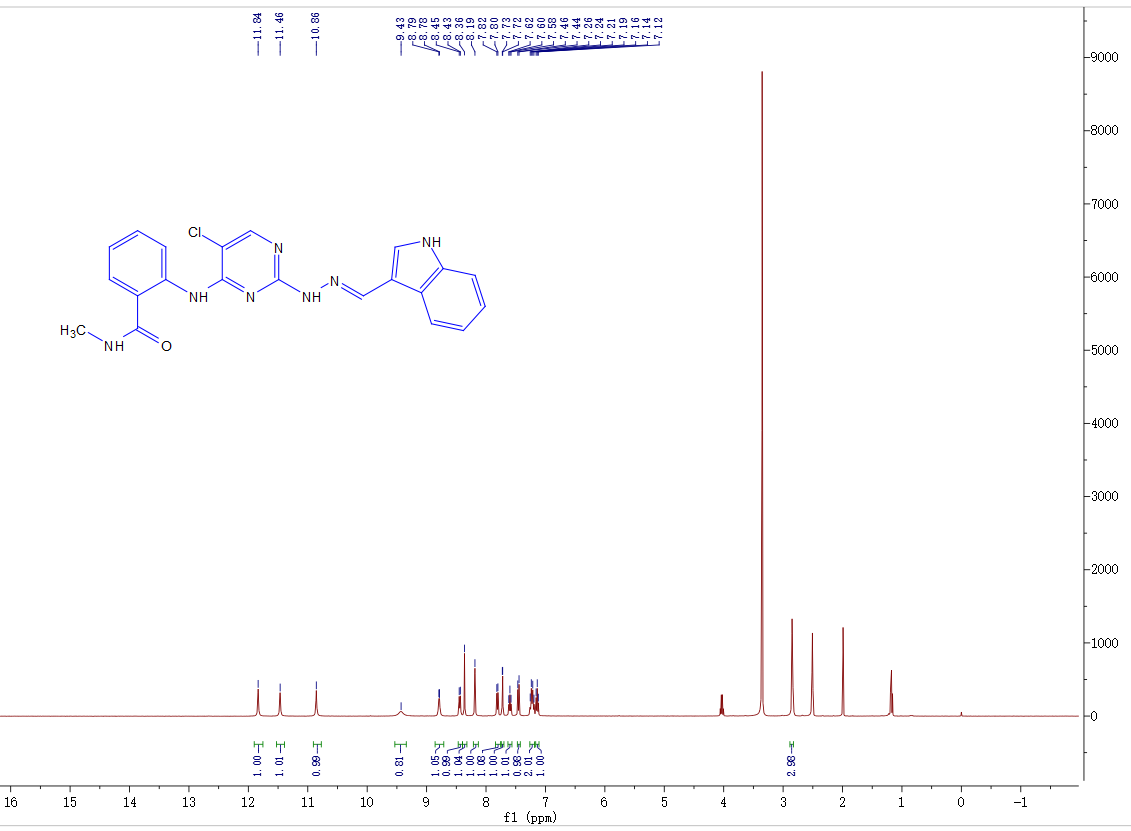


**Figure S69**. ^1^H NMR spectrum of compound 14x (400 MHz, DMSO-d6)


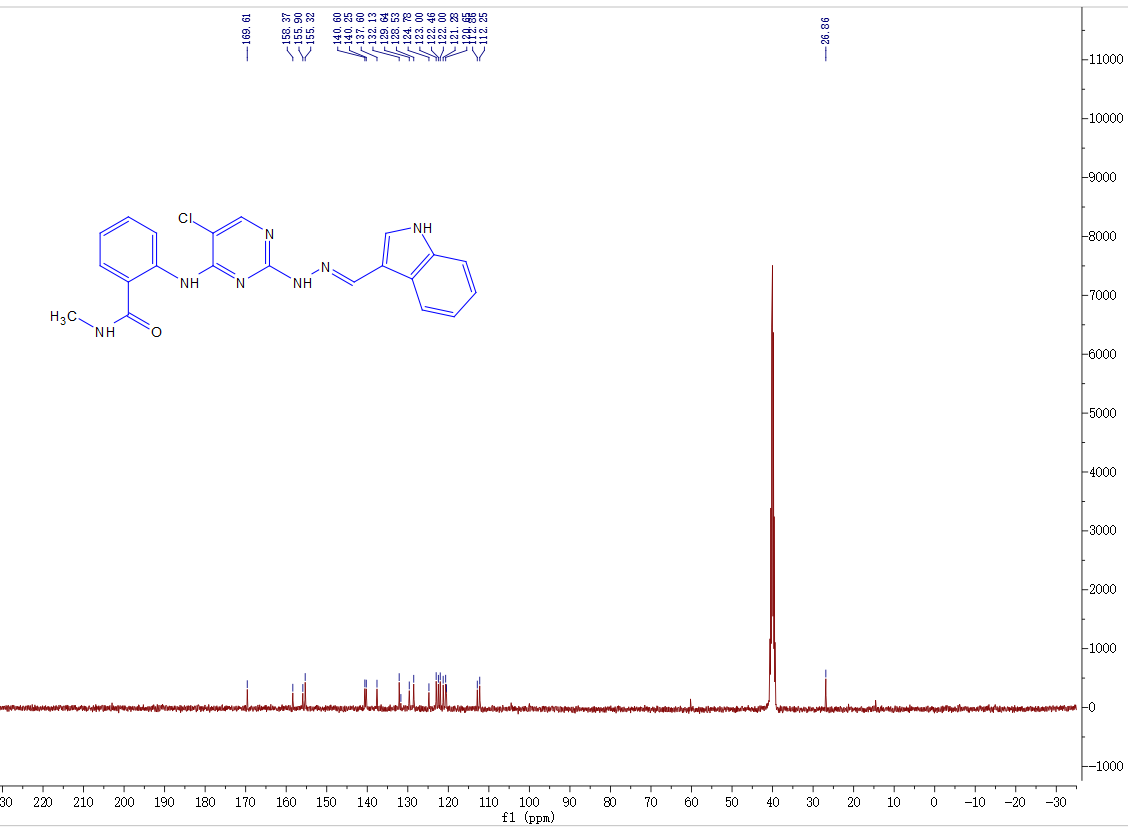


**Figure S70**. ^13^C NMR spectrum of compound 14x (100 MHz, DMSO-d6)


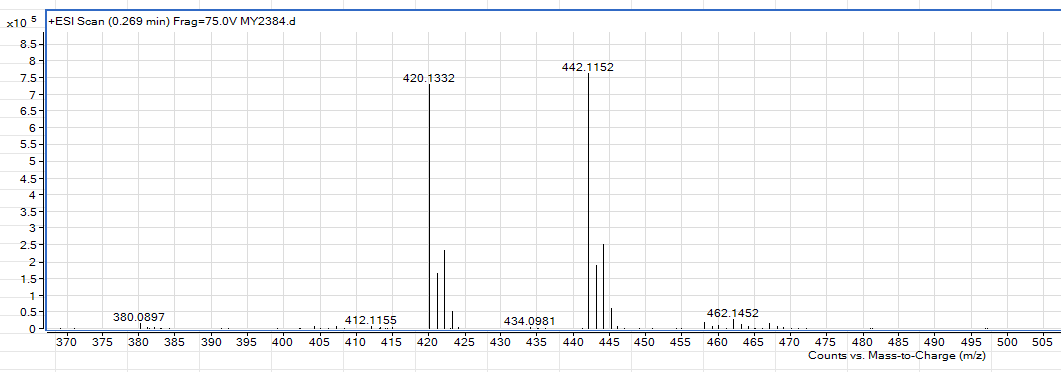


**Figure S71**. HRMS spectrum of compound 14x

- ^1^H, ^13^C-NMR and HRMS of compound 14y


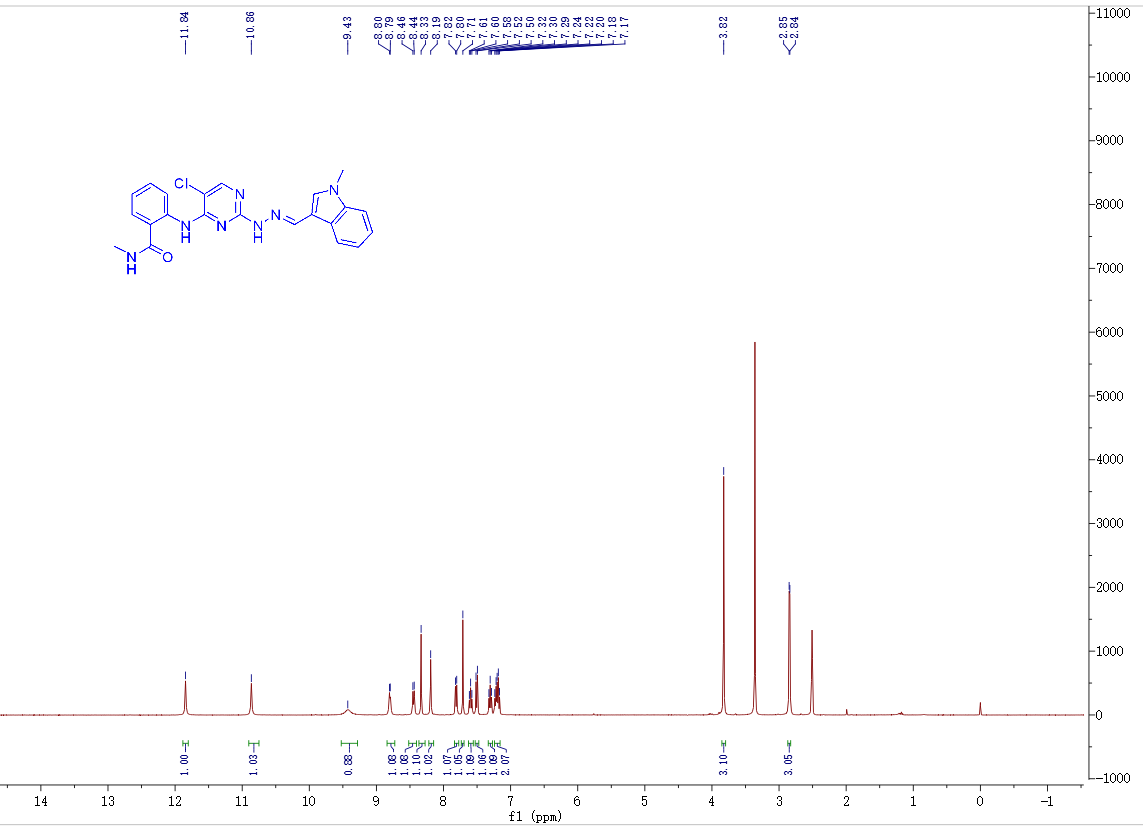


**Figure S72**. ^1^H NMR spectrum of compound 14y (400 MHz, DMSO-d6)


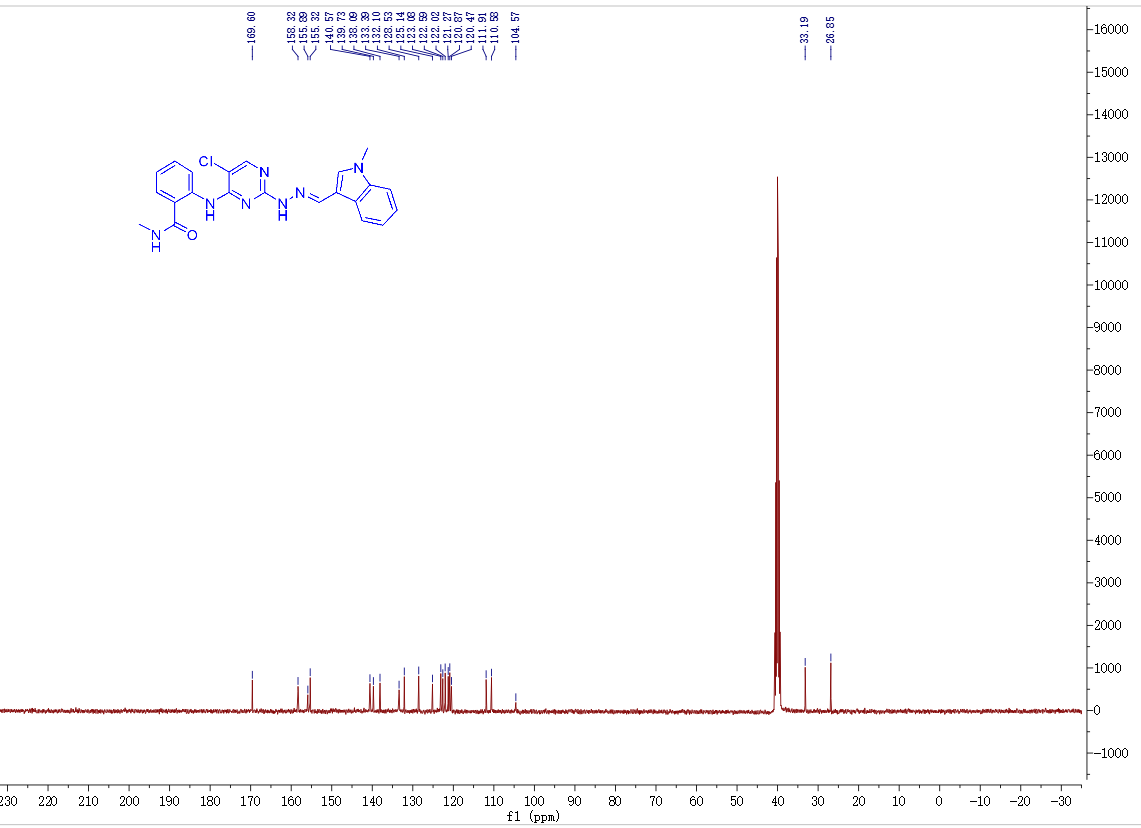


**Figure S73**. ^13^C NMR spectrum of compound 14y (100 MHz, DMSO-d6)


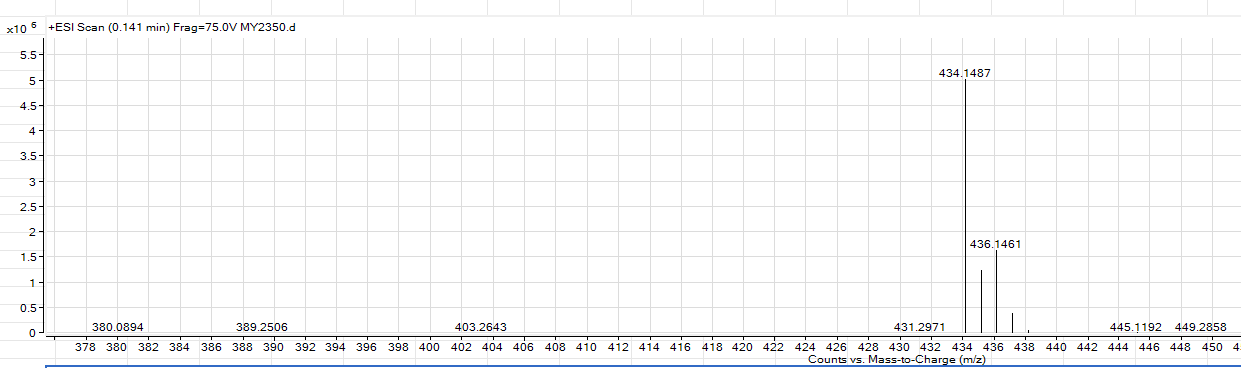


**Figure S74**. HRMS spectrum of compound 14y

- ^1^H, ^13^C-NMR and HRMS of compound 14z


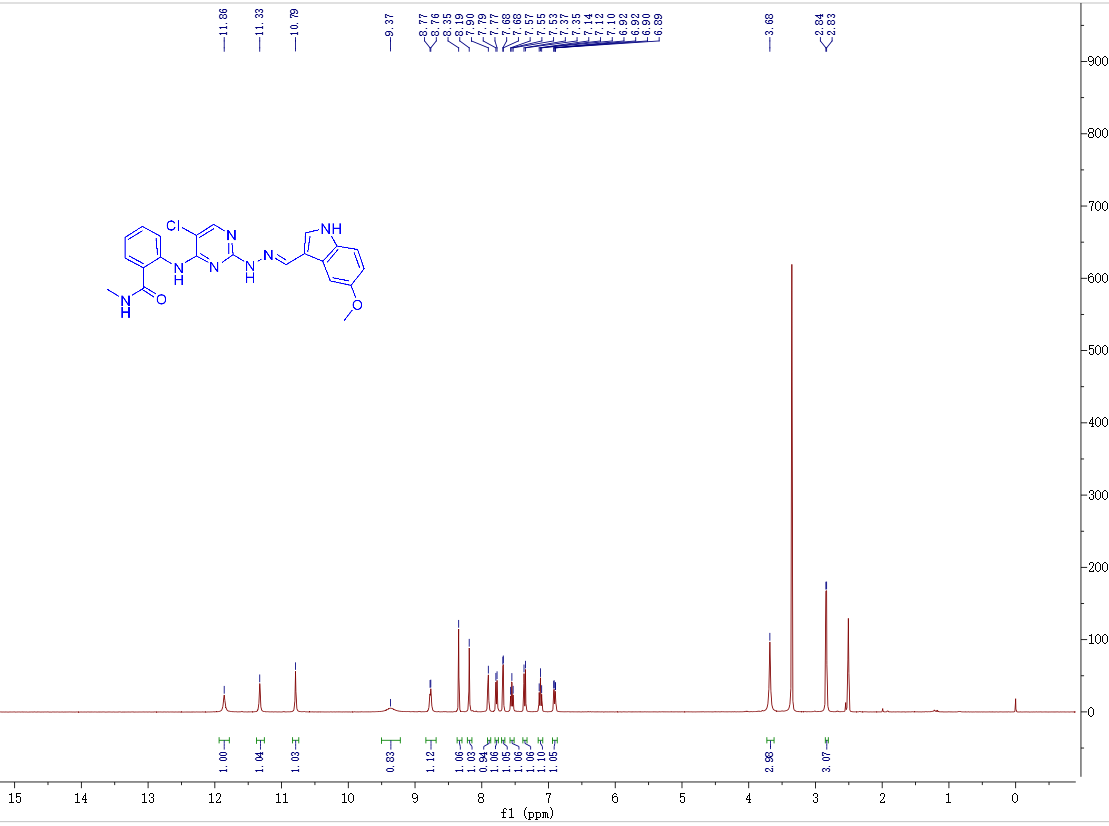


**Figure S75**. ^1^H NMR spectrum of compound 14z (400 MHz, DMSO-d6)


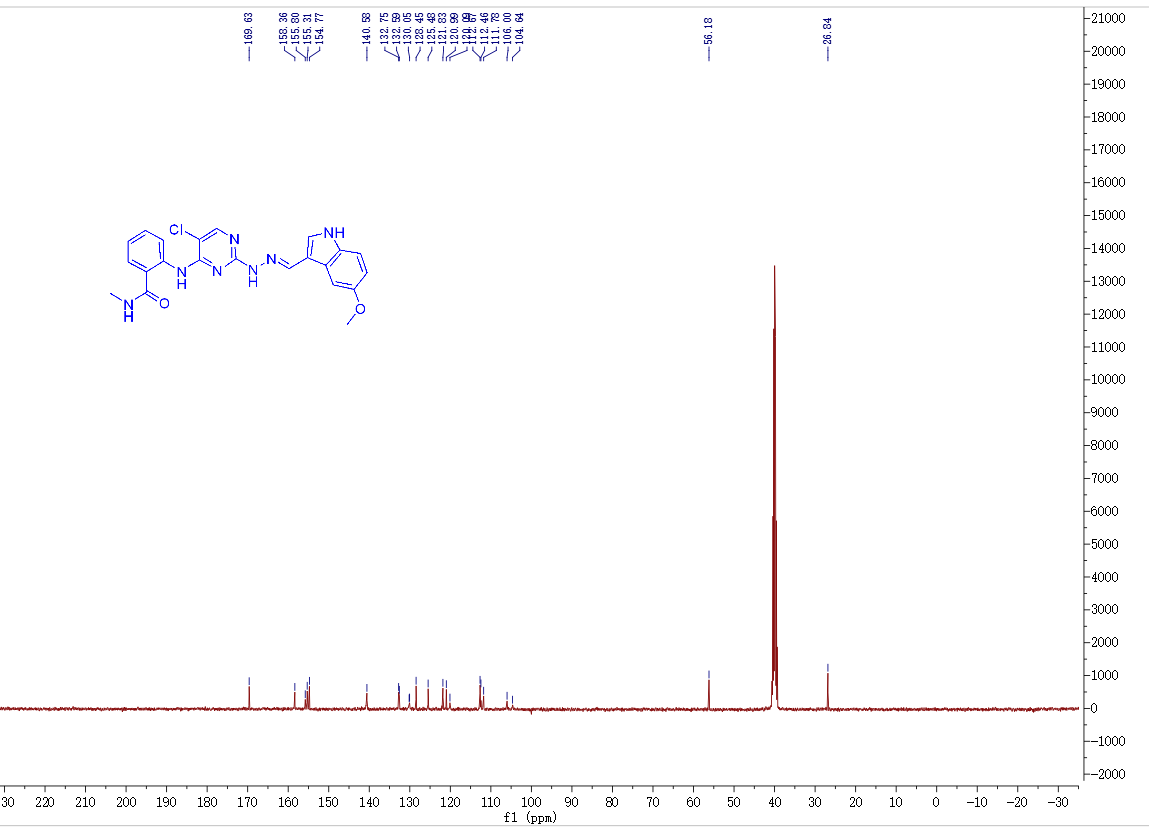


**Figure S76**. ^13^C NMR spectrum of compound 14z (100 MHz, DMSO-d6)


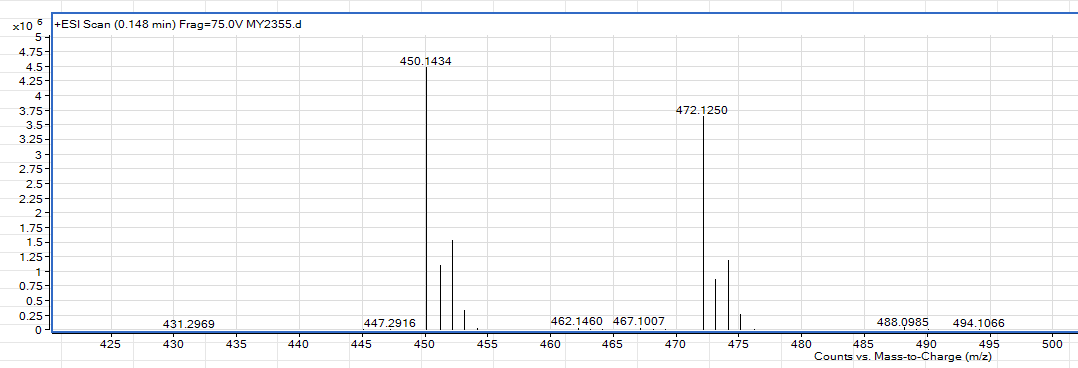


**Figure S77**. HRMS spectrum of compound 14z

- ^1^H, ^13^C-NMR and HRMS of compound 14aa


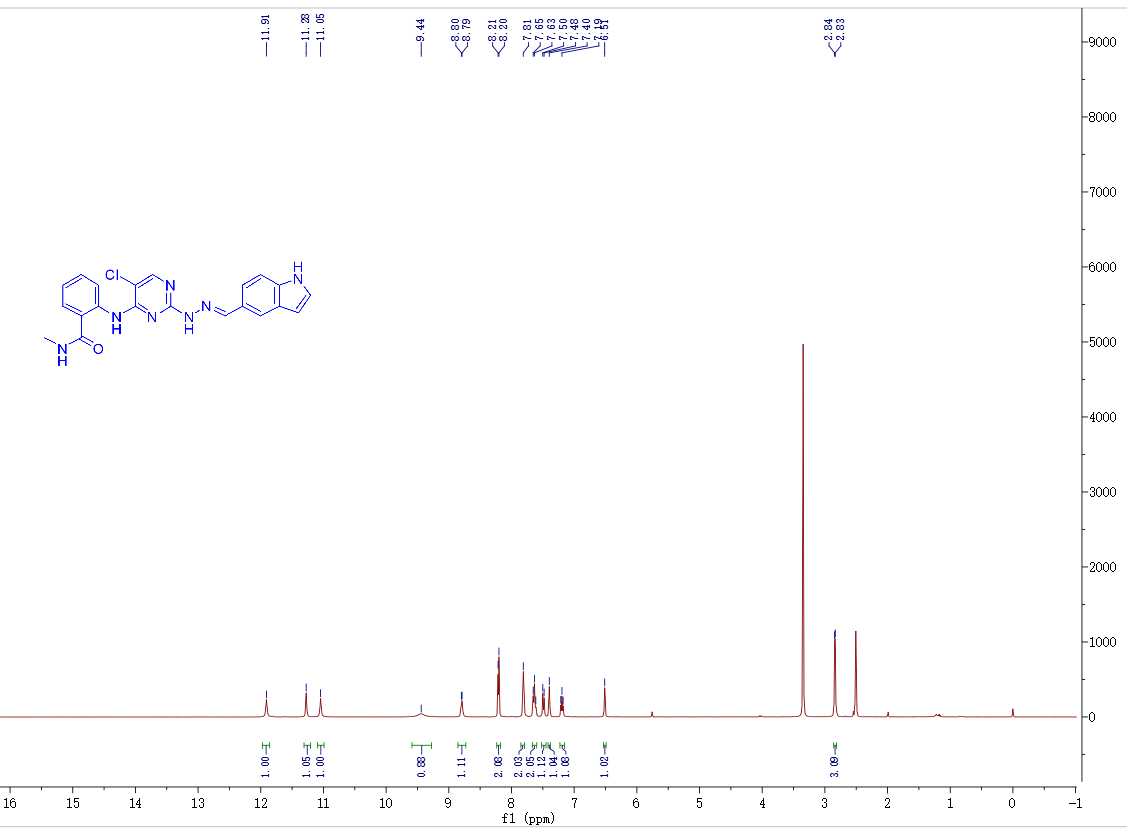


**Figure S75**. ^1^H NMR spectrum of compound 14aa (400 MHz, DMSO-d6)


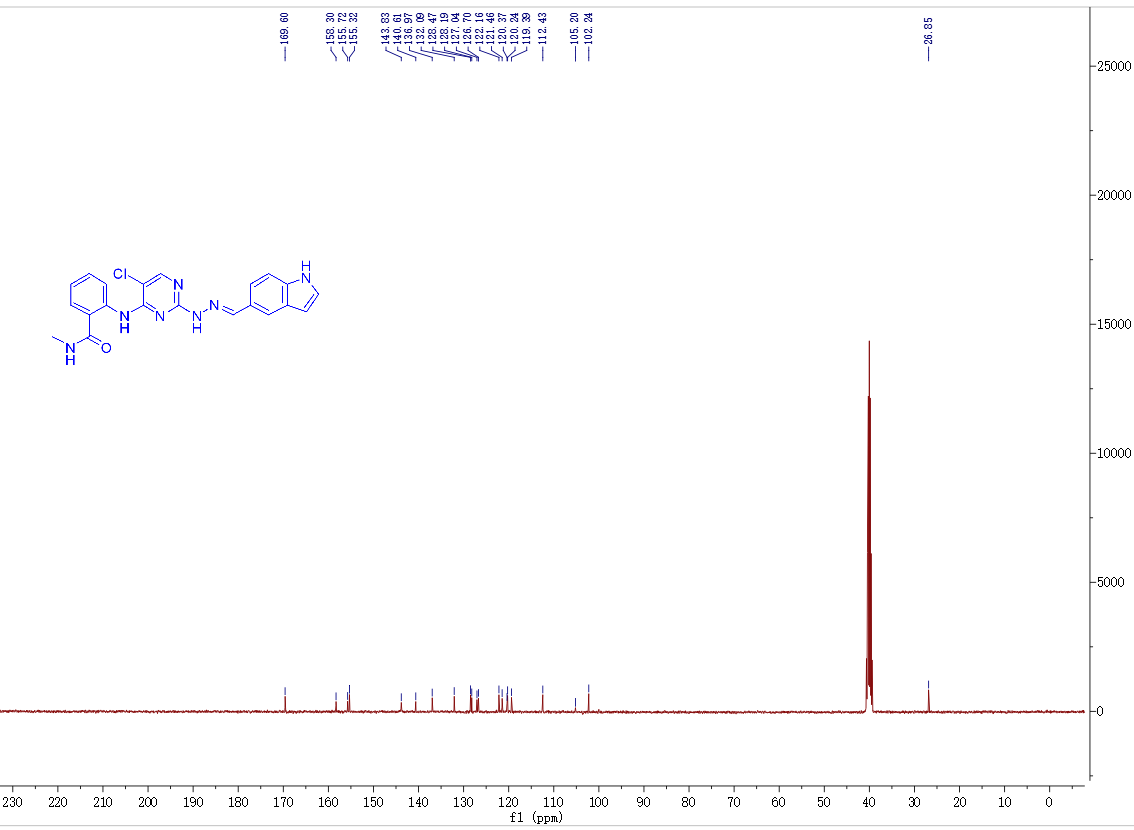


**Figure S76**. ^13^C NMR spectrum of compound 14aa (100 MHz, DMSO-d6)


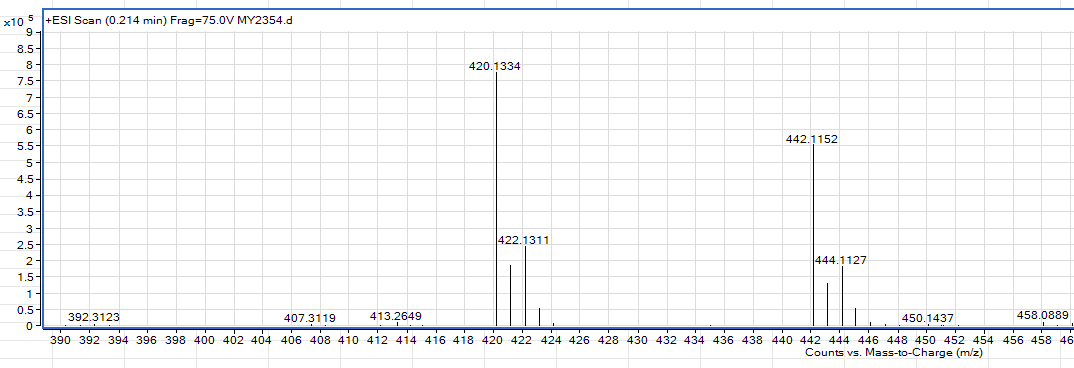


**Figure S77**. HRMS spectrum of compound 14aa

- ^1^H, ^13^C-NMR and HRMS of compound 14ab


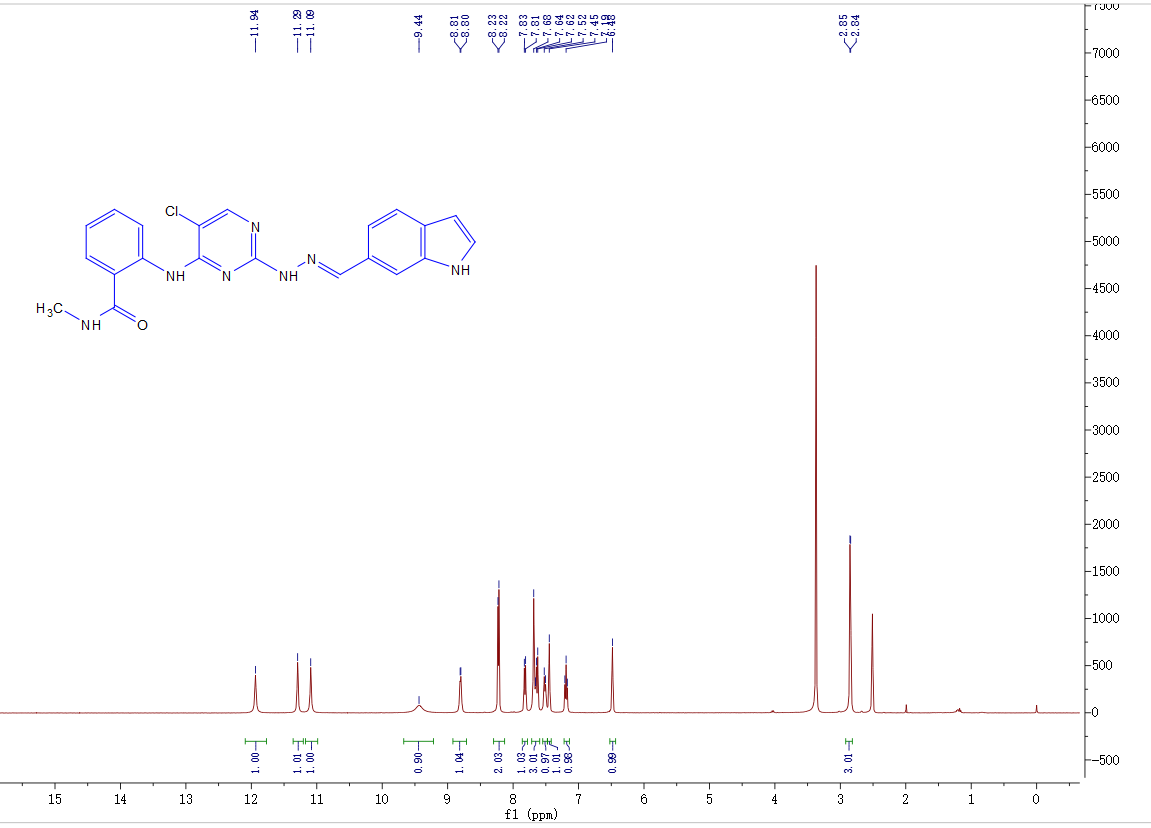


**Figure S78**. ^1^H NMR spectrum of compound 14ab (400 MHz, DMSO-d6)


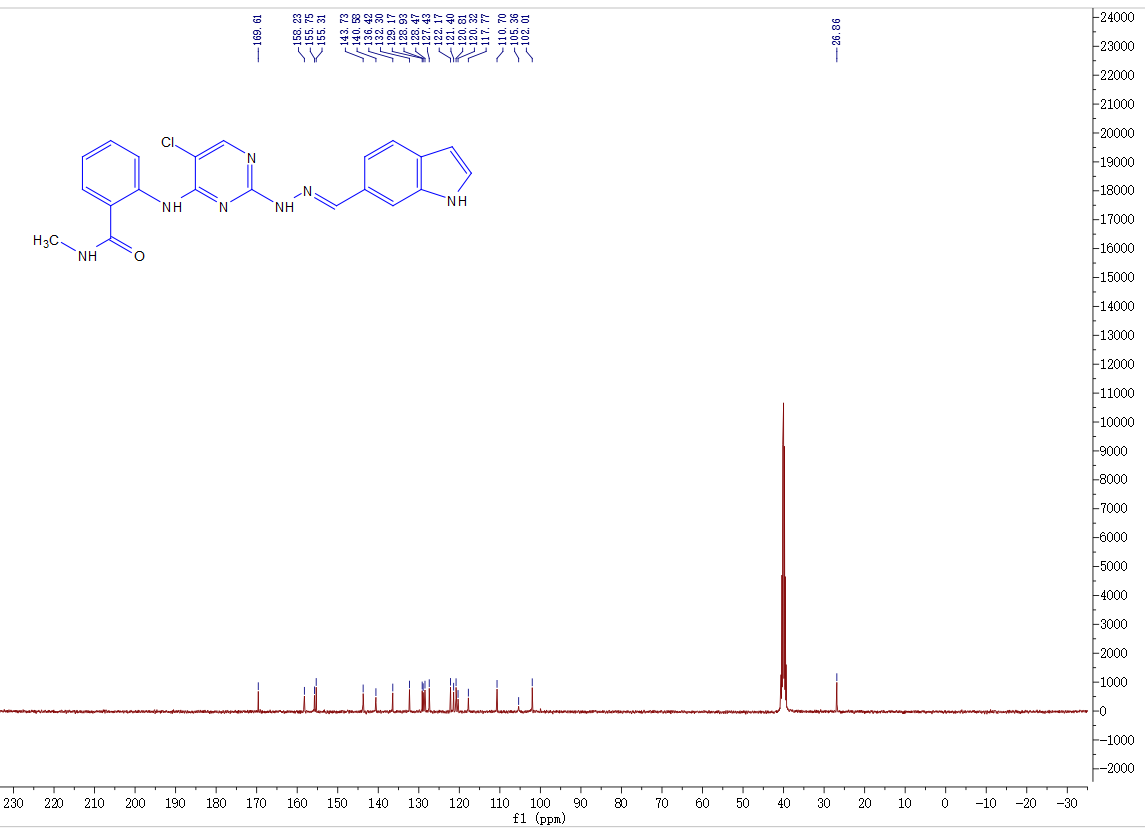


**Figure S79**. ^13^C NMR spectrum of compound 14ab (100 MHz, DMSO-d6)


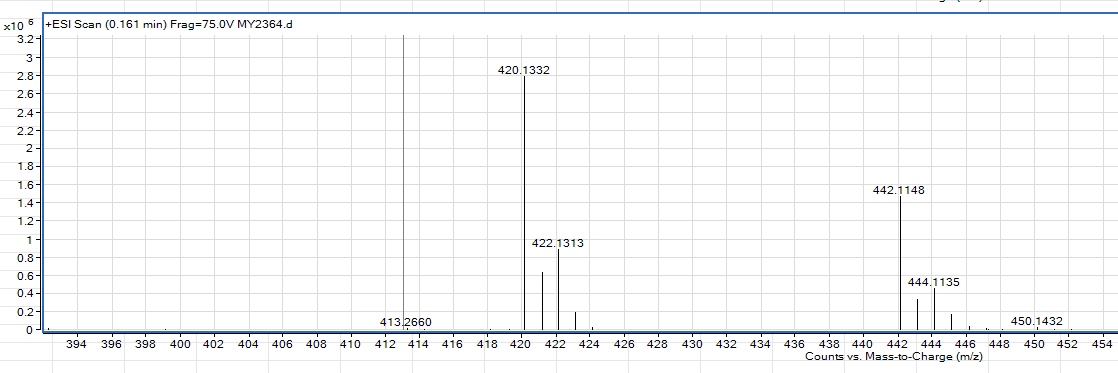


**Figure S80**. HRMS spectrum of compound 14ab

- ^1^H, ^13^C-NMR and HRMS of compound 14ac


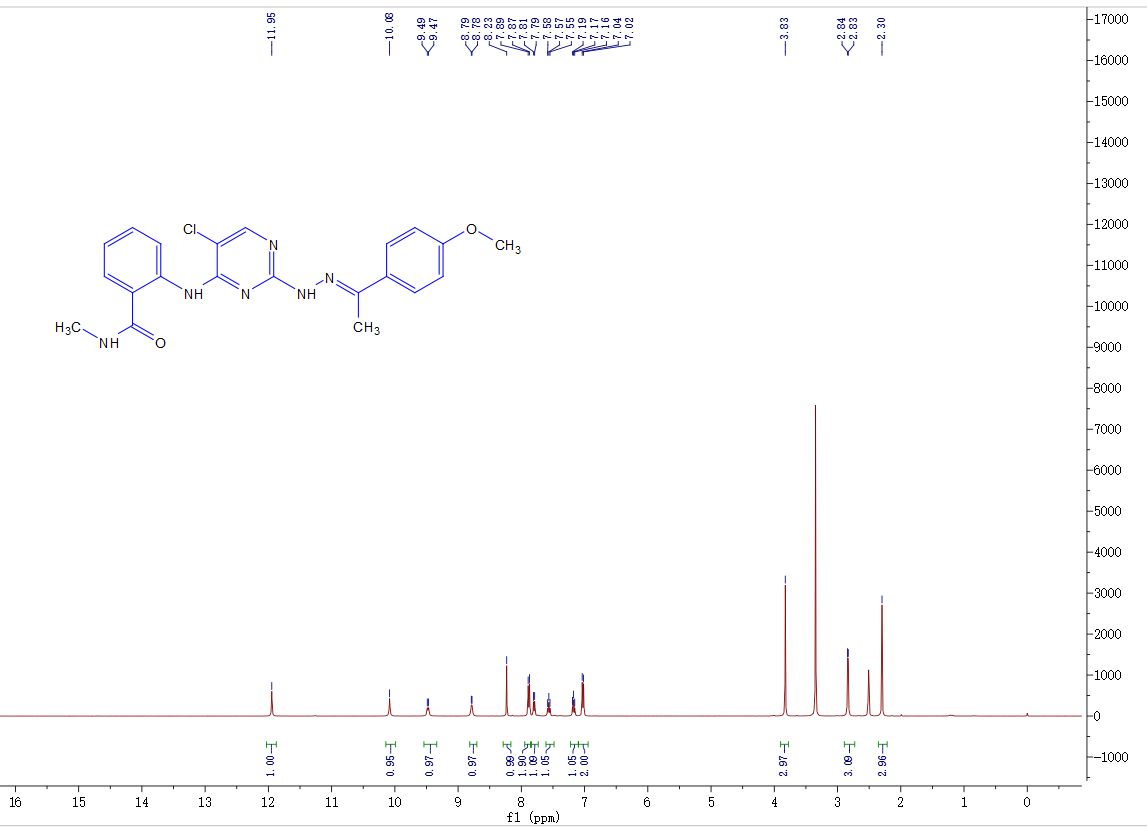


**Figure S81**. ^1^H NMR spectrum of compound 14ac (400 MHz, DMSO-d6)


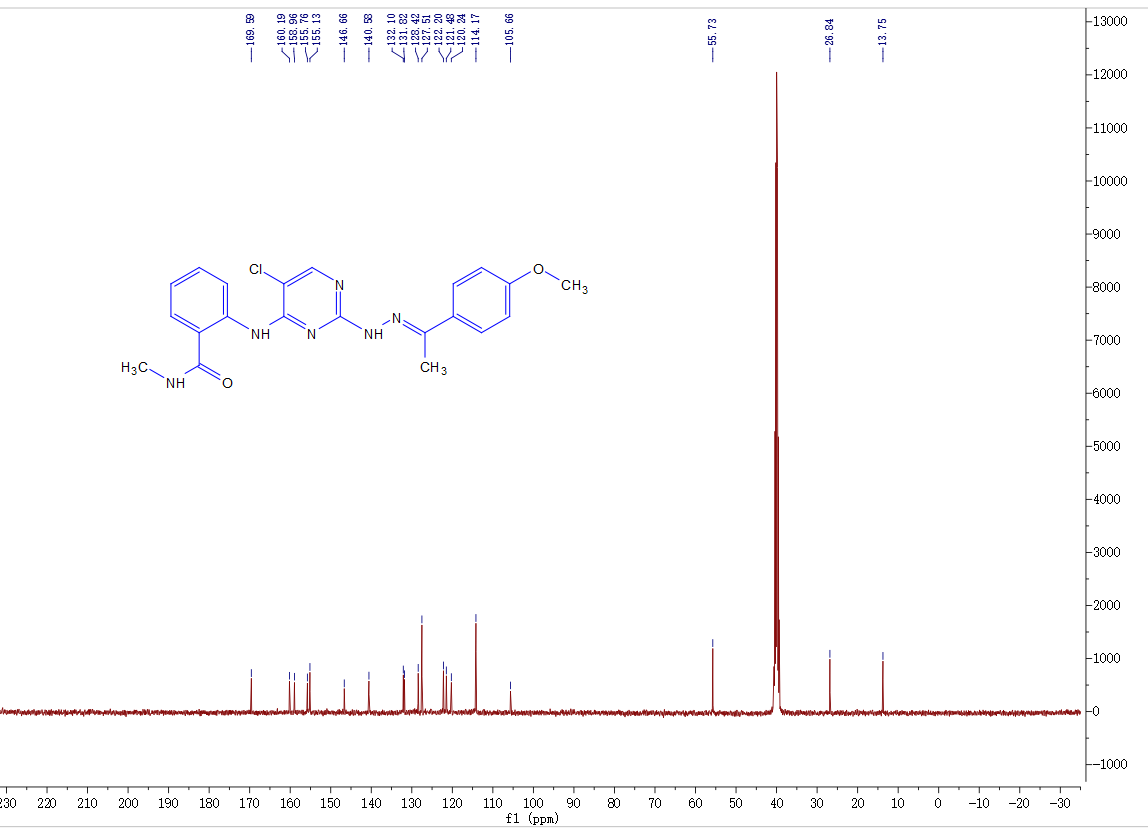


**Figure S82**. ^13^C NMR spectrum of compound 14ac (100 MHz, DMSO-d6)


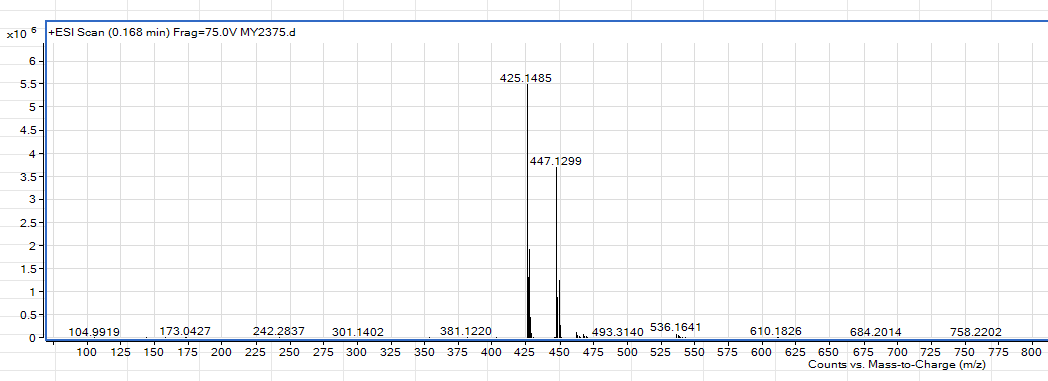


**Figure S83**. HRMS spectrum of compound 14ac

- ^1^H, ^13^C-NMR and HRMS of compound 14ad


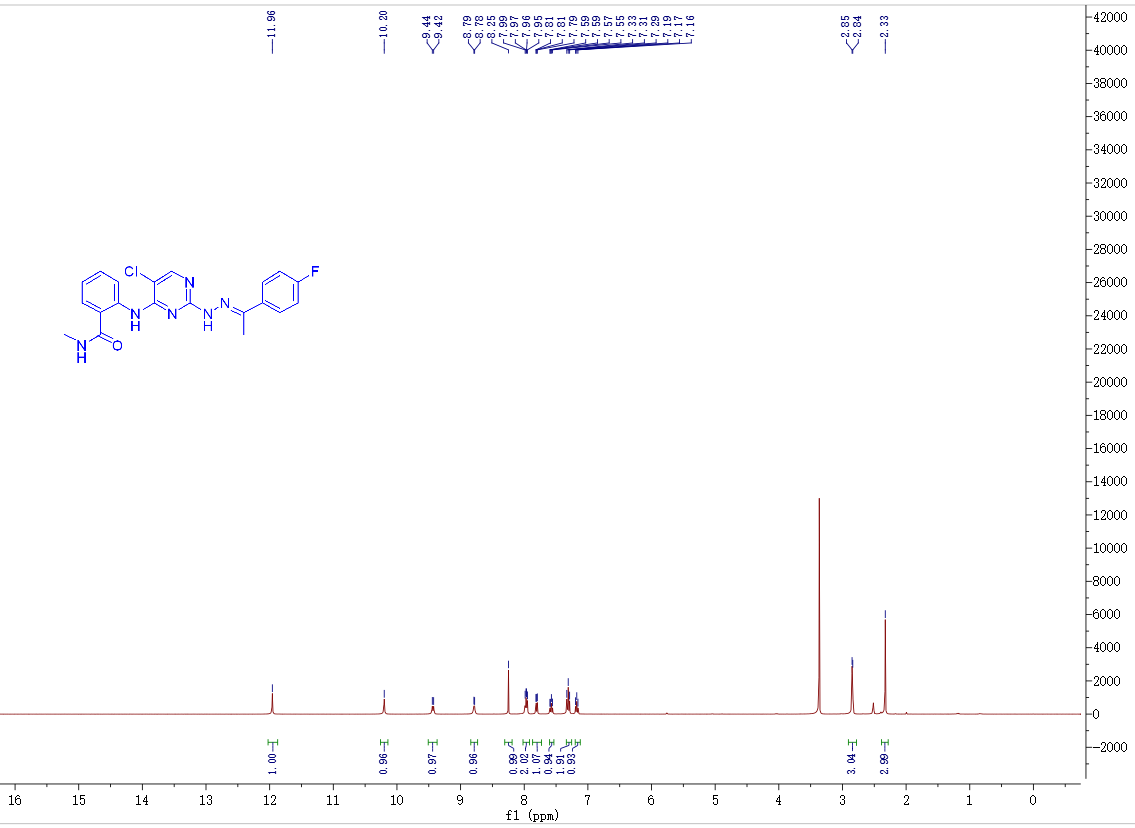


**Figure S84**. ^1^H NMR spectrum of compound 14ad (400 MHz, DMSO-d6)


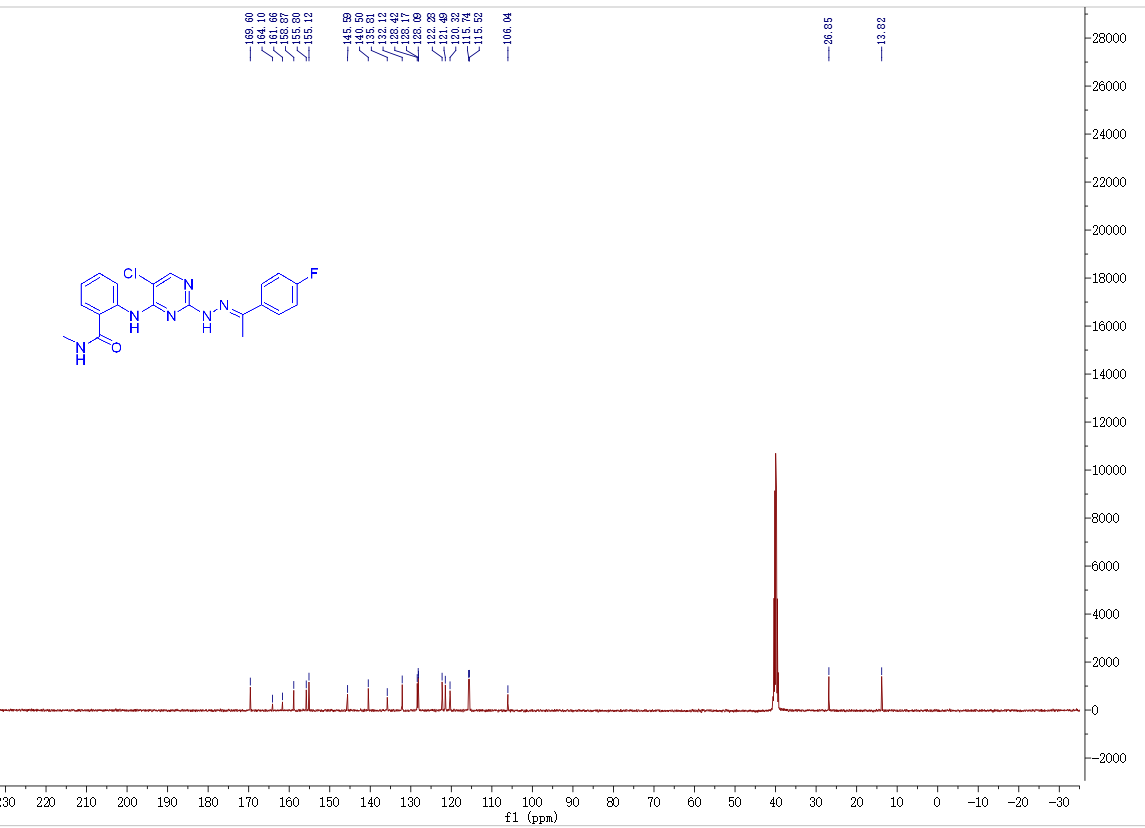


**Figure S85**. ^13^C NMR spectrum of compound 14ad (100 MHz, DMSO-d6)


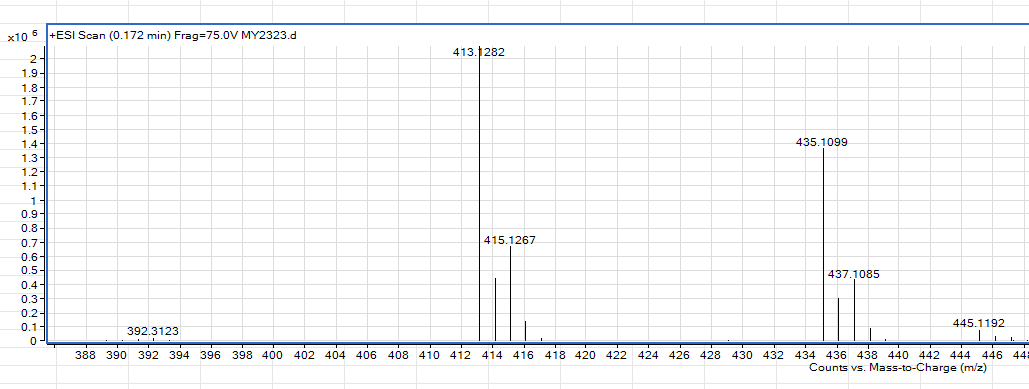


**Figure S86**. HRMS spectrum of compound 14ad
